# Ascorbic acid during the suckling period is required for proper DNA demethylation in the liver

**DOI:** 10.1038/s41598-020-77962-7

**Published:** 2020-12-04

**Authors:** Kenichi Kawahori, Yoshitaka Kondo, Xunmei Yuan, Yuki Kawasaki, Nozomi Hanzawa, Kazutaka Tsujimoto, Fumiko Wada, Takashi Kohda, Akihito Ishigami, Tetsuya Yamada, Yoshihiro Ogawa, Koshi Hashimoto

**Affiliations:** 1 Department of Molecular Endocrinology and Metabolism, Graduate School of Medical and Dental Sciences, Graduate School of Medical and Dental Sciences, Tokyo Medical and Dental University, Tokyo, 113-8510 Japan; 2 Molecular Regulation of Aging, Tokyo Metropolitan Institute of Gerontology, Tokyo, 173-0015 Japan; 3 Biomedical Gerontology Laboratory, Faculty of Human Sciences, Waseda University, Tokorozawa, 359-1192 Japan; 4 Department of Molecular and Cellular Metabolism, Graduate School of Medical and Dental Sciences, Tokyo Medical and Dental University, Tokyo, 113-8510 Japan; 5 Department of Epigenetics, Medical Research Institute, Tokyo Medical and Dental University, Bunkyo-ku, Tokyo, 113-8510 Japan; 6 Laboratory of Embryology and Genomics, Department of Biotechnology, Faculty of Life and Environmental Sciences, University of Yamanashi, Kofu, Yamanashi 400-8510 Japan; 7 Department of Medicine and Bioregulatory Science, Graduate School of Medical Sciences, Kyushu University, 3-1-1 Maidashi, Higashi-ku, Fukuoka, 812-8582 Japan; 8 Department of Preemptive Medicine and Metabolism, Graduate School of Medical and Dental Sciences, Tokyo Medical and Dental University, 1-5-45 Yushima, Bunkyo-ku, Tokyo, 113-8510 Japan; 9 Department of Diabetes, Endocrinology and Hematology, Dokkyo Medical University Saitama Medical Center, 2-1-50 Minami-Koshigaya, Koshigaya, Saitama 343-8555 Japan

**Keywords:** Developmental biology, Genetics, Molecular biology

## Abstract

Ascorbic acid (AA, vitamin C) serves as a cofactor for ten-eleven translocation (TET) enzymes and induces DNA demethylation in vitro. However, its role in DNA demethylation in vivo remains unclear. We previously reported that DNA demethylation in the mouse liver was enhanced during the suckling period. Therefore, we hypothesized that DNA demethylation is enhanced in an AA-dependent manner during the suckling period. To examine our hypothesis, we employed wild-type (WT) mice, which synthesize AA, and senescence marker protein-30/gluconolactonase (SMP30/GNL) knockout (KO) mice, which cannot synthesize AA, and analyzed the DNA methylation status in the livers of offspring in both the suckling period and adulthood. SMP30/GNL KO offspring showed DNA hypermethylation in the liver possibly due to low plasma and hepatic AA levels during the suckling period despite the administration of rescue-dose AA to dams. Furthermore, DNA hypermethylation of the fibroblast growth factor 21 gene (*Fgf21*), a PPARα target gene, persisted into adulthood. In contrast, a high-dose AA administration to SMP30/GNL KO dams during the lactation period restored DNA demethylation in the livers of offspring. Even though a slight increase was observed in plasma AA levels with the administration of rescue-dose AA to WT dams during the gestation and lactation periods, DNA demethylation in the livers of offspring was minimally enhanced. The present results demonstrate that AA intake during the suckling period is required for proper DNA demethylation in the liver.

## Introduction

Ten-eleven translocation (TET) enzymes catalyze the oxidation of 5-methylcytosine (5mC) to 5-hydroxymethylcytosine (5hmC)^1–3^. Ascorbic acid (AA, vitamin C) promoted DNA demethylation in embryonic stem cells and induced pluripotent stem cells^4,5^ and was also shown to enhance 5hmC levels in a TET-dependent manner^6–8^. Recent studies on humans suggested that plasma AA concentrations are related to aberrant DNA methylation in genomic DNA derived from peripheral leukocytes in patients with some cancers^9,10^. However, it currently remains unclear whether AA affects DNA methylation patterns in vivo.

Humans, other primates, and animal species, such as Guinea pigs and fruit bats, lack the enzyme L-gulonolactone oxidase, which catalyzes the final step in AA biosynthesis; therefore, they are incapable of synthesizing AA^11^. Senescence marker protein-30 (SMP30) is involved in AA biosynthesis in non-primate mammals^12,13^. We previously reported that SMP30 served as a gluconolactonase (GNL), which catalyzed the penultimate step of the AA biosynthesis pathway^13,14^. Therefore, SMP30/GNL-deficient (knockout: KO) mice fed an AA-free diet exhibited the symptoms of scurvy. Moreover, an insufficient AA intake during the gestation period induced dilated cardiomyopathy in perinatal SMP30/GNL KO mice^15^. Therefore, SMP30/GNL KO mice are suitable for investigating the physiological functions of AA in vivo.

Despite the importance of AA in development in childhood^15–18^, the molecular mechanisms by which AA affects development remain unclear. We previously reported the marked DNA demethylation of the fibroblast growth factor 21 gene (*Fgf21*), a peroxisome proliferator-activated receptor (PPAR) α target gene, in the liver during the suckling period^19^. In recent years, AA has been shown to affect DNA demethylation in vitro, whereas its effects in vivo are still unknown.

In the present study, we focused on the potential role of AA in DNA demethylation in the liver. We investigated whether AA affects DNA demethylation in the mouse liver during the suckling period. Using a genome-wide analysis of DNA methylation, we identified genes susceptible to AA-dependent DNA demethylation in the liver during the suckling period. We also demonstrated that the DNA methylation status of *Fgf21*, a PPARα target gene, in early life was maintained in later life.

## Results

### SMP30/GNL KO mice exhibited DNA hypermethylation in livers of offspring due to low plasma and hepatic AA levels during the suckling period

SMP30/GNL KO mice, which cannot synthesize AA, were crossed to obtain SMP30/GNL KO offspring. We previously reported that SMP30/GNL KO offspring were born and matured normally using free access to water containing 1.5 g/L AA, defined as a rescue dose to avoid the effects of an AA deficiency^20^, for dams and offspring^14^. Therefore, we administered 1.5 g/L AA water to SMP30/GNL KO dams during the gestation and lactation periods. In a preliminary experiment, we assessed AA levels in milk obtained from the gastric contents of offspring 16 days after birth (d16), which was derived from AA supplemented and unsupplemented KO dams during the lactation period (Supplementary Fig. 1a). We found that AA levels, including AA and dehydroascorbic acid (DHA), the oxidized form of AA, in milk from supplemented KO dams were significantly higher than in that from unsupplemented dams (Supplementary Fig. 1b). These results suggested that the administration of 1.5 g/L AA water to SMP30/GNL KO dams during the lactation period significantly increased AA levels in milk.

**Figure 1:**
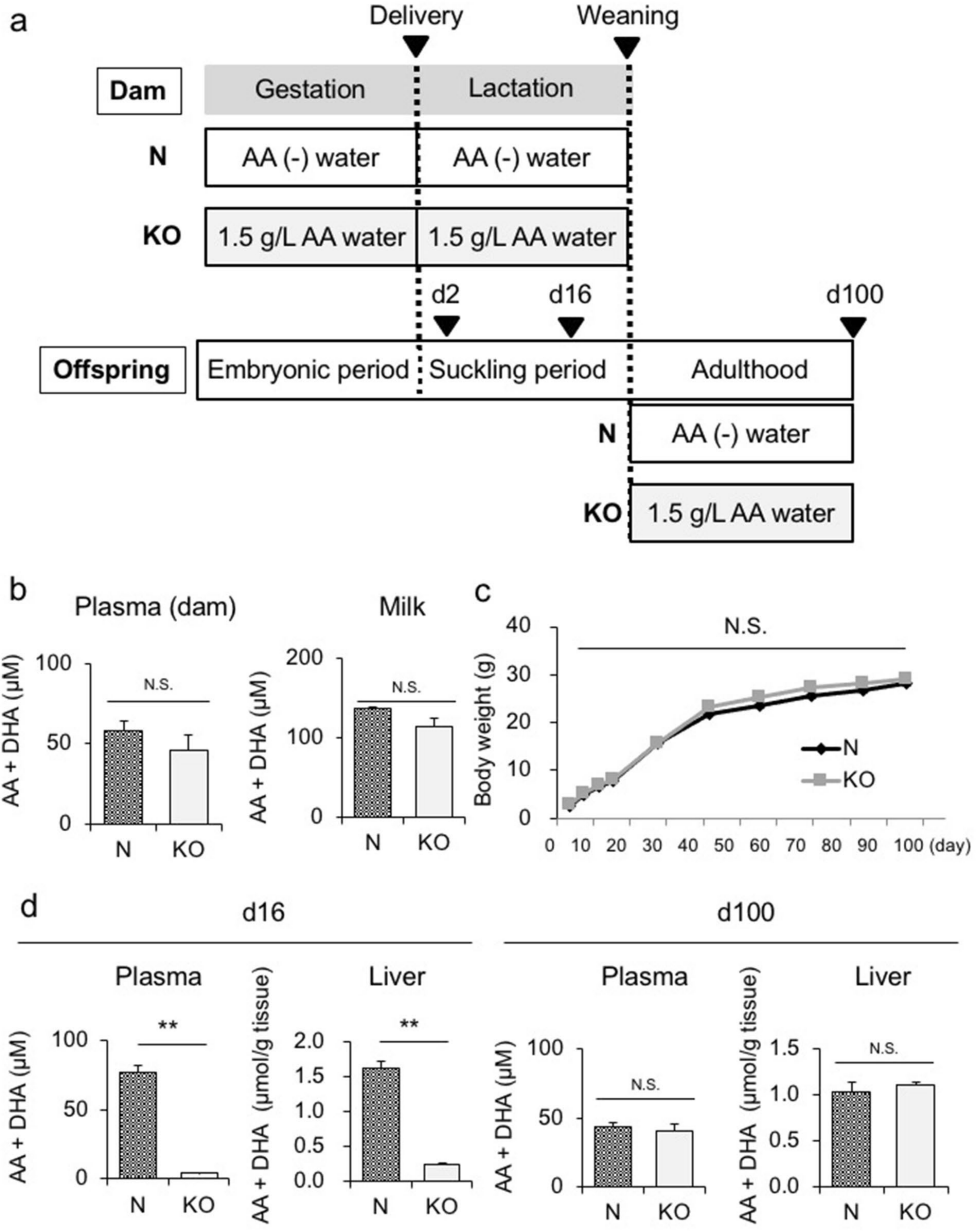
Analysis of SMP30/GNL KO offspring. **(a)** Experimental protocol to analyze WT (N) and SMP30/GNL KO (KO) offspring. Free access to water containing 1.5 g/L AA was given to SMP30/GNL KO dams and offspring throughout the experiment, whereas N dams and offspring were fed AA-free water. (**b**) Plasma and milk AA levels of dams in the KO and N groups on d2 (n = 6–8 per group). (**c**) Body weights of KO and N offspring from birth to d100 (n = 6 per group, statistical analysis using a two-way ANOVA with Tukey’s multiple comparison test). (**d**) Plasma and hepatic AA levels in KO and N offspring on d16 (left panel) and d100 (right panel) (n = 6 per group). Statistical analysis using an unpaired Student’s *t*-test unless otherwise indicated. Data are expressed as the mean ± SEM. ** *p* < 0.01; N.S., not significant vs. KO offspring.

Upon weaning, we administered AA-free water and 1.5 g/L AA water to wild-type (WT) (N) and SMP30/GNL KO (KO) offspring, respectively (Fig. 1a). We examined N and KO offspring in both the suckling period (d16) and in adulthood (d100). As shown in Fig. 1b, no significant differences were observed in plasma or milk AA levels between N and KO dams upon delivery (d2). The body weights of offspring in both groups were similar from birth to adulthood (Fig. 1c). Moreover, the liver weights of offspring in both groups were similar on d16 and d100 (Supplementary Fig. 2a). However, the plasma and hepatic AA levels on d16 were significantly higher in N offspring than in KO offspring (Fig. 1d, left panel). On the other hand, in adulthood (d100), no significant differences were noted in plasma or hepatic AA levels between N and KO offspring (Fig. 1d, right panel). Collectively, these results indicated that AA levels were significantly lower in KO offspring than in N offspring predominantly during the suckling period.

**Figure 2:**
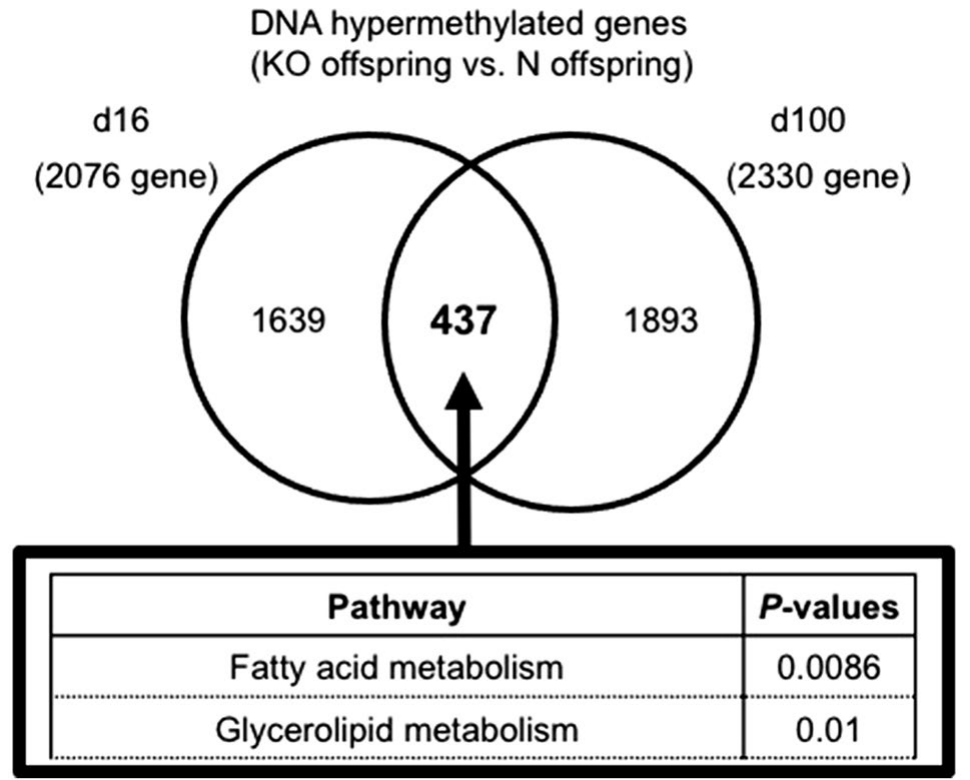
Venn diagram showing results of MIAMI. **(a)** We identified 437 genes that were DNA hypermethylated in KO offspring relative to those in N offspring on both d16 and d100. We performed a pathway analysis with these genes.

We also employed a Microarray-based Integrated Analysis of Methylation by Isoschizomers (MIAMI)^21^ to examine the genome-wide DNA methylation status of the livers of N and KO offspring. On d16, KO offspring had more hypermethylated genes than N offspring, which was also confirmed on d100. More than 2000 genes were hypermethylated in KO offspring relative to those in N offspring on both d16 and d100 (Table 1a, Supplementary Table 1a–d). These results suggested that AA affected DNA demethylation in the liver during the suckling period, and that the administration of 1.5 g/L AA water to KO dams was insufficient to induce physiological levels of DNA demethylation in the livers of KO offspring. A pathway analysis of these genes yielded cell proliferation- and differentiation-related pathways and glucose- or lipid metabolism-related pathways (Table 1b). We identified 437 genes that were hypermethylated in KO offspring relative to those in N offspring on both d16 and d100 (Fig. 2, Supplementary Table 2). We also found 9 PPAR α target genes that were hypermethylated on both d16 and d100 (Table 2), and a pathway analysis of the 437 genes confirmed that the fatty acid metabolism pathway was significant, which included *Fgf21* (Fig. 2, Table 2), a gene that was reportedly subjected to PPARα-dependent DNA demethylation during the suckling period^19,22^.

**Table 1:** (a) The number of genes with DNA methylation changes in the liver of KO offspring relative to those in N offspring on d16 and d100. (b) Pathway analysis of genes that were DNA hypermethylated in KO offspring relative to those in N offspring on d16 (top) and d100 (bottom).

	Number of genes		
	Total number DNA	DNA hypomethylation	DNA hypermethylation
**(a)**			
**d16**	27,110	361	2076
**d100**	28,347	574	2330

**(b) d16**			
Pathway	*P* values		
Glycerolipid metabolism	3.8E-4		
mTOR signaling pathway	4.7E-3		
Insulin signaling pathway	5.4E-3		
ErbB signaling pathway	5.6E-3		
Adherens junction	1.0E-2		
Neurotrophin signaling pathway	1.2E-2		
Axon guidance	1.3E-2		
PPAR signaling pathway	1.4E-2		
Glycine, serine and threonine metabolism	1.5E-2		
Fatty acid metabolism	3.2E-2		

**(c) d100**			
Pathway	*P *values		
Wnt signaling pathway	2.3E-5		
Pathway in cancer	2.6E-5		
Basal cell carcinoma	3.6E-5		
TGF-beta signaling pathway	5.1E-5		
Insulin signaling pathway	2.8E-3		
Neurotrophin signaling pathway	2.2E-2		
Glycosphingolipid biosynthesis	2.3E-2		
MAPK signaling pathway	3.1E-2		
mTOR signaling pathway	3.1E-2

**Table 2:** PPARα target genes that were DNA hypermethylated on both d16 and d100 (KO offspring vs. N offspring).

Gene symbol	Gene name
*Cptla*	carnitine palmitoyltransferase 1a, muscle
*Fgf21*	fibroblast growth factor 21
*Acaala*	acetyl-Coenzyme A acyltransferase 1A
*Ehhadh*	enoyl-CoA hydratase and 3-hydroxyacyl CoA dehydrogenase
*Peci*	penoyl-Coenzyme A delta isomerase 2
*Aldh3a2*	aldehyde dehydrogenase family 3, subfamily A2
*Agpat2*	1-acylglycerol-3-phosphate O-acyltransferase 2
*Srebfl*	sterol regulatory element binding transcription factor 1
*Pnpla2*	patatin-like phospholipase domain containing 2

**Figure 3:**
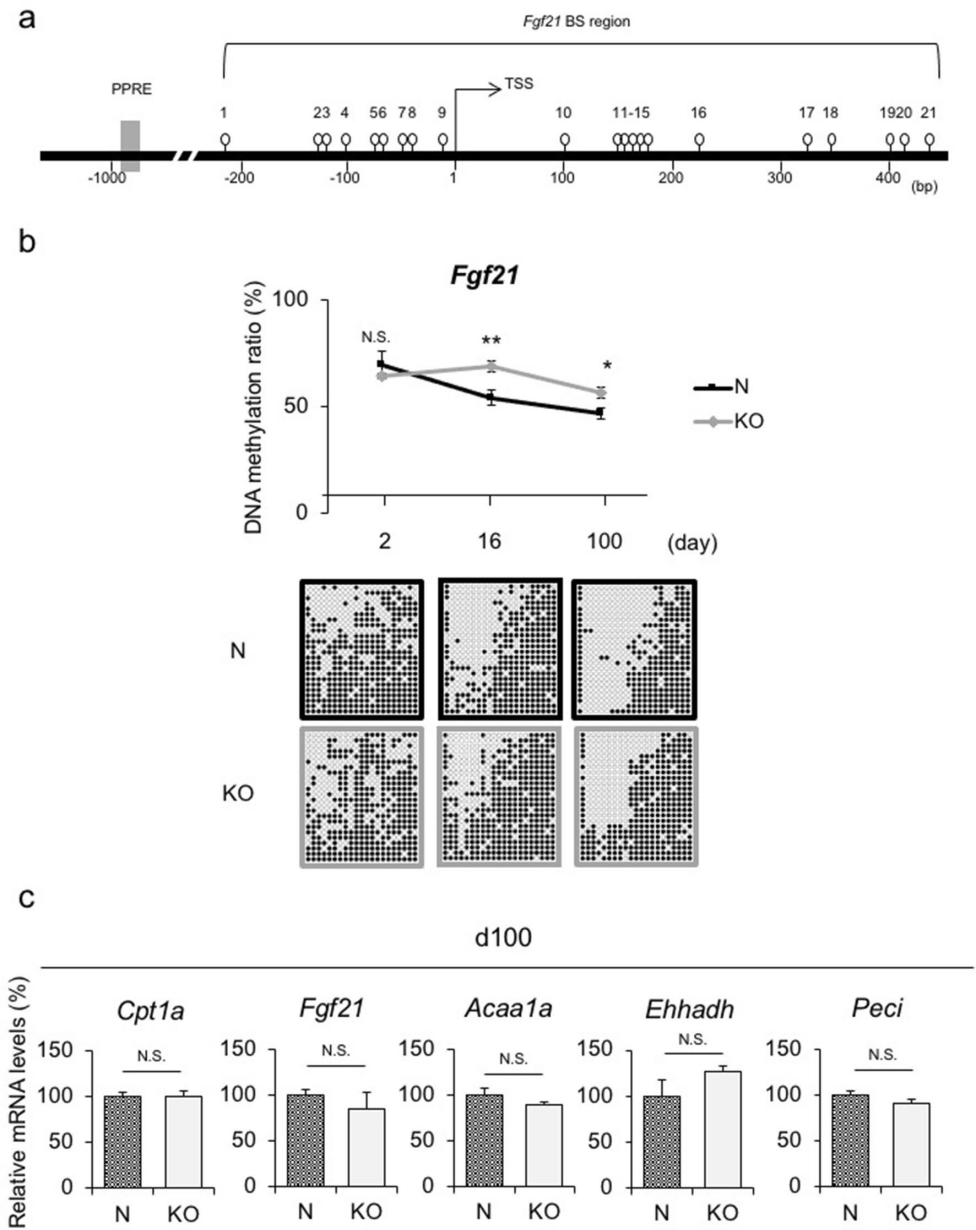
DNA methylation status and gene expression levels of PPARα target genes in livers of KO and N offspring. **(a)** Schematic representation of the promoter region of *Fgf21*. Open circles and gray boxes indicate CpG sites and PPAR response elements (PPREs), respectively. The bisulfite-sequencing (BS) analysis region encompassing the transcription start site (TSS) is indicated. (**b**) *Fgf21* DNA methylation status in the livers of N and KO offspring via the BS analysis (n = 4 per group, statistical analysis using a one-way ANOVA with Tukey’s multiple comparison test). Representative BS data are depicted below the graph. Data are expressed as the mean ± SEM. *p < 0.05; ** *p* < 0.01; N.S., not significant vs. KO offspring. (**c**) Hepatic *Cpt1a*, *Fgf21*, *Acaa1a*, *Ehhadh*, and *Peci* mRNA expression levels on d16, which were DNA hypermethylated on both d16 and d100. (n = 6 per group). Statistical analysis using an unpaired Student’s *t*-test unless otherwise indicated. Data are expressed as the mean ± SEM. N.S., not significant vs. KO offspring.

We previously identified *Fgf21* as an epigenetic memory gene that plays a pivotal role in the developmental programming of obesity^22^. In silico searches revealed 21 sites for CpG dinucleotides (i.e., cytosine followed by guanine) for DNA methylation around the transcription start site (TSS) of *Fgf21* (Fig. 3a)^22,23^. To verify whether *Fgf21* DNA demethylation physiologically occurred in an AA-dependent manner, we examined the DNA methylation status of the *Fgf21* promoter region in the livers of N and KO offspring using a bisulfite-sequencing analysis. The results of the bisulfite-sequencing analysis revealed that the difference in the *Fgf21* DNA methylation status on d16 between N and KO offspring remained until d100 (Fig. 3b). We also evaluated the DNA methylation status at each CpG site in the *Fgf21* promoter region on d2, d16, and d100 and found some CpG sites at which the DNA methylation rate in KO offspring was significantly higher than that in N offspring (Supplementary Fig. 3). However, no specific regulatory elements were detected at these CpG sites.

On the other hand, DNA demethylation did not increase lipid metabolism-related gene expression, including *Fgf21*, in the livers of N offspring at a steady state (Fig. 3c). Accordingly, no significant differences were observed in serum triglyceride (TG), total cholesterol (T-Chol), non-esterified fatty acids (NEFA), or blood glucose levels between N and KO offspring on d100 (Supplementary Fig. 2b).

### Administration of high-dose AA to SMP30/GNL KO dams during the lactation period restored DNA demethylation in livers of offspring

**Figure 4:**
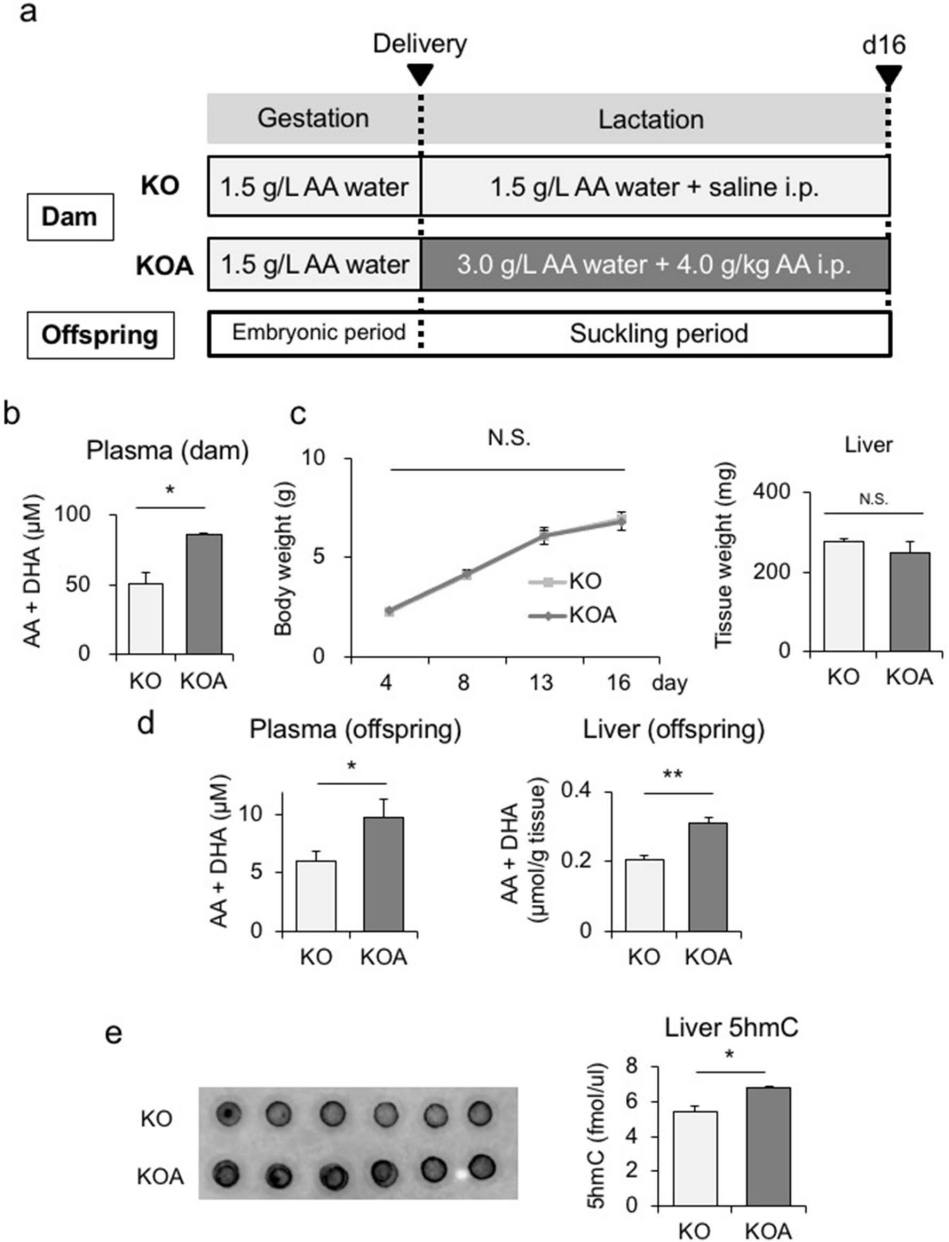
Maternal administration of high-dose AA to SMP30/GNL KO dams during the lactation period. (**a**) Experimental protocol of the maternal administration of high-dose AA to SMP30/GNL KO dams during the lactation period. Upon delivery (d2), KO dams were divided into the KO and KOA groups. The KO group had free access to 1.5 g/L AA water, whereas the KOA group was given 3.0 g/L AA water orally and injected intraperitoneally (i.p.) with 4.0 g/L AA. Upon birth, during the lactation period, male offspring were divided into the two groups as above. Plasma AA levels in dams on d2 (n = 6 per group). (**c**) Body weights of KO and KOA offspring from birth to d16 (left panel, statistical analysis using a one-way ANOVA with Tukey’s multiple comparison test) and liver weight on d16 (right panel) (n = 6 per group). (**d**) Plasma and hepatic AA levels in KO and KOA offspring on d16 (n = 6–8 per group). (**e**) 5hmC levels in the livers of KO and KOA offspring on d16 (n = 6 per group). Statistical analysis using an unpaired Student’s *t*-test unless otherwise indicated. Data are expressed as the mean ± SEM. *p < 0.05; ** *p* < 0.01; N.S., not significant vs. KOA offspring.

Based on the results obtained, we administered high-dose AA to KO dams during the lactation period as a second experiment to clarify whether DNA demethylation in KO offspring was restored. Upon delivery (d2), KO dams were divided into the KO and KOA groups. The KO group had free access to 1.5 g/L AA water, whereas the KOA group was given 3.0 g/L AA water orally and injected intraperitoneally (i.p.) with 4.0 g/L AA (Fig. 4a). On d2, plasma AA levels were significantly higher in KOA dams than in KO dams (Fig. 4b). Body and liver weights in both groups were similar between d4 and d16 (Fig. 4c). However, plasma and hepatic AA levels were significantly higher in KOA offspring than in KO offspring on d16 (Fig. 4d).

To clarify whether DNA demethylation in KOA offspring was induced by increased TET catalysis, we analyzed 5hmC levels in the livers of KOA and KO offspring on d16. The results obtained showed that 5hmC levels in the liver were significantly higher in KOA offspring than in KO offspring (Fig. 4e). These results indicated that the activation of TET led to the DNA demethylation of genes in the livers of KOA offspring.

MIAMI of the offspring on d16 revealed that more genes were hypomethylated in KOA offspring than in KO offspring. In total, 716 and 172 genes were hypomethylated and hypermethylated, respectively, in KOA offspring relative to those in KO offspring, suggesting that the high-dose AA administration to KOA dams restored DNA demethylation in the livers of their offspring (Table 3a, Supplementary Table 3a, b). We performed a pathway analysis of the 716 genes that were hypomethylated in KOA offspring, which yielded Wnt, TGF-β, Jak-STAT, and insulin pathways with significant differences (Table 3b, Supplementary Table 3a). We also found 5 PPAR α target genes among the 716 genes (Table 3c). However, *Fgf21* was not included among the 5 genes.

**Table 3:** (a) The number of genes with DNA methylation changes in the liver of KOA offspring relative to those in KO offspring on d16. (b) Pathway analysis of genes that were DNA hypomethylated in KOA offspring relative to those in KO offspring on d16. (c) PPAR target genes that were DNA hypomethylated on d16 (KOA offspring vs. KO offspring).

	Number of genes		
	Total number	DNA hypomethylation	DNA hypermethylation
**(a) d16**	29,307	716	172

**(b)**			
Pathway	*P* values		
Wnt signaling pathway	2.5E-5		
Axon guidance	3.2E-4		
TGF-beta signaling pathway	7.3E-3		
Cell cycle	8.9E-3		
Jak-STAT signaling pathway	1.1E-2		
MAPK signaling pathway	1.3E-2		
Insulin signaling pathway	3.7E-2		

**(c)**			
Gene symbol	Gene name		
*Acot7*	acyl-CoA thioesterase 7		
*Abcg8*	ATP binding cassette subfamily G member 8		
*Scarb2*	Scavenger receptor class B, member 2		
*Srebf1*	Sterol regulatory element binding transcription factor 1		
*Ifi47*	Interferon gamma-inducible protein 47		

We identified 163 common genes between the 716 genes hypomethylated in KOA offspring and the 2076 genes hypermethylated in the KO offspring relative to those in wild-type (N) offspring on d16 (Table 1 and Supplementary Table 1b). These 163 genes were presumably hypermethylated in KO offspring relative to those in N offspring and hypomethylated by the high-dose AA supplementation (Supplementary Table 4).

We also performed MIAMI to compare the DNA methylation status in the livers of KOA and N offspring and identified 1034 genes that were hypermethylated in KOA offspring relative to those in N offspring on d16, similar to KO offspring (Supplementary Table 5a, 6a,b). A pathway analysis of the 1034 genes revealed cell proliferation and differentiation-related pathways and the glucose or lipid metabolism-related pathway, which was similar to KO offspring shown in Table 1 (Supplementary Table 5b).

### Rescue-dose AA administration did not exert an additive effect on DNA demethylation in livers of WT mice

**Figure 5:**
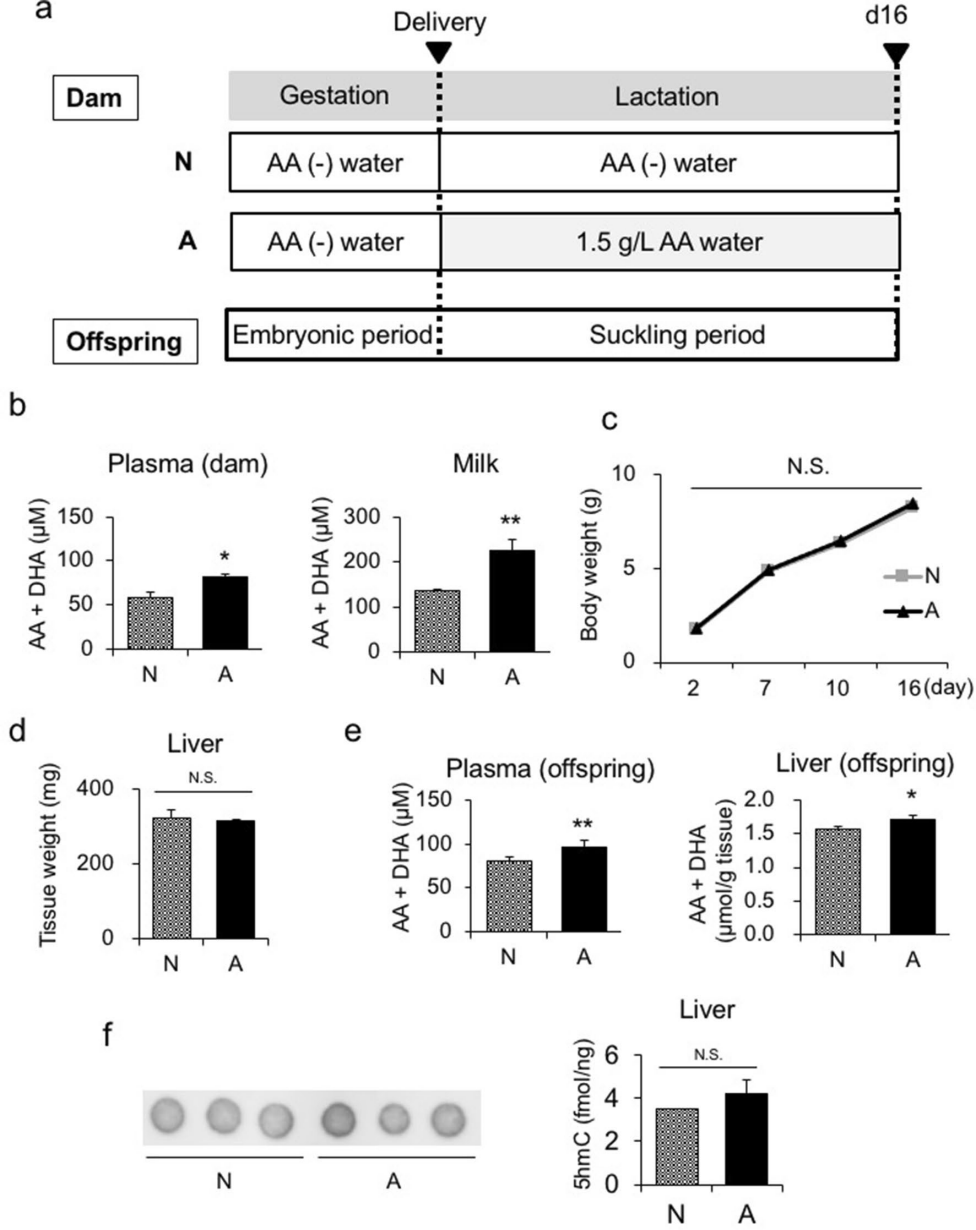
Maternal administration of rescue-dose AA to WT dams during the lactation period. (**a**) Experimental protocol of the maternal administration of AA to WT dams during the lactation period. Upon delivery (d2), dams were divided into two groups; one was fed AA-free water, referred to as the N group, and the other was fed 1.5 g/L AA water as the A group. Upon birth, during the lactation period, male offspring were divided into the two groups as above. (**b**) Plasma AA levels in dams on d16 (n = 6–8 per group). (**c**) Body weights of N and A offspring from birth to d16 (left panel, statistical analysis using a one-way ANOVA with Tukey’s multiple comparison test) and (**d**) liver weight on d16 (right panel) (n = 6–8 per group). (**e**) Plasma and hepatic AA levels in N and A offspring on d16 (n = 6–8 per group). (**f**) 5hmC levels in the livers of N and A offspring on d16 (n = 3 per group). Statistical analysis using an unpaired Student’s *t*-test unless otherwise indicated. Data are expressed as the mean ± SEM. *p < 0.05; ** *p* < 0.01; N.S., not significant vs. A offspring.

As described above, WT mice are capable of synthesizing AA, in contrast to humans and Guinea pigs. In the third experiment, we administered AA-free water and diet to WT dams until delivery. Upon delivery, during the lactation period, dams were divided into two groups: one was fed AA-free water and the other was fed 1.5 g/L AA water. Male offspring of the dams fed AA-free water and 1.5 g/L AA water were referred to as N and A offspring, respectively (Fig. 5a). On d16, the plasma and milk AA levels of dams were significantly higher in the A group than in the N group (Fig. 5b). Body and liver weights in both groups were similar from birth to d16 (Fig. 5c, d). On d16, plasma and hepatic AA levels were significantly higher in the A group than in the N group (Fig. 5e).

In addition, 5hmC levels in livers were not significantly different between the N and A groups. These results suggested that in the livers of WT mice, in which AA levels are sufficient, slight increases in AA levels did not induce DNA demethylation through TET activity (Fig. 5f).

We employed MIAMI to analyze the genome-wide DNA methylation status in the livers of offspring in both groups on d16 (Table 4). We found that 560 and 409 genes were hypomethylated and hypermethylated, respectively, in the A group relative to those in the N group (Table 4, Supplementary Table 7a, b). MIAMI revealed that the number of hypomethylated genes was similar in the A and N groups, which confirmed that a slight increase in plasma and liver AA levels in WT mice had a minimal effect on DNA demethylation.

**Table 4:** The number of genes with DNA methylation changes in the liver of A offspring relative to those in N offspring on d16.

	Number of genes		
	Total number	DNA hypomethylation	DNA hypermethylation
**d16**	25,091	560	409

## Discussion

In the present study to elucidate the role of AA in DNA demethylation in the murine liver during the suckling period, we performed three experiments using WT and SMP30/GNL KO mice.

We employed MIAMI to evaluate the DNA methylation status of genes in the liver. In MIAMI, we only evaluated the DNA methylation status on the HpaII/MspI restriction enzyme site CCGG. Therefore, we did not examine whole DNA methylation sites, which is a limitation of the present study. Moreover, the DNA methylation status of each gene was calculated as a relative, not an absolute value. Therefore, we were unable to precisely observe changes in the DNA methylation status of each gene with AA supplementation, which is another limitation.

The AA transporters, sodium-dependent vitamin C transporter (SVCT) 1 and SVCT2, and DHA transporters, glucose transporter (GLUT) 1, GLUT3, and GLUT4, are expressed in the small intestine of both WT and SMP30/GNL KO mice^24^. Therefore, N and KO offspring are both capable of taking in AA and DHA from milk. In the first experiment, even though plasma and milk AA levels in SMP30/GNL KO dams were similar to those in WT dams, plasma and hepatic AA levels were markedly lower in KO offspring than in N offspring, suggesting that AA levels in offspring during the suckling period largely depended on the intrinsic synthesis of AA in the offspring themselves. Furthermore, markedly low AA levels contributed to global DNA hypermethylation in the liver. Therefore, the intrinsic synthesis of AA in offspring during the suckling period appears to be necessary and sufficient for proper DNA demethylation in the liver. Although we found no morphological changes in the livers of the offspring in any of the experiments conducted, KO offspring in later life (d100) exhibited the DNA hypermethylation of genes in the mTOR, Wnt, TGF-β, and MAPK signaling pathways, which are essential for organogenesis and development of the liver. Therefore, AA may exert DNA demethylation effects in many organs, including the liver, because a lack of AA in early life was shown to inhibit normal development of the brain, heart, and lungs^15,25^. Moreover, in a previous study^15^, we reported embryonic lethality and severe organ developmental disorders in SMP30/GNL KO mice without AA supplementation, which may be attributed to aberrant DNA methylation.

Although AA levels in KO offspring were restored by the administration of 1.5 g/L AA water in adulthood, the DNA methylation status of PPARα target genes, including *Fgf21*, was hypermethylated relative to those in N offspring and persisted into adulthood, suggesting that the suckling period may be the ‘critical window’ for the modulation of the DNA methylation status in the liver, which we recently reported^19,22^. These results led to the second experiment to support this hypothesis because high-dose AA administration to KOA dams during the lactation period induced global DNA demethylation in the livers of KOA offspring during the suckling period.

AA induces TET-dependent DNA demethylation *in vitro*^15,25^. The present results supported AA also inducing DNA demethylation in vivo. Hypermethylated genes in KO offspring relative to those in N offspring on d16 and d100 included lipid metabolism-related genes, such as *Fgf21,* suggesting that an AA deficiency in the suckling period results in disorders in hepatic lipid metabolism in the offspring to adulthood. SMP30/GNL KO mice without AA supplementation developed fatty liver and dyslipidemia in adulthood^25,26^. DiTroia et al. recently attempted to elucidate the role of AA in the developing germline, employing Gulo knockout mice, which cannot synthesize their own AA^27,28^. They demonstrated that a maternal AA deficiency during gestation up to embryonic day 13.5 did not affect overall embryonic development, but led to reduced numbers of germ cells, delayed meiosis, and reduced fecundity in adult offspring. They also showed that it led to the aberrant DNA methylation of meiosis regulators, transposable elements, and genes related to lipid and body weight homeostasis in the embryonic germline of the progeny, which was observed in germ cells in TET1 knockout mice^27,29^. These findings suggested that AA is required for proper DNA demethylation in germ cells and that lipid metabolism-related genes are susceptible to AA-dependent DNA demethylation during the gestation period, which is consistent with the present results.

In the first experiment, even though hepatic lipid metabolism-related genes were hypermethylated in KO offspring, no significant differences were observed in gene expression levels or serum lipid parameters between N and KO offspring. Previous studies reported that differences in the DNA methylation status may not reflect differences in steady-state gene expression^30,31^. However, in a transcriptionally active state, even subtle differences in the DNA methylation status may induce marked differences in gene expression^30–32^. Thus, some environmental cues, such as a high-fat diet, may be required to highlight changes in lipid metabolism-related gene expression and the metabolic phenotypes of KO offspring in adulthood. In the third experiment, the administration of 1.5 g/L AA water to WT dams resulted in a slight increase in plasma and hepatic AA levels in their offspring. However, this increase did not markedly induce any additional DNA demethylation in the liver, which suggested that the intrinsic synthesis of AA in the offspring was sufficient for proper DNA demethylation. Since humans are incapable of synthesizing AA, the present results are of significance; a maternal AA deficiency during the lactation period may induce the DNA hypermethylation of genes related to organogenesis and lipid metabolism in the livers of babies, which may result in organ disorders and metabolic diseases in adulthood. A previous study reported that AA prevented an aberrant DNA methylation status in offspring associated with maternal smoking in pregnancy^18^.

In the present study, we did not administer high-dose AA, which was administered to KOA dams, to WT dams. However, since KOA offspring had more hypermethylated genes than N offspring on d16, similar to KO offspring by MIAMI, high-dose AA administered to KOA dams was not sufficient to restore plasma AA levels in KOA offspring. Moreover, because *Fgf21* was not included in the genes that were hypomethylated in KOA offspring relative to those in KO offspring on d16, it is conceivable that plasma AA levels in KOA offspring did not induce the hypomethylation of *Fgf21*.

We identified 163 genes that were presumably hypermethylated in KO offspring and hypomethylated by high-dose AA supplementation (Supplementary Table 4). Based on these results, the few genes hypermethylated in KO offspring were hypomethylated by high-dose AA supplementation, which may have been attributed to the lower plasma AA concentration in KO offspring despite high-dose AA supplementation than in N offspring. However, we do not think that only a small fraction of genes hypermethylated in KO offspring were hypomethylated by high-dose AA supplementation because 172 genes were hypermethylated in KOA offspring relative to those in KO offspring on d16 (Table 3a), whereas up to 2076 genes were hypermethylated in KO offspring relative to those in N offspring (Table 1a). Further studies are needed to clarify whether the administration of markedly higher doses of AA to WT dams induces hypomethylation in the livers of their offspring, which was a limitation of the present study.

In conclusion, the present study provides in vivo evidence to show that AA epigenetically regulates many genes, including those related to organogenesis and lipid metabolism, in the liver during the suckling period. Thus, a maternal AA deficiency in humans, particularly during the lactation period, may exert deleterious effects in babies, which is a crucial issue for lifelong health.

## Materials and methods

### Animals

All animal experiments were approved by the Tokyo Medical and Dental University Committee on Animal Research (#0,170,125 A) and the Animal Care and Use Committee of the Tokyo Metropolitan Institute of Gerontology (#17,074). All methods involving animals were performed in accordance with the relevant guidelines and regulations. All mice were treated in accordance with the Fundamental Guidelines for the Proper Conduct of Animal Experiments and Related Activities in Academic Research Institutions under the jurisdiction of the Ministry of Education, Culture, Sports, Science and Technology of Japan. All efforts were made to minimize suffering and to reduce the number of animals used.

Pregnant female C57BL6 mice, which were primiparous and housed individually, were purchased from CLEA Japan (Tokyo, Japan) on gestational day 13. After delivery, litter size was adjusted to 5–6 pups (all male) per dam to avoid metabolic drifts due to nutrient availability during the lactation period. SMP30/GNL KO mice were generated using a gene-targeting technique as described previously^33^. Female SMP30/GNL KO (SMP30/GNL-/-) mice were mated with male SMP30/GNL KO (SMP30/GNLY/-) mice to produce SMP30/GNL KO (male SMP30/GNLY/- and female SMP30/ GNL-/-) mice under normal AA conditions; mice had free access to water containing 1.5 g/L AA and 100 µmol/L EDTA to avoid the effects of an AA deficiency^20^. The concentration of AA in drinking water was sufficient to maintain normal AA levels in all tissues^20^. EDTA was added to stabilize AA in drinking water, which remained in a stable state for at least 4 days^20^. Water bottles were changed every 3 or 4 days. In addition, all mice were fed an AA-free diet (CL-2, CLEA Japan, Tokyo, Japan) ad libitum. During the lactation period, KOA and KO dams received an i.p. injection of AA in saline (4.0 g/L) and solvent only, respectively. Throughout the experiments, animals were maintained on a 12-h light/dark cycle in a controlled environment.

### Measurement of AA concentrations

The total AA concentration, including AA and dehydroascorbic acid (DHA), the oxidized form of AA, was measured using high-performance liquid chromatography and electrochemical detection in accordance with previously described methods^34^.

### DNA methylation profiling

Mouse liver genomic DNA was extracted using a standard proteinase K method. MIAMI was performed as described previously^19,35^. Briefly, one microgram of genomic DNA from the samples of two groups was digested with the methylation-sensitive restriction enzyme HpaII followed by adaptor ligation and PCR amplification. Amplified DNA from one sample was labeled with Cy3 and the other with Cy5, and they were then cohybridized to gene promoter arrays containing 41,332 probes. The fluorescence signals obtained were normalized using the global normalization method, and signal differences between samples (i.e. Cy3 signal intensity/Cy5 signal intensity), which indicated DNA methylation differences between samples, were assessed. Genomic DNA was also digested with the methylation-insensitive isoschizomer MspI followed by the same procedure to correct for false positives caused by single nucleotide polymorphisms or incomplete digestion. The HpaII/MspI signal difference for each probe was measured as the methylation difference value. Values of < 0.75 and > 1.33 denoted DNA hypomethylation and hypermethylation, respectively. DAVID v6.7 (http://david.abcc.ncifcrf.gov/) was employed for the pathway analysis. Corrected P-values were used to judge candidate genes (*p* < 0.05 was considered to be significant)^22^.

The complete experimental protocol is available at http://epigenome.dept.showa.gunmau.ac.jp/~hatada/miami/image/MIAMI%20Protocol%20V4.pdf.

### Bisulfite-sequencing analysis

A bisulfite-sequencing analysis was performed as follows. To prepare the bisulfite stock solution, 3.8 g of sodium metabisulfite (#31,609–45, Nacalai Tesque, Kyoto, Japan), 1.34 g of ammonium sulfite monohydrate (#014–03,505, Wako Pure Chemical Industries, Osaka, Japan), and 10 mL of 50% ammonium hydrogen sulfite solution (#014–02,905, Wako) were mixed and heated at 80 °C until dissolved. To prepare a working bisulfite solution (8.4 M, pH 5.2–5.3), 12 mL of the bisulfite stock solution was diluted with 2.28 mL of distilled water (dH_2_O). Two micrograms of genomic DNA was denatured in 0.3 N NaOH in a total volume of 24.75 μL at 37 °C for 30 min. The sample was mixed with 275 μL of the working bisulfite solution and incubated at 70 °C for 1 h in the dark. DNA was recovered using a genomic DNA purification kit (#NPK-101, Toyobo Life Science Department, Osaka, Japan) and dissolved in 100 μL of dH_2_O. The DNA sample was mixed with 100 μL of 0.4 N NaOH (freshly prepared) and incubated at 37 °C for 15 min. DNA was recovered by adding 150 μL of 5 M ammonium acetate (pH 7.0), 2 μL of ethacinmate (#312–01,791, Nippon Gene, Tokyo, Japan), and 750 μL of ethanol. The DNA-containing precipitate was dissolved in 20 μL of 10 mM Tris–HCl/1 mM EDTA (TE; pH 7.5) and subjected to PCR amplification. The sequential PCR amplification of *Fgf21* was performed using specific primers as follows: forward: TTTAGTTTTTTTTTTTAGATTTAGGAGTGTAGATT, reverse: TCCTCCTCTCAACCTCCATAAAAC. Reaction profiles were 40 cycles at 96 °C for 15 s, 59 °C for 30 s, and 72 °C for 60 s. Amplified fragments were ligated into pGEM-T easy vectors (#A1360, Promega, Madison, WI, USA), and more than 13 clones were sequenced per reaction. We used a web-based quantification tool for the bisulfite-sequencing analysis of CpG methylation (http://quma.cdb.riken.jp/)^36^.

### 5hmC dot blot analysis

Isolated DNA (450 ng per sample) was denatured in 1 M NaOH at 95 °C for 10 min. Samples were neutralized with 5 M NH_4_OAc on ice. DNA samples were spotted on a Nylon membrane (Hybond-N + , GE-Amersham, UK). The blotted membrane was dried at 80 °C for 30 min, and UV cross-linked at 70,000 µjoules/cm^2^. The membrane was then incubated with Blocking One (Nacalai Tesque, Kyoto, Japan) at room temperature for 1 h. The rabbit polyclonal antibody anti-5hmC (No. 39770; Active Motif, USA, dilution 1:5000) in PVDF Blocking Reagent for Can Get Signal 1 (Toyobo, Osaka, Japan) was added at room temperature for 2 h. The membrane was washed for 10 min 3 × in TBST buffer, and then incubated with HRP-conjugated anti-rabbit IgG (#NA934V, GE-Amersham, UK) secondary antibodies in Can Get Signal 2 (Toyobo, Osaka, Japan) at room temperature for 1 h. The membrane was then washed for 10 min 3 × in TBST and visualized by chemiluminescence with ECL Prime (GE-Amersham, UK).

### Biochemical assays

Serum NEFA, TG, and T-Chol levels were measured using NEFA C-Test Wako (#279–75,401), TG E-Test Wako (#432–40,210), and T-Chol E-Test Wako (#439–17,501) kits (Wako Pure Chemical Industries, Ltd., Osaka, Japan), respectively^19,22^. Blood glucose was measured using a glucometer (Glutest PRO R; Sanwa Kagaku Kenkyusho Co., Ltd., Aichi, Japan)^22^. As milk samples, we used the gastric contents of offspring, which mainly consisted of milk derived from dams, on d16^22^.

### Statistical analysis

Data are expressed as the mean ± standard error of the mean (SEM). Data were compared using an unpaired Student’s *t*-test. Comparisons of body weight differences were performed using a two-way analysis of variance (ANOVA) with Tukey’s multiple comparison test. A *p*-value of < 0.05 was considered to be significant. Statistical analyses were performed using Prism 6 (GraphPad Software, Inc., La Jolla, CA, USA).

## Supplementary information

Supplementary Information.

## Data Availability

Data that support the results of the present study are available from the corresponding author upon reasonable request.
