# Author Correction: Ascorbic acid during the suckling period is required for proper DNA demethylation in the liver

**DOI:** 10.1038/s41598-021-91691-5

**Published:** 2021-06-03

**Authors:** Kenichi Kawahori, Yoshitaka Kondo, Xunmei Yuan, Yuki Kawasaki, Nozomi Hanzawa, Kazutaka Tsujimoto, Fumiko Wada, Takashi Kohda, Akihito Ishigami, Tetsuya Yamada, Yoshihiro Ogawa, Koshi Hashimoto

**Affiliations:** 1 Department of Molecular Endocrinology and Metabolism, Graduate School of Medical and Dental Sciences, Graduate School of Medical and Dental Sciences, Tokyo Medical and Dental University, Tokyo, 113‑8510 Japan; 2 Molecular Regulation of Aging, Tokyo Metropolitan Institute of Gerontology, Tokyo, 173‑0015 Japan; 3 Biomedical Gerontology Laboratory, Faculty of Human Sciences, Waseda University, Tokorozawa, 359‑1192 Japan; 4 Department of Molecular and Cellular Metabolism, Graduate School of Medical and Dental Sciences, Tokyo Medical and Dental University, Tokyo, 113‑8510 Japan; 5 Department of Epigenetics, Medical Research Institute, Tokyo Medical and Dental University, Bunkyo‑ku, Tokyo, 113‑8510 Japan; 6 Laboratory of Embryology and Genomics, Department of Biotechnology, Faculty of Life and Environmental Sciences, University of Yamanashi, Kofu, Yamanashi 400‑8510 Japan; 7 Department of Medicine and Bioregulatory Science, Graduate School of Medical Sciences, Kyushu University, 3‑1‑1 Maidashi, Higashi‑ku, Fukuoka, 812‑8582 Japan; 8 Department of Preemptive Medicine and Metabolism, Graduate School of Medical and Dental Sciences, Tokyo Medical and Dental University, 1‑5‑45 Yushima, Bunkyo‑ku, Tokyo, 113‑8510 Japan; 9 Department of Diabetes, Endocrinology and Hematology, Dokkyo Medical University Saitama Medical Center, 2‑1‑50 Minami‑Koshigaya, Koshigaya, Saitama 343‑8555 Japan

Correction to: *Scientific Reports* 10.1038/s41598-020-77962-7, published online 04 December 2020

This Article contains an error in Figure 3 where the baseline of the graph is shifted upward in panel (b).

The correct Figure 3 appears below as Figure 1.

**Figure 1:**
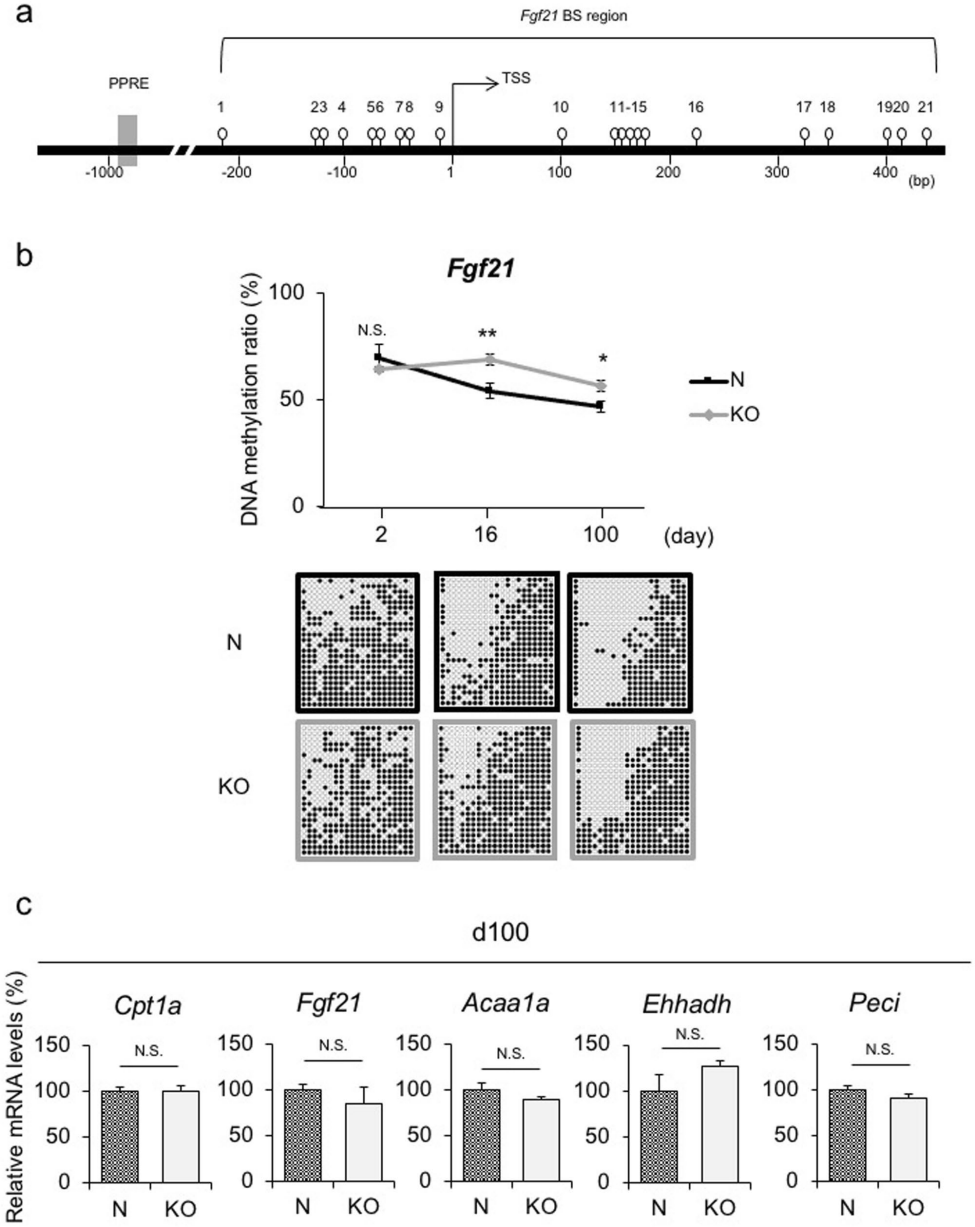
DNA methylation status and gene expression levels of PPARα target genes in livers of KO and N offspring. **(a)** Schematic representation of the promoter region of *Fgf21*. Open circles and gray boxes indicate CpG sites and PPAR response elements (PPREs), respectively. The bisulfite-sequencing (BS) analysis region encompassing the transcription start site (TSS) is indicated. (**b**) *Fgf21* DNA methylation status in the livers of N and KO offspring via the BS analysis (n = 4 per group, statistical analysis using a one-way ANOVA with Tukey’s multiple comparison test). Representative BS data are depicted below the graph. Data are expressed as the mean ± SEM. *p < 0.05; ** *p* < 0.01; N.S., not significant vs. KO offspring. (**c**) Hepatic *Cpt1a*, *Fgf21*, *Acaa1a*, *Ehhadh*, and *Peci* mRNA expression levels on d16, which were DNA hypermethylated on both d16 and d100. (n = 6 per group). Statistical analysis using an unpaired Student’s *t*-test unless otherwise indicated. Data are expressed as the mean ± SEM. N.S., not significant vs. KO offspring.

