# Supplementary material for: Ascorbic acid during the suckling period is required for proper DNA demethylation in the liver
Source: Sci Rep. 2020 Dec 4;10:21228. doi: 10.1038/s41598-020-77962-7 (PMC7718234; doi:10.1038/s41598-020-77962-7)
Supplement: Supplementary file 1 — Supplementary Information. [file 41598_2020_77962_MOESM1_ESM.pdf]

Supplementary information

Ascorbic acid during the suckling period is required for proper DNA demethylation in the liver

Kenichi Kawahori, Yoshitaka Kondo, Xunmei Yuan, Yuki Kawasaki, Nozomi Hanzawa, Kazutaka Tsujimoto, Fumiko Wada, Takashi Kohda, Akihito Ishigami, Tetsuya Yamada, Yoshihiro Ogawa and Koshi Hashimoto

Address all correspondence to:
Koshi Hashimoto, M.D., Ph.D.
Department of Diabetes, Endocrinology and Hematology,
Dokkyo Medical University Saitama Medical Center,
2-1-50 Minami-Koshigaya, Koshigaya, Saitama 343-8555, Japan
Tel/Fax: +81-48-965-1111/8253. E-mail: k-hashi@dokkyomed.ac.jp

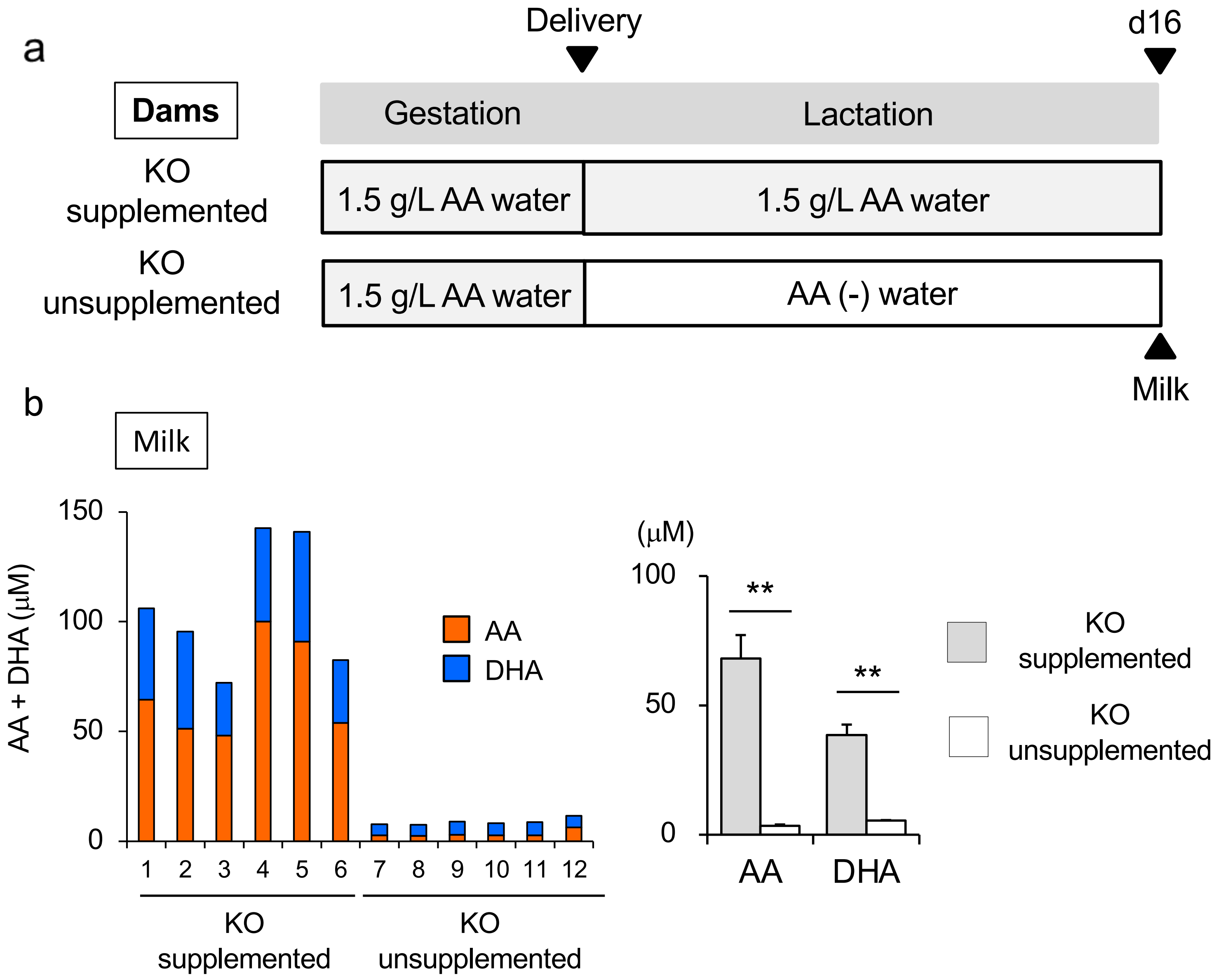

Supplementary Figure 1
Administration of 1.5 g/L AA water to SMP30/GNL KO (KO) dams during the lactation period and AA levels in milk.
(a) Protocol of the preliminary experiment. (b) AA levels in the milk of each dam (left panel). Statistical analysis of AA and DHA levels in the milk of KO supplemented (n=6) and unsupplemented (n=6) dams (right panel).
Statistical analysis using an unpaired Student's *t*-test.
Data are expressed as the mean ± SEM. $^{**}P < 0.01$ vs. KO supplemented dams.

a

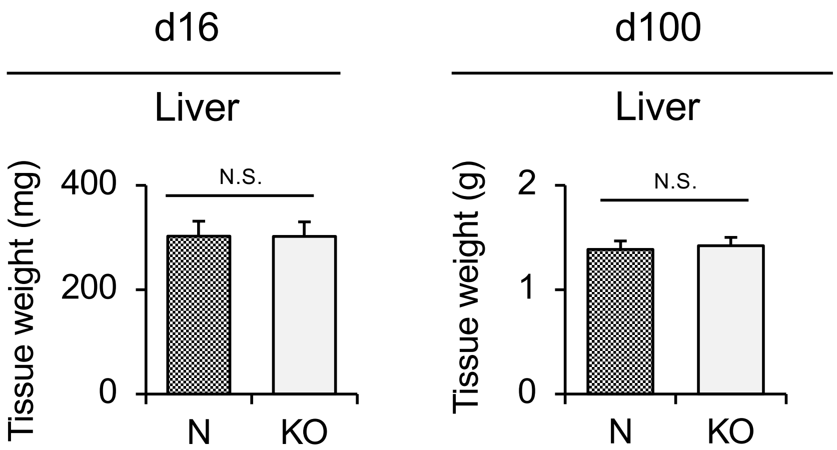

b

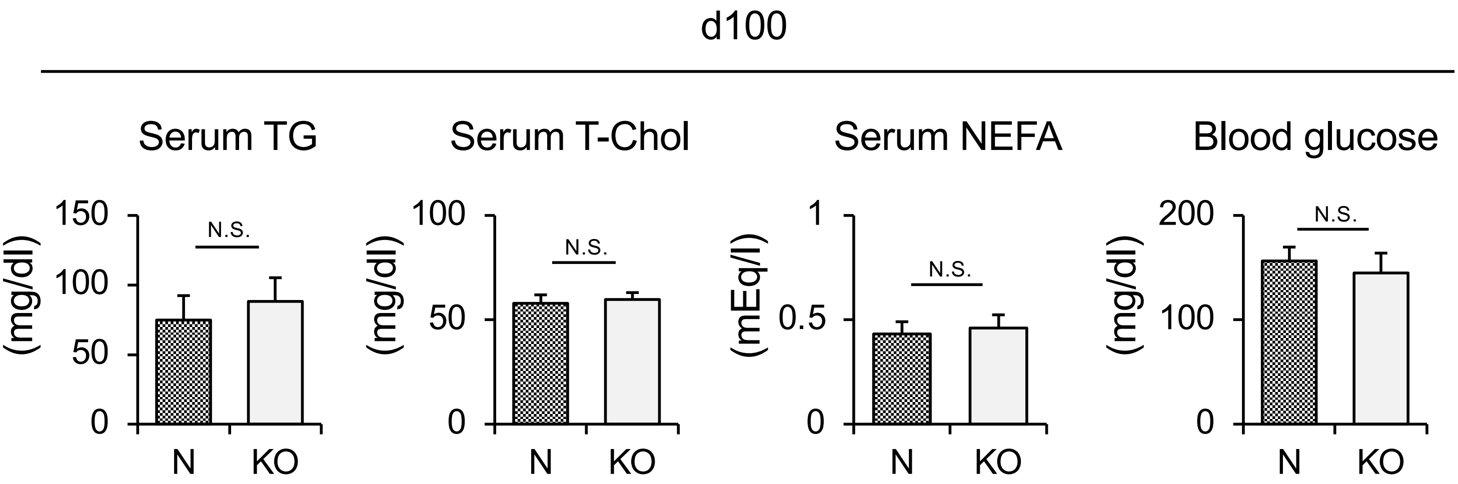

Supplementary Figure 2
(a) Liver weights in KO and N offspring on d16 (left panel) and d100 (right panel) (n=6–8 per group).
(b) Serum TG, T-Chol, NEFA, and blood glucose concentrations on d100 (n=6–8 per group). Statistical analysis using an unpaired Student's *t*-test.
Data are expressed as the mean ± SEM. N.S., not significant vs. N offspring.

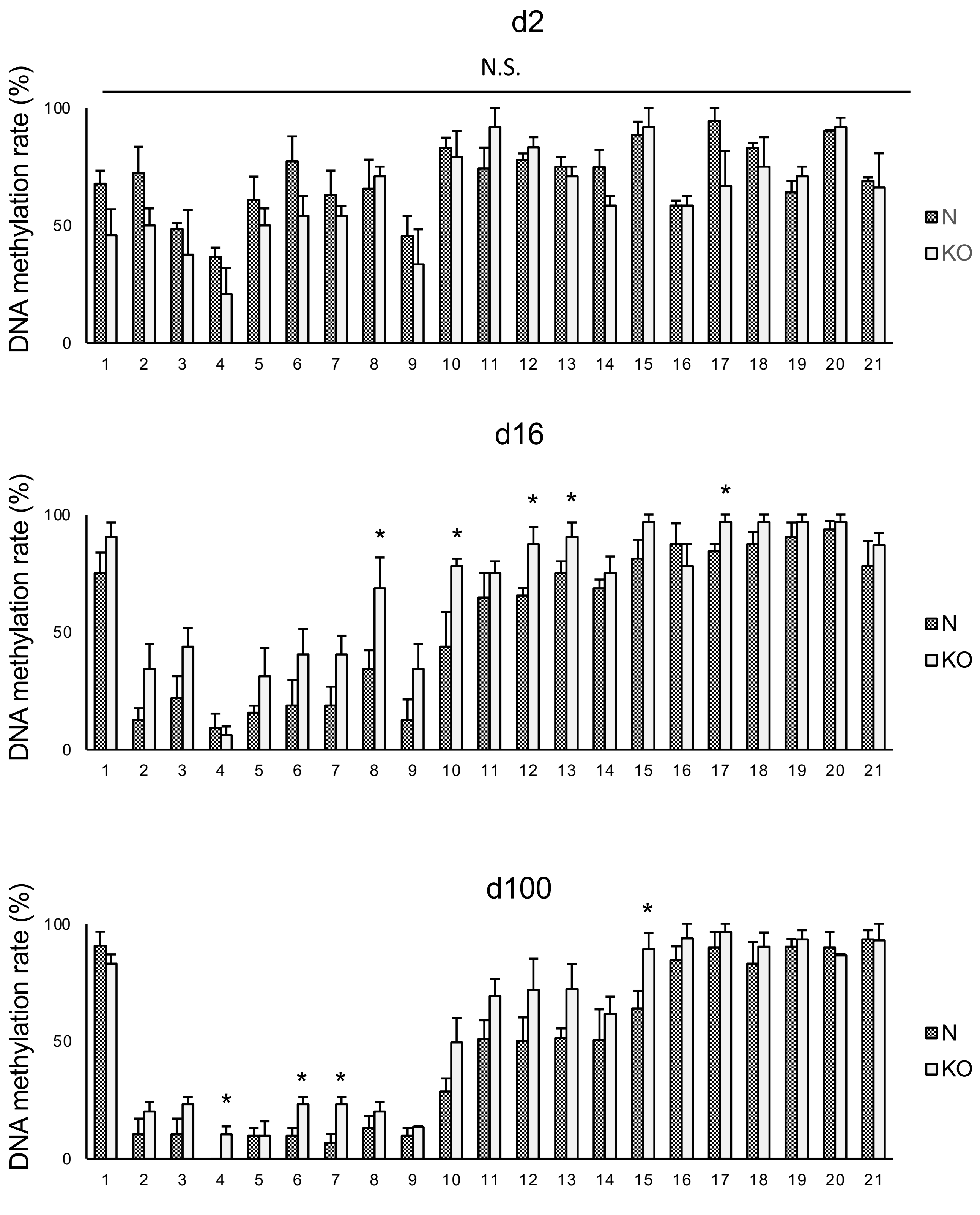

Supplementary Figure 3

Statistical analysis of DNA methylation rates at each CpG site of *Fgf21*.

N (n=4), KO (n=3–4). Statistical analysis using an unpaired Student's *t*-test.

Data are expressed as the mean ± SEM. **P* < 0.05; N.S., not significant vs. N offspring.

Supplementary Table 1a: N vs KO 361 hypomethylation genes on d16

Probe Name	Gene Name	Methylation difference value
A_68_P08214884	Irak3-Tmbim4	0.125921
A_68_P04499666	Camkk2	0.279317
A_68_P09233269	Acadvl-Dlgh4	0.312319
A_68_P02014894	Urm1	0.344014
A_68_P08357114	Trhde	0.34902
A_68_P01716664	Gja9	0.391103
A_68_P02014879	Urm1	0.395382
A_68_P12145566	C1ql4-2810451A06Rik	0.396091
A_68_P05957708	Nkx6-2	0.399
A_68_P05872318	Scnn1a	0.413974
A_68_P09560702	Tbx21	0.424432
A_68_P09449784	Chad	0.426009
A_68_P04844116	Cxcl4	0.461124
A_68_P06597280	Gramd1a	0.477726
A_68_P02023970	1110061N23Rik	0.481116
A_68_P06221701	Bag3	0.481848
A_68_P00632313	Mpzl1	0.485613
A_68_P10712044	Ubqln1	0.496671
A_68_P10116466	Jag2	0.49781
A_68_P13434054	B3galt4	0.500667
A_68_P13174876	Dom3z	0.500697
A_68_P14209556	Rin1	0.506115
A_68_P08991491	1110036O03Rik	0.508581
A_68_P14181866	2700081O15Rik	0.513096
A_68_P13305263	Asah3-Clpp	0.514857
A_68_P13384331	Tcfeb	0.51738
A_68_P13214276	Gpsm3	0.518699
A_68_P12120920	Aqp5	0.523591
A_68_P01560829	2810408M09Rik-Slc2a10	0.524824
A_68_P12864479	Popdc2	0.539121
A_68_P09437569	Zrsr1	0.540649
A_68_P06352181	BC053749-Hspb6	0.541929
A_68_P06117321	Rshl1	0.542194

A_68_P02203170	Elf5	0.546067
A_68_P09528972	Zfp454	0.546671
A_68_P13174873	Dom3z	0.548361
A_68_P09473033	Slc6a4	0.551388
A_68_P01390546	Tcfap2c	0.554509
A_68_P14274310	Xpnpep1	0.555075
A_68_P14305323	Gng3	0.560524
A_68_P00750173	Pde6d	0.568877
A_68_P14495356	I830134H01Rik-Cep55	0.572836
A_68_P07820591	Pcbp4	0.572952
A_68_P09547539	BC017647	0.575527
A_68_P03534967	Zfp593	0.581867
A_68_P07329396	Gm587	0.583631
A_68_P02602374	Gm128	0.584557
A_68_P07651775	Cspg5	0.584654
A_68_P06887581	Hsf4	0.587977
A_68_P09110706	Vamp2	0.588126
A_68_P04453213	Gpr81	0.588632
A_68_P01674549	2610528K11Rik	0.588811
A_68_P08214273	Dapk3	0.592258
A_68_P09153352	Rnf185	0.595229
A_68_P01674542	2610528K11Rik	0.597532
A_68_P12098583	Prph1	0.598549
A_68_P13920652	Npc1	0.598715
A_68_P13266254	Thbs2	0.602386
A_68_P13927242	Pcdhac2	0.602414
A_68_P01316736	Sh2d3c	0.604611
A_68_P13965496	Pstpip2	0.605318
A_68_P01519278	1700037H04Rik	0.605721
A_68_P05872300	Scnn1a	0.606359
A_68_P09547535	BC017647	0.606866
A_68_P07240689	Jund1	0.608162
A_68_P07864839	Gup1	0.608188
A_68_P01851539	Fkhl18	0.613074
A_68_P08829070	Galnt4	0.613235

A_68_P11632181	Jph4	0.616921
A_68_P09548393	Aoc3	0.622859
A_68_P03733795	Tcfap2e	0.623707
A_68_P14628736	6430550H21Rik	0.62502
A_68_P12465449	Kdelr3	0.62509
A_68_P12047179	Sh3bp1	0.625272
A_68_P00789035	Ankrd39	0.625876
A_68_P05240711	Rarres2	0.62619
A_68_P09099286	A830053O21Rik	0.627881
A_68_P00364377	Zfp451	0.627981
A_68_P08564779	Rps15	0.628822
A_68_P11217197	Ltb4r2	0.629809
A_68_P02681348	BC107364	0.632958
A_68_P00906158	Cxcr7	0.63305
A_68_P12543635	Mcm4-Prkdc	0.634384
A_68_P02898238	Cyr61	0.634977
A_68_P08002166	1700104A03Rik	0.635226
A_68_P04137921	Alox5ap	0.635499
A_68_P03957585	Necap2	0.636179
A_68_P04345682	2410025L10Rik	0.63642
A_68_P05030572	Cd27	0.639387
A_68_P04699015	Zp3	0.640996
A_68_P02267970	Chn1	0.642925
A_68_P09017457	2310040C09Rik	0.644335
A_68_P03751620	Zc3h12a	0.647902
A_68_P07374455	Rpl13	0.648297
A_68_P07140842	Pik3r2	0.649261
A_68_P05138684	Cand2	0.649927
A_68_P14545948	Ubqln2	0.650703
A_68_P08435891	Edg6	0.650775
A_68_P14235870	Scyl1	0.650869
A_68_P03743542	Zswim5	0.651373
A_68_P01851550	Fkhl18	0.652145
A_68_P06938838	Egln1	0.652247
A_68_P06053429	Tssc4	0.652901

A_68_P01239005	Cd40	0.653058
A_68_P09240367	Tbkbp1	0.653762
A_68_P14229760	Npas4	0.654225
A_68_P04112279	Gpr30	0.65575
A_68_P13355809	Grm4	0.656133
A_68_P09433957	0610009B22Rik	0.656763
A_68_P11912864	Krt8	0.656893
A_68_P12773755	EG328644	0.656902
A_68_P09042391	P2rx1	0.657686
A_68_P06596029	Ppp5c	0.657997
A_68_P09613199	Coro6	0.660504
A_68_P11423240	E130112L23Rik	0.661076
A_68_P05416737	Cxcl12	0.661122
A_68_P06900371	Ogfod1	0.662146
A_68_P06409764	Atp2a1	0.663605
A_68_P04092027	Dnajb5	0.664341
A_68_P01102474	Sox13	0.6644
A_68_P14311105	Kcnip2	0.666394
A_68_P13594483	Csnk1g3	0.667548
A_68_P00776589	Zfp142-Bcs1l	0.667806
A_68_P13056021	Tnf	0.667863
A_68_P00275693	Gpbar1	0.670061
A_68_P08025256	Pdzd3	0.670416
A_68_P13484729	Tbc1d24	0.671049
A_68_P04999281	5730596B20Rik	0.67269
A_68_P10747285	1110007C09Rik	0.672715
A_68_P12151832	Wnt10b	0.672795
A_68_P10640236	Nid1	0.673032
A_68_P04868247	Coro1c	0.673163
A_68_P09978294	Papln	0.673603
A_68_P13385328	4930546H06Rik	0.673863
A_68_P14704391	Cited1	0.674611
A_68_P05933947	Mical2	0.674843
A_68_P11875450	A_68_P11875450	0.677288
A_68_P01028979	Ptprv-Ptpn7	0.677652

A_68_P00740579	Fzd5	0.677704
A_68_P00273226	EG240916	0.677783
A_68_P09652627	BC029169	0.677939
A_68_P09906321	A830093I24Rik	0.678157
A_68_P06652023	Mrvi1	0.679824
A_68_P01873041	Eng	0.680099
A_68_P12831564	Ets2	0.680777
A_68_P09596001	Patz1	0.681082
A_68_P04617651	Ncor2	0.681624
A_68_P08675960	Palm	0.683342
A_68_P05438068	Kcna1	0.684301
A_68_P05734641	Gata2	0.68551
A_68_P10908480	Dcdc2a	0.685771
A_68_P09449773	Chad	0.685848
A_68_P08669395	Moxd1	0.686129
A_68_P04460565	Sh2b3	0.686167
A_68_P06930178	B3gnt3	0.68797
A_68_P08763291	Hmg20b	0.688355
A_68_P06578180	D930014E17Rik	0.689157
A_68_P03709353	Plcl4	0.690147
A_68_P14292422	Hps6	0.690356
A_68_P08782489	Rkhd1	0.691004
A_68_P04148014	Polr2b	0.691478
A_68_P12241657	Plec1	0.691555
A_68_P06660845	Arntl	0.692637
A_68_P04460565	Sh2b3	0.692791
A_68_P02631941	Nexn	0.693323
A_68_P10014745	Pnma1	0.693359
A_68_P09572006	4933439F11Rik	0.69461
A_68_P13119784	Plg	0.694679
A_68_P12092853	Ppp1r1a	0.695136
A_68_P04453398	Ints1	0.695922
A_68_P15196933	Tex13	0.696127
A_68_P13166506	Ddah2	0.696202
A_68_P03487385	Clcnka	0.696207

A_68_P09166146	Tmc8	0.696329
A_68_P11494144	Nudt18	0.696618
A_68_P05548217	Chd4	0.696779
A_68_P07855774	Hmgn3	0.69731
A_68_P10975508	Gmnn	0.697722
A_68_P05303155	Plxna1	0.69801
A_68_P01110961	Nek2	0.698011
A_68_P03011094	Tmem79	0.698861
A_68_P02220259	9130404D14Rik	0.699233
A_68_P10747279	1110007C09Rik	0.699403
A_68_P03496087	Rcc1	0.700272
A_68_P10018570	Ap4s1	0.700294
A_68_P08708090	Aire	0.700375
A_68_P08376634	Lrrc3	0.700464
A_68_P04028116	Cldn19	0.700611
A_68_P04422939	Ssh1-Dao1	0.700904
A_68_P00443803	Ptma	0.701754
A_68_P10508113	Tbc1d7	0.701874
A_68_P04934457	Ttc28	0.702231
A_68_P03602288	Cdca8	0.702874
A_68_P05896796	Mogat2	0.702971
A_68_P10714293	Wdr37	0.703125
A_68_P12215401	Sstr3	0.703892
A_68_P03361653	Clta	0.703919
A_68_P04371627	Acacb	0.704858
A_68_P10096903	Bdkrb1	0.705774
A_68_P04617651	Ncor2	0.706079
A_68_P04148014	Polr2b	0.706351
A_68_P07903837	Rgl3	0.706554
A_68_P04934457	Ttc28	0.708233
A_68_P04868247	Coro1c	0.708323
A_68_P12916411	Aifm3	0.708763
A_68_P13684816	Pcdha12	0.709138
A_68_P10330008	Sostdc1	0.709319
A_68_P04028278	2410166I05Rik	0.709542

A_68_P06111935	5830482F20Rik	0.710844
A_68_P13166533	Ddah2	0.711201
A_68_P04893781	Snx8	0.711642
A_68_P06522471	Rhog	0.711889
A_68_P10103614	Tnfaip2	0.712084
A_68_P06614033	B3gnt8	0.712098
A_68_P08513877	Pcnt-2610028H24Rik	0.712241
A_68_P12363258	Gdnf	0.712776
A_68_P01977788	Mybl2	0.713067
A_68_P09593299	OTTMUSG00000000934	0.713303
A_68_P09525413	Hes7	0.71332
A_68_P04321318	Sparcl1	0.713426
A_68_P11375105	Ap1g2	0.713831
A_68_P12689036	St6gal1	0.715021
A_68_P05934099	Eef2k	0.715064
A_68_P04519541	Gfi1	0.715563
A_68_P09214455	Kctd11	0.715811
A_68_P13439828	Ptprs	0.716297
A_68_P12784817	St3gal6	0.716503
A_68_P11718247	Tspan14	0.717309
A_68_P04877727	Bmp3	0.717431
A_68_P03792648	Yrdc	0.717437
A_68_P02884425	D3Ertd300e	0.717454
A_68_P05234904	Vps24	0.717545
A_68_P06124278	Myo7a	0.717896
A_68_P01296051	Slc34a3	0.717958
A_68_P06256619	Lgals7	0.718671
A_68_P12215542	Parvb	0.719236
A_68_P08513992	Cdk4	0.72038
A_68_P07957476	Naalad2	0.720388
A_68_P06584744	Leng9	0.720687
A_68_P09491191	D130058I21Rik	0.720861
A_68_P04488676	Slc26a1	0.721268
A_68_P01253610	Hoxd1	0.721289
A_68_P14209573	Rin1	0.72165

A_68_P09425733	2610019A05Rik	0.722488
A_68_P02099291	Mastl-Yme1l1	0.72253
A_68_P09292536	Gria1	0.723134
A_68_P14280936	Plcb3	0.723248
A_68_P08636022	Myl6	0.723833
A_68_P01680617	Gyltl1b-Pex16	0.724056
A_68_P04422939	Ssh1-Dao1	0.72428
A_68_P04047546	Icmt	0.724809
A_68_P01573803	Srxn1	0.725053
A_68_P11165637	Fut11	0.72549
A_68_P05636961	Fkbp14-Plekha8	0.725849
A_68_P13201109	Jmjd2b	0.72621
A_68_P13355818	Grm4	0.727152
A_68_P12341775	Plec1	0.727287
A_68_P10857126	Cdc14b	0.727792
A_68_P05934093	Eef2k	0.728659
A_68_P02083343	2310003F16Rik	0.728743
A_68_P00496856	Ankrd23	0.728936
A_68_P01923886	Mc3r	0.729017
A_68_P03751620	Zc3h12a	0.729428
A_68_P07246627	Rfx1	0.729883
A_68_P09356521	Enpp7	0.729887
A_68_P03496087	Rcc1	0.729996
A_68_P06928238	Spire2	0.73006
A_68_P05908753	Cd81	0.730184
A_68_P00363449	Teddm1	0.730516
A_68_P07887111	Dock6	0.730736
A_68_P07287900	Gpr56	0.730926
A_68_P03255820	Extl1	0.73101
A_68_P07950323	Sh3px3	0.731444
A_68_P05389639	Ldhb	0.731474
A_68_P11613508	Kctd12	0.73185
A_68_P10446717	Irx4	0.73249
A_68_P09161409	Scrn2	0.732636
A_68_P13241937	Nfkbie	0.732862

A_68_P09104652	Aatk	0.733589
A_68_P14131501	Lox	0.734047
A_68_P03973054	Dvl1	0.734255
A_68_P06599172	Grin2d-Kdelr1	0.734478
A_68_P13127089	Csnk2b-Bat4	0.735142
A_68_P04698270	Fndc4	0.735177
A_68_P03867484	Clstn1	0.735425
A_68_P04218037	Tbx3	0.735503
A_68_P04812922	Ugdh	0.735848
A_68_P04337962	Rnf4	0.736145
A_68_P07180083	Rnf166	0.73641
A_68_P07321844	Ank1	0.736415
A_68_P08527799	Psap	0.73649
A_68_P05548225	Chd4	0.736814
A_68_P07600936	Gnai2	0.73698
A_68_P10363078	Six6	0.737019
A_68_P11507604	Pcdh17	0.73739
A_68_P03792648	Yrdc	0.737405
A_68_P14154937	Ankrd2	0.737757
A_68_P10602136	Btn2a2	0.737769
A_68_P06444721	Snrpa	0.737845
A_68_P03606907	Cd72	0.737901
A_68_P03457489	Ppcs-Zmynd12	0.738639
A_68_P14814534	Gpm6b	0.738689
A_68_P07185461	Wfdc1	0.738804
A_68_P08275638	Rfx4	0.739075
A_68_P00160667	Dpt	0.739301
A_68_P00606676	Gpa33	0.739334
A_68_P05691152	Chn2	0.739362
A_68_P06833358	Gpr56	0.739499
A_68_P09941671	Sstr1	0.74007
A_68_P04070267	Dhcr24	0.740619
A_68_P06666492	C030039L03Rik	0.740669
A_68_P04751477	1110006O24Rik	0.741191
A_68_P05331102	Gng11	0.741293

A_68_P05957743	Nkx6-2	0.741499
A_68_P04240365	Crmp1	0.741724
A_68_P10379467	Slc25a21	0.741849
A_68_P12199134	4930572J05Rik	0.742044
A_68_P13948787	Arhgap12	0.742451
A_68_P06012484	2610207I05Rik-4930583K01Rik	0.742491
A_68_P13644965	Tubb6	0.743278
A_68_P10198482	Akt1	0.743675
A_68_P13205225	Trim40-Trim31	0.743786
A_68_P08513384	Slc35d3	0.743852
A_68_P13450793	Zbtb22	0.744209
A_68_P12526550	A_68_P12526550	0.744331
A_68_P01015609	Cdc73	0.744382
A_68_P03439401	Dmbx1	0.744462
A_68_P13140353	Rhoq	0.745189
A_68_P00854962	Phlda3	0.745195
A_68_P01075518	Wnt6	0.745267
A_68_P14889744	Mid1ip1	0.745313
A_68_P10337840	Npas3	0.745441
A_68_P00072270	Ptpn14	0.74565
A_68_P07291347	Calb2	0.746013
A_68_P03797739	Nrd1	0.746187
A_68_P09651106	2400006H24Rik	0.746323
A_68_P12007011	Exosc4	0.746508
A_68_P06935487	Arhgef7	0.746566
A_68_P07422403	Lyl1	0.746612
A_68_P06064392	Foxa3	0.74673
A_68_P10491539	Hist1h2ac-Hist1h2bc	0.747252
A_68_P06554044	Cd151	0.74777
A_68_P06026491	St8sia2	0.748199
A_68_P14751401	Usp11	0.7483
A_68_P00186067	A830006F12Rik	0.748319
A_68_P07309112	Dnase2a	0.748356
A_68_P13748923	Zadh2	0.748589
A_68_P02667260	Pcdh10	0.748733

A_68_P00722329	Des	0.748981
A_68_P12191245	Dgat1	0.748994
A_68_P12361670	Zfp385	0.749539
A_68_P03032928	Hcn3	0.749552
A_68_P08112408	Slc44a2	0.749614
A_68_P07512596	Apeh	0.749822
A_68_P01060395	Jph1	0.749868
A_68_P02254675	Trp53bp1	0.750289
A_68_P00957880	Eif5b	0.750973
A_68_P14299016	2900009I07Rik	0.751093
A_68_P02560176	Efna1	0.751514
A_68_P09134740	Col1a1	0.7517
A_68_P10174581	Gsc	0.751757

Supplementary Table 1b: N vs KO 2076 hypermethylation genes on d16

Probe Name	Gene Name	Methylation difference value
A_68_P07868328	BC021608	149.5089
A_68_P12887690	Prodh	56.51778
A_68_P00618538	Etnk2	41.47804
A_68_P12362697	Gpt1	37.37363
A_68_P00900301	Pcmtd1	35.40694
A_68_P12329747	Arid2	35.40673
A_68_P14169988	Abcc2	25.8389
A_68_P05887814	Echs1	21.04673
A_68_P08321362	Apon	20.82213
A_68_P05307915	Adipor2	19.10023
A_68_P02464513	Ccrn4l	17.55715
A_68_P05678107	Inmt	16.7483
A_68_P08974352	ENSMUST00000100735.1:-5885	16.20611
A_68_P09537504	Afmid	15.9442
A_68_P08563152	Ela2	15.63068
A_68_P05519173	Mkln1	15.6077
A_68_P03900413	Klhdc7a	13.73074
A_68_P13594309	Lipg	12.32939
A_68_P13318294	Ntn2l	12.10038
A_68_P07593875	Slc38a3	11.27771
A_68_P06842129	Mlf1ip	10.56861
A_68_P05908415	2700050L05Rik	10.22173
A_68_P09723695	BC018371	10.10064
A_68_P05943561	Slc27a5	9.543653
A_68_P08066253	Slc38a3	9.35168
A_68_P07593867	Slc38a3	9.316429
A_68_P08691684	Atg5	9.177752
A_68_P08005788	Odf3l1	8.806411
A_68_P11542106	Itih1	8.359333
A_68_P04599720	Cxcl11	8.030745
A_68_P03805415	Ambp	7.904292
A_68_P04899477	Brap	7.728543
A_68_P07946875	Mst1	7.503386

A_68_P03581859	St3gal3	7.389434
A_68_P09529779	Smarcd2-Tcam1	7.286503
A_68_P09726405	Ace	7.273568
A_68_P03733137	Rnf186	7.273549
A_68_P11040896	Peci	7.174847
A_68_P03430349	2010305A19Rik	6.971917
A_68_P12362731	Mfsd3	6.899038
A_68_P06878871	Mtus1	6.818042
A_68_P12673756	Nr1i2	6.745857
A_68_P13360070	A_68_P13360070	6.741152
A_68_P06895632	Ces1	6.672445
A_68_P04281361	Hpd	6.622698
A_68_P06842128	Mlf1ip	6.576161
A_68_P13374816	Zbtb12	6.548569
A_68_P04983814	Abcb9	6.515812
A_68_P04376201	Micall2	6.515287
A_68_P07599181	6230410P16Rik	6.39384
A_68_P04281361	Hpd	6.213813
A_68_P00901045	4632411B12Rik	6.157244
A_68_P08439511	Ftcd	6.083482
A_68_P09529795	Smarcd2	5.906523
A_68_P01293034	4833422F24Rik	5.881187
A_68_P13499424	Slc29a1	5.799522
A_68_P05979604	Lsr	5.644396
A_68_P06410148	Zfp710	5.633832
A_68_P01163299	0610012H03Rik	5.543465
A_68_P13319154	9530058B02Rik	5.511274
A_68_P13318311	Ntn2l	5.48332
A_68_P14259005	AI450540	5.479673
A_68_P01219086	Pck1	5.389813
A_68_P09575212	Rsad1	5.372731
A_68_P05975621	Thrsp	5.360202
A_68_P12670694	Lpp	5.226043
A_68_P03900427	Klhdc7a	5.217989
A_68_P09152032	Rnf135	5.197919

A_68_P09016285	Myo18a	5.172534
A_68_P07501017	Apoa5	5.107357
A_68_P14652218	ENSMUST00000037596.5:-3006	5.10705
A_68_P13695623	Ablim3	5.100664
A_68_P04599730	Cxcl11	4.951345
A_68_P06293260	Mir16	4.879288
A_68_P11718847	2200001I15Rik	4.835499
A_68_P08595238	2310011J03Rik	4.822747
A_68_P10766271	Slc12a7	4.79233
A_68_P08992028	Pnpo	4.763937
A_68_P03900439	Klhdc7a	4.763754
A_68_P11762025	Rnase13	4.641695
A_68_P04543843	Bri3	4.621074
A_68_P05640859	Fabp1	4.613207
A_68_P01293529	A530013C23Rik	4.598874
A_68_P07622451	Apoc3	4.578159
A_68_P12051107	Naprt1	4.569646
A_68_P06048161	Pak4	4.564847
A_68_P08364613	Gstt3	4.540542
A_68_P05811578	Akr1d1	4.526943
A_68_P11089043	Hmgcr	4.516345
A_68_P14367604	Chka	4.444416
A_68_P09582270	2010305C02Rik	4.435232
A_68_P02145945	Clp1	4.322997
A_68_P03375546	Slc25a34	4.182237
A_68_P04546813	Slc2a9	4.170806
A_68_P03059976	Ptgfr	4.147474
A_68_P02110645	Ccbl1	4.135499
A_68_P04081654	Dio1	4.128077
A_68_P07444192	Indol1	4.120093
A_68_P13396158	Dusp1	4.086357
A_68_P12208598	2810451A06Rik	4.079221
A_68_P06312658	Tmem147	4.075241
A_68_P01920530	Uap1l1	4.0708
A_68_P06459191	Saa4	4.066211

A_68_P07697198	Acaa1a	4.063827
A_68_P11936126	Josd1-Gtpbp1	4.005444
A_68_P11406546	Ang1	3.963736
A_68_P14030415	Etf1	3.946627
A_68_P05885085	Gfpt1	3.93886
A_68_P03625246	Zswim5	3.927893
A_68_P13070859	S3-12	3.850016
A_68_P13879820	Slc23a1	3.830024
A_68_P09628447	Arfl4	3.822164
A_68_P08173092	Cyp8b1	3.787066
A_68_P05932565	Polr2i	3.785788
A_68_P06096064	Adam12	3.772641
A_68_P07894607	E330026B02Rik	3.770963
A_68_P08433161	Gstt3	3.76907
A_68_P00846988	Wdr42a	3.759741
A_68_P04755481	Ociad2	3.757315
A_68_P12046833	ENSMUST00000083630.1:-3976	3.727492
A_68_P14275989	Tbc1d12	3.71587
A_68_P05596978	Rnf103	3.715107
A_68_P09620642	Dcxr	3.673635
A_68_P10554343	Pik3r1	3.667529
A_68_P10766266	Slc12a7	3.664243
A_68_P04101457	Gsh2	3.648931
A_68_P13322670	Prr3	3.621062
A_68_P14019783	Zfp532	3.619501
A_68_P10027436	Rgs6	3.611875
A_68_P09620647	Dcxr	3.609131
A_68_P10035212	Zfyve1	3.608449
A_68_P11558065	Dhrs4	3.572378
A_68_P12411237	Derl1	3.562609
A_68_P09289118	Aldh3a2	3.556626
A_68_P06214832	Aqp8	3.552239
A_68_P02421072	Dnajb4-Fubp1	3.547501
A_68_P06631626	Wtip	3.499663
A_68_P01249319	Sephs1	3.480791

A_68_P13107839	Abcg8	3.46978
A_68_P08416674	Pbld	3.462775
A_68_P03439290	1110049F12Rik	3.449798
A_68_P01717991	Btbd14a	3.436893
A_68_P00880269	Zfand2b	3.410346
A_68_P07920795	Apoa4	3.408297
A_68_P00121769	Rpe	3.390084
A_68_P07210491	F10	3.378174
A_68_P07897281	Rdx	3.362075
A_68_P07889364	Tex12	3.355204
A_68_P06223065	Sephs2	3.352343
A_68_P13586042	C3	3.338934
A_68_P01875370	Kcnb1	3.32409
A_68_P06926631	A230052G05Rik	3.317019
A_68_P08066246	Slc38a3	3.308154
A_68_P03526224	Cda	3.307556
A_68_P06099969	Cebpa	3.298459
A_68_P08064720	6230410P16Rik	3.289929
A_68_P09413265	Adam11	3.281643
A_68_P07384679	Pabpnl1	3.251871
A_68_P05678119	Inmt	3.241554
A_68_P04277065	Cxcl1	3.232483
A_68_P03300156	Anp32b	3.218152
A_68_P01342780	Ccbl1	3.191739
A_68_P13013556	Ppl	3.188637
A_68_P06983908	5033428A16Rik	3.184366
A_68_P08717087	Tcba1	3.162999
A_68_P01619771	Insm1	3.162789
A_68_P00991622	Capn8	3.147283
A_68_P09483427	Sfrs2-2600014M03Rik	3.144074
A_68_P05338523	Cmas	3.141646
A_68_P00961427	Tada1l	3.138739
A_68_P14332767	Ttc9c	3.115044
A_68_P05996158	Flt3l	3.111308
A_68_P14836142	Trex2	3.110182

A_68_P14217831	Klf9	3.106999
A_68_P09257037	ENSMUST00000083595.1:-5713	3.098821
A_68_P09804391	Siva1	3.083022
A_68_P01293546	A530013C23Rik	3.051482
A_68_P06041220	0610012D14Rik	3.030374
A_68_P04831083	Fndc4-Gckr	3.023787
A_68_P09606302	Ern1	3.02252
A_68_P03863568	Il11ra1	3.013612
A_68_P12156053	Tigd5	3.005614
A_68_P08449896	Foxo3a	2.996945
A_68_P09503245	BC018371	2.995543
A_68_P03733127	Rnf186	2.995411
A_68_P12138338	Hoxc13	2.993144
A_68_P02980842	Pex2	2.984359
A_68_P13594311	Lipg	2.981591
A_68_P08110716	Acp5	2.979295
A_68_P12843197	Ehhadh	2.979046
A_68_P13107615	Ticam1	2.974038
A_68_P01585335	NM_027230:-12014	2.964752
A_68_P07561473	2310005P05Rik	2.959764
A_68_P06473014	Slc27a5	2.956857
A_68_P11399794	Gjb2	2.954948
A_68_P05596974	Rnf103	2.947421
A_68_P01776420	Tcf15	2.943853
A_68_P03670798	Masp2	2.938985
A_68_P10766257	Slc12a7	2.938504
A_68_P07467134	Slc38a3	2.929505
A_68_P06023375	Insc	2.920786
A_68_P09210759	Ccdc49	2.912065
A_68_P01440416	Fbxw2	2.910985
A_68_P05640423	Ldhb	2.910001
A_68_P03300156	Anp32b	2.903748
A_68_P01361686	Agpat2	2.896085
A_68_P06318305	Igf1r	2.89603
A_68_P09383678	Pdk2	2.895932

A_68_P01139487	Wnt6	2.894217
A_68_P08183902	Hyal1	2.88674
A_68_P13152306	Slc9a3r2	2.882054
A_68_P04831083	Fndc4-Gckr	2.881455
A_68_P06328757	B3gnt8	2.878127
A_68_P06495435	Anpep	2.867574
A_68_P10662976	Fgfr4	2.866971
A_68_P13494922	Spsb3	2.857541
A_68_P06677187	Agt	2.848927
A_68_P01655861	F730014I05Rik	2.847079
A_68_P13428964	1700001C19Rik	2.840882
A_68_P02134180	Abl1	2.834028
A_68_P01902701	2700007P21Rik	2.830826
A_68_P09480262	BC018371	2.815238
A_68_P07697209	Acaa1a	2.814605
A_68_P04661116	Sema3c	2.812567
A_68_P00641555	Nr1i3	2.806854
A_68_P07561489	2310005P05Rik	2.782162
A_68_P11169779	Ndst2	2.764472
A_68_P04323144	Sult1d1	2.758509
A_68_P09136314	Baiap2	2.757393
A_68_P08824256	Traf3ip2	2.755806
A_68_P03522568	Nbl1	2.753369
A_68_P04222183	Smarcd3	2.748406
A_68_P09295953	Pctp	2.747116
A_68_P09214836	Mpdu1	2.738918
A_68_P04323144	Sult1d1	2.738819
A_68_P03250140	Inpp5b	2.721431
A_68_P01176002	Clp1	2.71744
A_68_P07734324	Tusc2	2.710532
A_68_P12240032	Eppk1	2.706811
A_68_P07772617	Rab39	2.702879
A_68_P04590703	Stx1a	2.69696
A_68_P06376689	Npas1	2.691118
A_68_P13228675	Chd1	2.68884

A_68_P01309646	Gzf1	2.685509
A_68_P02564830	Igsf3	2.676821
A_68_P09148759	Igtp	2.671107
A_68_P01342773	Ccbl1	2.670489
A_68_P00418602	Adora1	2.670161
A_68_P10556434	Rnf44-BC040758	2.662607
A_68_P07124748	Lypla3	2.661755
A_68_P12476467	Pigp	2.639006
A_68_P04081647	Dio1	2.635988
A_68_P12635594	Ehhadh	2.633028
A_68_P13251244	Cyp4f13	2.628512
A_68_P02865901	Mef2d	2.618358
A_68_P05978278	Chst8	2.614958
A_68_P08437597	Trhde	2.610955
A_68_P05278653	Capza2	2.607221
A_68_P09559562	Shmt1	2.605692
A_68_P08223987	Wdr18	2.590578
A_68_P07306669	Car5a-Banp	2.58095
A_68_P13059000	Hmga1	2.567957
A_68_P02944888	Prune	2.565067
A_68_P08388906	1810043G02Rik	2.563427
A_68_P02726221	Dclre1b	2.560761
A_68_P09729896	Phb	2.560086
A_68_P11932638	Cacnb3	2.557921
A_68_P09845387	Ppp1r13b	2.557738
A_68_P10702707	Slc35d2	2.533268
A_68_P05433825	Gm839	2.533074
A_68_P00714552	Uap1	2.530881
A_68_P12051103	Naprt1	2.525914
A_68_P05893671	6330512M04Rik	2.521061
A_68_P04983819	Abcb9	2.518814
A_68_P07254767	Elmod2	2.51774
A_68_P08425563	Lss	2.511958
A_68_P08614767	Rap1b	2.496942
A_68_P10863296	Lect2	2.496456

A_68_P06873958	Ranbp10	2.496356
A_68_P02093470	U46068	2.488964
A_68_P09612757	Pcyt2	2.481565
A_68_P01797463	A530013C23Rik	2.481148
A_68_P08173101	Cyp8b1	2.475308
A_68_P13251243	Cyp4f13	2.475173
A_68_P05466381	Zc3hc1	2.471338
A_68_P05358495	Rassf8	2.470641
A_68_P09500328	Tspan10	2.468053
A_68_P03296780	Klhl9	2.464396
A_68_P08624242	Apba3	2.463312
A_68_P05439808	Irak2	2.457734
A_68_P08376605	Lrrc3	2.455004
A_68_P14081599	5730405I09Rik	2.449208
A_68_P13093038	1600002H07Rik	2.446507
A_68_P04114789	Add1	2.444557
A_68_P11865033	Lgals2	2.443036
A_68_P04107032	0610009O03Rik	2.441583
A_68_P04403090	Slc2a9	2.440666
A_68_P07946873	Mst1	2.440302
A_68_P02060698	4933440H19Rik	2.437239
A_68_P09398667	2210020M01Rik	2.434596
A_68_P05945054	Usf2	2.434095
A_68_P04390199	Ung	2.429486
A_68_P08533341	Nodal	2.424166
A_68_P03513611	St3gal3	2.422837
A_68_P04069200	Klf4	2.422161
A_68_P06624530	Centd2	2.420805
A_68_P09252755	ENSMUST00000100735.1:-6717	2.420324
A_68_P01582438	Surf2	2.417966
A_68_P02999674	Slc10a5	2.415681
A_68_P03087438	Pklr	2.408139
A_68_P06061704	Myo7a	2.407684
A_68_P08415566	Mettl7b	2.405015
A_68_P04107032	0610009O03Rik	2.402721

A_68_P05972322	Xylt1	2.401768
A_68_P02025392	Ntng2	2.399047
A_68_P03632444	2810405K02Rik	2.398607
A_68_P03359505	Trim62	2.398127
A_68_P12677554	Mrap	2.392233
A_68_P02764963	Tbl1xr1	2.387782
A_68_P09350828	Smcr8	2.374491
A_68_P13172382	Rhot2	2.366167
A_68_P10532742	Auh	2.364403
A_68_P06683149	Gtl3	2.357396
A_68_P10735929	Hmgcr	2.35734
A_68_P07142897	Taf1c	2.354426
A_68_P03496599	Il22ra1	2.347343
A_68_P11308601	Tgm1	2.346194
A_68_P06523791	Zfp94	2.343766
A_68_P02904357	Kcna3	2.335647
A_68_P02154342	NM_027230:-12364	2.329461
A_68_P01628111	Dab2ip	2.328603
A_68_P10573446	Larp5	2.324369
A_68_P08191110	Ifrd2	2.323141
A_68_P09697830	Sec14l4	2.32306
A_68_P00396257	Hhat	2.321683
A_68_P08064723	6230410P16Rik	2.320892
A_68_P14421027	Pdlim1	2.320329
A_68_P04909167	D5Wsu178e	2.319522
A_68_P09195968	Asgr1	2.312983
A_68_P01772350	Lcn12	2.312137
A_68_P07306676	Car5a	2.309876
A_68_P09350836	Smcr8	2.309123
A_68_P07248138	Kifc3	2.303954
A_68_P02454024	Mtmr11	2.296738
A_68_P04909167	D5Wsu178e	2.295616
A_68_P01700350	Set	2.294143
A_68_P09739582	Nfe2l1	2.292697
A_68_P03359505	Trim62	2.291905

A_68_P06372045	C730027J19Rik	2.291516
A_68_P09082170	Pitpna	2.287789
A_68_P01943462	Ctsz	2.281854
A_68_P12672593	Polr2h	2.276607
A_68_P08834117	Ddit4	2.268307
A_68_P04318728	Pebp1	2.267146
A_68_P13546277	Ptk7	2.260121
A_68_P02116222	Slco4a1	2.25961
A_68_P13319163	9530058B02Rik	2.248109
A_68_P06653585	Plekha4	2.243493
A_68_P07309218	Kifc3	2.242923
A_68_P06804280	Irf2	2.242819
A_68_P04608378	Rnf6-Cdk8	2.235086
A_68_P12448089	Csnk1e	2.230008
A_68_P04899482	Brap	2.227235
A_68_P08628191	Cdc34	2.223585
A_68_P04764801	Sh2b2	2.223334
A_68_P15086158	Bcor	2.222255
A_68_P03401491	Zdhhc18	2.222238
A_68_P09410666	Ankrd43	2.22061
A_68_P10015672	Cdc42bpb	2.215493
A_68_P06388543	Fgf21	2.214782
A_68_P09975941	Crip1	2.214291
A_68_P04899482	Brap	2.209027
A_68_P13235156	Ccnd3	2.208879
A_68_P14357243	Stx5a	2.200926
A_68_P01628078	Dab2ip	2.200786
A_68_P01375793	Serping1	2.200737
A_68_P12877631	Ahsg	2.198088
A_68_P14420129	Ldb1	2.197755
A_68_P07884006	Csk	2.188885
A_68_P03078310	Tnfaip8l2	2.18658
A_68_P13425194	Acat3	2.179741
A_68_P11522706	Clu	2.1793
A_68_P00493944	Hs6st1	2.178304

A_68_P01788371	Dgkz	2.175455
A_68_P07210532	F10	2.171598
A_68_P09009810	Asgr1	2.169984
A_68_P00458829	Rab17	2.165423
A_68_P03987047	Arhgef19	2.164841
A_68_P07306600	Pde4c	2.164265
A_68_P13155345	A_68_P13155345	2.162256
A_68_P04389279	Fscn1	2.161226
A_68_P15185453	Tmem47	2.161007
A_68_P05514834	4921507P07Rik	2.160907
A_68_P06314101	Myo7a	2.154651
A_68_P08793639	Mon2	2.149339
A_68_P13292578	Rnps1	2.145298
A_68_P02025387	Ntng2	2.141241
A_68_P07614068	Trex1	2.137033
A_68_P09656833	Agxt2l2	2.130865
A_68_P12721918	1500031L02Rik	2.130426
A_68_P03318061	Rcc2	2.12795
A_68_P05965663	Slc7a10	2.127828
A_68_P01367667	Rbck1	2.125261
A_68_P06020155	Slc22a18	2.125127
A_68_P06397866	Ldha	2.125032
A_68_P14742409	Pdzx	2.124636
A_68_P04003490	Efhd2	2.122804
A_68_P11351920	Ube2e1	2.122718
A_68_P11100986	Inhba	2.119196
A_68_P06017740	Ppp2r2d	2.117704
A_68_P06814843	Ocel1	2.116272
A_68_P06827522	Pcoln3	2.113259
A_68_P06928371	Atp6v0d1	2.111896
A_68_P05251168	8430408G22Rik	2.109618
A_68_P05441762	St8sia1	2.108445
A_68_P06390438	AI428936	2.108242
A_68_P01293536	A530013C23Rik	2.105704
A_68_P06046623	Ppp2r2d	2.105225

A_68_P09459558	Tcf7	2.102402
A_68_P06275539	Saa3	2.097308
A_68_P13425877	Klc4	2.097008
A_68_P13249138	Tap2	2.093152
A_68_P09709913	Igfbp1	2.092937
A_68_P01495917	Spag4l-Bpil1	2.092138
A_68_P10754219	Peci	2.090161
A_68_P12117966	LOC554292	2.08931
A_68_P10642062	Lrrc16	2.088253
A_68_P00208770	Serpinb8	2.085657
A_68_P08262151	Mdm1	2.082818
A_68_P06599541	Armc5	2.082517
A_68_P03730091	Ube2j2	2.078767
A_68_P09582284	2010305C02Rik	2.078021
A_68_P12362832	Gpt1	2.077018
A_68_P03197768	Mtmr11	2.075908
A_68_P08308992	Tdg	2.074748
A_68_P09013605	Higd1b	2.073842
A_68_P00634491	Tmem58	2.073557
A_68_P03682005	Dscr1l2	2.069652
A_68_P05591343	Zyx	2.067755
A_68_P06369053	Zdhhc13	2.06556
A_68_P00619218	Nek2	2.060574
A_68_P13711135	E230022H04Rik	2.059753
A_68_P11658285	Kcnk5	2.054507
A_68_P06049341	Egln2	2.054385
A_68_P08692362	Bloc1s1-Itga7	2.052585
A_68_P13441074	Pacsin1	2.051962
A_68_P08454871	Nodal	2.041094
A_68_P13752126	2810439F02Rik	2.040437
A_68_P05495854	Casd1	2.039907
A_68_P08803717	Hsd17b6	2.039646
A_68_P06584985	Ppfibp2	2.038819
A_68_P02103490	Notch1	2.032303
A_68_P08446465	Ncln	2.029448

A_68_P05565706	A230083G16Rik	2.029003
A_68_P14536587	Atp11c	2.028972
A_68_P04533410	2610524H06Rik	2.025811
A_68_P01406624	Bmf	2.015335
A_68_P07006300	Indol1	2.014286
A_68_P06468835	Homer2	2.013563
A_68_P01757589	1110002H13Rik-Adamtsl2	2.011019
A_68_P08903907	Tspan31-Centg1	2.010663
A_68_P09697832	Sec14l4	2.009106
A_68_P03852202	Tbc1d2	2.006292
A_68_P06336609	Gtf2h1	2.004562
A_68_P07017381	Slc20a2	2.002702
A_68_P09264444	Srebf1	1.998832
A_68_P14153156	Chuk	1.997575
A_68_P04107141	Dnajb6	1.997288
A_68_P13728082	Lims2	1.995951
A_68_P03496599	Il22ra1	1.991967
A_68_P09485551	Mafg	1.987257
A_68_P03492105	Galt	1.986888
A_68_P03361643	Clta	1.983516
A_68_P09795589	1110008B24Rik	1.979197
A_68_P00207772	Lactb2	1.978465
A_68_P05754477	C1rl	1.974813
A_68_P12046837	ENSMUST00000083630.1:-1410	1.974438
A_68_P11042264	Jmy	1.972869
A_68_P09425721	2610019A05Rik	1.96943
A_68_P03228980	Acot7	1.969102
A_68_P06922348	Insl3	1.968286
A_68_P07372436	Ces6	1.962542
A_68_P06438929	Cdkn1c	1.962174
A_68_P08546101	Rdh7	1.962033
A_68_P03940509	Adc	1.960995
A_68_P07154152	Eif4ebp1	1.960891
A_68_P04111770	Lrrc8d	1.956755
A_68_P10323285	Rps6ka5	1.956488

A_68_P12306569	Cmbl	1.956434
A_68_P12256637	Lifr	1.955564
A_68_P02046867	Egfl7	1.953733
A_68_P02200339	Sema6d	1.950511
A_68_P07002293	Terf2	1.949812
A_68_P04671642	Unc84a	1.94961
A_68_P10006538	Rps6ka5	1.949041
A_68_P04155063	Evi5	1.949028
A_68_P03513151	Elovl1	1.946778
A_68_P09689162	Hoxb2	1.9445
A_68_P10113544	Bag5	1.944178
A_68_P00303681	Igfbp5	1.942407
A_68_P01491133	Acvr2a	1.942059
A_68_P06682236	Ing1	1.941668
A_68_P10249145	Tyki	1.940866
A_68_P08621775	Nab2	1.936782
A_68_P02482324	Pip5k1b-Vps72	1.934422
A_68_P11477858	Tpt1	1.930782
A_68_P04764801	Sh2b2	1.93055
A_68_P03059990	Ptgfr	1.928532
A_68_P05057762	Hk2	1.927756
A_68_P03489466	Ahdc1	1.925808
A_68_P00625151	Centg2	1.921725
A_68_P13127101	Csnk2b	1.921417
A_68_P00372598	Agxt	1.920314
A_68_P12905298	Rtp4	1.919099
A_68_P06382815	Cpeb1	1.919016
A_68_P11890247	Rbm9	1.917342
A_68_P00379196	Slc11a1	1.916193
A_68_P04271280	Mdh2	1.916104
A_68_P08571770	A630077B13Rik	1.91345
A_68_P13114736	Lama1	1.913136
A_68_P08296613	Hkdc1	1.91036
A_68_P05385669	Thnsl2	1.90859
A_68_P03285872	Tnfrsf1b	1.908378

A_68_P05599970	C1r	1.908325
A_68_P10002168	Dpf3	1.908274
A_68_P06160077	1600014C10Rik	1.907732
A_68_P09177526	Atp5g1	1.906731
A_68_P05419130	Pzp	1.90643
A_68_P06098635	Lrrc28	1.906233
A_68_P02764965	Tbl1xr1	1.905727
A_68_P08583824	Mbd3	1.903545
A_68_P03401481	Zdhhc18	1.90218
A_68_P01986063	Mcm8	1.899118
A_68_P12645029	Map6d1	1.898922
A_68_P09654823	AI595406	1.898247
A_68_P11173103	Abhd6	1.898195
A_68_P00656928	2500001K11Rik	1.897566
A_68_P14554864	Mid1ip1	1.894417
A_68_P09665920	Tubd1	1.894158
A_68_P03501268	Akr1a4	1.892023
A_68_P08777819	Nfic	1.890879
A_68_P07559872	Armet	1.890693
A_68_P06109595	Paox	1.889424
A_68_P13972593	Prrc1	1.888095
A_68_P04277062	Cxcl1	1.88786
A_68_P11927862	Prlr	1.885034
A_68_P13173432	Decr2	1.881355
A_68_P13014388	Atp6v1a-Nat13	1.881272
A_68_P04346560	Ankrd56	1.880436
A_68_P11937882	BC030396	1.879034
A_68_P03654137	St3gal3	1.877778
A_68_P13560632	Slc22a7	1.876822
A_68_P13251986	Srrm2	1.876383
A_68_P07269129	Ushbp1-5430437P03Rik	1.875962
A_68_P04346560	Ankrd56	1.873934
A_68_P07576746	Dcamkl3	1.873153
A_68_P04019601	Ccl27	1.87194
A_68_P02748644	Bcan	1.870519

A_68_P13442963	Clcn7	1.868043
A_68_P01088601	Rassf5	1.86686
A_68_P04398638	Evi5	1.866648
A_68_P06212420	Egln2	1.865091
A_68_P02826367	Fabp2	1.865027
A_68_P00746014	Il10	1.862417
A_68_P04336891	Pkd2	1.862199
A_68_P03303258	Errfi1	1.861575
A_68_P14403538	Cpt1a	1.857741
A_68_P06872367	Pcid2	1.85596
A_68_P11100963	Inhba	1.855446
A_68_P14334327	Acy3	1.855079
A_68_P10309955	Gsc	1.850686
A_68_P01122382	Srp9	1.850182
A_68_P06738728	Ddx28	1.848224
A_68_P07781934	Sidt2	1.846294
A_68_P08204430	Timp3	1.84597
A_68_P14484742	Dpf2	1.845285
A_68_P03773670	Tlr12	1.845033
A_68_P08023922	Smad6	1.843314
A_68_P03818525	Grhpr	1.842269
A_68_P06181223	Abcc6	1.83986
A_68_P06677165	Agt	1.838395
A_68_P12298037	Lynx1	1.836963
A_68_P09108875	Aatk	1.836674
A_68_P02842022	Sypl2	1.8352
A_68_P14361889	Chka	1.833384
A_68_P06558085	Cebpa	1.832806
A_68_P08991556	Mrps24	1.832014
A_68_P10735909	Hmgcr	1.83157
A_68_P03305252	Foxo6	1.830242
A_68_P12600288	Pcyt1a	1.830192
A_68_P08248705	3110056O03Rik	1.829078
A_68_P01293547	A530013C23Rik	1.828284
A_68_P06423010	Abcc8	1.826431

A_68_P08299334	1700021F05Rik	1.822089
A_68_P08165567	Usp4	1.821966
A_68_P09130970	Fads6	1.820172
A_68_P12651548	Pla1a	1.819904
A_68_P08936079	A_68_P08936079	1.818224
A_68_P01216441	Src	1.817777
A_68_P02694381	Magi3	1.816313
A_68_P02312547	Ptgfrn	1.814858
A_68_P09070782	Tbkbp1	1.814167
A_68_P05904665	Hpxn	1.813692
A_68_P12966444	Lpp	1.813202
A_68_P05896362	Apoc4	1.809549
A_68_P09488544	P4ha2	1.808212
A_68_P12046836	ENSMUST00000083630.1:-2118	1.8081
A_68_P14867946	Aff2	1.807574
A_68_P12056452	Slc39a4	1.807308
A_68_P03805515	Ski	1.80583
A_68_P04542655	Cdk8	1.805226
A_68_P09080012	Gtlf3b	1.804934
A_68_P12237143	Fkbp11	1.804871
A_68_P09404104	Gm2a	1.804803
A_68_P09017231	Gpx3	1.804258
A_68_P09641776	Asgr1	1.804029
A_68_P12129693	Csad	1.803261
A_68_P08777345	Sumo3	1.802674
A_68_P13297332	Slc37a1	1.802587
A_68_P03818525	Grhpr	1.801349
A_68_P02161296	Sulf2	1.801007
A_68_P05933804	Thrsp	1.799896
A_68_P00327181	Lemd1	1.799492
A_68_P10248166	Rnf113a2	1.79901
A_68_P00652222	Psen2	1.798472
A_68_P11066165	Mtap1b	1.797142
A_68_P01772352	Lcn12	1.795051
A_68_P13236370	1700001C19Rik	1.793518

A_68_P12129679	Csad	1.792381
A_68_P00418605	Adora1	1.791288
A_68_P05502098	Cops7a	1.790002
A_68_P13293578	1700012G19Rik	1.788277
A_68_P04144481	Slc34a2	1.786972
A_68_P08210289	9-Mar	1.785031
A_68_P09886396	2310044G17Rik	1.784424
A_68_P06924447	0710008K08Rik	1.784328
A_68_P09354866	Gucy2e	1.784312
A_68_P04763153	Rasal1	1.781054
A_68_P02272802	Plcb1	1.780157
A_68_P02375830	Pdzk1	1.77799
A_68_P04379119	Baiap2l1	1.777719
A_68_P07210518	F10	1.777548
A_68_P12216260	Hoxc8	1.777421
A_68_P06494002	Calm3	1.777314
A_68_P01772847	Ypel4	1.776762
A_68_P06431278	Zfp114	1.775592
A_68_P03590695	Nr0b2	1.7751
A_68_P07455723	Yap1	1.774206
A_68_P13504342	Tnfaip8l1	1.771455
A_68_P14258825	Mxi1	1.771178
A_68_P13499441	Slc29a1	1.769536
A_68_P04267219	Emid2	1.768563
A_68_P08580643	Amd1	1.768524
A_68_P04586643	Fbxl10	1.768373
A_68_P11723129	2600011E07Rik	1.767973
A_68_P00389624	Cxcr4	1.767453
A_68_P03791293	Ipo13	1.767418
A_68_P09312923	Cacng5	1.767256
A_68_P14089035	Myoz3	1.765482
A_68_P04617575	Recc1	1.764499
A_68_P08608506	Lrrc10	1.764487
A_68_P11342427	Samd4	1.763249
A_68_P09410100	Dlx3	1.763049

A_68_P14271769	Cstf2t	1.762759
A_68_P01722996	Pck1	1.762494
A_68_P07966190	Slc44a2	1.761603
A_68_P06656873	Gdpd5	1.761242
A_68_P06047855	Nupr1	1.761036
A_68_P09969351	Tmem18	1.761029
A_68_P09529776	Smarcd2-Tcam1	1.757541
A_68_P09422847	Atox1	1.757212
A_68_P07680483	Thy1	1.756134
A_68_P13909021	Pmaip1	1.75557
A_68_P09517349	1110020P15Rik	1.754214
A_68_P11380311	Itih4	1.752637
A_68_P11311203	Lrtm1	1.751689
A_68_P08315623	Ddt	1.749203
A_68_P06052290	Neud4	1.748895
A_68_P00899912	Cnnm4	1.748735
A_68_P06682522	Herpud1	1.748028
A_68_P10666960	Jarid2	1.747577
A_68_P06654366	Plekhg2	1.744997
A_68_P05299879	Hoxa3	1.744924
A_68_P13883453	Pcdha8	1.744072
A_68_P05472974	8430419L09Rik	1.743552
A_68_P03520477	Rgs3	1.742998
A_68_P04267219	Emid2	1.742067
A_68_P05519175	Mkln1	1.74127
A_68_P11308602	Tgm1	1.740967
A_68_P14899047	Tsc22d3	1.739306
A_68_P04617575	Recc1	1.737718
A_68_P00716971	Tnfrsf11a	1.736541
A_68_P00815120	Wdr26	1.736526
A_68_P10168388	Otub2	1.736092
A_68_P09199922	Pitpna	1.7346
A_68_P10251388	Glrx5	1.734567
A_68_P08952297	1700052N19Rik-Rmnd1	1.734292
A_68_P04925865	Ncor2	1.733403

A_68_P09891693	Cdkl1	1.733125
A_68_P08934064	Nuak1	1.733077
A_68_P07356450	Cfdp1	1.731121
A_68_P03386207	Ece1	1.730575
A_68_P13078804	0610016J10Rik	1.730287
A_68_P03307843	Hp1bp3	1.730017
A_68_P02469440	Atp1a1	1.729773
A_68_P02767804	Bmpr1b	1.72913
A_68_P00544739	Pde6d-Cops7b	1.728242
A_68_P08361803	Gamt-Dazap1	1.726767
A_68_P05059005	5730419I09Rik	1.724994
A_68_P03540115	Espn-Hes2	1.724554
A_68_P07890306	Axud1	1.72446
A_68_P13156383	Cryaa	1.720469
A_68_P02163464	D430028G21Rik	1.720247
A_68_P07643197	Ctdspl	1.718839
A_68_P11779362	Adcy4	1.716452
A_68_P09926629	BC048191:-5083	1.715671
A_68_P11619267	Cbln3	1.715361
A_68_P10384816	1700020O03Rik	1.71321
A_68_P09238680	Psmd11	1.711343
A_68_P10402504	Slc25a29	1.711268
A_68_P06373804	Ucp2	1.710468
A_68_P00556266	Ctdsp1	1.710246
A_68_P01887581	Shc4	1.708721
A_68_P11069909	Gcnt2	1.708597
A_68_P03791284	Ipo13	1.707785
A_68_P00527441	Tagln2	1.70755
A_68_P04024977	6230416J20Rik	1.707465
A_68_P08148553	Raver1	1.706901
A_68_P01061123	Fzd5	1.704739
A_68_P06628737	Ppme1-AU020772	1.704599
A_68_P14346830	Gpam	1.704094
A_68_P14285527	Slc3a2	1.703612
A_68_P02545158	Cttnbp2nl	1.703145

A_68_P03305252	Foxo6	1.701562
A_68_P13330489	Ehd3	1.700505
A_68_P04336891	Pkd2	1.700112
A_68_P02124248	Ndufa8	1.699006
A_68_P01491115	Acvr2a	1.698694
A_68_P06529572	LOC434179	1.697668
A_68_P13784416	Mapk4	1.69653
A_68_P04163382	Steap1	1.694702
A_68_P03213988	Eno1	1.694444
A_68_P05459814	Eif4e3	1.693537
A_68_P08019033	A_68_P08019033	1.692033
A_68_P02401061	Ccdc109b	1.690838
A_68_P13574622	Efna5	1.690617
A_68_P08138658	AI593442	1.690179
A_68_P07819977	Elovl5	1.690069
A_68_P09913941	Six4-Mnat1	1.689989
A_68_P15004402	Armcx6-Armcx3	1.689321
A_68_P09363837	Mapk7	1.689202
A_68_P12306344	Krt18	1.688743
A_68_P08744512	Ddo	1.688455
A_68_P03615374	Laptm5	1.687311
A_68_P11230853	Jub	1.68699
A_68_P12027743	Ghr	1.686145
A_68_P00114985	D1Ertd161e	1.685478
A_68_P06994523	Bst2	1.684881
A_68_P09384009	Kcnj2	1.68472
A_68_P05838606	Cd9	1.682847
A_68_P09497502	Krt23	1.68234
A_68_P11180833	Itih3	1.682029
A_68_P14334322	Acy3	1.681512
A_68_P09172525	Rmnd5b	1.681227
A_68_P11537411	Nisch-Tnnc1	1.680721
A_68_P01869763	2810030E01Rik-Mllt10	1.680439
A_68_P09245273	Lasp1	1.679214
A_68_P05945053	Usf2	1.679064

A_68_P03635289	Srrm1	1.678627
A_68_P08125332	Clk3	1.677992
A_68_P03011101	Tmem79	1.677599
A_68_P07200083	Snapc2	1.677559
A_68_P09221382	Lyrm7	1.676536
A_68_P04024977	6230416J20Rik	1.674557
A_68_P08817347	Upb1	1.67346
A_68_P02369472	Palmd	1.673346
A_68_P10147981	Dnmt3a	1.673243
A_68_P06416571	Anpep	1.671084
A_68_P12980215	Cdgap	1.670838
A_68_P02537358	Il6ra	1.670733
A_68_P09177531	Atp5g1	1.668944
A_68_P13351723	Wdr24	1.668001
A_68_P02691140	D3Ucla1	1.66768
A_68_P03795291	Pink1	1.667548
A_68_P07629982	Tmed1	1.666914
A_68_P08935606	1700060H10Rik	1.66646
A_68_P14501275	Syvn1	1.66549
A_68_P05154910	Hnrpf	1.665104
A_68_P06166301	Sphk2	1.66475
A_68_P02004607	Gm1631	1.664048
A_68_P08893436	Dos-Atp5d	1.663782
A_68_P04763153	Rasal1	1.66365
A_68_P10211435	Slc25a29	1.661095
A_68_P10461509	Arid4b	1.661061
A_68_P11529693	Synpo2l-Sec24c	1.660902
A_68_P12080801	Krt18	1.660603
A_68_P07506791	Cck	1.659952
A_68_P13297330	Slc37a1	1.659631
A_68_P10017918	2610204M08Rik	1.659385
A_68_P03611397	4833401D15Rik	1.658251
A_68_P06765322	Wwc2	1.657722
A_68_P12188742	Slc39a4	1.657115
A_68_P13415559	Sepx1	1.656762

A_68_P09086358	Gas2l1	1.65623
A_68_P09185997	Tmem97	1.656213
A_68_P10437458	Slc35b3	1.65504
A_68_P11551201	Ccdc25	1.653605
A_68_P14474892	Dak	1.653378
A_68_P01237936	Hnrpa3	1.651356
A_68_P09442851	Map2k4	1.650757
A_68_P11947806	Cacnb3	1.649653
A_68_P00924507	Efhd1	1.648142
A_68_P03502660	Aldh1b1	1.647413
A_68_P02586990	Tmem144	1.647299
A_68_P09428652	Nme1	1.647239
A_68_P03145504	Otud7b	1.647043
A_68_P09134930	Scn4a	1.64704
A_68_P07391807	Def8	1.646458
A_68_P03590695	Nr0b2	1.645978
A_68_P12341810	Plec1	1.645839
A_68_P14176669	Ehd1	1.645826
A_68_P11574765	Slc39a14	1.645729
A_68_P06441611	Mia1	1.645333
A_68_P04089283	Tnfrsf18	1.645211
A_68_P03811860	Lck	1.642307
A_68_P07588985	Birc3	1.64194
A_68_P08949151	Rassf3	1.641748
A_68_P02046877	Egfl7	1.640819
A_68_P04377705	Fbxl10	1.640446
A_68_P14149983	Tle4	1.639549
A_68_P08672889	Wibg	1.638666
A_68_P04742466	Ywhag	1.638592
A_68_P12599267	Etv5	1.638314
A_68_P03758436	Arid1a	1.637901
A_68_P06095079	Aldoa	1.637617
A_68_P00761110	5033414K04Rik	1.637491
A_68_P08676565	9030224M15Rik	1.635884
A_68_P04156720	Tpst2	1.635374

A_68_P13999799	Dpysl3	1.635239
A_68_P06092905	Rhpn2	1.635174
A_68_P08833006	Bsg	1.634862
A_68_P06165341	Dbp	1.634037
A_68_P03726231	Ptprf	1.633009
A_68_P04515316	Cdx2	1.632768
A_68_P11226211	Hs6st3	1.632336
A_68_P09245907	Ifi47	1.631707
A_68_P02581367	Wnt2b	1.63129
A_68_P09621434	Ttc1	1.630926
A_68_P07012772	Hand2	1.630586
A_68_P06242258	Ins2	1.630464
A_68_P07189492	BC015286	1.629805
A_68_P03513612	St3gal3	1.629487
A_68_P05437345	Rtkn	1.629321
A_68_P02046834	Egfl7	1.628973
A_68_P08026226	Sema3b	1.62869
A_68_P09641035	Tlx3	1.627931
A_68_P13977871	Pcdhga5	1.627913
A_68_P01781180	Bcas1	1.627627
A_68_P07581377	Zmynd10	1.627558
A_68_P10699562	Isca1	1.626009
A_68_P04761491	Sds	1.625947
A_68_P10050045	Etv1	1.623652
A_68_P03233222	Lyn	1.623174
A_68_P11831952	Elp3	1.622957
A_68_P09565009	D11Ertd636e	1.622855
A_68_P03531428	Capzb	1.622461
A_68_P08201798	Mon2	1.622447
A_68_P08395271	Smarcc2	1.622296
A_68_P07684876	Tgm4	1.620179
A_68_P04458056	Tomm7	1.619853
A_68_P04372701	Atp5k	1.619247
A_68_P03341194	Trp53inp1	1.618632
A_68_P12162253	Krt74	1.61845

A_68_P06203949	Pnkp	1.618388
A_68_P09561658	Slc22a4	1.618194
A_68_P14354305	Rad9	1.618031
A_68_P08064730	6230410P16Rik	1.617821
A_68_P06221575	2400001E08Rik	1.617249
A_68_P02480010	Bmpr1b	1.615811
A_68_P11457646	Gja3	1.615353
A_68_P09179041	1110067D22Rik	1.615282
A_68_P03176910	6530418L21Rik	1.615154
A_68_P08147797	Trf	1.614962
A_68_P14263582	Foxb2	1.614787
A_68_P02773136	5730470L24Rik	1.614178
A_68_P11133900	Lect1	1.614083
A_68_P14186918	Acsl5	1.612814
A_68_P12590377	Dnm1l	1.612755
A_68_P08914346	Aifm2	1.612555
A_68_P01367672	Rbck1	1.612233
A_68_P06233679	A_68_P06233679	1.611892
A_68_P02025412	Ntng2	1.611273
A_68_P10974751	Prr7	1.61105
A_68_P10880824	BC005537	1.610679
A_68_P10689991	Aldh5a1-Gpld1	1.610133
A_68_P11082814	Snag1	1.608855
A_68_P06048158	Pak4	1.608737
A_68_P12250087	Nup50	1.608595
A_68_P13348089	Slc29a1	1.608509
A_68_P12267844	Krt18	1.608247
A_68_P04458056	Tomm7	1.606483
A_68_P06519319	Fes	1.606293
A_68_P04044353	Med18	1.605962
A_68_P09933404	Cdc42bpb	1.605505
A_68_P10942741	Tmed9	1.603585
A_68_P13382698	BC048355	1.603392
A_68_P04761491	Sds	1.603189
A_68_P07267949	Adat1	1.602997

A_68_P11632582	Zfp395	1.602811
A_68_P08125324	Clk3	1.602191
A_68_P06322245	Rrm1	1.601041
A_68_P10261319	Vipr2	1.600963
A_68_P10290413	Zfyve26	1.600039
A_68_P04730242	Otop1	1.599816
A_68_P04551716	Crybb3	1.599298
A_68_P09870970	Tspan13	1.598295
A_68_P00312340	Arpc5	1.597096
A_68_P00401602	En1	1.596417
A_68_P00310817	Klf7	1.595597
A_68_P10946345	A530095I07Rik	1.595489
A_68_P08152699	Cep57	1.595465
A_68_P03053569	Crct1	1.595044
A_68_P07678840	2010110K16Rik	1.595029
A_68_P12449654	Ankrd46	1.593971
A_68_P01379456	Nat5	1.593333
A_68_P02064375	2610510H03Rik	1.592596
A_68_P06087354	Pnpla2	1.592196
A_68_P07069507	Irf8	1.591495
A_68_P14414972	Ehbp1l1	1.590989
A_68_P00953228	Exo1	1.590859
A_68_P09504059	Kif1c	1.590584
A_68_P10820641	Zfp708	1.590447
A_68_P14368537	Ankrd1	1.589893
A_68_P08321588	BC025920	1.589707
A_68_P03370361	Cer1	1.588658
A_68_P09786813	Ptpn21	1.587859
A_68_P13092632	2610110G12Rik	1.58777
A_68_P02581370	Wnt2b	1.587644
A_68_P01442677	Adam33	1.586011
A_68_P09235545	Sectm1b	1.585228
A_68_P03978335	Rbm35a	1.58381
A_68_P03349816	Hkr3	1.583474
A_68_P05279250	Sfxn5	1.583147

A_68_P10772170	Gpbp1	1.583001
A_68_P03611383	9530048O09Rik-4833401D15Rik	1.582838
A_68_P02139611	Cry2	1.582598
A_68_P05979597	Lsr	1.582332
A_68_P14345132	Pcgf5	1.582328
A_68_P04671714	Ttyh3	1.582185
A_68_P08815201	Egr2	1.582126
A_68_P08314943	Oaz1	1.581132
A_68_P11000432	Elmo1	1.580518
A_68_P10671048	Shc3	1.58047
A_68_P10574240	Trim27	1.580338
A_68_P09477165	Med11	1.580178
A_68_P13515767	Psmb9	1.579019
A_68_P03670784	Masp2	1.578955
A_68_P11760124	Tssk4	1.577968
A_68_P14670347	Ammecr1	1.576865
A_68_P13083393	Rpp21	1.576289
A_68_P13732806	Cdc25c	1.576092
A_68_P06734330	Gtf2e2	1.575478
A_68_P08039933	AU019823	1.57489
A_68_P09349695	2400006H24Rik	1.574472
A_68_P09409119	Gprc5c	1.574418
A_68_P14460066	Sfrp5	1.574369
A_68_P06872414	Efcbp2	1.574224
A_68_P07411612	5830416A07Rik	1.573943
A_68_P09982964	2410016O06Rik	1.573101
A_68_P10302919	Ppp2r5c	1.573089
A_68_P01214326	Nmt2	1.573085
A_68_P09071035	Phospho1	1.572794
A_68_P05553670	Rarres2	1.572551
A_68_P14232405	Gpr137	1.57222
A_68_P00303683	Igfbp5	1.571916
A_68_P02409507	Sox2	1.571894
A_68_P12201222	Cdc42ep1	1.57174
A_68_P04308259	D830046C22Rik	1.571199

A_68_P03160430	Mab21l1	1.571106
A_68_P06807426	Lamp1	1.569412
A_68_P14319765	Ifit2	1.569215
A_68_P10126148	Lamb1-1	1.568291
A_68_P13728079	Lims2	1.568223
A_68_P03705807	Bsdc1	1.56785
A_68_P04742466	Ywhag	1.56752
A_68_P04334909	Slc2a9	1.566539
A_68_P08124293	2410004A20Rik	1.56634
A_68_P12293869	Cyp2d26	1.566215
A_68_P01505261	Fbxw5	1.566118
A_68_P06837568	Mtnr1a	1.564454
A_68_P01772363	Lcn12	1.562675
A_68_P04308259	D830046C22Rik	1.562517
A_68_P01313060	Slc35c1	1.562355
A_68_P12438209	Pphln1	1.561591
A_68_P00578461	Zc3h11a	1.561212
A_68_P14045959	4933403F05Rik	1.560919
A_68_P07137852	2010315L10Rik	1.560897
A_68_P08376641	Lrrc3	1.55967
A_68_P07835094	Acy1	1.558895
A_68_P04384115	3732412D22Rik	1.557034
A_68_P07099616	1500041N16Rik	1.555909
A_68_P00932178	Epha4	1.555378
A_68_P02868715	Pklr	1.555266
A_68_P02248603	Snrpb	1.555167
A_68_P08779311	Cradd	1.554816
A_68_P00372593	Agxt	1.553863
A_68_P03370361	Cer1	1.55371
A_68_P12133074	3-Sep	1.553401
A_68_P03580331	Btf3l4	1.553071
A_68_P09287289	Tk1	1.55279
A_68_P11132853	5730469M10Rik	1.552665
A_68_P10610122	Mxd3	1.552372
A_68_P10924386	Gpbp1	1.552291

A_68_P08007768	Sidt2	1.552155
A_68_P00625152	Centg2	1.552045
A_68_P12045613	Tenc1	1.551177
A_68_P06818505	Usp10	1.551129
A_68_P06782036	Alg11	1.550423
A_68_P05216651	BC049816	1.550087
A_68_P11336884	Dhrs1	1.55008
A_68_P06152810	Prkcdbp	1.549171
A_68_P12311790	Plec1	1.548824
A_68_P08826569	L3mbtl3	1.54857
A_68_P04557067	Srd5a2l	1.548408
A_68_P08161434	Wdr51a	1.547545
A_68_P13855408	BC031181	1.547384
A_68_P06788628	BC021891	1.54653
A_68_P06485428	Arrb1	1.546202
A_68_P07987068	Pdcd7	1.545832
A_68_P08755354	Popdc3	1.545523
A_68_P09736545	Abca8b	1.545125
A_68_P05919592	Dctn5	1.545026
A_68_P04333196	1110008J03Rik	1.544959
A_68_P08235834	Ccdc38	1.54484
A_68_P02273373	2610042O14Rik	1.544522
A_68_P13879047	Ccdc68	1.544123
A_68_P08906221	Csnk1g2	1.543529
A_68_P14228457	Rrp12	1.543515
A_68_P02828323	Tnfsf10	1.543267
A_68_P03787366	Ssbp3	1.543113
A_68_P03853104	Tinagl	1.542871
A_68_P08881687	Ppa1	1.542429
A_68_P07908610	Slc44a2	1.541975
A_68_P01943459	Ctsz	1.541802
A_68_P12809031	Chrd	1.541505
A_68_P13871637	Ndst1	1.541193
A_68_P06002891	Actn4	1.541084
A_68_P14311529	Fkbp2	1.540579

A_68_P03883233	Gpr157	1.540441
A_68_P13525866	Snrpc	1.540202
A_68_P09598068	Acox1	1.539831
A_68_P11835015	Ppif	1.539661
A_68_P00783015	Farp2	1.539309
A_68_P03491937	2610528J11Rik	1.539283
A_68_P12250659	9330154K18Rik	1.539247
A_68_P06871160	Bst2-1110012M11Rik	1.538792
A_68_P06435140	Arhgef1	1.537806
A_68_P05740623	Tmem43	1.537391
A_68_P03303259	Errfi1	1.537191
A_68_P03747745	Ptprf	1.536366
A_68_P05919068	C79127-Rtn2	1.535761
A_68_P08883482	Nhsl1	1.535163
A_68_P08377706	Tle6	1.53495
A_68_P13080463	Abcg8	1.534941
A_68_P09119456	Spata20	1.534564
A_68_P11846764	Cerk	1.533896
A_68_P04730667	Ddx55	1.533874
A_68_P09320523	Dynll2	1.533421
A_68_P02633829	Palmd	1.533341
A_68_P14826906	Gprasp1	1.532992
A_68_P04650751	Ppargc1a	1.532554
A_68_P09492905	Psmd3	1.532448
A_68_P14042388	Ier3ip1	1.532122
A_68_P07829944	Paqr9	1.532087
A_68_P11845292	Krt84	1.532078
A_68_P06049595	Ctbp2	1.531918
A_68_P04576046	Wsb2	1.531885
A_68_P09926608	BC048191:-5384	1.53188
A_68_P05547809	Lrrc23	1.531873
A_68_P03371533	Tox	1.531639
A_68_P05591340	Zyx	1.531621
A_68_P00445025	H3f3a	1.531609
A_68_P10927198	ENSMUST00000091828.3:101286	1.530827

A_68_P03773699	Tlr12	1.530769
A_68_P14455471	Dmrt3	1.530661
A_68_P01584465	Gata3	1.530412
A_68_P12189033	Card10	1.52987
A_68_P11966468	Bik	1.529802
A_68_P14148167	Zdhhc6	1.529502
A_68_P06583812	Zfp84	1.529052
A_68_P04306525	Zan	1.529023
A_68_P09168843	2810408A11Rik	1.528653
A_68_P09702045	9530068E07Rik	1.528526
A_68_P09234373	Lrrc59	1.526122
A_68_P10584493	F12	1.526038
A_68_P04552046	Tpst2	1.525704
A_68_P05762546	Lrrtm1	1.525479
A_68_P07437462	Brd7	1.525369
A_68_P01808653	Edem2	1.524744
A_68_P13246201	Slc9a3r2	1.524637
A_68_P04706193	Denr	1.52461
A_68_P06687682	Cyld	1.524472
A_68_P07173318	Zfp709	1.523687
A_68_P00702326	ENSMUST00000083599.1:-2617	1.523507
A_68_P07584309	Azi2	1.522844
A_68_P01823173	Zc3h8	1.522733
A_68_P13436122	Xdh	1.522665
A_68_P06862966	Lcat	1.522482
A_68_P14155349	Catsper1	1.521839
A_68_P06176024	Tbc1d10b	1.521695
A_68_P10388625	Psma3	1.521571
A_68_P04627439	Perq1	1.52125
A_68_P10886970	Thoc3	1.521146
A_68_P06125211	Athl1	1.520824
A_68_P01774410	5430432M24Rik	1.520228
A_68_P01269454	Defb25-Rem1	1.519859
A_68_P12204213	Sh3bp1	1.519619
A_68_P05472973	8430419L09Rik	1.519542

A_68_P02567272	Ptbp2	1.519195
A_68_P02189755	Hrh3	1.518957
A_68_P12255657	Baiap2l2	1.518597
A_68_P12323106	Jrk	1.517152
A_68_P11167360	Rpl15	1.516853
A_68_P13720085	Hsd17b4	1.51632
A_68_P14319570	Fen1	1.516319
A_68_P09457808	E230016K23Rik	1.516003
A_68_P11018859	Ibrdc2	1.515571
A_68_P08297291	Map2k2	1.515346
A_68_P08147904	Sacm1l	1.515304
A_68_P15093622	Nxt2	1.514999
A_68_P12929782	Ap2m1	1.514902
A_68_P06975176	Cherp	1.514495
A_68_P09689731	Cygb	1.514231
A_68_P09031138	Rac3	1.514027
A_68_P14462408	Rtn3	1.513942
A_68_P11125550	Dzip1	1.513526
A_68_P06920823	Irf8	1.513158
A_68_P04081645	Dio1	1.512652
A_68_P13533861	Ddx11	1.51247
A_68_P13204986	4931440B09Rik	1.511919
A_68_P10148637	Six1	1.511655
A_68_P09309976	Krt35	1.510478
A_68_P01127569	Tsga10-EG623661	1.510223
A_68_P02450195	Tspan5	1.510213
A_68_P13195944	Tsc2	1.510209
A_68_P04706193	Denr	1.509838
A_68_P00372595	Agxt	1.509235
A_68_P09212392	Atox1-G3bp	1.508725
A_68_P09135433	Spnb2	1.508485
A_68_P08196421	Myrip	1.508354
A_68_P05978276	Chst8	1.508244
A_68_P01335447	Tmem15	1.508123
A_68_P02663973	Slc7a11	1.508055

A_68_P06070085	Nr2f2	1.508014
A_68_P06737990	Tm6sf2	1.507957
A_68_P03223240	Efhd2	1.507832
A_68_P13337800	Slc37a1	1.507134
A_68_P05171782	D330017J20Rik	1.506412
A_68_P02609116	Ctbs	1.505324
A_68_P14075007	2810433K01Rik	1.504948
A_68_P08706942	Dazap1	1.504895
A_68_P02569272	Tmem144	1.504859
A_68_P05270630	Tada3l	1.504787
A_68_P13560216	Tmem63b	1.504767
A_68_P02661989	3110045G13Rik	1.504635
A_68_P00183437	Hsd11b1	1.504583
A_68_P09561712	Tbrg4	1.504062
A_68_P06842888	Nqo1	1.50378
A_68_P12822563	Glis2	1.503718
A_68_P12410230	Tmprss6	1.50269
A_68_P09111113	Stk10	1.50266
A_68_P06239470	4632411J06Rik	1.502557
A_68_P09709914	Igfbp1	1.502249
A_68_P13194682	Cfb	1.502245
A_68_P01214281	Nmt2	1.501857
A_68_P04623587	Actb	1.501291
A_68_P04200611	Abcb4	1.501289
A_68_P01019744	Cdc73	1.500788
A_68_P00777015	Tcfap2b	1.500526
A_68_P08293317	Pah	1.500474
A_68_P03116256	Fdps	1.500366
A_68_P06515895	Klk7	1.500346
A_68_P03580331	Btf3l4	1.500274
A_68_P06236098	Numa1	1.50005
A_68_P14154160	Ina	1.499852
A_68_P01581394	Msrb2	1.499851
A_68_P02538277	2810046L04Rik	1.499431
A_68_P03701939	Ube4b	1.49933

A_68_P01773274	Spo11	1.498968
A_68_P08569765	Mdm1	1.498929
A_68_P13847539	Pcdhga9	1.498679
A_68_P05115327	Lmo3	1.498207
A_68_P04548189	Mvk	1.497773
A_68_P03303259	Errfi1	1.497325
A_68_P04730242	Otop1	1.497269
A_68_P06987322	Vps4a	1.496804
A_68_P00072272	Ptpn14	1.496656
A_68_P05425988	Uroc1	1.496605
A_68_P04850699	Slc24a6	1.496255
A_68_P03848066	A330049M08Rik	1.49583
A_68_P04166134	Rheb	1.495697
A_68_P12045626	Tenc1	1.495185
A_68_P04199401	Gusb	1.494713
A_68_P02095165	A930034L06Rik	1.494544
A_68_P03285893	Tnfrsf1b	1.494452
A_68_P04567641	A230097K15Rik	1.49408
A_68_P14926982	Klhl13	1.493792
A_68_P06280706	Tmem126a	1.493113
A_68_P01643192	Psd4	1.493083
A_68_P13069277	Zfp161	1.492737
A_68_P03821972	Lrrn6c	1.492345
A_68_P01833934	Pax6	1.492224
A_68_P13786129	Zfp521	1.492141
A_68_P08713341	Apof	1.492023
A_68_P08592121	BC067068	1.491903
A_68_P05489661	Plxnd1	1.491591
A_68_P04730667	Ddx55	1.491466
A_68_P12461401	Sfrs2ip	1.491209
A_68_P09032850	Gja12	1.490339
A_68_P08883504	Nhsl1	1.490069
A_68_P04650751	Ppargc1a	1.489565
A_68_P04203052	Oas3	1.489494
A_68_P12264171	Smug1	1.489468

A_68_P03328786	Ube2j2	1.488864
A_68_P13251990	Srrm2	1.488836
A_68_P07908749	Coro2b	1.488818
A_68_P00615596	Mcm3	1.488505
A_68_P01935105	Trp53inp2	1.488119
A_68_P03363098	Map3k6	1.487913
A_68_P03279715	Tnfrsf14	1.487573
A_68_P02931199	Tspan5	1.487125
A_68_P08264606	Upb1	1.487112
A_68_P04453201	Gpr81	1.487078
A_68_P10974141	Ibrdc2	1.487075
A_68_P05193196	Bhlhb2	1.486407
A_68_P06027163	Furin	1.485408
A_68_P05684309	1110061O04Rik	1.485196
A_68_P01092822	Fmo1	1.485161
A_68_P08398846	BC030440	1.484813
A_68_P02293895	Wdr3	1.484784
A_68_P04403724	En2	1.484733
A_68_P12384339	Faim2	1.48465
A_68_P12452838	Pim3	1.484413
A_68_P04237158	Slc12a9	1.484049
A_68_P13883460	Pcdha9	1.483592
A_68_P13221182	2300002M23Rik	1.483365
A_68_P10200952	Tnfaip2	1.483083
A_68_P04827265	6530401C20Rik	1.482908
A_68_P13379850	Gfer	1.482626
A_68_P10378975	Otub2	1.482241
A_68_P14239859	Dmrt2	1.481924
A_68_P12884206	Dppa4	1.481689
A_68_P03968747	Melk	1.481554
A_68_P08208323	Tsfm	1.48144
A_68_P04203052	Oas3	1.481087
A_68_P06465255	Egln2	1.480924
A_68_P11860531	BC004728	1.480485
A_68_P03773673	Tlr12	1.480365

A_68_P11904302	Mapk15	1.480307
A_68_P11197285	Peli2	1.480283
A_68_P04200611	Abcb4	1.480071
A_68_P09611503	Doc2b	1.480063
A_68_P14380191	Tmem20	1.480031
A_68_P05265653	Aqp1	1.479679
A_68_P01810918	Ascc3l1	1.479564
A_68_P07758524	Nt5e	1.478965
A_68_P04664680	Cldn3	1.478748
A_68_P03943090	E130308A19Rik	1.478708
A_68_P03435110	Slc45a1	1.478466
A_68_P03736449	Galt	1.477605
A_68_P13753170	Mc5r	1.477093
A_68_P07480474	Pthr1	1.476385
A_68_P08538263	Gnptab	1.476382
A_68_P00952031	4930511H11Rik	1.476252
A_68_P04333196	1110008J03Rik	1.476219
A_68_P05259670	EG232599	1.475688
A_68_P14475320	Avpi1	1.47559
A_68_P03581858	St3gal3	1.47554
A_68_P14380196	Tmem20	1.475082
A_68_P09050344	Irgm	1.474905
A_68_P05321910	Kcnd2	1.474903
A_68_P05332408	Wnt7a	1.474868
A_68_P09959320	Hbp1	1.474518
A_68_P10346997	BC022687	1.474234
A_68_P14467081	1810009A15Rik	1.474176
A_68_P12269379	Slc1a3	1.473899
A_68_P02205490	Epb4.1l1	1.473807
A_68_P09109051	Sat2	1.473677
A_68_P05357104	Podxl2	1.473383
A_68_P03611411	9530048O09Rik-4833401D15Rik	1.473271
A_68_P03205122	Tmem57	1.473181
A_68_P06458357	Sphk2	1.472909
A_68_P00015050	Tmcc2	1.472899

A_68_P10442918	Hist1h4c	1.472336
A_68_P14357229	Stx5a	1.47231
A_68_P14180333	Ppapdc2	1.472111
A_68_P02139635	Cry2	1.472067
A_68_P14420911	Slc1a1	1.472054
A_68_P10322597	Itpk1	1.471877
A_68_P04908551	Pptc7	1.471804
A_68_P15175279	Tcfe3	1.471577
A_68_P10611060	Gadd45g	1.470455
A_68_P02981059	Anxa9-Lass2	1.470382
A_68_P01650076	Slc23a2	1.470248
A_68_P03589975	Npal3	1.470064
A_68_P08817697	Sgk	1.470035
A_68_P07723707	Csk	1.47002
A_68_P10664399	Gpbp1	1.469917
A_68_P08706306	Lrig3	1.469894
A_68_P03512349	C1qa	1.469851
A_68_P12896952	A_68_P12896952	1.469748
A_68_P03974014	Gja4	1.469338
A_68_P08310252	Arid5b	1.469075
A_68_P11769464	E430028B21Rik	1.467519
A_68_P04328380	Hnrpd	1.467386
A_68_P06666207	Cd37	1.466997
A_68_P09413365	Og9x	1.466959
A_68_P05085436	5730596B20Rik	1.466053
A_68_P08464845	ORF61	1.466034
A_68_P04435271	Man2b2	1.465602
A_68_P07517444	Apoa1	1.46503
A_68_P09211126	Serpinf1	1.464924
A_68_P13439820	Ptprs	1.464498
A_68_P03042236	Rorc	1.464455
A_68_P01238242	Pacsin3	1.464309
A_68_P00619216	Nek2	1.464303
A_68_P09355175	Sirt7	1.464004
A_68_P03176909	6530418L21Rik	1.463419

A_68_P04587149	Dtx2	1.463352
A_68_P13286081	Gnmt	1.463125
A_68_P12412293	Irak4	1.462879
A_68_P11625843	Kcnma1	1.46168
A_68_P09105482	Kcnip1	1.461467
A_68_P10412744	Esrrb	1.461455
A_68_P05623990	Trh	1.461438
A_68_P07370542	1700067K01Rik	1.461293
A_68_P07571962	Smad6	1.461241
A_68_P09553977	Wipi1	1.460796
A_68_P07304573	BC048644	1.460769
A_68_P10735916	Hmgcr	1.460451
A_68_P05734059	Uroc1	1.459961
A_68_P12151407	2010109I03Rik	1.459711
A_68_P04338789	Oas2	1.459507
A_68_P14301707	Rcor2	1.459389
A_68_P14265560	Sema4g	1.459317
A_68_P09651374	Actg1	1.459115
A_68_P11746907	Zfp503	1.459074
A_68_P03226841	Pla2g2c	1.459
A_68_P07401393	Unc13a-Jak3	1.458882
A_68_P07237261	Tubgcp3	1.458152
A_68_P09413609	Tk1	1.45776
A_68_P08461195	Ccdc53	1.457504
A_68_P11855104	5730410E15Rik	1.456762
A_68_P07666214	BC043934	1.456695
A_68_P06327205	Kcnc3	1.456661
A_68_P04271916	Idua	1.456311
A_68_P08465588	Prdm1	1.456099
A_68_P08645665	Dot1l	1.455854
A_68_P12286824	Prpf40b	1.455795
A_68_P04055134	Clspn	1.45562
A_68_P12955390	Hes1	1.45488
A_68_P05271973	Vhlh	1.45486
A_68_P13130716	Cacna1h	1.454817

A_68_P03853104	Tinagl	1.454207
A_68_P06519325	Fes	1.453941
A_68_P11545530	EG328479	1.45386
A_68_P13359207	Lrpprc	1.453509
A_68_P08470122	Lrrc10	1.453151
A_68_P08878746	Myf5	1.452546
A_68_P11966471	Bik	1.452381
A_68_P07060963	Zdhhc7	1.4523
A_68_P11345930	ENSMUST00000083663.1:-4472	1.450971
A_68_P03634588	Gjb5	1.45094
A_68_P09006057	Hoxb4	1.450782
A_68_P11941919	EG432987	1.450437
A_68_P05445839	Shfm1	1.450418
A_68_P03786862	Tmem64	1.450056
A_68_P10307843	Smek1	1.44999
A_68_P11347888	Bnip3l	1.449971
A_68_P01681120	Hars2	1.449803
A_68_P13671847	Pcdhgb8	1.449786
A_68_P13446694	A_68_P13446694	1.449601
A_68_P04012043	C1qc	1.449499
A_68_P08866073	Sycp3	1.449455
A_68_P13271863	Gtf2h4	1.448877
A_68_P07350102	Mbtps1	1.44832
A_68_P08817485	D10Jhu81e	1.448223
A_68_P05358487	Rassf8	1.448168
A_68_P08146769	Tbx18	1.447889
A_68_P10702699	Slc35d2	1.447859
A_68_P07188476	St3gal2	1.447195
A_68_P07561953	Aph1c	1.447054
A_68_P13146687	A_68_P13146687	1.44675
A_68_P13897391	Trim36	1.446738
A_68_P09172542	Myo1c	1.446464
A_68_P10205338	Yy1	1.446294
A_68_P02983875	6330549D23Rik	1.446224
A_68_P11173859	Fgf17	1.445486

A_68_P01397647	5730407K14Rik	1.445279
A_68_P13889057	Pqlc1	1.445208
A_68_P09613151	Eif4a3	1.44508
A_68_P01220646	4833422F24Rik	1.44491
A_68_P14071655	Lipg	1.444906
A_68_P08135755	Tbx20	1.444635
A_68_P03525379	Dnajc6	1.444565
A_68_P05193381	Phf14	1.444277
A_68_P04047544	Icmt	1.444191
A_68_P09409829	2310033P09Rik	1.444157
A_68_P04518724	Ubc	1.443886
A_68_P03010171	C030011O14Rik-Usp33	1.443548
A_68_P07234504	Cdh1	1.443441
A_68_P03334646	Npr2	1.44343
A_68_P07324810	Hsd11b2	1.443287
A_68_P13445330	Tap2	1.443123
A_68_P02983160	Pcdh10	1.443029
A_68_P10880830	BC005537	1.44275
A_68_P05987685	Olfr503	1.442328
A_68_P08204474	Inhbe	1.442297
A_68_P07953884	Cbl	1.442292
A_68_P14380340	Cdk2ap2	1.442253
A_68_P13470840	Snf1lk	1.44215
A_68_P11658283	Kcnk5	1.441586
A_68_P03406414	Ubxd3	1.441534
A_68_P03568831	Gjb3	1.441268
A_68_P09016819	Pcgf2	1.441229
A_68_P09694962	Hn1	1.440619
A_68_P00866288	B3gnt7	1.440501
A_68_P03498845	Id3	1.440415
A_68_P08838102	Rab32	1.440212
A_68_P12199092	4930572J05Rik	1.440077
A_68_P09550803	Tnfsf13	1.440059
A_68_P02301823	Tpd52	1.439817
A_68_P07646992	Als2cl	1.439145

A_68_P04400748	Zcchc8	1.438848
A_68_P04178073	Phox2b	1.438624
A_68_P10960939	Cd83	1.438605
A_68_P08356244	Dusp6	1.438435
A_68_P07631418	Ap1m2	1.438401
A_68_P09497481	Krt23	1.438379
A_68_P14346771	Ms4a7	1.438087
A_68_P07117899	Zfp90	1.437828
A_68_P03205122	Tmem57	1.437755
A_68_P01212050	Tcfap2c	1.437727
A_68_P08300127	Olfr796	1.437219
A_68_P06130973	Tm6sf1	1.43721
A_68_P00729624	Susd4	1.436958
A_68_P11308572	Tgm1	1.436956
A_68_P03766316	Dffb	1.436953
A_68_P06817355	1700067K01Rik	1.436912
A_68_P09694967	Ghdc	1.436808
A_68_P03987198	Dio1	1.436744
A_68_P10151513	Psma6	1.436742
A_68_P15013691	Las1l	1.436736
A_68_P06329715	Tnfrsf19l	1.436433
A_68_P07455275	Plscr4	1.436274
A_68_P07941290	Ttc12	1.436246
A_68_P06418431	Ascl2	1.43618
A_68_P04014066	Mfsd2	1.435986
A_68_P13972523	Fchsd1	1.435936
A_68_P14198543	Tmem132a	1.435892
A_68_P12371670	Atp5g2	1.435802
A_68_P04618587	Fgl2	1.435778
A_68_P09738639	Doc2b	1.434645
A_68_P02824099	Mfn1	1.434416
A_68_P13981763	Mapk4	1.434064
A_68_P12256635	Lifr	1.433977
A_68_P06357663	Zfp536	1.433422
A_68_P11249669	Il17rb	1.433253

A_68_P14078494	Slc25a46	1.433198
A_68_P06865553	Chrnb3	1.433009
A_68_P01157255	Muc15	1.432698
A_68_P00626527	Atf3	1.432621
A_68_P12202170	Grhl2	1.432486
A_68_P12998516	Olig1	1.432448
A_68_P04146264	Txk	1.43237
A_68_P04141689	Srd5a2l	1.432019
A_68_P06481214	Ap2a1	1.431394
A_68_P10478798	Foxf2	1.431098
A_68_P13840590	Mppe1	1.431078
A_68_P08817448	D10Jhu81e	1.431062
A_68_P02863075	Crtc2	1.431041
A_68_P06010301	EG210155	1.430945
A_68_P08979891	Krt42	1.430717
A_68_P04616479	Hsd17b11	1.430139
A_68_P06285193	Pnkp	1.430132
A_68_P11857308	Spatc1	1.430124
A_68_P12916378	Pigz	1.429979
A_68_P02375820	Pdzk1	1.429251
A_68_P12437623	Ndufa6	1.428991
A_68_P13384162	Cd70	1.428769
A_68_P11226200	Hs6st3	1.428643
A_68_P08401341	Rnf126-Fstl3	1.42858
A_68_P01123531	Mreg	1.427345
A_68_P07533203	Snx22	1.426848
A_68_P00181824	Ccdc108	1.42615
A_68_P12713735	Atp5o	1.425642
A_68_P12756270	Pigz	1.425371
A_68_P13449298	Scube3	1.425344
A_68_P07504222	Stt3b	1.425313
A_68_P14355984	Cybasc3	1.425285
A_68_P13339960	Pde9a	1.42442
A_68_P09701026	Tcf2	1.424155
A_68_P04393156	Trafd1-C330023M02Rik	1.423769

A_68_P11522475	6720456H20Rik	1.423216
A_68_P14303909	1700019N12Rik	1.423036
A_68_P09330626	Kcnab3	1.422833
A_68_P02818663	Efna1	1.422755
A_68_P06985598	Pkd1l2	1.422495
A_68_P08976711	Aspscr1	1.422151
A_68_P07836928	Nradd	1.421921
A_68_P09168851	2810408A11Rik-0610025P10Rik	1.421339
A_68_P11069210	Shc3	1.421312
A_68_P08436624	D630029K05Rik	1.421011
A_68_P11561956	Bnip3l	1.420641
A_68_P09565027	D11Ertd636e	1.420617
A_68_P11534471	Lgals3	1.420573
A_68_P03279715	Tnfrsf14	1.420541
A_68_P03753591	Hivep3	1.420448
A_68_P11708828	Rnase10	1.420242
A_68_P05968610	Fxyd1	1.420085
A_68_P08628535	Lgr5	1.419756
A_68_P09109482	Rars	1.419538
A_68_P06706577	Irx5	1.41944
A_68_P15191852	Ogt	1.419377
A_68_P09404111	Gm2a	1.419372
A_68_P05567935	Gcc1	1.419256
A_68_P01275891	Fign	1.419248
A_68_P09937366	Flrt2	1.419085
A_68_P06218982	U2af2	1.418837
A_68_P01301589	Acvr1	1.418826
A_68_P04562142	Hsd17b11	1.418792
A_68_P12239462	Dgat1	1.418616
A_68_P06633817	Gm1082	1.418121
A_68_P08834134	Ddit4	1.417451
A_68_P06548919	Drd4	1.417019
A_68_P01678644	Hck	1.416811
A_68_P00896703	2500001K11Rik	1.416706
A_68_P05437348	Rtkn	1.416545

A_68_P03534308	Bsdc1	1.416436
A_68_P07822223	Nr2e3	1.416241
A_68_P01657359	BC020535	1.41608
A_68_P01910329	Grb14	1.416035
A_68_P12991567	Ppil2	1.4158
A_68_P14960260	Gpc4	1.415751
A_68_P07663357	Megf11	1.415656
A_68_P04093287	Cited4	1.414417
A_68_P06595084	Dpy19l3	1.414311
A_68_P11711513	Rpp14	1.414297
A_68_P05998356	Bnip3	1.414179
A_68_P08888274	Cd63	1.413673
A_68_P10267584	Pik3cg	1.413653
A_68_P00522030	Fev	1.413312
A_68_P07896816	Bnip2	1.413203
A_68_P04976323	Cdk8	1.413009
A_68_P12071211	Trib1	1.412917
A_68_P05568084	Ube2h	1.41275
A_68_P12824258	Adcy9	1.412542
A_68_P05011827	Ppm1k	1.412537
A_68_P05032875	Akr1b8	1.412516
A_68_P03577203	Wnt4	1.412179
A_68_P06251950	Pde2a	1.412121
A_68_P07296135	BC003267	1.412025
A_68_P04225894	Slc10a6	1.41192
A_68_P11792784	Nr1d2	1.411907
A_68_P12701085	Tbx1	1.411715
A_68_P03842194	C1qb	1.411368
A_68_P08611292	Tjp3	1.411262
A_68_P08839310	Snrpd3	1.410557
A_68_P00626532	Atf3	1.409928
A_68_P04668612	Qdpr	1.409669
A_68_P08526195	Gstt2	1.409095
A_68_P12204339	Panx2	1.40859
A_68_P05634158	Hoxa13	1.4084

A_68_P08586867	Myf5	1.407948
A_68_P01482299	Zbp1	1.40785
A_68_P07081044	Fbxw9	1.407796
A_68_P13443354	Lrfn2	1.407457
A_68_P08917740	Usp15	1.407389
A_68_P03818522	Grhpr	1.407176
A_68_P08586858	Myf5	1.406853
A_68_P09645961	OTTMUSG00000000934	1.406851
A_68_P12638874	Tbx1-4930588K23Rik	1.406691
A_68_P11984719	Nr4a1	1.406403
A_68_P05955052	Hnrpl	1.406317
A_68_P08815062	Yeats4	1.406161
A_68_P11899315	BC024139	1.406085
A_68_P09289135	Pipox	1.405882
A_68_P13374868	Rps6ka2	1.405852
A_68_P01897769	F730014I05Rik	1.405616
A_68_P07581953	Foxl2	1.405551
A_68_P07469052	Usp2	1.405524
A_68_P09455243	Rnf185	1.405436
A_68_P14254972	Sh3pxd2a	1.404968
A_68_P01310632	Mett5d1-Kif18a	1.40493
A_68_P04548189	Mvk	1.404736
A_68_P09287099	D130058I21Rik	1.40361
A_68_P12092104	Krt79	1.403355
A_68_P02033295	Ccdc3	1.403106
A_68_P07340578	Uba52	1.403015
A_68_P05599948	C1r	1.402884
A_68_P11235583	Ccdc122	1.402699
A_68_P08777342	Sumo3	1.402462
A_68_P07828967	Ccdc84	1.40236
A_68_P04635161	Pomp	1.4014
A_68_P05653925	Pot1a	1.401393
A_68_P13038294	Klhl22	1.401296
A_68_P09512336	Osbp2	1.401245
A_68_P08033503	BC018242	1.401027

A_68_P10846718	Ntrk2	1.400852
A_68_P00372594	Agxt	1.400527
A_68_P01674991	Sall4	1.400176
A_68_P07876589	Fbxo9-Ick	1.400175
A_68_P06528889	Nr2f2	1.399985
A_68_P13396934	Pspn	1.399967
A_68_P10254185	Egln3	1.39981
A_68_P06116789	Slc6a5	1.399372
A_68_P08826802	Hkdc1	1.399266
A_68_P04013537	Nsmaf	1.399119
A_68_P10982190	Rnf44-BC040758	1.39882
A_68_P12186373	Ly6i	1.398572
A_68_P03889508	Ppap2b	1.398415
A_68_P09929935	AI132487	1.398322
A_68_P07249032	Cdk10	1.398121
A_68_P00557108	Dnajb3-Ugt1a1	1.397994
A_68_P04520897	Wasf3	1.397786
A_68_P06144845	Tm2d3	1.397546
A_68_P02994061	Nudt6	1.397301
A_68_P01251024	Gfra4	1.397196
A_68_P14437284	5730596K20Rik	1.397124
A_68_P01853992	Hoxd4	1.39679
A_68_P03294383	Masp2	1.396656
A_68_P07433896	Ptger1	1.396541
A_68_P04616479	Hsd17b11	1.396504
A_68_P07107255	Adcy7	1.396433
A_68_P09685818	Gal3st1	1.396188
A_68_P03279739	Tnfrsf14	1.396072
A_68_P00471093	Rgs7	1.395771
A_68_P03755148	Ddi2	1.395656
A_68_P06124181	Art5	1.395524
A_68_P04604077	Nkx6-1	1.395485
A_68_P06562602	BC048679	1.395233
A_68_P01334676	9230107O10Rik	1.395156
A_68_P09503250	BC018371	1.394799

A_68_P08402819	Pla2g12b	1.394699
A_68_P03535794	Tal2	1.394502
A_68_P08384244	5330439J01Rik	1.394371
A_68_P09096475	Hexim1	1.394275
A_68_P09441021	D11Wsu99e-D11Wsu47e	1.394258
A_68_P03926366	Lrrc42	1.393687
A_68_P03634588	Gjb5	1.393588
A_68_P06664663	Tex101	1.393323
A_68_P09393624	Myo18a	1.39329
A_68_P06302675	Cdkn1c-Slc22a18	1.39323
A_68_P04809489	Mfsd7	1.393038
A_68_P07881511	Ostb	1.39297
A_68_P04108821	1110039B18Rik	1.392763
A_68_P12293861	Cyp2d26	1.392613
A_68_P11848014	Tob2	1.392516
A_68_P09613181	Coro6	1.392221
A_68_P01910453	C1qtnf4	1.392066
A_68_P07471973	Cdkn2d	1.39206
A_68_P09016291	Myo18a	1.391718
A_68_P07030310	Acta1	1.391687
A_68_P09694966	Hn1	1.391387
A_68_P10675646	Tmem174	1.391176
A_68_P03357445	Rprc1	1.390793
A_68_P03577203	Wnt4	1.390543
A_68_P12126766	Tmprss6	1.390396
A_68_P07498897	Npsr1	1.390158
A_68_P06323698	Fgf21-Fut1	1.389935
A_68_P05543283	Kbtbd8	1.389905
A_68_P11254372	Ephx2	1.389425
A_68_P14092716	Hbegf	1.389316
A_68_P13954256	Malt1	1.389258
A_68_P11746912	Zfp503	1.388145
A_68_P08204480	Inhbe	1.388135
A_68_P05307920	Adipor2	1.388039
A_68_P07448050	Proz	1.387751

A_68_P05279238	Sfxn5	1.387737
A_68_P02234571	Gm691	1.387597
A_68_P01483724	Ptk6	1.387365
A_68_P13869080	Hspa9	1.387173
A_68_P00864420	Lefty1	1.387151
A_68_P10918505	Phactr1	1.386866
A_68_P01863211	Cel	1.386746
A_68_P08328254	Sash1	1.386591
A_68_P01443651	Dph4	1.386206
A_68_P04908551	Pptc7	1.386072
A_68_P09372910	Lif	1.386032
A_68_P03655045	Pik3cd	1.385719
A_68_P11916202	Ttc33	1.385672
A_68_P03452908	Olfr157	1.385543
A_68_P01805516	Tank	1.385473
A_68_P02929745	Efna4	1.385329
A_68_P08924359	Arid5b	1.38483
A_68_P03938391	Pef1	1.38467
A_68_P09062144	Fstl4	1.384531
A_68_P14297183	Hps6	1.384467
A_68_P06519231	4933417E01Rik	1.384303
A_68_P03087427	Pklr	1.384162
A_68_P03460707	1810054D07Rik	1.383547
A_68_P04156716	Tpst2	1.383382
A_68_P13522497	Foxp4	1.383341
A_68_P00719128	Lad1	1.383301
A_68_P01396737	Itgb6	1.383246
A_68_P03974013	Gja4	1.382937
A_68_P13129124	Tcp11	1.382829
A_68_P09651125	2400006H24Rik	1.382334
A_68_P08408708	Nudt4	1.382304
A_68_P09191005	Akap1	1.382135
A_68_P10880009	Fcho2	1.382073
A_68_P01524680	C230093N12Rik	1.382037
A_68_P10016006	Bdkrb1	1.381879

A_68_P05459443	Atoh8	1.381872
A_68_P11389124	Cog3	1.381589
A_68_P15088553	Tmem164	1.381575
A_68_P07536331	Lrrn6a	1.381362
A_68_P01251537	Gata5	1.381279
A_68_P11865490	Rai14	1.381127
A_68_P07869246	Nnmt	1.381111
A_68_P00301978	Ugcgl1	1.381093
A_68_P13358098	Zbtb12	1.380905
A_68_P02686084	Igsf3	1.380833
A_68_P09307716	Doc2b	1.380792
A_68_P02230555	C1qtnf4	1.380743
A_68_P11380312	Itih4	1.380728
A_68_P00783024	Farp2	1.380723
A_68_P03067196	P2ry1	1.380694
A_68_P01846550	Jph2	1.380631
A_68_P01927620	Neurod1	1.380513
A_68_P05970860	Phox2a	1.380406
A_68_P04316025	Arhgap24	1.380341
A_68_P12268481	Aqp2	1.380157
A_68_P03290633	Cdc20	1.379809
A_68_P13445587	Tbcc	1.379756
A_68_P01480955	D930001I22Rik	1.379723
A_68_P12113540	9130401M01Rik	1.379607
A_68_P07707920	Scotin	1.37921
A_68_P00336050	Glul	1.379196
A_68_P01982873	BC066135	1.379019
A_68_P08121432	Rpl14	1.379
A_68_P08195645	Glb1	1.378778
A_68_P01441335	C630035N08Rik	1.378713
A_68_P11964810	Cyp2d22	1.378636
A_68_P08249583	Rbms2	1.37863
A_68_P03753591	Hivep3	1.378012
A_68_P14171474	Prpf19	1.377885
A_68_P12061660	Cacnb3	1.37783

A_68_P11072806	Tgfbi	1.377755
A_68_P02283161	2310010M24Rik	1.377636
A_68_P02814467	Fgb-Plrg1	1.37743
A_68_P07924824	Col7a1	1.376996
A_68_P14318689	Pi4k2a	1.376537
A_68_P03484747	Pax7	1.376464
A_68_P11201342	Efha1	1.376296
A_68_P08847295	Egr2	1.376216
A_68_P01722976	Pck1	1.375718
A_68_P03589975	Npal3	1.375486
A_68_P00145314	B230209C24Rik	1.375474
A_68_P10147980	Dnmt3a	1.375059
A_68_P09328696	Thra	1.374982
A_68_P11327352	Kpna3	1.374889
A_68_P03412051	1700018C11Rik	1.374741
A_68_P09167140	Hs3st3a1	1.374575
A_68_P09313263	Slc16a13	1.374498
A_68_P05795785	Shfm1	1.374492
A_68_P04680665	Ptcd1	1.3741
A_68_P05452018	Asns	1.374082
A_68_P04316294	Mapre3	1.37402
A_68_P00547023	Hsd17b7	1.373833
A_68_P08582890	Slc19a1	1.37352
A_68_P06445706	Rnf121	1.373222
A_68_P14246998	Dkk1	1.373103
A_68_P09344068	Cacnb1-Rpl19	1.373063
A_68_P09850298	Smoc1	1.372897
A_68_P03987040	Arhgef19	1.372813
A_68_P11030208	Ddx4-9130023D20Rik	1.372699
A_68_P00776600	Bcs1l	1.372601
A_68_P03435110	Slc45a1	1.372291
A_68_P08007769	Sidt2	1.372047
A_68_P01524597	Hspa12b	1.372041
A_68_P01433156	Pkp4	1.371939
A_68_P08570664	Tmem26	1.371764

A_68_P13468024	Unkl	1.371743
A_68_P00174516	Chrnd	1.371641
A_68_P05244159	ENSMUST00000083652.1:2772	1.371606
A_68_P05049344	Slc6a6	1.371554
A_68_P02255259	Barhl1	1.370922
A_68_P10354469	Jag2	1.370889
A_68_P05936205	Ifitm5	1.370722
A_68_P06370386	Bnc1	1.370363
A_68_P01091016	Msc	1.370344
A_68_P02192119	Id1	1.370226
A_68_P05058253	Arf5	1.36998
A_68_P07892558	Isl2	1.369729
A_68_P09015240	Naglu	1.369264
A_68_P14499945	Rab1b	1.369186
A_68_P04877098	Slc2a9	1.368716
A_68_P12343989	1810044A24Rik	1.368601
A_68_P08846688	Spryd4	1.3683
A_68_P01348835	Kynu	1.368157
A_68_P14231961	Cnnm2	1.367965
A_68_P13126791	Atp6v1g2	1.367869
A_68_P06061323	Kdelr1	1.367856
A_68_P06480423	Lgals4	1.367703
A_68_P07976988	4733401H18Rik-Dalrd3	1.367257
A_68_P01655862	F730014I05Rik	1.367217
A_68_P07872086	Tmem108	1.366956
A_68_P13172213	Ccnf	1.366942
A_68_P07434767	Insl3	1.366922
A_68_P14478305	Dmrt3	1.366788
A_68_P11209114	Camk2g	1.366663
A_68_P15169058	Bcor	1.366594
A_68_P02180398	Ovol2	1.366446
A_68_P06011169	BC057627	1.366415
A_68_P01505234	Set	1.36641
A_68_P11659210	Fbxl3	1.366291
A_68_P11333407	1810034K20Rik	1.36605

A_68_P06948293	Dctn6	1.365828
A_68_P05893701	6330512M04Rik	1.365626
A_68_P09402805	Lhx1	1.365573
A_68_P14205827	Fen1	1.365296
A_68_P12412503	Cyp2d22	1.365036
A_68_P13159875	Tnfsf14	1.365005
A_68_P09477301	Hoxb5	1.364903
A_68_P02711952	Ccna2	1.364885
A_68_P01735828	Serping1	1.364832
A_68_P05052721	Atoh8	1.364831
A_68_P03890853	Epha8	1.36456
A_68_P09389126	Aurkb	1.364507
A_68_P12320215	Myo10	1.364325
A_68_P08894237	Ftcd	1.364095
A_68_P14429653	Pitx3	1.363858
A_68_P09840571	Hs1bp3	1.363666
A_68_P04412246	Dgkq	1.36329
A_68_P07202004	1200003I07Rik	1.363146
A_68_P14335363	Dpf2	1.363058
A_68_P04406264	Sri	1.363057
A_68_P04441942	4930519G04Rik	1.362966
A_68_P00331311	EG240916	1.362892
A_68_P06124180	Art5	1.362879
A_68_P06602766	Homer2	1.362786
A_68_P10658756	Cartpt	1.3627
A_68_P05522702	9130019P16Rik	1.362239
A_68_P09118933	Pib5pa	1.362119
A_68_P09985893	Mia2	1.361866
A_68_P08643752	Rtn4ip1	1.361839
A_68_P05938143	Klk15	1.361748
A_68_P10418410	Mgat2	1.361533
A_68_P03954968	Eno1	1.361501
A_68_P04945718	Preb-Tcf23	1.361425
A_68_P14229235	Cpn1	1.361424
A_68_P07029190	Sh3rf1	1.361375

A_68_P01345408	Api5	1.361265
A_68_P07005244	Best2	1.361143
A_68_P11868103	Khdrbs3	1.361098
A_68_P04093054	Gm694-Zbtb17	1.360762
A_68_P04618584	Fgl2	1.360719
A_68_P09172983	Sec14l4	1.360609
A_68_P06852818	Cul4a	1.360397
A_68_P00836926	Eif4e2	1.360378
A_68_P08725568	Akap12	1.360367
A_68_P04945718	Preb-Tcf23	1.360191
A_68_P00480042	F13b	1.360155
A_68_P09261609	Hoxb13	1.3598
A_68_P08844667	Shmt2	1.359771
A_68_P04393156	Trafd1-C330023M02Rik	1.359573
A_68_P07019190	Hmox1	1.359116
A_68_P11884951	Cbx7	1.359049
A_68_P14159031	Atad1	1.358331
A_68_P09042930	Sectm1a	1.358075
A_68_P09212221	G6pc	1.357706
A_68_P03419478	1200015A19Rik	1.357664
A_68_P06848303	Slc7a6	1.357514
A_68_P10912255	Abt1	1.357501
A_68_P13547882	Pla2g7	1.35728
A_68_P04141273	Dnajc2	1.357091
A_68_P11720885	Pcdh17	1.356579
A_68_P02766324	Vps45	1.356517
A_68_P11409158	Egr3	1.356206
A_68_P06545205	Herc2	1.355988
A_68_P09683452	Trim47	1.355706
A_68_P13224117	Fkbpl	1.355488
A_68_P04964439	Tmem132d	1.355053
A_68_P10277554	Adssl1	1.354443
A_68_P08848709	Kcnmb4	1.354411
A_68_P14159522	Slit1	1.354406
A_68_P02935668	Zfand1	1.354143

A_68_P08669008	Snrpd3	1.354006
A_68_P09187052	Tbrg4	1.353905
A_68_P06293636	Luzp2	1.353853
A_68_P12069241	Phf5a	1.353812
A_68_P07279929	Bcar1	1.353809
A_68_P06230676	Capns1	1.353677
A_68_P04222873	A_68_P04222873	1.353354
A_68_P00780459	4930418G15Rik	1.353342
A_68_P05316111	Prdm5	1.353247
A_68_P09739941	Hoxb7	1.352059
A_68_P04406264	Sri	1.351855
A_68_P04877098	Slc2a9	1.351658
A_68_P07279933	Bcar1	1.351642
A_68_P04155853	Vdp	1.351424
A_68_P14345739	Vegfb	1.351423
A_68_P09032908	Gja12	1.351397
A_68_P14219536	Klf9	1.351378
A_68_P07618605	Gnai2	1.35125
A_68_P04384102	3732412D22Rik	1.351162
A_68_P01199031	NM_027230:-11579	1.351084
A_68_P01164203	Mcts2	1.350976
A_68_P08160048	Paqr5	1.350869
A_68_P07806205	Col12a1	1.35078
A_68_P03966293	Dmbx1	1.35072
A_68_P08907272	Gng7	1.350645
A_68_P12303298	Hoxc8	1.350252
A_68_P04937975	Ncor2	1.350173
A_68_P11984771	3-Sep	1.350123
A_68_P13294540	Arrdc5-Uhrf1	1.34999
A_68_P13251553	Ergic1	1.349967
A_68_P03755098	Ndufs5	1.349936
A_68_P07179485	Fgfr1	1.349803
A_68_P03296241	Edg2	1.349727
A_68_P10905572	Nrn1	1.34968
A_68_P04510543	Nos3	1.349518

A_68_P06929857	Tpm4	1.349483
A_68_P02390624	Crct1	1.349158
A_68_P07172113	Kcnn1-A230052G05Rik	1.349071
A_68_P09369987	Apoh	1.349015
A_68_P14038094	Sh3tc2	1.348769
A_68_P07807897	Plscr2	1.348715
A_68_P00542050	Npas2	1.348586
A_68_P13388154	Bat5	1.348585
A_68_P12836522	Bdh1	1.348484
A_68_P13747694	Rnuxa	1.348397
A_68_P06035698	Plekhf1	1.348306
A_68_P00711134	Slc45a3	1.348202
A_68_P14849946	Kcnd1	1.347914
A_68_P10564825	Emb	1.34784
A_68_P03318310	Slc6a9	1.34777
A_68_P11517987	Lgi3	1.347758
A_68_P09089981	Tax1bp3	1.347588
A_68_P03452908	Olfr157	1.347536
A_68_P02991577	Sec22b	1.347466
A_68_P08773234	Ikzf4	1.346955
A_68_P05918934	Peg3	1.346499
A_68_P02412661	Cyr61	1.346407
A_68_P12213551	Gpihbp1	1.346302
A_68_P03755813	Nans	1.346246
A_68_P12242254	Syngr1	1.345938
A_68_P06522134	Vps33b	1.345822
A_68_P01498090	Plcg1	1.345814
A_68_P09629443	Kctd2	1.345761
A_68_P04193832	Nudt1	1.345384
A_68_P06983274	Jund1	1.345365
A_68_P03959086	Slc45a1	1.345095
A_68_P10202512	Lin52	1.344946
A_68_P11680943	Entpd4	1.344925
A_68_P04316025	Arhgap24	1.344622
A_68_P09776288	Acot1	1.344496

A_68_P14207949	D930010J01Rik	1.344216
A_68_P10251378	Glrx5	1.344153
A_68_P07939274	4931406C07Rik-Josd3	1.344001
A_68_P04331822	Cdc7	1.343979
A_68_P07688516	2200002K05Rik	1.343834
A_68_P07718457	Topbp1	1.343785
A_68_P09515612	Arl5c	1.34352
A_68_P06504997	Slc5a2	1.343518
A_68_P07887108	Dock6	1.343508
A_68_P00418617	Adora1	1.343472
A_68_P03273020	Srrm1	1.342971
A_68_P14423460	Ehd1	1.34275
A_68_P04629383	Pitpnm2	1.342653
A_68_P09340278	Cd300e	1.342653
A_68_P05255015	Hoxa2	1.342652
A_68_P03954968	Eno1	1.342574
A_68_P09459434	Dus1l	1.342536
A_68_P01813685	Dolpp1	1.342438
A_68_P04836896	A330070K13Rik	1.342419
A_68_P04062108	Rpl11	1.342401
A_68_P13873326	Nfatc1	1.342216
A_68_P03439034	2810432D09Rik	1.342085
A_68_P09758153	Lrrc9	1.342081
A_68_P13436543	Bysl	1.34191
A_68_P08531371	Nfic	1.341815
A_68_P04944285	Zfand2a	1.341688
A_68_P02567209	Cth	1.341618
A_68_P14961796	Trpc5	1.341591
A_68_P07349274	Rbm13	1.341505
A_68_P09374043	Trim47	1.341361
A_68_P04803354	Arpc1b	1.341301
A_68_P00206509	St8sia4	1.341296
A_68_P12438489	Sfrs2ip	1.341195
A_68_P12930757	Usp25	1.341182
A_68_P00882129	2310038H17Rik	1.341068

A_68_P14109314	Osbpl1a	1.340885
A_68_P02219550	Traf1	1.340575
A_68_P08679914	Scyl2	1.340469
A_68_P04093287	Cited4	1.34041
A_68_P03930447	Cldn19	1.340004
A_68_P10050034	Trmt5	1.339914
A_68_P11924228	L3mbtl2	1.339842
A_68_P12189029	Card10	1.339354
A_68_P04493529	Tbx3	1.339308
A_68_P09512501	Butr1	1.339248
A_68_P00163908	Twist2	1.339236
A_68_P01248802	Lrrc4c	1.339142
A_68_P01823709	Nphp1	1.339022
A_68_P01075327	Nmur1	1.338942
A_68_P05653891	Pde3a	1.338797
A_68_P01396752	Itgb6	1.338786
A_68_P10703955	Bhmt	1.338775
A_68_P15177773	Nsbp1	1.338724
A_68_P11648927	Mettl6	1.338673
A_68_P08773799	Dnajc14	1.338628
A_68_P05150769	Tax1bp1	1.33797
A_68_P08979889	Krt42	1.337941
A_68_P04225894	Slc10a6	1.337922
A_68_P15175273	Tcfe3	1.337766
A_68_P05428176	Slc2a3	1.337702
A_68_P13502362	Lsm2	1.337471
A_68_P09298703	Myo18a	1.337009
A_68_P12097337	Krt73	1.336887
A_68_P05233471	1110001J03Rik	1.336783
A_68_P03360536	2410166I05Rik	1.336782
A_68_P01092733	Lbr	1.336759
A_68_P03307859	Hp1bp3	1.336706
A_68_P00401634	Adora1	1.336581
A_68_P01553757	Mapk8ip1	1.336339
A_68_P10316211	Zfyve26	1.336171

A_68_P09640992	Psmc3ip	1.336165
A_68_P06887604	Hsf4	1.336164
A_68_P06706147	BC021891	1.336058
A_68_P07337104	Gfod2	1.335881
A_68_P06926624	A230052G05Rik	1.335854
A_68_P01342599	2010011I20Rik	1.33585
A_68_P02265690	C230093N12Rik	1.335798
A_68_P09379981	Smarcd2-Tcam1	1.33561
A_68_P14525996	Timm8a1	1.335288
A_68_P09104644	Aatk	1.335067
A_68_P04735366	Ugt2a3	1.334872
A_68_P04308903	Areg	1.334266
A_68_P04062108	Rpl11	1.334152
A_68_P09582843	Itga3	1.334047
A_68_P09021305	Fmnl1	1.333958
A_68_P14711280	Gpr64	1.333822
A_68_P04162059	Zfp326	1.333766
A_68_P04580659	Mpv17	1.333711
A_68_P14431095	Chuk	1.333707
A_68_P05935174	Cd33	1.3337
A_68_P01869178	Foxa2	1.333583
A_68_P05543279	Kbtbd8	1.333445
A_68_P03533898	Nbl1	1.333352
A_68_P12809678	Tmem50b	1.333178
A_68_P10902235	Pitx1	1.333066
A_68_P04731732	Sdsl	1.332863
A_68_P02825852	Cd1d1	1.332847
A_68_P04275151	Arpc1b	1.332839
A_68_P04844132	Cxcl4	1.33282
A_68_P09880659	Acot1	1.33255
A_68_P14042310	Pcdhga8	1.332507
A_68_P07815979	Gk5	1.332291
A_68_P12751159	B830017H08Rik	1.33221
A_68_P01670540	Rpl35	1.332147
A_68_P04451113	Slc5a1	1.331686

A_68_P10105119	Acot5	1.331665
A_68_P03412051	1700018C11Rik	1.331334
A_68_P07278572	Spfh2	1.331234
A_68_P04597777	Corin	1.331151
A_68_P09378071	Pfn1	1.331113
A_68_P07540560	Aldh1a2	1.331025
A_68_P06373812	Ucp2	1.330931
A_68_P07561548	Clk3	1.33083
A_68_P06653589	Plekha4	1.330529
A_68_P01299923	Hoxd3	1.330252
A_68_P02576267	Gnb4	1.330108
A_68_P09190672	Nr1d1	1.330101
A_68_P08649165	Stk11	1.330069

Supplementary Table 1c: N vs KO 574 hypomethylation genes on d100

Probe Name	Gene Name	Methylation difference value
A_68_P01158774	Rpp38	0.117586
A_68_P04306467	Whsc2	0.44667
A_68_P04768574	Nkx6-1	0.450274
A_68_P04768574	Nkx6-1	0.456722
A_68_P11079538	Sirt5	0.469916
A_68_P05715101	Xpc	0.47726
A_68_P09018865	Cbx4	0.48022
A_68_P02314772	Mtx1-Thbs3	0.48654
A_68_P06626588	6430526N21Rik	0.505216
A_68_P08138715	Mcam	0.512579
A_68_P01143715	Bai3	0.520126
A_68_P14431113	Mxi1	0.521987
A_68_P03651216	Acot11	0.526222
A_68_P04365059	Klb	0.534576
A_68_P07679434	Atp1b3	0.535726
A_68_P05540049	Erc1	0.538101
A_68_P03883271	Scp2	0.544018
A_68_P11132389	Gnl3	0.550753
A_68_P06643826	Gpr4	0.554059
A_68_P09805220	Trib2	0.554567
A_68_P10745919	Ipo11	0.55673
A_68_P14628736	6430550H21Rik	0.556792
A_68_P13302036	Six2	0.55781
A_68_P07912195	Yap1	0.55967
A_68_P02367976	Isg20l2	0.561072
A_68_P13730781	Sema6a	0.56479
A_68_P02919830	Lrrn6d	0.568718
A_68_P12871003	Sema5b	0.577088
A_68_P06843515	2810428I15Rik	0.578968
A_68_P06955360	Ank1	0.578969
A_68_P10309940	Gsc	0.581853
A_68_P01134685	Col19a1	0.583897
A_68_P05939018	A630091E08Rik	0.585316

A_68_P07923222	AI593442	0.586695
A_68_P08005788	Odf3l1	0.589204
A_68_P14157373	Sorcs1	0.589494
A_68_P05596974	Rnf103	0.590833
A_68_P01737808	Srp14	0.600586
A_68_P14240187	Sart1	0.601667
A_68_P08777976	A530089I17Rik	0.602493
A_68_P11820556	Tmem110	0.603203
A_68_P04028116	Cldn19	0.604347
A_68_P11922964	Klf10	0.605503
A_68_P05500620	Stk31	0.60648
A_68_P10176316	Zbtb25	0.61219
A_68_P04104973	Tpte2	0.612323
A_68_P00708979	Pax3	0.612669
A_68_P04742115	Pkd2	0.613064
A_68_P02865901	Mef2d	0.615885
A_68_P07107737	Gse1	0.616863
A_68_P10692125	Mtap1b	0.616997
A_68_P06699548	Pou4f2	0.622794
A_68_P01482084	Idh3b-Ebf4	0.624479
A_68_P11770812	Nefm	0.625311
A_68_P12189296	Letmd1	0.625624
A_68_P10411237	Atxn7l4	0.626317
A_68_P10375909	Six6os1	0.626728
A_68_P09966298	1810011O16Rik	0.626949
A_68_P06843937	Hydin	0.628241
A_68_P03226190	Pla2g2e	0.630122
A_68_P08913495	Hmha1	0.631253
A_68_P06984570	Psmd7	0.631504
A_68_P04104973	Tpte2	0.631847
A_68_P02709474	Foxo1	0.632642
A_68_P01413716	D430039N05Rik	0.635353
A_68_P11738050	Homez	0.639324
A_68_P08104050	Pcsk7	0.640161
A_68_P11559162	Nid2	0.642847

A_68_P12432050	Galnt6	0.643854
A_68_P07078935	Atbf1	0.644039
A_68_P09378399	Ppp1r1b	0.645095
A_68_P11019872	Ptch1	0.645149
A_68_P03226190	Pla2g2e	0.646786
A_68_P08777819	Nfic	0.647609
A_68_P13317398	Arhgdig	0.648079
A_68_P14075007	2810433K01Rik	0.651669
A_68_P09693315	2010305C02Rik	0.65459
A_68_P03139948	Zbtb7b	0.6547
A_68_P06327058	Slc28a1	0.655497
A_68_P05552653	Gabarapl1	0.655712
A_68_P10113786	Bcl11b	0.655849
A_68_P11799087	Lgals3	0.656278
A_68_P04834752	Cutl1	0.65636
A_68_P07640747	Fxyd6	0.657501
A_68_P13232871	Itfg3-Luc7l	0.657704
A_68_P05905001	Ccnd1	0.658338
A_68_P09782980	Kcnf1	0.658418
A_68_P11108606	Ripk1	0.658581
A_68_P13439820	Ptprs	0.65903
A_68_P01431419	Tcfap2c	0.659031
A_68_P01134681	Col19a1	0.660059
A_68_P06922018	Lrrc25	0.662443
A_68_P04137135	Smurf1	0.663187
A_68_P06668394	Igf2	0.664825
A_68_P06631626	Wtip	0.66487
A_68_P11404297	Selk	0.666577
A_68_P04661116	Sema3c	0.667061
A_68_P05301787	LOC380687	0.667457
A_68_P09195114	A830031A19Rik-Bcl11a	0.667459
A_68_P03184759	Gucy1a3	0.668029
A_68_P15095336	Hdac8	0.669755
A_68_P05088298	Hipk2	0.669892
A_68_P09732738	1500005I02Rik	0.67009

A_68_P09498627	Mink1	0.670587
A_68_P12129669	Csad	0.670684
A_68_P06293636	Luzp2	0.670984
A_68_P08666230	Hnrph3	0.671512
A_68_P08504576	Cfd	0.672942
A_68_P03807576	Wdtc1	0.672964
A_68_P03495355	Mib2	0.673332
A_68_P04519541	Gfi1	0.673533
A_68_P05565689	Ptms	0.674708
A_68_P04178083	Phox2b	0.676166
A_68_P11486688	Lect1	0.676721
A_68_P06358071	Jmjd5	0.677043
A_68_P12254359	C730034F03Rik	0.677381
A_68_P06205022	Lhb	0.677382
A_68_P10787030	Nr2f1	0.678045
A_68_P11173846	Fgf17	0.678116
A_68_P07518868	Chrna5	0.678525
A_68_P09122772	Accn1	0.678624
A_68_P04519541	Gfi1	0.678924
A_68_P01804429	Pxmp4-Zfp341	0.678979
A_68_P04629065	Gfi1	0.679573
A_68_P12511902	Sim2	0.679789
A_68_P13620460	LOC639653	0.679809
A_68_P01875370	Kcnb1	0.680068
A_68_P09880778	Rps7	0.680826
A_68_P13786129	Zfp521	0.681316
A_68_P15206339	Sox3	0.681804
A_68_P02205842	Rya3	0.682398
A_68_P04216765	Hip1	0.682562
A_68_P01733029	Rassf2	0.683103
A_68_P13319154	9530058B02Rik	0.683382
A_68_P00719131	Lad1	0.683445
A_68_P05039877	Ccnd2	0.683838
A_68_P03786862	Tmem64	0.684374
A_68_P11936126	Josd1-Gtpbp1	0.684594

A_68_P02165297	Cpxm1-4933425O20Rik	0.684787
A_68_P06476351	Bcam	0.685215
A_68_P10132985	Id2	0.685735
A_68_P06075223	Aplp1	0.686026
A_68_P02046877	Egfl7	0.686442
A_68_P04107140	Dnajb6	0.687186
A_68_P04949699	Otof-1700001C02Rik	0.687455
A_68_P01831728	Ddx31	0.68769
A_68_P04935832	Aff1	0.688011
A_68_P01147199	Lmod1	0.688223
A_68_P01624279	A430105I19Rik-1810007E14Rik	0.688864
A_68_P05448318	Kcna5	0.68927
A_68_P14336883	AU041783	0.689526
A_68_P13555549	Ppm1b	0.689595
A_68_P08112343	C230081A13Rik-Hmg20a	0.690693
A_68_P13789140	Arhgap12	0.69101
A_68_P05969338	Htra1	0.691246
A_68_P02060987	Defb25-Rem1	0.691256
A_68_P02681301	Lhx8	0.692064
A_68_P13976384	Csnk1a1	0.69207
A_68_P11912864	Krt8	0.692364
A_68_P08990544	Cnot8	0.692458
A_68_P14314284	Trim8	0.692729
A_68_P07882801	Cplx3	0.692778
A_68_P00234397	Enah	0.692884
A_68_P03926726	Acot11	0.693338
A_68_P07453625	Ky	0.693542
A_68_P06387117	Hdgfrp3	0.693637
A_68_P02560192	Efna1	0.693787
A_68_P01294402	Tlk1	0.693973
A_68_P06739082	2310022B05Rik	0.694111
A_68_P13303514	Zfp101	0.694683
A_68_P02489623	Ankrd34	0.694873
A_68_P11433919	Tpt1	0.695004
A_68_P11927843	Prlr	0.695392

A_68_P09466672	Atp5g1	0.695604
A_68_P06237422	Msx3	0.695632
A_68_P04126741	AA407659	0.696251
A_68_P04730667	Ddx55	0.696903
A_68_P00271539	Accn4	0.697042
A_68_P10325481	Mycn	0.69712
A_68_P04262240	Mapk10	0.697561
A_68_P14260104	Spnb3	0.698115
A_68_P06373804	Ucp2	0.698182
A_68_P12204339	Panx2	0.69846
A_68_P07355188	Cdh11	0.698509
A_68_P08435891	Edg6	0.699009
A_68_P15144551	Sat1	0.699452
A_68_P09535003	B4galnt2	0.700355
A_68_P13398221	Tnrc5	0.700688
A_68_P03863568	Il11ra1	0.700935
A_68_P15022938	Irak1	0.701275
A_68_P03668203	Slc44a1	0.701482
A_68_P07304552	BC048644	0.701887
A_68_P08840096	Lrig3	0.701897
A_68_P07602168	Crabp1	0.701967
A_68_P06410543	Mesp1	0.702275
A_68_P13570124	1700106N22Rik	0.702611
A_68_P14242816	Sorbs1	0.702781
A_68_P13090914	Telo2	0.70373
A_68_P06208597	2900093B09Rik	0.703745
A_68_P04522816	Pdx1	0.703949
A_68_P10018570	Ap4s1	0.704897
A_68_P11319776	Stk24	0.704949
A_68_P00708991	Pax3	0.705136
A_68_P07428129	Irx3	0.705412
A_68_P06515895	Klk7	0.705658
A_68_P07142897	Taf1c	0.705989
A_68_P08773789	1110012D08Rik-Dnajc14	0.706013
A_68_P06922348	Insl3	0.706748

A_68_P13403566	Ccnd3	0.70681
A_68_P05217166	Ephb6	0.706857
A_68_P01797463	A530013C23Rik	0.707271
A_68_P01725854	Ctsa	0.707914
A_68_P12694774	Olig2	0.708003
A_68_P06756488	Zmat4	0.708105
A_68_P13463798	Zbtb22	0.708449
A_68_P07897281	Rdx	0.708498
A_68_P12725023	Ap2m1	0.708837
A_68_P09608031	Egfr	0.709227
A_68_P04949699	Otof-1700001C02Rik	0.709397
A_68_P13215738	Mrps18b	0.709609
A_68_P04808481	Ephb4	0.709773
A_68_P07809706	Rbms3	0.710201
A_68_P09536836	Pnmt	0.710279
A_68_P03087438	Pklr	0.710372
A_68_P10243773	Bcl11b	0.710504
A_68_P14224733	Map4k2	0.710717
A_68_P02095165	A930034L06Rik	0.71119
A_68_P12098370	Plec1	0.711219
A_68_P10800279	Pols	0.711318
A_68_P13453584	Prkce	0.711453
A_68_P00484490	Igfbp5	0.71166
A_68_P10400455	Calm1	0.711838
A_68_P08527794	Psap	0.712247
A_68_P00961427	Tada1l	0.712351
A_68_P11292465	Nfatc4	0.712508
A_68_P09846917	Rhob	0.712696
A_68_P06630253	Shkbp1	0.712726
A_68_P04696354	Iqce-AA881470	0.712747
A_68_P13158664	1300003B13Rik	0.713376
A_68_P14426150	Lzts2	0.713412
A_68_P13678065	Nfatc1	0.713504
A_68_P06826869	Pcm1	0.713608
A_68_P01687732	Gatm	0.713882

A_68_P00932178	Epha4	0.713892
A_68_P03738288	Serinc2	0.713937
A_68_P04475202	Caln1	0.713952
A_68_P06550934	Lrrc4b	0.714051
A_68_P10114516	BC048191:656	0.714552
A_68_P08513384	Slc35d3	0.714612
A_68_P01381082	Myt1	0.715598
A_68_P14228699	Eef1g	0.715616
A_68_P06506665	Apoe	0.715624
A_68_P10626473	BC003212:-702	0.715632
A_68_P09162950	Tmc6	0.716272
A_68_P01886185	Stmn3	0.716399
A_68_P01254928	Ntng2	0.71654
A_68_P13240096	Rpl7l1	0.716827
A_68_P10928545	Gcnt2	0.716852
A_68_P13651831	Sil1	0.717036
A_68_P03345996	Cdkn2a	0.7171
A_68_P13131084	Cd2ap	0.717138
A_68_P14301707	Rcor2	0.717149
A_68_P06506007	Gys1	0.717228
A_68_P00702326	ENSMUST00000083599.1:-2617	0.717343
A_68_P06515293	Opa3	0.717569
A_68_P07295590	5830457O10Rik	0.717591
A_68_P12730511	Senp5	0.717678
A_68_P12052833	AW549877	0.717799
A_68_P06948296	Dctn6	0.717894
A_68_P11280485	Jph4	0.717962
A_68_P13917502	Ccdc100	0.718234
A_68_P12963407	Igsf4d	0.718612
A_68_P03926726	Acot11	0.719132
A_68_P03581099	5430416O09Rik	0.719254
A_68_P09350088	Pmp22	0.719328
A_68_P04926196	Fzd10	0.719438
A_68_P00400062	Kif1a	0.71952
A_68_P04967489	Med28	0.719628

A_68_P02835248	Celsr2	0.719747
A_68_P02622581	Chrnb2	0.719778
A_68_P08575022	Col13a1	0.719844
A_68_P02899649	Adamtsl4	0.720331
A_68_P01515364	ENSMUST00000083577.1:-5512	0.720383
A_68_P07570037	Kirrel3	0.720504
A_68_P14495624	Nkx2-3	0.721408
A_68_P14218567	Aldh18a1	0.721656
A_68_P07912188	Yap1	0.72185
A_68_P01782726	Evx2-Hoxd13	0.721966
A_68_P11569601	Ebf2	0.722002
A_68_P06685279	Gpr124	0.722235
A_68_P01966322	C430004E15Rik	0.722636
A_68_P07646992	Als2cl	0.722738
A_68_P07228622	Cotl1	0.722757
A_68_P10408377	Actr10	0.723072
A_68_P10949115	Fst	0.723107
A_68_P06764028	Spg7	0.723217
A_68_P09370370	Cbx8	0.723395
A_68_P09455667	Clk4	0.723549
A_68_P14425233	Ldb1	0.723554
A_68_P05840537	Tra2a	0.723616
A_68_P10021274	Fos	0.723796
A_68_P04606333	A930017N06Rik	0.723866
A_68_P06958349	Wrn	0.724148
A_68_P09015003	A230051G13Rik	0.724167
A_68_P06412434	Bnc1	0.724338
A_68_P09703855	Epn3	0.72453
A_68_P12129893	Cdc42ep1	0.724796
A_68_P07935467	Bsn	0.724924
A_68_P00536905	Btg2	0.725066
A_68_P09861753	Degs2	0.725268
A_68_P08033503	BC018242	0.725339
A_68_P04199774	Kctd10	0.72534
A_68_P04114789	Add1	0.725696

A_68_P13256078	Tbc1d22b	0.725803
A_68_P11576401	Wnt5a	0.725829
A_68_P06274233	Gdpd5	0.725896
A_68_P13431017	4930546H06Rik	0.726111
A_68_P07561476	2310005P05Rik	0.726183
A_68_P12730433	Igf2bp2	0.726511
A_68_P13267090	Hsp90ab1	0.726632
A_68_P09642978	Stx8	0.726649
A_68_P05822242	9630033F20Rik	0.726654
A_68_P08525614	Phlda1	0.72719
A_68_P05127678	Epha1	0.727376
A_68_P05985938	Rgs10	0.727446
A_68_P10642062	Lrrc16	0.727983
A_68_P02037117	Itga8	0.728134
A_68_P09723695	BC018371	0.728156
A_68_P01427925	Gata3	0.728227
A_68_P12310778	Krt18	0.72827
A_68_P14693838	Pcyt1b	0.728336
A_68_P08200511	Hmga2	0.728405
A_68_P04263889	Emilin1	0.728439
A_68_P05906531	2010208K18Rik	0.728507
A_68_P08288180	Tmtc2	0.728552
A_68_P01275047	Cdh22	0.728638
A_68_P07860043	Bace1	0.728797
A_68_P07231075	Tomm20	0.728863
A_68_P10480061	Foxf2	0.728968
A_68_P09415895	D11Bwg0434e	0.729111
A_68_P06098566	Rabac1	0.729684
A_68_P09306619	Metrnl	0.729713
A_68_P05461859	Hoxa9	0.729962
A_68_P09457623	Ddc8	0.730038
A_68_P13186454	Kcnk12	0.730298
A_68_P11227662	Cab39l	0.730309
A_68_P07646962	Als2cl	0.730384
A_68_P05823725	Slc6a6	0.730605

A_68_P06437968	Pnkp	0.730685
A_68_P11625862	Kcnma1	0.7307
A_68_P02754662	Mab21l2	0.730731
A_68_P01019049	Paqr8	0.73084
A_68_P03801846	Phf13	0.730934
A_68_P09906095	Frmd6	0.730935
A_68_P07862583	Calml4	0.731087
A_68_P06000741	Adck4	0.731108
A_68_P04264000	Mtif3	0.731196
A_68_P11068610	Ccdc127	0.731234
A_68_P01605724	Egfl7	0.731243
A_68_P13250291	Srrm2	0.731286
A_68_P14763789	Esx1	0.731302
A_68_P08133364	E330016A19Rik	0.731448
A_68_P09421796	Nog	0.731484
A_68_P11829712	Gjb2	0.731818
A_68_P06580232	Etfb	0.731825
A_68_P10757654	ENSMUST00000083641.1:-5603	0.73213
A_68_P04266446	Dok7	0.732252
A_68_P08159927	Nope	0.732276
A_68_P13753104	Proc	0.732658
A_68_P07663357	Megf11	0.732672
A_68_P04329254	Cdk2ap1	0.732682
A_68_P03214010	Eno1	0.732725
A_68_P07578383	Pknox2	0.73296
A_68_P14218841	Nrxn2	0.732987
A_68_P14809906	Ldoc1	0.733005
A_68_P14938222	Cited1	0.733277
A_68_P05933183	6330512M04Rik	0.733293
A_68_P03632444	2810405K02Rik	0.733335
A_68_P04359736	Epha5	0.733712
A_68_P04032944	Wasf2	0.733713
A_68_P03938697	Spen-B330016D10Rik	0.733786
A_68_P12766510	Hmgn1	0.733819
A_68_P10222137	Coch	0.733984

A_68_P00310817	Klf7	0.734238
A_68_P02567272	Ptbp2	0.734407
A_68_P08612220	Lrp1	0.734423
A_68_P00556271	Ctdsp1	0.734455
A_68_P01139487	Wnt6	0.73457
A_68_P01081579	Nmnat2	0.734572
A_68_P05313100	A_68_P05313100	0.734574
A_68_P02071843	Ifih1	0.734603
A_68_P04671528	Slc10a4	0.734638
A_68_P09467824	OTTMUSG00000003802	0.734834
A_68_P03167353	Ift80-Smc4	0.734841
A_68_P07836186	Yipf2	0.735095
A_68_P00029318	Irs1	0.735273
A_68_P03385664	Btf3l4-Txndc12	0.735303
A_68_P05287542	A_68_P05287542	0.735512
A_68_P07323106	Hapln4	0.735662
A_68_P02612525	Hdgf	0.735723
A_68_P13331861	Tgif	0.735743
A_68_P01021262	Fmn2	0.735776
A_68_P13074929	Psmb8	0.735827
A_68_P09345179	BB220380	0.735948
A_68_P05547796	Lrrc23	0.736307
A_68_P07842392	Paqr5	0.736478
A_68_P12599259	Etv5	0.736701
A_68_P00289278	Usp21	0.736871
A_68_P02294728	Lef1	0.736978
A_68_P12368109	Eppk1	0.737154
A_68_P01190215	Orc4l	0.73737
A_68_P11843718	Cbx6	0.737411
A_68_P08676565	9030224M15Rik	0.737513
A_68_P07467098	Cbl	0.737528
A_68_P09049316	Mrpl45	0.737562
A_68_P03127711	Sox2	0.737656
A_68_P04475206	Caln1	0.737808
A_68_P02349175	Dcamkl1	0.737811

A_68_P01887600	Shc4	0.737862
A_68_P11915779	Foxred2	0.737915
A_68_P11564468	9430077A04Rik-Prrxl1	0.738024
A_68_P12476467	Pigp	0.738032
A_68_P01767792	Tshz2	0.738188
A_68_P06895632	Ces1	0.738199
A_68_P02243584	Agpat7	0.738371
A_68_P09691900	Unc119	0.738813
A_68_P06736693	Csmd1	0.738884
A_68_P09697830	Sec14l4	0.73889
A_68_P02531278	Muc1	0.738954
A_68_P04182410	2810006K23Rik	0.739007
A_68_P08223563	Sbno2	0.73914
A_68_P07820492	Gsta2	0.739193
A_68_P04904123	Pdgfa	0.739559
A_68_P09021937	Rtn4	0.739617
A_68_P14505402	Asrgl1	0.73971
A_68_P05328185	Chd4	0.739797
A_68_P09853995	Tmem121	0.739967
A_68_P12835494	Smpd4	0.740147
A_68_P04730667	Ddx55	0.740272
A_68_P00344176	Sox17	0.740281
A_68_P12185368	Basp1	0.740289
A_68_P14935324	EG331392	0.740425
A_68_P05419197	Usp5-Cdca3	0.740584
A_68_P09683594	Cdc42ep4	0.740598
A_68_P13594311	Lipg	0.74084
A_68_P14444515	Lrfn4	0.740894
A_68_P08446316	Timp3	0.741042
A_68_P01421703	Tgif2	0.741047
A_68_P13916985	Gm672	0.741355
A_68_P07392546	C330011M18Rik	0.741362
A_68_P14344143	Wnt8b	0.741376
A_68_P07839049	Onecut1	0.741434
A_68_P01470919	Gm691	0.741559

A_68_P09233273	Acadvl-Dlgh4	0.741567
A_68_P00202781	Dars2	0.741642
A_68_P04245232	Kcnh2	0.741816
A_68_P05308590	Cav1	0.74192
A_68_P06193351	Dmpk	0.741963
A_68_P04886874	Fzd1	0.741973
A_68_P04233932	Cnot6l	0.742018
A_68_P10675635	Tmem174	0.74208
A_68_P04903904	BC019731	0.742136
A_68_P04070290	Dhcr24	0.742143
A_68_P07313708	Csmd1	0.742166
A_68_P06728417	Cyld	0.742198
A_68_P00694738	Aff3	0.742201
A_68_P09209213	Ubtf	0.742228
A_68_P06654006	Ifitm2	0.742233
A_68_P05954805	Tmem160	0.742255
A_68_P14501274	Syvn1	0.742268
A_68_P09638188	Rad51l3	0.742278
A_68_P10809479	Ndufs4	0.742407
A_68_P09176378	1810073N04Rik	0.742606
A_68_P10088239	Pum2	0.742624
A_68_P03612259	Dnajc8	0.742654
A_68_P10456926	Hist1h1b	0.742853
A_68_P14324730	Cpt1a	0.742853
A_68_P07596637	Vsig2	0.743085
A_68_P03952273	Gpr3-Cd164l2	0.743138
A_68_P08951831	Cct2	0.743156
A_68_P09143801	Pps	0.743194
A_68_P12277588	Hoxc9	0.743237
A_68_P02451509	2010016I18Rik	0.743456
A_68_P02653832	BC028528-Aph1a	0.743739
A_68_P08253385	ORF61-Cnn2	0.743837
A_68_P07433896	Ptger1	0.743898
A_68_P13070859	S3-12	0.744065
A_68_P04414063	Cdx2	0.744135

A_68_P06463897	Foxa3	0.744137
A_68_P01503212	Gm691	0.744166
A_68_P04793013	2700038N03Rik	0.744214
A_68_P08122742	Smad3	0.744219
A_68_P04178083	Phox2b	0.744279
A_68_P12332349	C1qtnf6	0.744305
A_68_P12092825	Sstr3	0.744308
A_68_P01120361	Igsf8	0.744486
A_68_P11766248	Cadps	0.744537
A_68_P14418019	Eef1g	0.744649
A_68_P08221853	Slc1a6	0.744795
A_68_P06261649	Lrrk1	0.744864
A_68_P07411833	Mmp15	0.744877
A_68_P03164989	Alx3	0.744943
A_68_P13522497	Foxp4	0.744954
A_68_P07398504	Sfrp1	0.745021
A_68_P13205279	Cyp1b1	0.745085
A_68_P04159074	3110082I17Rik	0.745143
A_68_P09050152	Ace	0.745194
A_68_P11213391	Kctd6	0.745237
A_68_P06201940	Usp47	0.745579
A_68_P13117095	Bak1	0.745621
A_68_P02040073	Tcp11l1	0.746083
A_68_P04802046	Drd5	0.74617
A_68_P10852234	Tcfap2a	0.746173
A_68_P12397525	Top1mt	0.746175
A_68_P03832008	Pax5	0.746237
A_68_P08285737	Frs2	0.74626
A_68_P05936540	Atp1a3	0.746262
A_68_P06280706	Tmem126a	0.746266
A_68_P03506024	Sdc3	0.746499
A_68_P09611840	Tbx2	0.746576
A_68_P04337955	Rnf4	0.746695
A_68_P09036672	Plekhh3	0.746831
A_68_P08295564	4930404N11Rik	0.746885

A_68_P08756474	Dyrk2	0.746929
A_68_P06099522	Apoc1	0.747018
A_68_P07138762	Znrf1	0.747312
A_68_P09842319	Adssl1	0.747433
A_68_P07915233	Trip4	0.747483
A_68_P03801846	Phf13	0.747541
A_68_P09804391	Siva1	0.747647
A_68_P13125889	Akap8	0.747744
A_68_P02870026	2310007A19Rik-Tnrc4	0.74791
A_68_P08634266	Sf3a2	0.74793
A_68_P04205923	Spink2	0.747965
A_68_P05289468	Bcat1	0.748068
A_68_P10960928	Cd83	0.748074
A_68_P03954981	Eno1	0.748094
A_68_P08112656	Pafah1b2	0.748115
A_68_P13076456	Smoc2	0.748152
A_68_P08689401	Gpr6	0.748254
A_68_P09146494	Cnot6	0.74843
A_68_P09882292	Jundm2	0.748603
A_68_P01669852	Fpgs	0.748647
A_68_P08949156	Rassf3	0.748698
A_68_P14336503	Arhgap19	0.748776
A_68_P03984671	Lrp8	0.748798
A_68_P11454291	Dpysl2	0.748831
A_68_P03859452	Slc31a1	0.748991
A_68_P07587140	Thy1	0.749044
A_68_P02399008	4933421E11Rik	0.749187
A_68_P11623670	Gata4	0.749408
A_68_P03223989	Epha10	0.749467
A_68_P06771759	Snapc2	0.74955
A_68_P14270093	D030056L22Rik	0.749799
A_68_P07504222	Stt3b	0.749927
A_68_P10045379	Nfkbia	0.750031
A_68_P05906990	Snx26	0.750251
A_68_P08303985	Olig3	0.750339

A_68_P12225777	Tnfrsf13c	0.750418
A_68_P13139253	Rxrb	0.750468
A_68_P07335325	Fhod1	0.750607
A_68_P12081602	Rbm9	0.750625
A_68_P10262250	Cyp46a1	0.750654
A_68_P08668997	Snrpd3	0.750895
A_68_P08306365	Gli1	0.750942
A_68_P04533661	Rhof	0.750957
A_68_P08245924	Pias4-Eef2	0.751015
A_68_P09657177	Otx1	0.751015
A_68_P08061931	Lbxcor1	0.751092
A_68_P07673174	Aplp2	0.751115
A_68_P02885891	Edg1	0.751515
A_68_P01704130	C1ql3	0.751609
A_68_P02216243	Grem1	0.751653
A_68_P12464812	Pim3	0.751758

Supplementary Table 1d: N vs KO 2330 hypermethylation genes on d100

Probe Name	Gene Name	Methylation difference value
A_68_P04228571	Barhl2	3.337522
A_68_P14372856	1700019N19Rik	3.145096
A_68_P04403726	En2	3.115225
A_68_P05982951	Tnnt3	2.979039
A_68_P06457634	Adam12	2.937375
A_68_P02004720	Rab22a	2.851541
A_68_P01573803	Srxn1	2.82118
A_68_P04449097	Suds3	2.814325
A_68_P13225573	2310039H08Rik	2.808632
A_68_P11526170	Sox7	2.769721
A_68_P10379467	Slc25a21	2.753301
A_68_P05207111	Rerg	2.731487
A_68_P08382720	Msrb3	2.71797
A_68_P05804478	D6Wsu116e	2.706824
A_68_P10147998	Ppp2r5c	2.705301
A_68_P00332108	Prrx1	2.700878
A_68_P01675194	Dlx1	2.692303
A_68_P04613872	Fscn1	2.686821
A_68_P09701217	Grn	2.681831
A_68_P13219901	Cpne5	2.681237
A_68_P08644962	Pcmt1-Nup43	2.680099
A_68_P09663278	Rpl23a-Rab34	2.677353
A_68_P01110961	Nek2	2.676828
A_68_P08062567	Ei24	2.675238
A_68_P07794298	Qars	2.64844
A_68_P06307353	Tial1	2.643263
A_68_P06099687	Med25	2.635524
A_68_P05011836	Ppm1k	2.626684
A_68_P03496087	Rcc1	2.62644
A_68_P02385105	Ssr3	2.623533
A_68_P04449097	Suds3	2.617687
A_68_P00214101	Pkp1	2.611459
A_68_P02629065	Slc35a3	2.608849

A_68_P09624201	Poldip2	2.598509
A_68_P04137440	Nmu	2.592398
A_68_P13977860	Pcdhga5	2.58649
A_68_P12092853	Ppp1r1a	2.58383
A_68_P06300520	Bccip	2.582791
A_68_P10049305	Insm2	2.567687
A_68_P03496087	Rcc1	2.565922
A_68_P03493306	Rnf11	2.555736
A_68_P00560687	Lrrn2	2.548238
A_68_P06390013	Nfkbib-Sirt2	2.541628
A_68_P09638187	Rad51l3	2.540066
A_68_P12151832	Wnt10b	2.52726
A_68_P09377206	Nr1d1	2.524575
A_68_P04812922	Ugdh	2.519579
A_68_P07796438	Rexo2	2.517563
A_68_P01878180	Phf19	2.514013
A_68_P09233032	Anxa6	2.51313
A_68_P08803491	Zdhhc17	2.5042
A_68_P01796557	Ass1	2.502683
A_68_P12784817	St3gal6	2.492509
A_68_P10857126	Cdc14b	2.492043
A_68_P04047546	Icmt	2.48036
A_68_P04047546	Icmt	2.479973
A_68_P09138754	Hes7	2.479074
A_68_P01948333	Mertk	2.478022
A_68_P11893417	2410187C16Rik	2.468463
A_68_P12729304	Senp2	2.46389
A_68_P09522954	Nol11	2.457453
A_68_P04337962	Rnf4	2.456921
A_68_P06497762	Tmem86a	2.455624
A_68_P01939828	D2Ertd750e	2.453501
A_68_P13186432	Kcnk12	2.45319
A_68_P04803447	Afp	2.449024
A_68_P05569059	6330503C03Rik	2.431746
A_68_P12181545	Gdnf	2.430888

A_68_P12887691	Prodh	2.430837
A_68_P14292416	Hps6	2.423795
A_68_P04803447	Afp	2.422914
A_68_P08612561	Gnaz	2.41599
A_68_P06656274	Ap2a1	2.410547
A_68_P12264947	BC026404:11009	2.401361
A_68_P08384239	5330439J01Rik	2.395098
A_68_P00064328	Mpzl1	2.38971
A_68_P09189144	Timm22	2.388465
A_68_P04535315	Zfp113	2.385913
A_68_P03457489	Ppcs-Zmynd12	2.377034
A_68_P12383540	Ext1	2.371956
A_68_P05278622	9130019P16Rik	2.369969
A_68_P06438449	Mphosph10-Mcee	2.365081
A_68_P00129081	1110058L19Rik	2.364358
A_68_P01085951	Nsl1	2.362552
A_68_P13484729	Tbc1d24	2.361081
A_68_P04535315	Zfp113	2.358729
A_68_P06854598	Jund1	2.356154
A_68_P10884575	Foxc1	2.350868
A_68_P05734641	Gata2	2.350272
A_68_P06201825	Kndc1	2.349644
A_68_P03327028	Pigv	2.349577
A_68_P13220762	Cdc5l	2.349196
A_68_P04449105	Suds3	2.338022
A_68_P14259431	Kazald1	2.337493
A_68_P05933525	Stx4a	2.334643
A_68_P03382917	Tal2	2.332901
A_68_P02550013	Car2	2.330425
A_68_P09366506	Rpl27	2.32869
A_68_P13112327	Gabbr1	2.32771
A_68_P08363067	Lta4h	2.326955
A_68_P12755135	Thap7	2.322586
A_68_P08649164	Stk11	2.317753
A_68_P14233008	Tle4	2.313067

A_68_P06027267	Hmx3	2.304626
A_68_P06936393	Comp	2.302946
A_68_P09673267	Grb7	2.30176
A_68_P05343553	Pde3a	2.301712
A_68_P15159717	Idh3g-Ssr4	2.297848
A_68_P04083220	Hint2	2.286952
A_68_P03829596	Tmem67	2.28632
A_68_P09322877	Lhx1	2.284423
A_68_P02017175	5430407P10Rik	2.283616
A_68_P09906321	A830093I24Rik	2.280764
A_68_P05005759	Crbn	2.2786
A_68_P13299682	Lrg1	2.277395
A_68_P11040977	Col4a3bp	2.272894
A_68_P13344540	Bnip1	2.267459
A_68_P00346078	Selp	2.267069
A_68_P12330978	D15Wsu169e	2.264887
A_68_P03434990	Hes3-Icmt	2.263801
A_68_P10183217	Lgmn	2.263635
A_68_P00119254	B4galt3	2.259801
A_68_P10418423	Mgat2	2.257265
A_68_P03434990	Hes3-Icmt	2.256579
A_68_P03361653	Clta	2.253339
A_68_P00957880	Eif5b	2.253128
A_68_P12435230	Dnalc4	2.251678
A_68_P11980858	Arid2	2.251619
A_68_P00932143	Epha4	2.249608
A_68_P06889526	Tbc1d9	2.245753
A_68_P02432272	Man1a2	2.245117
A_68_P09621591	Aftph	2.242861
A_68_P10259566	Rgs6	2.24233
A_68_P00830537	Pou2f1	2.239323
A_68_P02335661	Adamtsl4	2.239011
A_68_P11769454	E430028B21Rik	2.238988
A_68_P11367835	Selk	2.237847
A_68_P06458410	A_68_P06458410	2.236842

A_68_P10434170	Kcns3	2.235
A_68_P03513133	Elovl1	2.233824
A_68_P08079164	Ccdc72	2.233702
A_68_P11947922	Npr3	2.225963
A_68_P10113784	Bcl11b	2.221893
A_68_P14918066	Ap1s2	2.221875
A_68_P09069018	Arl5c	2.22117
A_68_P13174301	Dll1	2.22085
A_68_P05948960	AI480556-Rras	2.211045
A_68_P07075012	Dhodh-Pkd1l3	2.210895
A_68_P08715298	Prdm1	2.207515
A_68_P01060395	Jph1	2.202907
A_68_P07074552	Usp38	2.195384
A_68_P13315253	Six3	2.19501
A_68_P09343429	Cldn7	2.193336
A_68_P12373485	Atp5g2	2.191661
A_68_P00721355	En1	2.190564
A_68_P09326313	Hoxb3	2.188061
A_68_P12873765	Zdhhc8	2.185787
A_68_P00545742	Stx6	2.184467
A_68_P05932875	Fgf15	2.183996
A_68_P13125876	Akap8	2.183795
A_68_P12062345	Pla2g6	2.18107
A_68_P06686735	Cyb5b	2.18047
A_68_P00970532	Arl4c	2.179171
A_68_P13191027	Khsrp	2.176522
A_68_P03287486	Rps6	2.175643
A_68_P04030531	Pcsk9	2.172458
A_68_P04908551	Pptc7	2.16907
A_68_P04416495	Maea	2.167954
A_68_P03921654	Trim14	2.167842
A_68_P07185461	Wfdc1	2.167173
A_68_P14706450	Diap2	2.164363
A_68_P09080936	AA536749	2.16321
A_68_P03899398	N28178	2.15895

A_68_P09420324	B3gnt2	2.158856
A_68_P07178939	Zswim4	2.157341
A_68_P02285696	Pbxip1	2.156529
A_68_P05908259	1500019G21Rik	2.155881
A_68_P03647830	Kti12	2.155011
A_68_P13422177	Dusp1	2.15181
A_68_P04999281	5730596B20Rik	2.15035
A_68_P12045930	Sub1	2.149073
A_68_P13494803	Tmem112	2.146009
A_68_P06635585	Itpkc-Adck4	2.141942
A_68_P08111967	Armet	2.141615
A_68_P11721186	D14Ertd449e	2.141272
A_68_P10856492	Barx1	2.140881
A_68_P03327028	Pigv	2.140202
A_68_P07532738	Edg8	2.133499
A_68_P06112468	Efcab4a	2.129958
A_68_P02436068	Ccbl2	2.128974
A_68_P05827685	Hoxa10	2.127219
A_68_P01253809	Nat10	2.126568
A_68_P14255125	D19Bwg1357e	2.126277
A_68_P02778268	Sh3glb1	2.126149
A_68_P06001714	Foxi2	2.121927
A_68_P15000956	Emd	2.119857
A_68_P10653099	6530403A03Rik	2.116992
A_68_P03720734	Mmel1	2.114946
A_68_P11542106	Itih1	2.114866
A_68_P13169647	Fgd2	2.112452
A_68_P09701016	Tcf2	2.11228
A_68_P03963324	Ankrd38	2.112091
A_68_P06970993	5330410G16Rik-Crlf1	2.111277
A_68_P13436852	Brd2	2.106271
A_68_P08318176	Hsp90b1	2.105277
A_68_P06342857	Irgq	2.104575
A_68_P00406171	Sccpdh	2.104113
A_68_P11739198	Neil2	2.103502

A_68_P08099628	Ccdc84	2.102325
A_68_P08049237	4930579K19Rik	2.102201
A_68_P12423002	Lmbrd2	2.095144
A_68_P06343722	Mrpl48	2.094081
A_68_P08725566	Akap12	2.087982
A_68_P06085388	Ccne1	2.087838
A_68_P09666800	Phf15	2.087094
A_68_P12684234	Tmem39a	2.086847
A_68_P11545530	EG328479	2.086218
A_68_P04893781	Snx8	2.084056
A_68_P10927042	Bmp6	2.083662
A_68_P11270335	Dnajc3	2.081282
A_68_P11133279	Zfp219	2.080669
A_68_P09166322	1810009O10Rik	2.078709
A_68_P03513133	Elovl1	2.077287
A_68_P10748794	Ddx46	2.077244
A_68_P03032397	Eif4e	2.077016
A_68_P07674282	Acp5	2.076748
A_68_P01920415	Ada	2.076462
A_68_P11869447	Npal2	2.076099
A_68_P11623667	Gata4	2.076096
A_68_P01251534	Gata5	2.073989
A_68_P11401551	Pxk	2.073976
A_68_P03720734	Mmel1	2.073061
A_68_P06076156	Isoc2b	2.070427
A_68_P04935830	Aff1	2.069399
A_68_P05244766	Atp6v1f	2.068696
A_68_P13732324	Zadh2	2.068615
A_68_P04948730	Cops4	2.067778
A_68_P14299016	2900009I07Rik	2.065889
A_68_P03361653	Clta	2.064157
A_68_P02472958	Man1a2	2.063233
A_68_P00299406	Casp8	2.061679
A_68_P12908674	Magmas	2.057114
A_68_P04978117	Fosl2	2.05677

A_68_P02148754	Gpsm1	2.054103
A_68_P07486005	Acvr2b	2.05376
A_68_P08924328	Arid5b	2.052915
A_68_P06425567	Rpl27a	2.052362
A_68_P10839604	Nkd2	2.051346
A_68_P09752161	Laptm4a	2.051258
A_68_P03864897	Bmp8a	2.048082
A_68_P10419770	Chx10	2.047039
A_68_P03151582	Magi3	2.04672
A_68_P11949838	Arhgap8	2.046082
A_68_P02095922	Edn3	2.045853
A_68_P06168319	Isg20l1	2.041963
A_68_P01984542	1700011J10Rik	2.040171
A_68_P09330841	Cpeb4	2.039201
A_68_P13033674	1190017O12Rik	2.037712
A_68_P02126677	Exdl1	2.03753
A_68_P10170178	Six4	2.036455
A_68_P05099470	Cpne9	2.036064
A_68_P12824261	Adcy9	2.034187
A_68_P04670414	Paics	2.031744
A_68_P04908551	Pptc7	2.028737
A_68_P10134551	Lrfn5	2.028656
A_68_P03944455	Ppap2b	2.028107
A_68_P12814189	A_68_P12814189	2.027692
A_68_P09987054	0610010D24Rik	2.026083
A_68_P01616976	Dido1-2310003C23Rik	2.024599
A_68_P12599271	Etv5	2.022431
A_68_P06832441	Junb	2.02211
A_68_P08068605	Tpm1	2.020664
A_68_P04390199	Ung	2.020266
A_68_P08679914	Scyl2	2.019527
A_68_P13422581	Pigq	2.018175
A_68_P13045306	Dcbld2	2.016941
A_68_P12794264	Umps	2.016794
A_68_P06448706	C330005M16Rik	2.015908

A_68_P07815971	Mtap4	2.012681
A_68_P04952826	Crlz1	2.008026
A_68_P13241937	Nfkbie	2.007871
A_68_P07956999	0610040D20Rik	2.007834
A_68_P11342235	Tpt1	2.005267
A_68_P07721541	Icam1	2.000976
A_68_P08045069	Cacna2d2	2.000386
A_68_P05609111	Bms1l	1.998651
A_68_P12192337	Slc11a2	1.998181
A_68_P10432329	Cpsf2	1.994807
A_68_P05158200	Rad52	1.994582
A_68_P04905346	Fbxo21	1.993357
A_68_P05688755	Eno2	1.990397
A_68_P12355067	BC084730:-1892	1.989841
A_68_P07180176	Cklf	1.989743
A_68_P06026021	Nup98	1.989431
A_68_P13218255	Tcf19-Cchcr1	1.987806
A_68_P03457489	Ppcs-Zmynd12	1.987383
A_68_P03797739	Nrd1	1.987344
A_68_P06607751	Syt17	1.986985
A_68_P04027673	Zfp46	1.985715
A_68_P00880287	Tmem63a	1.985523
A_68_P00696895	Rdh10	1.984061
A_68_P02288477	Sec24b	1.983781
A_68_P05250665	Tera	1.983721
A_68_P03335667	Jak1	1.982117
A_68_P13614340	B4galt6	1.981813
A_68_P04684877	9330182L06Rik	1.980818
A_68_P01173313	Stx16	1.980801
A_68_P14301695	Rcor2	1.978289
A_68_P05822226	9630033F20Rik	1.977234
A_68_P02631941	Nexn	1.97718
A_68_P11937882	BC030396	1.975466
A_68_P06580235	Cldnd2	1.974643
A_68_P00810608	Rnf2	1.97213

A_68_P04560031	Crmp1	1.971663
A_68_P14640431	Mtmr1	1.9704
A_68_P08598543	Cd24a	1.969615
A_68_P05723966	2310016C08Rik	1.969236
A_68_P02117183	Vav2	1.968789
A_68_P07769329	Isl2	1.968524
A_68_P11718247	Tspan14	1.966827
A_68_P09124584	Nxph3	1.966222
A_68_P04389279	Fscn1	1.965866
A_68_P07172050	Has3	1.96488
A_68_P11569544	Ebf2	1.964338
A_68_P12589363	Nfkbiz	1.960452
A_68_P03019531	H2afz	1.96004
A_68_P02478471	Npr1-Ilf2	1.959865
A_68_P07607157	Srpr	1.959407
A_68_P12402447	Spryd3-Igfbp6	1.956744
A_68_P05972807	Rps5	1.956179
A_68_P00990943	F11r	1.956017
A_68_P06727785	Galnt2	1.95572
A_68_P02123485	Manbal	1.955227
A_68_P08711980	Fbxo7	1.954184
A_68_P01906139	Pmpca	1.953636
A_68_P13064207	Cuta	1.953298
A_68_P08930401	Slc39a3	1.952323
A_68_P10523836	5133401N09Rik	1.951918
A_68_P08736561	Atp2b1	1.950118
A_68_P12674816	Dvl3	1.95006
A_68_P01503914	Zc3h15	1.94968
A_68_P09941671	Sstr1	1.949546
A_68_P09648257	Tha1	1.948933
A_68_P11646964	Abhd4	1.946547
A_68_P11311120	Trim35	1.946456
A_68_P03455183	Pappa	1.946385
A_68_P15160009	Usp9x	1.946143
A_68_P04263717	Tbc1d1	1.943967

A_68_P00866275	B3gnt7	1.943838
A_68_P05844417	6332401O19Rik	1.942303
A_68_P14284359	Trim8	1.941409
A_68_P07265861	Pard3	1.940612
A_68_P05636961	Fkbp14-Plekha8	1.939769
A_68_P14391999	Pten	1.938965
A_68_P08365485	Mbc2	1.938767
A_68_P01716379	Cbln4	1.938124
A_68_P09651122	2400006H24Rik	1.934648
A_68_P00892242	Rgs16	1.934632
A_68_P00740546	Fzd5	1.93438
A_68_P12620501	A_68_P12620501	1.933355
A_68_P05234904	Vps24	1.932617
A_68_P07729237	Dnmt1	1.93211
A_68_P09433957	0610009B22Rik	1.932084
A_68_P09349429	Pscd1	1.931757
A_68_P08465679	4930430F08Rik	1.930634
A_68_P07890310	Axud1	1.929324
A_68_P00456630	Nck2	1.928157
A_68_P07439349	Kcnk1	1.927991
A_68_P09669852	Car10	1.927179
A_68_P11481863	Ift88	1.927044
A_68_P09817675	0610009D07Rik	1.926969
A_68_P06620745	Phca	1.925161
A_68_P00938087	Angel2	1.923666
A_68_P10044582	Gphn	1.919722
A_68_P09017457	2310040C09Rik	1.91907
A_68_P03079371	Txnip	1.918119
A_68_P01876102	Dpm1	1.917988
A_68_P01300229	AA467197	1.914943
A_68_P00776589	Zfp142-Bcs1l	1.914869
A_68_P12958422	Alg3-1810009K13Rik	1.913965
A_68_P07887111	Dock6	1.913214
A_68_P06158591	Fgf3	1.911033
A_68_P04210835	Pdx1	1.908985

A_68_P13439828	Ptprs	1.908575
A_68_P10007352	Sfrs5	1.907147
A_68_P07651826	Camkv	1.907073
A_68_P09249597	Ntn1	1.906636
A_68_P14527591	Klhl15	1.903352
A_68_P13871877	AW554918	1.901854
A_68_P06062478	Zfp74	1.901011
A_68_P07809708	Rbms3	1.90013
A_68_P10586886	1110007C09Rik	1.898274
A_68_P04667146	Eif2b4	1.897879
A_68_P05417396	Repin1	1.897635
A_68_P06413702	Prss8	1.895674
A_68_P11120668	Foxd1	1.895428
A_68_P03223683	Rarsl	1.894904
A_68_P13228720	Ccdc128	1.892804
A_68_P14333432	Eml3	1.888811
A_68_P03474445	Rdhe2	1.888319
A_68_P12183136	Fmnl3	1.887823
A_68_P10061368	Pygl	1.886236
A_68_P08314943	Oaz1	1.886217
A_68_P09838513	Fos	1.885224
A_68_P07790130	Slc17a5	1.883521
A_68_P01401963	Hoxd11	1.88264
A_68_P01516110	Csrp2bp	1.881846
A_68_P01197425	Ndufaf1	1.879618
A_68_P05581130	Bpgm	1.879051
A_68_P09672660	Sox9	1.878904
A_68_P12069494	Tars	1.878792
A_68_P08148574	Raver1	1.878444
A_68_P14431117	Sf1	1.878406
A_68_P00793457	Aldh9a1	1.878231
A_68_P03245499	Ripk2	1.877528
A_68_P12962987	Yars2	1.877052
A_68_P05222011	Prmt8	1.876623
A_68_P08546101	Rdh7	1.876195

A_68_P03655599	ENSMUST00000095076.1:224	1.875514
A_68_P10947641	Hist1h1c	1.87511
A_68_P03439026	2810432D09Rik	1.874903
A_68_P08436635	D630029K05Rik	1.873171
A_68_P04952826	Crlz1	1.873044
A_68_P06075552	Bet1l-Ric8	1.870882
A_68_P12007011	Exosc4	1.868498
A_68_P14159635	Hrasls3	1.867799
A_68_P05266008	Zfp398	1.867225
A_68_P07676962	Eif3s4	1.866679
A_68_P01189722	St6galnac4	1.866021
A_68_P04667146	Eif2b4	1.86455
A_68_P04138116	Fzd10	1.8636
A_68_P12304021	Ncald	1.863015
A_68_P12697673	Vpreb1-Top3b	1.862771
A_68_P12545629	Rbm11	1.862306
A_68_P06268416	Stk32c	1.861404
A_68_P06935487	Arhgef7	1.860604
A_68_P09221699	Crkrs	1.860319
A_68_P08400813	Cited2	1.859894
A_68_P12246611	Myo10	1.859541
A_68_P13334488	Ltbp1	1.858353
A_68_P10082912	Txndc1	1.858171
A_68_P01486059	Samhd1	1.857424
A_68_P01703022	2610042O14Rik	1.855304
A_68_P02423351	Tacr3	1.853351
A_68_P00445898	Traf5	1.852635
A_68_P11139984	Nefl	1.851324
A_68_P01382492	D730039F16Rik	1.851049
A_68_P03938702	Spen-B330016D10Rik	1.849492
A_68_P10997874	Ptch1	1.849287
A_68_P02301655	Mrpl9	1.848722
A_68_P08250811	Matk	1.848539
A_68_P06586389	Hdgfrp3	1.846919
A_68_P10593859	Cox7c	1.84656

A_68_P13318970	Trim10	1.84607
A_68_P14154231	Mgea5	1.845361
A_68_P11431014	Mrp63	1.845236
A_68_P05969121	Tnfrsf19l	1.845226
A_68_P08976800	2410008K03Rik	1.844374
A_68_P05459434	Atoh8	1.84427
A_68_P05438081	Kcna1	1.843801
A_68_P09685128	Ormdl3	1.843799
A_68_P04560031	Crmp1	1.843033
A_68_P09647261	Rpl38	1.842621
A_68_P04538401	Rxfp2	1.841305
A_68_P01621810	2310010M24Rik	1.840758
A_68_P09006082	Hoxb4	1.83987
A_68_P13176455	Galnt14	1.838707
A_68_P12563206	Ncbp2	1.838584
A_68_P03630396	Asah3l	1.838544
A_68_P02791266	Bcar3	1.837614
A_68_P13219394	4732474A20Rik	1.837392
A_68_P06967275	Chst5	1.837288
A_68_P06166301	Sphk2	1.83645
A_68_P04684119	Prkag2	1.836316
A_68_P02997179	Col25a1	1.835986
A_68_P10443608	Rgnef	1.834864
A_68_P10373895	Strn3	1.833957
A_68_P14131438	Cdx1-Pdgfrb	1.833786
A_68_P14369749	Minpp1	1.833212
A_68_P09856737	Hspa2	1.833131
A_68_P09159521	Wdr68	1.832603
A_68_P13317232	Nudt12	1.832238
A_68_P11457289	Isgf3g	1.831971
A_68_P09506042	Klhl11	1.830911
A_68_P06573624	Hras1-Lrrc56	1.830581
A_68_P08477979	Nfic	1.829644
A_68_P14557687	Fmr1	1.82935
A_68_P14478327	Cyp26a1	1.829157

A_68_P13799338	Zfp438	1.828568
A_68_P06348925	Tgfb1	1.827855
A_68_P14183872	Rbm14	1.827755
A_68_P08883214	Trhde	1.82693
A_68_P10383006	Ttc8	1.826405
A_68_P00209581	Ripk5	1.825991
A_68_P05430011	Setd5	1.825365
A_68_P04595878	Dhx15	1.825298
A_68_P11769809	Hs6st3	1.824872
A_68_P10194608	Gpr65	1.824486
A_68_P00288847	C030014K22Rik	1.824318
A_68_P02437875	Wdr77	1.822024
A_68_P00389123	Adss	1.821613
A_68_P02074284	Frmd5	1.819823
A_68_P03581015	Ubiad1	1.819455
A_68_P13980028	Abhd3	1.817887
A_68_P04548919	Bapx1	1.81605
A_68_P03634721	Ccdc21	1.815145
A_68_P08341329	Fstl3	1.813945
A_68_P12109018	Rapgef3	1.813844
A_68_P01593485	Rpl35	1.812215
A_68_P13331989	Scube3	1.811348
A_68_P04047233	Necap2	1.810617
A_68_P02560176	Efna1	1.810066
A_68_P08386191	AK122525	1.80997
A_68_P12008927	Myc	1.809744
A_68_P06855930	Ednra	1.809399
A_68_P04601964	Qdpr	1.808536
A_68_P08153495	Slc26a6	1.808466
A_68_P11888723	Poldip3	1.808406
A_68_P06660845	Arntl	1.8083
A_68_P10511179	Ryr2	1.806094
A_68_P14154178	Ina	1.805548
A_68_P04800330	Nup54	1.804385
A_68_P11172854	Egr3	1.80396

A_68_P11713637	1700123O20Rik	1.803103
A_68_P05892293	Sbk1	1.801705
A_68_P13214626	Trip10	1.801488
A_68_P04182394	2810006K23Rik	1.800519
A_68_P06096079	Adam12	1.800359
A_68_P09565009	D11Ertd636e	1.80029
A_68_P08473002	Smarcb1	1.799823
A_68_P07318774	Gcsh	1.798402
A_68_P13330489	Ehd3	1.797926
A_68_P00020230	Rnf152	1.793962
A_68_P01286134	Ptpn1	1.793102
A_68_P06987885	Ppp1r3b	1.792529
A_68_P11711513	Rpp14	1.790412
A_68_P13510246	Srpk1	1.78949
A_68_P06330220	Pop4	1.789264
A_68_P00872357	Zbtb41	1.788646
A_68_P10186628	Dgkb	1.788447
A_68_P04846735	Rasgef1b	1.787135
A_68_P01319108	Urm1	1.785789
A_68_P06334357	Kctd15	1.784588
A_68_P09423036	Hes7	1.784279
A_68_P14103444	Ppp2r2b	1.783314
A_68_P00786420	Tnrc15	1.781588
A_68_P08766840	Atcay-9130206N08Rik	1.780705
A_68_P03699707	Ints8	1.778848
A_68_P09241039	Tmc6-Tmc8	1.778752
A_68_P04729371	Ap1s1	1.778535
A_68_P07422547	Cpne7	1.777714
A_68_P00606676	Gpa33	1.777635
A_68_P05952222	Gab2	1.777073
A_68_P09238663	Psmd11	1.776178
A_68_P03581015	Ubiad1	1.775831
A_68_P09261833	Eme1	1.775159
A_68_P05273970	Plxna4	1.774646
A_68_P00388163	Ccnt2	1.773014

A_68_P03775227	Mllt3	1.772663
A_68_P12210303	Hdac10	1.772369
A_68_P13748923	Zadh2	1.771622
A_68_P14704384	Cited1	1.771592
A_68_P03776838	Adfp	1.769984
A_68_P07374777	Irf2	1.769691
A_68_P03811052	4732473B16Rik	1.769297
A_68_P03864894	Bmp8a	1.766879
A_68_P09708163	Hoxb9	1.766718
A_68_P06299201	Snrpa1	1.764489
A_68_P10787950	Isl1	1.763965
A_68_P09161409	Scrn2	1.763278
A_68_P00000588	Tmem37	1.762367
A_68_P04125045	Prom1	1.761691
A_68_P08332822	Foxo3a	1.761521
A_68_P09218206	Ift20	1.761444
A_68_P01681116	Hars2	1.761426
A_68_P01674549	2610528K11Rik	1.761283
A_68_P01516448	Acvr1	1.760655
A_68_P12180199	Lrrc24	1.760408
A_68_P06816947	Ddx39	1.760161
A_68_P07155758	Ctcf	1.759927
A_68_P13700686	Wdr36	1.759812
A_68_P11593283	D930049A15Rik	1.758734
A_68_P09207936	Sf3a1	1.758458
A_68_P00998730	Apoa2	1.758143
A_68_P07169042	AA960436	1.757389
A_68_P05969116	Tnfrsf19l	1.756815
A_68_P03184721	Sf3b4	1.75656
A_68_P04324543	Nat8l	1.755839
A_68_P01755233	4930519N13Rik	1.755724
A_68_P08376671	Dusp6	1.754486
A_68_P11911306	Fkbp11	1.754095
A_68_P08984861	Zfp39	1.754037
A_68_P09503254	BC018371	1.753894

A_68_P03864879	Toe1	1.752791
A_68_P14159560	AI450540	1.752385
A_68_P07933913	Tspan3	1.752353
A_68_P10327112	Six1	1.751268
A_68_P07738687	Ncam1	1.75125
A_68_P05315942	Tspan33	1.751159
A_68_P11186339	Slc7a8	1.750646
A_68_P13317958	Acat3	1.750378
A_68_P08663643	Tspyl4	1.750367
A_68_P00722343	Des	1.750316
A_68_P01202677	Dlx2	1.749273
A_68_P03867476	Clstn1	1.748435
A_68_P00328324	Tuba4	1.745586
A_68_P08293317	Pah	1.74374
A_68_P00103028	Asb1	1.743407
A_68_P05962045	2900092E17Rik	1.742634
A_68_P14284014	2700081O15Rik	1.74255
A_68_P00995388	Stk16	1.742184
A_68_P06079493	Neud4	1.741264
A_68_P13152165	Ppp2r1a	1.740901
A_68_P00170988	Tmem177	1.74037
A_68_P04283534	Sgcb	1.738892
A_68_P14015951	Cabyr	1.738727
A_68_P07403071	Fut10	1.738418
A_68_P01324447	Mafb	1.736316
A_68_P14555209	Ndufa1-Rnf113a1	1.735577
A_68_P02947763	Spg20	1.735354
A_68_P08907905	Ppm1h	1.735003
A_68_P09978294	Papln	1.734515
A_68_P13905974	Tubb6	1.734457
A_68_P04595878	Dhx15	1.734154
A_68_P00872587	Smg7	1.733663
A_68_P12623101	Txndc11	1.733618
A_68_P09554165	Eif4enif1	1.733182
A_68_P00579109	Rabgap1l	1.732825

A_68_P02088647	Atp5g3	1.731854
A_68_P13508778	Wdr27-1600012H06Rik	1.73184
A_68_P13190395	Pacsin1	1.73159
A_68_P14378156	Slc15a3	1.73105
A_68_P07596191	Kirrel3	1.730882
A_68_P09474548	Hoxb3	1.730867
A_68_P05749221	Cops7a	1.730325
A_68_P01804677	Polr3f	1.730187
A_68_P12416339	Myh9	1.728982
A_68_P14409670	Rce1	1.728658
A_68_P11475668	Psme2-Rnf31	1.728398
A_68_P00179487	Dusp27	1.728198
A_68_P12069241	Phf5a	1.72766
A_68_P10039268	Rps7	1.727634
A_68_P14198559	Tmem132a	1.726872
A_68_P12916411	Aifm3	1.726358
A_68_P03516417	Fndc5	1.726181
A_68_P05290376	Pigy	1.726054
A_68_P04729379	Ap1s1	1.725298
A_68_P06225259	Zfp36	1.725124
A_68_P02263285	Prkra	1.723874
A_68_P08840705	Arid3a	1.723529
A_68_P11363424	Pcdh8	1.723335
A_68_P12395393	Tcf20	1.722789
A_68_P06424695	Irf2bp1	1.722317
A_68_P01458385	2810003C17Rik	1.721704
A_68_P03867476	Clstn1	1.720799
A_68_P08453527	Arl1	1.720695
A_68_P11686771	Egr3	1.720471
A_68_P01455729	Gata3	1.720392
A_68_P05769184	Plxna1	1.720205
A_68_P14560524	Chst7	1.720109
A_68_P03503055	Dmrta1	1.719373
A_68_P00443803	Ptma	1.718924
A_68_P00828037	AW822216	1.718648

A_68_P03434986	Hes3-Icmt	1.718577
A_68_P02301018	Ahcyl1	1.718529
A_68_P09052542	Alkbh5	1.7185
A_68_P06245175	Nr2f2	1.717927
A_68_P05768851	Gmcl1	1.716762
A_68_P05298582	Tex261	1.716549
A_68_P11724371	Ngdn	1.716068
A_68_P13547884	Pla2g7	1.715797
A_68_P12456697	Mtdh	1.715426
A_68_P04596407	Dtx1	1.713837
A_68_P03743542	Zswim5	1.713427
A_68_P01640750	Zbtb6	1.712778
A_68_P07423734	Slc7a5	1.712762
A_68_P09703840	Epn3	1.71174
A_68_P01680443	Svs2	1.711699
A_68_P07370238	Pgpep1	1.711637
A_68_P10785733	1300014I06Rik	1.710798
A_68_P13332835	Vapa	1.709893
A_68_P06023142	Rnh1	1.709555
A_68_P06272066	Chrna7	1.708289
A_68_P08843330	5330439J01Rik	1.708279
A_68_P14420129	Ldb1	1.707862
A_68_P06965881	Bcnp1	1.707692
A_68_P07498595	Cdc25a	1.707615
A_68_P04306176	Asl-Crcp	1.707418
A_68_P09557146	Rpl23	1.707408
A_68_P05730788	Ptms	1.707111
A_68_P12503063	Parn-Bfar	1.707046
A_68_P05606174	Ogg1	1.70665
A_68_P05303383	Tsen2	1.705893
A_68_P04884115	Scfd2	1.705755
A_68_P10002168	Dpf3	1.705204
A_68_P02325182	E130311K13Rik	1.705071
A_68_P07831910	Tle3	1.704956
A_68_P13460407	Tcte2	1.704381

A_68_P03328783	Ube2j2	1.703715
A_68_P12807535	2410018G20Rik	1.703577
A_68_P13503075	Dus3l	1.703471
A_68_P13384191	Sfrs3	1.702603
A_68_P09066771	Erbb2	1.702551
A_68_P11303295	Spcs1-Glt8d1	1.702497
A_68_P02020198	Dll4	1.702451
A_68_P11755499	Mtmr6	1.701921
A_68_P06301883	Vasp	1.701378
A_68_P08890341	Spock2	1.701125
A_68_P05590268	Bhlhb3	1.70085
A_68_P09262493	Aldh3a2	1.699748
A_68_P02134951	Ypel4	1.69969
A_68_P03781928	Rpa2	1.699175
A_68_P14773710	Magee1	1.698852
A_68_P04306176	Asl-Crcp	1.698774
A_68_P04403733	En2	1.698519
A_68_P09080309	ENSMUST00000083461.1:-590	1.697824
A_68_P09142915	Psme4	1.697293
A_68_P06544357	B4galnt4	1.696913
A_68_P01293841	Samd10	1.696364
A_68_P09704973	Mif4gd	1.695739
A_68_P11960753	Pick1	1.694812
A_68_P12281145	Mal2	1.693748
A_68_P12108249	Hoxc10	1.693111
A_68_P14823993	Suhw3	1.692958
A_68_P09708172	Hoxb9	1.692604
A_68_P06900371	Ogfod1	1.691893
A_68_P01584465	Gata3	1.691341
A_68_P12616352	2310008H04Rik	1.690565
A_68_P04877098	Slc2a9	1.690487
A_68_P12751159	B830017H08Rik	1.688537
A_68_P10541875	Heatr1	1.688105
A_68_P06578180	D930014E17Rik	1.688002
A_68_P08431950	Fyn	1.687206

A_68_P00288848	C030014K22Rik	1.685283
A_68_P07506973	Entpd3	1.684866
A_68_P10863564	Net1	1.68452
A_68_P13093048	1600002H07Rik	1.684437
A_68_P03260050	Fblim1	1.684392
A_68_P07220940	Irx3	1.683895
A_68_P10682964	Hist1h1a	1.683234
A_68_P03553944	Plk3	1.683078
A_68_P10677979	Zfp322a	1.682715
A_68_P08286568	Tmem19	1.682456
A_68_P05817882	Mrpl19	1.680437
A_68_P07069639	Bag4-Lsm1	1.680056
A_68_P06469960	Cttn	1.679442
A_68_P07653994	Prkcsh	1.679157
A_68_P05396284	D830050J10Rik	1.678857
A_68_P10039265	Rps7	1.678073
A_68_P13512342	Epas1	1.678065
A_68_P04722013	Ccdc98	1.675983
A_68_P04877098	Slc2a9	1.675097
A_68_P12219855	Hoxc5	1.674433
A_68_P12396652	Egflam	1.67426
A_68_P07321881	Gnao1	1.673656
A_68_P14356802	Dmrt2	1.673581
A_68_P06522130	Vps33b	1.672889
A_68_P14671288	Fgd1	1.672744
A_68_P01498746	Wfdc2	1.672504
A_68_P09507747	Rab1	1.67214
A_68_P08737710	Atp5b	1.671607
A_68_P02866300	Alg5	1.670905
A_68_P07915232	Trip4-Csnk1g1	1.670707
A_68_P07180083	Rnf166	1.670448
A_68_P12027768	Rbm9	1.670286
A_68_P04500317	Crlz1	1.669335
A_68_P05701894	Rpia	1.668941
A_68_P11868103	Khdrbs3	1.668939

A_68_P09087916	Stc2	1.668905
A_68_P11025669	Tmem14c	1.668857
A_68_P02133839	Plxdc2	1.668637
A_68_P10589495	Hist1h3d	1.668575
A_68_P00091256	Kcne4	1.668537
A_68_P09547535	BC017647	1.667692
A_68_P13626011	Iws1	1.667263
A_68_P04178934	Poln	1.667051
A_68_P10129716	Spata7	1.66479
A_68_P01025277	Il10	1.664124
A_68_P13075872	Mas1-Mrgprh	1.6641
A_68_P03953505	Ppp1r8	1.663976
A_68_P06141027	Zfp260	1.663519
A_68_P12890514	ENSMUST00000083623.1:-2960	1.663158
A_68_P09838128	Nudt14	1.662503
A_68_P13161337	Rpl10a	1.662411
A_68_P01768988	Drbp1	1.662359
A_68_P15046048	Gm784	1.661855
A_68_P02202234	Sstr4	1.661746
A_68_P12158711	Sqle	1.661348
A_68_P13816356	Lman1	1.66125
A_68_P12915716	Fgf12	1.661158
A_68_P15077927	Prkx	1.66101
A_68_P03376755	Tmem50a-D4Wsu53e	1.660885
A_68_P07289572	Atbf1	1.660011
A_68_P11457660	Gja3	1.660002
A_68_P03188323	Mtx1-Thbs3	1.659444
A_68_P03745420	Ak3l1	1.658886
A_68_P05957743	Nkx6-2	1.658826
A_68_P09321734	ENSMUST00000083461.1:-1777	1.658065
A_68_P12584284	Dscr3	1.657802
A_68_P00432589	Ormdl1	1.65629
A_68_P01419213	Nudt5	1.656072
A_68_P06693274	Irx5	1.65607
A_68_P11677837	Bmp4	1.656029

A_68_P01752384	Nkx2-4	1.655796
A_68_P06355575	Slco3a1	1.655768
A_68_P10030336	C79407	1.655707
A_68_P01622552	C230093N12Rik	1.655381
A_68_P01695051	Jag1	1.655124
A_68_P06307345	Tial1	1.654845
A_68_P15201514	Tbl1x	1.654516
A_68_P03409745	Il28ra	1.653951
A_68_P09719063	Cbx4	1.653835
A_68_P05128177	1200009O22Rik	1.65334
A_68_P14329457	Taf5	1.652192
A_68_P13462804	Igfals	1.652167
A_68_P02035365	Rnd3	1.65114
A_68_P06803453	Tlr3	1.650893
A_68_P09085134	Ube2b	1.649532
A_68_P11070381	Otp	1.649385
A_68_P01120360	Igsf8	1.649202
A_68_P01482765	Hoxd12	1.648778
A_68_P09149834	Nxn-Timm22	1.6485
A_68_P14229223	Cpn1	1.648461
A_68_P13176695	Cul7	1.647963
A_68_P09330633	Kcnab3	1.647857
A_68_P01718932	Ift52	1.647155
A_68_P10113821	Jundm2	1.646612
A_68_P08075208	Slc25a36	1.646602
A_68_P06444726	Snrpa	1.646338
A_68_P10175037	Kcnh5	1.646201
A_68_P06633522	Igf1r	1.646172
A_68_P04606893	Pex1	1.646168
A_68_P04460565	Sh2b3	1.645025
A_68_P10526799	Pik3r1	1.644761
A_68_P01402496	Mfap1a	1.644506
A_68_P02138222	Ogfr	1.644398
A_68_P09651376	Actg1	1.644165
A_68_P12323949	LOC432982	1.643447

A_68_P03798082	Urod	1.6433
A_68_P03413444	Col9a2	1.6431
A_68_P09408321	Slc39a11	1.642626
A_68_P08138725	Mcam	1.642431
A_68_P03798082	Urod	1.641807
A_68_P12873481	Dscr2	1.641582
A_68_P03984305	Nfib	1.640884
A_68_P02065709	Dpm1	1.640334
A_68_P13230180	AU023871	1.639589
A_68_P12651543	Pla1a	1.63957
A_68_P10953282	Gadd45g	1.639249
A_68_P04789378	Caln1	1.639172
A_68_P03263077	2410166I05Rik	1.63904
A_68_P00503604	Hes6	1.638552
A_68_P03703211	Tesk2	1.637874
A_68_P09153352	Rnf185	1.637563
A_68_P00896384	Zfp617-Spata3	1.637487
A_68_P03984305	Nfib	1.637087
A_68_P11815325	Jub	1.636895
A_68_P04582584	Flt1	1.636686
A_68_P00153972	Creg1	1.635479
A_68_P10223860	Sox11	1.634773
A_68_P13015323	2510009E07Rik	1.634377
A_68_P06398753	Cpeb1	1.634209
A_68_P00575076	Ivns1abp	1.63407
A_68_P11677501	Gfra2	1.633858
A_68_P13957248	Fzd8	1.633823
A_68_P01216278	1110008F13Rik	1.633377
A_68_P09322158	Utp6	1.632617
A_68_P01911888	Chrna4	1.632172
A_68_P02198501	Vapb	1.6311
A_68_P14139352	Sall3	1.630361
A_68_P05016628	Gata2	1.6299
A_68_P14217829	Klf9	1.629731
A_68_P00972983	Pdcl3	1.629669

A_68_P03746953	Akr1a4	1.62947
A_68_P09005238	Hoxb1	1.629429
A_68_P07978338	Thsd4	1.628728
A_68_P13992999	Pcdhgb2	1.628373
A_68_P14073891	Neto1	1.628304
A_68_P12795932	Es2el	1.627915
A_68_P06139805	Sae2	1.627884
A_68_P08835415	Onecut3	1.626205
A_68_P01772847	Ypel4	1.626205
A_68_P06975483	Gpr124	1.625469
A_68_P13637118	Dpysl3	1.625419
A_68_P13465611	Cbs	1.625031
A_68_P04460565	Sh2b3	1.624596
A_68_P08698894	Bclaf1	1.62414
A_68_P09525412	Hes7	1.624122
A_68_P02032707	Prpf40a	1.623532
A_68_P10198482	Akt1	1.623017
A_68_P11246048	Fgf17	1.622765
A_68_P08459023	Nrbf2	1.621846
A_68_P00119253	B4galt3	1.620203
A_68_P03666104	Mrto4	1.619213
A_68_P03864879	Toe1	1.618965
A_68_P03912404	Aqp7	1.618678
A_68_P09650324	Cabp7	1.618492
A_68_P10492549	Ippk	1.618467
A_68_P12638261	Pcqap	1.618384
A_68_P13253297	ENSMUST00000083557.1:-775	1.618059
A_68_P08023924	Smad6	1.617739
A_68_P09637312	Ulk2	1.617675
A_68_P03291536	Pdpn	1.61758
A_68_P09586180	Trim65	1.617272
A_68_P14936994	Gata1	1.617187
A_68_P12599275	Etv5	1.617063
A_68_P14356799	Dmrt2	1.616948
A_68_P14204143	Hspa12a	1.616443

A_68_P09726526	Ccng1	1.616104
A_68_P09369752	Ndel1	1.615859
A_68_P11205877	Zfp503	1.61576
A_68_P01570529	Hoxd3	1.615424
A_68_P03215971	Zswim5	1.615338
A_68_P02555897	Mcl1	1.615005
A_68_P13535883	Mapk13	1.614586
A_68_P01075332	Nmur1	1.614558
A_68_P09522952	Nol11	1.613945
A_68_P08398844	BC030440	1.6134
A_68_P04316595	Tesc	1.6129
A_68_P05117018	Igf2bp3	1.612398
A_68_P03334643	Npr2	1.611692
A_68_P04570465	Nptx2	1.61121
A_68_P04005930	9630013D21Rik	1.611127
A_68_P09883093	Clmn	1.610729
A_68_P04834757	Cutl1	1.61044
A_68_P03953505	Ppp1r8	1.6102
A_68_P09962049	Nfkbia	1.610103
A_68_P04373781	Pcdh7	1.609632
A_68_P07199454	Ctcf	1.609209
A_68_P02947753	Spg20	1.60909
A_68_P00488035	Clk1	1.608924
A_68_P08433555	Atcay	1.608457
A_68_P04693611	9130213B05Rik	1.608011
A_68_P08046867	Usp2	1.607949
A_68_P13324804	Chd1	1.607718
A_68_P11613508	Kctd12	1.60769
A_68_P14255652	Cybasc3	1.607688
A_68_P12730420	Igf2bp2	1.607412
A_68_P03666104	Mrto4	1.606922
A_68_P11916196	Ttc33	1.606194
A_68_P01538815	D2Ertd435e	1.605691
A_68_P12215542	Parvb	1.605679
A_68_P05891012	2400001E08Rik	1.605029

A_68_P03222015	Prdm16	1.604969
A_68_P06026485	St8sia2	1.604493
A_68_P11113445	Ranbp9	1.604118
A_68_P03917465	Foxd2	1.603335
A_68_P10961798	Gpbp1	1.602758
A_68_P03909943	Arid1a	1.602169
A_68_P09065321	Nfe2l1	1.601599
A_68_P09477576	Vamp2	1.601511
A_68_P04836511	Bcl7a	1.601459
A_68_P05885283	Kctd15	1.601459
A_68_P07422084	Tmco7	1.600974
A_68_P12138298	Gsdmdc1	1.600806
A_68_P07728979	Snf1lk2	1.60077
A_68_P06289528	Clns1a	1.600467
A_68_P06674043	Large	1.600086
A_68_P12788633	Tmem41a	1.599571
A_68_P04824992	Gtf3c2	1.598878
A_68_P14186183	Ccdc87	1.598783
A_68_P07623655	Pou2f3	1.598781
A_68_P00339676	Ctdsp1	1.597805
A_68_P02791087	Acadm	1.597704
A_68_P07217678	Mmp15	1.597658
A_68_P09296637	Mgat4b	1.597529
A_68_P13227104	Snf1lk	1.597192
A_68_P01836800	A130092J06Rik-Ppapdc3	1.596842
A_68_P01129968	Bivm	1.596706
A_68_P03169150	Mcoln2	1.59647
A_68_P05078221	Mrpl53	1.596401
A_68_P08088647	Pvrl1	1.596354
A_68_P03093935	Prpf38b	1.59629
A_68_P06832469	Junb	1.595502
A_68_P11739634	Gata4	1.595147
A_68_P04306177	Crcp	1.595028
A_68_P11896312	Sp1	1.594245
A_68_P07966190	Slc44a2	1.593817

A_68_P12462766	Top1mt	1.592435
A_68_P03935395	Aqp3	1.592223
A_68_P12731974	Wrb	1.59168
A_68_P00343608	Tmeff2	1.590867
A_68_P04964381	Cabp1	1.590723
A_68_P06686368	Il15	1.590254
A_68_P07351554	Ndufa13	1.590137
A_68_P09203526	Spnb2	1.590009
A_68_P00677413	Lmod1	1.589942
A_68_P05744313	Mrps33	1.589857
A_68_P05827700	Hoxa10	1.588593
A_68_P10256062	Map4k5	1.58856
A_68_P05059002	5730419I09Rik	1.588248
A_68_P01678630	Acss1	1.588027
A_68_P09320905	1810046J19Rik	1.587833
A_68_P11875450	A_68_P11875450	1.587818
A_68_P11760124	Tssk4	1.58776
A_68_P06912647	Lpl	1.587527
A_68_P09341975	Slfn8	1.587394
A_68_P02662675	Rap2b	1.587359
A_68_P10346375	Six1	1.587182
A_68_P04609970	B3bp	1.586684
A_68_P08365472	Mbc2	1.58658
A_68_P07941290	Ttc12	1.585867
A_68_P12584231	Adamts5	1.585856
A_68_P13465706	Caskin1	1.585567
A_68_P01808464	Pcsk2	1.585498
A_68_P14495742	Slc18a2	1.585071
A_68_P13855404	BC031181	1.584546
A_68_P06464175	Dgat2	1.584464
A_68_P09501340	0610025P10Rik	1.584347
A_68_P07958990	A_68_P07958990	1.583632
A_68_P06052133	Unc45a-Hddc3	1.583581
A_68_P02581370	Wnt2b	1.583111
A_68_P08084541	4933406E20Rik	1.582515

A_68_P12793055	ENSMUST00000083515.1:-5650	1.581937
A_68_P09312885	Mnt	1.581936
A_68_P04751677	Sparcl1	1.581473
A_68_P01924968	Freq	1.581218
A_68_P13393953	Slc25a27-Cyp39a1	1.580973
A_68_P07600764	Higd1a	1.580316
A_68_P11363422	Pcdh8	1.580153
A_68_P04286510	Pebp1	1.580023
A_68_P00119249	B4galt3	1.578837
A_68_P06120468	Nkx6-2	1.578799
A_68_P08569765	Mdm1	1.578612
A_68_P02499753	Rap1gds1	1.577044
A_68_P03888598	Slc25a33	1.575797
A_68_P05623653	Hoxa6	1.575652
A_68_P05005487	Snca	1.575429
A_68_P06461192	Mrps12	1.575194
A_68_P08347279	Chst3	1.574883
A_68_P09031067	Azi1	1.574773
A_68_P11518015	Lgi3	1.574769
A_68_P13654284	Impact	1.574452
A_68_P07114791	Cyld	1.574371
A_68_P05583186	Tgfa	1.573982
A_68_P03555259	Cdkn2a	1.573917
A_68_P07037628	Gse1	1.573917
A_68_P08873702	Pwp1	1.573553
A_68_P12298724	Npr3	1.573441
A_68_P08156833	D630004A14Rik	1.573226
A_68_P12989636	Rogdi	1.572532
A_68_P15169386	G6pdx	1.572365
A_68_P11173760	Efs-Il25	1.572289
A_68_P08312291	Tfam	1.571612
A_68_P00918692	2310035C23Rik	1.571385
A_68_P02434633	Il7	1.571314
A_68_P12204111	Derl1	1.571114
A_68_P13483729	Cdc5l	1.570945

A_68_P12241657	Plec1	1.570196
A_68_P05741964	Mkln1	1.570115
A_68_P08513385	Slc35d3	1.569858
A_68_P00151559	Rcor3	1.569451
A_68_P14230217	Mus81-Cfl1	1.569397
A_68_P03944448	Ppap2b	1.56938
A_68_P04697714	Tcf1	1.569312
A_68_P06953539	Homer3	1.568684
A_68_P13205297	Cyp1b1	1.568284
A_68_P02721475	Ampd2	1.567963
A_68_P00184086	C130074G19Rik	1.566469
A_68_P06491197	Btbd10	1.566154
A_68_P07666902	Calml4	1.566102
A_68_P02133861	Plxdc2	1.56601
A_68_P10073346	Mark3	1.565744
A_68_P09012371	Arf1	1.565734
A_68_P13496133	BC052484	1.56558
A_68_P02035368	Rnd3	1.565499
A_68_P04987277	Lhx5	1.565271
A_68_P07217865	Dpep1	1.565167
A_68_P00531572	Otos	1.564629
A_68_P04362592	Rufy3	1.564574
A_68_P10202509	Aldh6a1	1.564367
A_68_P08153393	Lars2	1.564274
A_68_P07524547	Ppm1m	1.564195
A_68_P08862608	Sgpl1	1.564169
A_68_P06388543	Fgf21	1.564111
A_68_P01418075	Zfp334	1.564082
A_68_P03452599	Stx17	1.563385
A_68_P05605215	Bcat1	1.563257
A_68_P06012484	2610207I05Rik-4930583K01Rik	1.563178
A_68_P03733795	Tcfap2e	1.563101
A_68_P02394230	Skil	1.563073
A_68_P03260050	Fblim1	1.562991
A_68_P09090040	Cep68	1.562771

A_68_P05752699	Impdh1	1.562264
A_68_P14147594	Tcirg1	1.561991
A_68_P05548217	Chd4	1.561694
A_68_P03909943	Arid1a	1.561661
A_68_P04491196	Uncx4.1	1.561354
A_68_P09090854	Slc16a6	1.56134
A_68_P14440236	Rad9	1.560908
A_68_P06069474	Wnt11	1.560818
A_68_P08436624	D630029K05Rik	1.560619
A_68_P05285116	Cpa2	1.560094
A_68_P10712042	Ubqln1	1.559997
A_68_P01273189	Sulf2	1.559526
A_68_P03301122	Galnt12	1.559034
A_68_P01564392	Nek6	1.558544
A_68_P10856510	Barx1	1.558298
A_68_P12273277	Acvrl1	1.558236
A_68_P12052843	AW549877	1.557381
A_68_P05118396	Cecr6	1.557115
A_68_P14393451	Ndufb8	1.556982
A_68_P13113749	BC038613	1.556752
A_68_P01159156	Ssfa2	1.556435
A_68_P00908136	Sumo1	1.555732
A_68_P14830739	Gnl3l	1.555556
A_68_P05123647	Edem1	1.555388
A_68_P08037587	Atg4d	1.555081
A_68_P03184042	Rnf13	1.555029
A_68_P04707007	Ars2	1.554576
A_68_P11665627	Zfhx2	1.554289
A_68_P05934093	Eef2k	1.554169
A_68_P04008295	AU022252	1.554017
A_68_P02215529	Fkbp1a	1.553938
A_68_P08649148	Stk11	1.553566
A_68_P12246610	Myo10	1.553134
A_68_P04306177	Crcp	1.551441
A_68_P03487933	Gpr3	1.55131

A_68_P06767570	AI427515	1.550907
A_68_P14274323	Xpnpep1	1.550768
A_68_P07909993	Elavl3	1.550438
A_68_P05322140	2010012C16Rik	1.550313
A_68_P11309177	Fndc3a	1.550136
A_68_P08174150	Cox5a	1.549866
A_68_P13182112	Metrn	1.549658
A_68_P03291536	Pdpn	1.54951
A_68_P01709615	A930018P22Rik	1.549397
A_68_P07775665	Sesn3	1.548598
A_68_P14343355	Map3k11	1.548395
A_68_P06552129	Arntl	1.548385
A_68_P03896383	Prdm16	1.547628
A_68_P13271863	Gtf2h4	1.547197
A_68_P01878753	C1ql3	1.546016
A_68_P12264171	Smug1	1.545662
A_68_P07758038	Tex264	1.545338
A_68_P11726164	Wdfy2	1.544784
A_68_P04008295	AU022252	1.544434
A_68_P12273274	Acvrl1	1.543698
A_68_P02452298	Lxn	1.543559
A_68_P09101677	Spred2	1.54354
A_68_P08715487	Cd24a	1.543471
A_68_P14674677	Tmem32	1.543402
A_68_P00317108	Cxcr4	1.543019
A_68_P08096900	Pias1	1.543
A_68_P02764946	Tbl1xr1	1.542878
A_68_P09718190	Sphk1	1.54266
A_68_P05179389	ENSMUST00000083652.1:-5555	1.54237
A_68_P14280238	Pik3ap1	1.542338
A_68_P02990496	Pitx2	1.540433
A_68_P08965912	Adora2a	1.540299
A_68_P12500587	Hira	1.540134
A_68_P12359376	AK162044	1.540092
A_68_P00138859	Hibch	1.540076

A_68_P12959248	Mrpl39	1.539564
A_68_P04987280	Lhx5	1.539328
A_68_P07916100	AY074887	1.538912
A_68_P02501934	4930504E06Rik	1.538333
A_68_P04736305	Znhit1	1.537835
A_68_P09443620	Tcf7	1.53776
A_68_P07212645	Zfp330	1.537736
A_68_P13150354	Lbh	1.537664
A_68_P14335838	Psat1	1.537478
A_68_P09987994	Pomc1	1.537178
A_68_P05804599	Mrpl19	1.537131
A_68_P06313338	Spon1	1.536538
A_68_P01201038	Casc4	1.536403
A_68_P00337663	Xkr4	1.535925
A_68_P15221797	Rps4x	1.535719
A_68_P06450771	Saa4	1.534994
A_68_P03439013	2810432D09Rik	1.534752
A_68_P03302039	Igsf21	1.534275
A_68_P05869378	Ncapd2-Mrpl51	1.534078
A_68_P15121231	Trappc2	1.534042
A_68_P12108244	Hoxc10	1.533999
A_68_P13223886	Shd	1.533886
A_68_P05907582	2700078K21Rik	1.533686
A_68_P00456627	Nck2	1.533365
A_68_P07467132	Slc38a3	1.533338
A_68_P01105977	Stat1	1.533201
A_68_P00904706	1810031K17Rik-BC038286	1.531932
A_68_P13186447	Kcnk12	1.531575
A_68_P01469068	Tcfap2c	1.531441
A_68_P03296663	Rnf38	1.530945
A_68_P01714764	Dnajc17	1.530897
A_68_P02917066	Gstm5	1.530772
A_68_P12048926	Plec1	1.530728
A_68_P14230211	Mus81-Cfl1	1.530032
A_68_P09264439	Srebf1	1.529813

A_68_P09369743	Ndel1	1.52925
A_68_P00766098	BC031781	1.52903
A_68_P13415565	Sepx1	1.528663
A_68_P10987290	Sdccag10-3110031B13Rik	1.528334
A_68_P10157510	A_68_P10157510	1.528278
A_68_P09357796	Rara	1.527757
A_68_P13119759	Nkx2-5	1.527748
A_68_P00740581	Fzd5	1.527718
A_68_P09574736	Sdf2	1.527632
A_68_P10975508	Gmnn	1.527299
A_68_P00327181	Lemd1	1.527265
A_68_P07935522	Sin3a	1.527051
A_68_P02024644	D030051N19Rik	1.526902
A_68_P12909205	Lmln	1.526883
A_68_P03569937	Psip1	1.52664
A_68_P04884115	Scfd2	1.526019
A_68_P08435476	Sf3b5	1.525375
A_68_P07441605	Cotl1	1.525314
A_68_P12854744	Adamts5	1.524922
A_68_P04663164	Fbxl13	1.523935
A_68_P14327516	Kcnk4	1.523671
A_68_P05272167	Igf2bp3	1.523323
A_68_P14350147	Gfra1	1.522771
A_68_P04851548	Lhx5	1.522571
A_68_P06994522	Bst2	1.522341
A_68_P00366170	1110034B05Rik	1.522251
A_68_P08841141	Ela2	1.52198
A_68_P09457498	Acbd4	1.521932
A_68_P01075518	Wnt6	1.521848
A_68_P04148375	Tmed5	1.521785
A_68_P09531764	Dlx4	1.521754
A_68_P13626996	Spry4	1.521536
A_68_P12745246	Parn	1.52144
A_68_P13409935	Wtap	1.520456
A_68_P06025177	Mesdc1	1.520406

A_68_P08949147	Rassf3	1.519627
A_68_P09185579	Mmd	1.519598
A_68_P10223655	Acot4	1.519111
A_68_P06788637	BC021891	1.519032
A_68_P09794243	A_68_P09794243	1.518623
A_68_P00122991	Rnpep	1.518313
A_68_P09264452	Srebf1	1.517881
A_68_P12600283	Pcyt1a	1.517737
A_68_P10699085	Sox4	1.517668
A_68_P12707523	Chrd	1.5176
A_68_P03584386	Bmp8b	1.51753
A_68_P00552426	Dst	1.517067
A_68_P08629718	B530045E10Rik	1.516672
A_68_P01137526	Niban	1.516635
A_68_P13992903	Gata6	1.51656
A_68_P00919787	Cd55	1.516467
A_68_P04339270	Tbx3	1.516363
A_68_P10735499	Txndc5	1.516041
A_68_P02258880	Gm561	1.515669
A_68_P01697623	Pbx3	1.515588
A_68_P00564995	Slc35f5	1.515539
A_68_P04930233	Rbm19	1.51503
A_68_P03296663	Rnf38	1.514947
A_68_P08523385	Smpd2	1.514766
A_68_P15097287	Mllt7	1.514609
A_68_P14142999	Smarca2	1.514315
A_68_P03843625	BC025833	1.514303
A_68_P02097439	Ptgis	1.513848
A_68_P12600716	Tiam1	1.513782
A_68_P09516342	Cdc27	1.513736
A_68_P01928464	Gm996	1.513504
A_68_P06458345	Sphk2	1.512936
A_68_P11331189	LOC432870	1.512829
A_68_P03630129	Thap3	1.512578
A_68_P08085317	Map2k5	1.512523

A_68_P07684876	Tgm4	1.512148
A_68_P12039466	D15Mgi27	1.511971
A_68_P07809628	Eef1a1	1.511496
A_68_P07804980	Pde4a	1.511336
A_68_P05591071	Pcbp1	1.510991
A_68_P09343920	BC030477	1.510702
A_68_P05822231	9630033F20Rik	1.510661
A_68_P13441074	Pacsin1	1.510282
A_68_P13134961	Tnfrsf21	1.509398
A_68_P06343664	Plekhg2	1.508641
A_68_P08999296	Wfikkn2	1.507785
A_68_P00906169	Cxcr7	1.507453
A_68_P06801217	Cc2d1a-4930432K21Rik	1.507074
A_68_P09016819	Pcgf2	1.507023
A_68_P07588982	Birc3	1.506965
A_68_P10421818	Sdc1	1.506705
A_68_P06358264	Slco3a1	1.506527
A_68_P13341641	Zdhhc14	1.50636
A_68_P07578379	Pknox2	1.504865
A_68_P09661960	Sphk1	1.504856
A_68_P06832423	Junb	1.504807
A_68_P13531990	Nubp2-Spsb3	1.504122
A_68_P03465779	Cdc20	1.502719
A_68_P13480940	Rgmb	1.502597
A_68_P08531609	Mtap7	1.502358
A_68_P04270189	C230008H04Rik	1.502254
A_68_P06531397	Suv420h2	1.502048
A_68_P11915780	Foxred2	1.501899
A_68_P00225463	Lamc2	1.501839
A_68_P10411213	Atxn7l4	1.50122
A_68_P04498604	Rnf34	1.501122
A_68_P07563562	Igsf4a	1.500649
A_68_P10402489	Slc25a29	1.500519
A_68_P13559195	Polh-Xpo5	1.500488
A_68_P10729669	Homer1	1.500433

A_68_P05534706	Lsm5	1.50035
A_68_P14263584	Foxb2	1.500345
A_68_P06105632	Cckbr	1.500108
A_68_P06035699	Plekhf1	1.499921
A_68_P04492972	Katnal1	1.499738
A_68_P11607346	Nefl	1.499354
A_68_P06031404	6330408A02Rik	1.499199
A_68_P13507114	Ccdc128	1.499033
A_68_P07860056	Bace1	1.498133
A_68_P11019732	Ddx41	1.498056
A_68_P08265273	Klf16	1.498033
A_68_P07954586	Epor	1.497437
A_68_P00396256	Hhat	1.49735
A_68_P00251563	Uap1	1.497242
A_68_P06678689	Xkr5	1.496959
A_68_P15160897	Phf6	1.496571
A_68_P02695523	A_68_P02695523	1.496302
A_68_P14097615	2010001M09Rik	1.495671
A_68_P01516077	Trim69	1.495562
A_68_P07955121	Tmem158	1.495359
A_68_P07060963	Zdhhc7	1.494905
A_68_P03693215	Zfp593	1.494683
A_68_P02317489	Hapln2-Gpatc4	1.49438
A_68_P02323952	Car3	1.494319
A_68_P06394783	Ube3a	1.494194
A_68_P01342780	Ccbl1	1.494124
A_68_P03810512	Zcchc7	1.494072
A_68_P13106799	Tulp1	1.493619
A_68_P09156463	Ppp1r1b	1.493382
A_68_P10129077	Ppp1r13b	1.493168
A_68_P14231112	Rps6kb2	1.492229
A_68_P11040896	Peci	1.491913
A_68_P08275638	Rfx4	1.491595
A_68_P00469149	Col5a2	1.491462
A_68_P13309401	Pbx2	1.491351

A_68_P09453654	Lsm12	1.490502
A_68_P05806684	Hoxa5	1.490446
A_68_P00780454	4930418G15Rik	1.490379
A_68_P05951991	Bub3	1.490217
A_68_P07487469	Cyb561d2	1.490214
A_68_P10725065	Elovl7	1.48993
A_68_P14471876	Sf3b2-Gal3st3	1.489361
A_68_P01771855	Snai1	1.489298
A_68_P00761115	5033414K04Rik	1.489213
A_68_P07674505	Ttk	1.489068
A_68_P07002293	Terf2	1.488294
A_68_P03452599	Stx17	1.488125
A_68_P00251562	Uap1	1.487897
A_68_P03584400	Bmp8b	1.487655
A_68_P13579167	Ltbp1	1.487613
A_68_P04403143	Sh2b2	1.487385
A_68_P02147557	Zfp661-Mrps5	1.487325
A_68_P13483867	Scube3	1.486997
A_68_P13680548	Rnf138	1.486838
A_68_P08718386	Inhbe	1.486021
A_68_P06143975	Gabra5	1.485237
A_68_P13162670	Clps	1.484857
A_68_P14180164	Fibp	1.484629
A_68_P01251036	Gfra4	1.484448
A_68_P03836687	4931406I20Rik	1.484441
A_68_P03262673	Inadl	1.484427
A_68_P02612003	Hist2h4	1.484379
A_68_P07396318	Asf1b	1.483937
A_68_P10130463	Pum2	1.483497
A_68_P14349190	Stip1	1.483414
A_68_P02032727	Arl6ip6	1.483347
A_68_P11078517	Hcn1	1.483242
A_68_P02221118	Lmx1b	1.48294
A_68_P09104779	Slfn9	1.482459
A_68_P03831176	Cyp2j6	1.482146

A_68_P06343665	Plekhg2	1.481991
A_68_P07506787	Cck	1.481753
A_68_P02419945	3110050N22Rik	1.48163
A_68_P00719162	Cnih3	1.481614
A_68_P04000868	Rnf207-Rpl22	1.48097
A_68_P14063148	Cxxc5	1.48086
A_68_P01661943	Phf21a	1.480679
A_68_P14256498	Entpd7	1.480529
A_68_P10347410	Pax9	1.480418
A_68_P09037026	Skp1a	1.480285
A_68_P09372157	Wnk4	1.480105
A_68_P13535873	Mapk13	1.479972
A_68_P12438489	Sfrs2ip	1.479931
A_68_P00327187	Lemd1	1.47945
A_68_P05447286	Rpn1	1.479346
A_68_P03474571	Lepr	1.479289
A_68_P01671416	Zfp313	1.478437
A_68_P08191867	Ncam1	1.478418
A_68_P02951670	Gbp6	1.478209
A_68_P06242540	6330503K22Rik	1.478056
A_68_P07262444	Aytl1	1.477903
A_68_P09726298	Coil	1.477663
A_68_P08860783	Olig3	1.477511
A_68_P14151854	Nudt8	1.477429
A_68_P02183976	Scand1	1.477376
A_68_P02465746	Ddit4l	1.477143
A_68_P02323477	Zfp687	1.477138
A_68_P08778842	Gja1	1.477079
A_68_P04090888	D4Wsu114e	1.476955
A_68_P09568749	Ube2b-Cdkl3	1.476486
A_68_P12309980	Aqp5	1.476209
A_68_P10618183	Zbed3	1.476159
A_68_P13486289	Clic5	1.475956
A_68_P03584400	Bmp8b	1.47563
A_68_P12802648	Runx1	1.475285

A_68_P11042649	Ocln	1.47515
A_68_P09096199	Mtmr4	1.474907
A_68_P03763133	Bach2	1.474846
A_68_P03305240	Foxo6	1.4748
A_68_P13656495	Snrpd1	1.474793
A_68_P02504418	Tpd52	1.474269
A_68_P13351558	Vav1	1.474169
A_68_P13565034	Stk38	1.474038
A_68_P07203069	Trmt1	1.474036
A_68_P08612636	Fabp7	1.473532
A_68_P04306515	Alkbh2-Ung	1.473504
A_68_P12202882	Grasp	1.473474
A_68_P06115755	Cacng3	1.473257
A_68_P05298275	Reep1	1.473199
A_68_P14953861	Rps6ka3	1.472322
A_68_P03963161	Pou3f1	1.47184
A_68_P15028309	Eif2s3x	1.47183
A_68_P03041117	Sfrp2	1.471285
A_68_P07512483	Dync1li1	1.4712
A_68_P01582652	E130319B15Rik	1.471146
A_68_P14597653	Acot9	1.470794
A_68_P10658758	Cartpt	1.470786
A_68_P00857131	Mapkapk2	1.47076
A_68_P12362731	Mfsd3	1.47069
A_68_P11132693	Ppp3cc	1.470398
A_68_P09123103	Pfn1	1.47027
A_68_P02740608	6330569M22Rik	1.470231
A_68_P07671679	Rbm5	1.469897
A_68_P09395677	1700007I06Rik-Stk10	1.469867
A_68_P02376979	Gipc2	1.468784
A_68_P09433951	0610009B22Rik	1.468724
A_68_P05252248	Chchd3	1.4687
A_68_P06163071	Abhd2	1.468599
A_68_P03912425	Aqp7	1.468333
A_68_P03831176	Cyp2j6	1.468287

A_68_P02744224	Nola1-Cfi	1.468108
A_68_P08917433	Cdc2a	1.468044
A_68_P07138739	Mcph1	1.467861
A_68_P15121373	Phf8	1.46757
A_68_P09732812	Pitpna	1.467463
A_68_P09816159	Bcl11b	1.467092
A_68_P07264824	Cbfb	1.467032
A_68_P09332948	Plekhm1	1.466902
A_68_P12886769	Ranbp1	1.466565
A_68_P09451674	Dnajc7-Nkiras2	1.466286
A_68_P05244196	ENSMUST00000083652.1:-3959	1.466144
A_68_P13596914	Gypc	1.46608
A_68_P01347361	Foxa2	1.465924
A_68_P05057770	Hk2	1.465192
A_68_P10419775	Chx10	1.465077
A_68_P08834118	Ddit4	1.465033
A_68_P00387540	Slc39a10	1.464297
A_68_P02033295	Ccdc3	1.464183
A_68_P07256672	Rwdd4a	1.463997
A_68_P07268697	Frg1	1.463972
A_68_P01251863	Notch1	1.463938
A_68_P05939019	A630091E08Rik	1.463783
A_68_P01572379	Ntng2	1.463728
A_68_P13972523	Fchsd1	1.463721
A_68_P01173314	Stx16	1.463388
A_68_P09358813	Limk2	1.463204
A_68_P08586130	Il20ra	1.463114
A_68_P14645447	Irs4	1.463063
A_68_P08372935	Btbd11	1.461531
A_68_P06386541	Brwd2	1.461252
A_68_P00540080	Dusp23	1.460988
A_68_P01584320	Dusp2	1.460961
A_68_P02396757	Aadacl1	1.4607
A_68_P06988313	Rspry1	1.460647
A_68_P09565488	Ap2b1	1.460173

A_68_P13841860	Zbtb7c	1.460139
A_68_P03747411	Tesk1	1.460107
A_68_P06349664	Igf1r	1.460034
A_68_P14134922	Mbp	1.460011
A_68_P07486322	Map2k1	1.45996
A_68_P07020688	Foxf1a	1.459893
A_68_P13426289	Ddr1	1.459511
A_68_P09309849	Eppb9	1.45908
A_68_P09065959	4933439F18Rik	1.458868
A_68_P06874380	Nfat5	1.458791
A_68_P07853625	Rrp9	1.458271
A_68_P07403941	Pik3r2	1.45795
A_68_P06841060	Pbx4	1.457646
A_68_P11291369	Bin3	1.457387
A_68_P03474931	Eif2c4	1.457305
A_68_P14333014	Tmem2	1.457149
A_68_P09838490	1810048J11Rik	1.456876
A_68_P07000614	Ing1	1.456741
A_68_P01524575	Hspa12b	1.456693
A_68_P01762558	BC061194	1.456685
A_68_P09441763	Myh10	1.456194
A_68_P09371327	Rtn4rl1	1.455881
A_68_P12864479	Popdc2	1.45568
A_68_P07554616	Rbms3	1.455667
A_68_P01695058	Jag1	1.455527
A_68_P14702906	Tro	1.455378
A_68_P09628615	Tmem88	1.454875
A_68_P09518146	Pcyt2	1.454804
A_68_P05281362	2610209M04Rik	1.454659
A_68_P06359553	Strn4	1.454646
A_68_P09387273	Fzd2	1.454451
A_68_P07950303	Sh3px3	1.4543
A_68_P09316344	Ywhae	1.453931
A_68_P00518747	Optc	1.453686
A_68_P03306890	Creb3	1.453588

A_68_P06322496	D430014M15	1.453439
A_68_P07565640	Tpbg	1.453208
A_68_P09200960	Lif	1.453182
A_68_P12108250	Hoxc10	1.452736
A_68_P01179906	D2Wsu81e	1.452526
A_68_P00905132	B3gnt7	1.452515
A_68_P09021294	Fmnl1	1.452287
A_68_P08629726	B530045E10Rik	1.452204
A_68_P03506214	Foxd2	1.452157
A_68_P05087618	Zxdc	1.452084
A_68_P12098583	Prph1	1.451806
A_68_P09666330	Gabarap	1.451399
A_68_P04919142	Mlxipl	1.451176
A_68_P09652627	BC029169	1.451089
A_68_P06925433	Pllp	1.450475
A_68_P07890285	Axud1	1.450327
A_68_P08866515	Adamtsl5	1.450234
A_68_P13981482	4930503L19Rik	1.450204
A_68_P06432034	Rasl2-9	1.450058
A_68_P06046622	Ppp2r2d	1.45001
A_68_P03505620	E2f2	1.449858
A_68_P05787946	Tsga14	1.449594
A_68_P13090912	Telo2	1.449589
A_68_P06658331	Zfp715	1.449267
A_68_P09107785	Tcap	1.448968
A_68_P04425904	Wasf3	1.448943
A_68_P01646909	BC061194	1.448936
A_68_P09681781	Timp2	1.44769
A_68_P09874616	1110008B24Rik	1.44752
A_68_P06323698	Fgf21-Fut1	1.447293
A_68_P12591564	Slc7a4	1.447064
A_68_P08556691	Aldh1l2	1.446744
A_68_P01588855	Frmd4a	1.446343
A_68_P00250137	Igsf4b	1.446318
A_68_P01979262	Sulf2	1.44623

A_68_P12651924	Ccdc54	1.446076
A_68_P02783108	Spata5	1.445896
A_68_P03421233	Dnaja1	1.445716
A_68_P05316094	Prdm5	1.445403
A_68_P04003641	Ccdc23	1.445205
A_68_P12303095	Wnt1	1.444849
A_68_P00343598	Tmeff2	1.444352
A_68_P09711728	Derl2-Mis12	1.444195
A_68_P15167347	Zrsr2	1.444035
A_68_P06364198	Zfp30	1.443779
A_68_P11855311	Slc25a32	1.443232
A_68_P03240726	2510006D16Rik	1.442897
A_68_P03338370	Dmrta2	1.442761
A_68_P12671996	Abcf3	1.442699
A_68_P07776192	Chst2	1.442656
A_68_P00743768	Hes6	1.44264
A_68_P02097433	Ptgis	1.442517
A_68_P04792804	Lfng	1.442189
A_68_P04328386	Hnrpd	1.442062
A_68_P01361685	Agpat2	1.441968
A_68_P08048266	6230427J02Rik	1.44193
A_68_P08708146	Lims1	1.441755
A_68_P02167804	Slc23a2	1.44171
A_68_P13846585	Dnajc18	1.441342
A_68_P13748929	Zadh2	1.441329
A_68_P07754822	Man2c1	1.441159
A_68_P01566039	Jag1	1.440769
A_68_P11039471	Irx4	1.440614
A_68_P07196635	Cnot7-Vps37a	1.440321
A_68_P04203215	Rpl9-Lias	1.440265
A_68_P05450007	Tra2a	1.440187
A_68_P02264567	C130021I20Rik	1.440076
A_68_P10122731	Gstz1	1.439784
A_68_P04711907	Arl6ip4	1.439497
A_68_P06848458	Dnajb1	1.439455

A_68_P04066415	2510039O18Rik	1.439313
A_68_P07559876	Armet	1.439208
A_68_P02058135	Mrg1	1.439179
A_68_P03056910	Golph4	1.43892
A_68_P01266404	Pax1	1.438803
A_68_P06431967	Siglecf	1.438757
A_68_P08934065	A_68_P08934065	1.43868
A_68_P06127842	Cebpg	1.438286
A_68_P09099238	Myo1d	1.43806
A_68_P14030415	Etf1	1.43792
A_68_P04824992	Gtf3c2	1.437902
A_68_P06776322	Mfap3l	1.437779
A_68_P09611938	Havcr2	1.437707
A_68_P11855904	Ly6d	1.43687
A_68_P04761495	Sdsl-Sds	1.436867
A_68_P07356122	Tk2-Cklf	1.436484
A_68_P05162900	Zfml	1.436461
A_68_P05979594	Lsr-Tmem162	1.436362
A_68_P03792681	Yrdc	1.436137
A_68_P03421224	Dnaja1	1.436122
A_68_P08089610	7-Sep	1.43594
A_68_P15016311	Ankrd58	1.435701
A_68_P01164203	Mcts2	1.435628
A_68_P05925982	Nlrp10-Eif3s5	1.435187
A_68_P04125064	Prom1	1.434219
A_68_P06314101	Myo7a	1.434164
A_68_P15042652	Suv39h1	1.433997
A_68_P01248802	Lrrc4c	1.433878
A_68_P14209567	Rin1	1.433811
A_68_P12441853	Adamts20	1.433802
A_68_P03465775	Cdc20	1.433773
A_68_P11752832	Wdhd1	1.433733
A_68_P08999972	Tbx21	1.433698
A_68_P04761495	Sdsl-Sds	1.433574
A_68_P02221102	Lmx1b	1.433491

A_68_P06113111	Foxa3	1.433447
A_68_P08161578	Acpp	1.43316
A_68_P09434018	Cntnap1	1.433152
A_68_P05689367	3321401G04Rik	1.433097
A_68_P10333896	Arl4a	1.43302
A_68_P11532193	Anxa8	1.432996
A_68_P11790055	Dzip1	1.432954
A_68_P06053964	Prkrir	1.432557
A_68_P03583518	Thrap3	1.432479
A_68_P07759159	Nktr	1.432346
A_68_P03026052	ENSMUST00000071298.4:28	1.432268
A_68_P02093470	U46068	1.432252
A_68_P04954999	Lrpap1	1.431922
A_68_P07424653	Ssbp4-Lrrc25	1.431787
A_68_P09617087	Flcn	1.431358
A_68_P03758436	Arid1a	1.431239
A_68_P07615452	Mst1r	1.431198
A_68_P12960661	Pla1a	1.431194
A_68_P02263070	Atf2	1.430926
A_68_P05324700	Zyx	1.430178
A_68_P08629268	Hkdc1	1.430064
A_68_P08139007	Ttk	1.430039
A_68_P04711907	Arl6ip4	1.429977
A_68_P03579116	1810054D07Rik	1.429959
A_68_P02577687	Sox2	1.429481
A_68_P07403939	Pik3r2	1.429434
A_68_P09376940	Slc16a3	1.429149
A_68_P08962443	B4galnt1	1.428792
A_68_P06293610	Luzp2	1.428669
A_68_P14768398	Apex2	1.428666
A_68_P07167615	Brd7	1.428506
A_68_P04339851	Ccdc92	1.428427
A_68_P08907273	A_68_P08907273	1.428392
A_68_P14251876	Sssca1	1.42819
A_68_P07615453	Mst1r	1.427854

A_68_P12834327	Dlgh1	1.427838
A_68_P12632289	Ece2	1.427734
A_68_P09002039	Phf23	1.427297
A_68_P12500589	Hira	1.427205
A_68_P12105096	Aaas	1.426705
A_68_P09614077	Phf12	1.426606
A_68_P01655397	BC050777	1.426414
A_68_P13875289	Rax	1.425902
A_68_P11166031	Wnt5a	1.425842
A_68_P10255666	Amn	1.425674
A_68_P05467387	Nol1	1.425566
A_68_P09736770	Gps1	1.425524
A_68_P09691873	Mdh1-AV249152	1.425494
A_68_P09248011	Tbx2	1.425297
A_68_P08176001	Myo1e	1.425015
A_68_P03776130	Rcc2	1.424913
A_68_P11558065	Dhrs4	1.424862
A_68_P09041858	A830091I15Rik	1.42481
A_68_P01405674	A730008L03Rik-BC061039	1.424667
A_68_P10386935	BC011209	1.424301
A_68_P12644912	2310061J03Rik	1.423442
A_68_P13483019	Hnrpll	1.423419
A_68_P12636175	Lsg1	1.423195
A_68_P08139005	Ttk	1.423065
A_68_P12225005	Ywhaz	1.422795
A_68_P07840841	Ccpg1	1.422322
A_68_P04005937	9630013D21Rik	1.422253
A_68_P10148637	Six1	1.422095
A_68_P09159717	Trp53i13-1300007F04Rik	1.421852
A_68_P00957898	Eif5b	1.421718
A_68_P12298037	Lynx1	1.421512
A_68_P07154228	1810029B16Rik	1.421163
A_68_P13293867	Ppp2r5d	1.421144
A_68_P01869763	2810030E01Rik-Mllt10	1.421027
A_68_P06262218	Six5	1.420177

A_68_P06142057	Ebf3	1.420161
A_68_P13178495	Rps18	1.420066
A_68_P01288747	Slc20a1	1.419961
A_68_P10859845	Prpf4b	1.419901
A_68_P11042659	Ocln	1.4198
A_68_P01655861	F730014I05Rik	1.419763
A_68_P10419771	Chx10	1.419644
A_68_P09548393	Aoc3	1.419406
A_68_P13267892	Tead3	1.419366
A_68_P14667558	Dock11	1.419253
A_68_P01531042	Nelf	1.419041
A_68_P04340627	Ung	1.418765
A_68_P11132881	Tkt	1.418734
A_68_P13930307	8030462N17Rik	1.418617
A_68_P01500368	Ube2e3	1.418301
A_68_P08250810	Matk	1.418298
A_68_P01256535	Dsn1	1.418174
A_68_P02836286	Narg1	1.418167
A_68_P06490200	2410015N17Rik	1.417919
A_68_P10179927	1110034A24Rik	1.417652
A_68_P06427461	BC017158	1.417265
A_68_P04862543	Sema3a	1.417223
A_68_P06652577	Shkbp1	1.417097
A_68_P08847295	Egr2	1.41628
A_68_P10532573	Ggps1	1.416277
A_68_P07727688	Mapk6	1.416144
A_68_P05599196	Kcna6	1.41612
A_68_P03785877	BC055111-Ttc22	1.415562
A_68_P11165637	Fut11	1.4155
A_68_P08846692	Spryd4	1.415497
A_68_P05383985	Xpc	1.41505
A_68_P04773450	Pdgfra	1.414745
A_68_P05177341	Egr4	1.414737
A_68_P08596256	Rps12	1.414491
A_68_P04267219	Emid2	1.414474

A_68_P08369612	Erbb3	1.414335
A_68_P01803899	Freq	1.414255
A_68_P11073717	Arsb	1.41418
A_68_P14648818	Mbtps2	1.414155
A_68_P01179072	Gata3	1.413519
A_68_P01471724	Lcn10	1.413126
A_68_P13558584	D630044L22Rik	1.412965
A_68_P08570676	Tmem26	1.41296
A_68_P14362696	Frat2	1.412927
A_68_P08695482	Thap2-Ccdc131	1.412775
A_68_P04993202	Kit	1.412386
A_68_P08933526	C78409	1.412292
A_68_P01253607	Hoxd1	1.412185
A_68_P08840694	Arid3a	1.41205
A_68_P06690927	2310079N02Rik	1.411868
A_68_P11148928	Anxa11	1.411635
A_68_P04112279	Gpr30	1.411359
A_68_P11889047	Plcxd3	1.411342
A_68_P13035016	B3gnt5	1.41126
A_68_P04331827	Cdc7	1.411212
A_68_P07544112	Arpp21	1.41066
A_68_P14064072	Reep5	1.410302
A_68_P12480253	Cldnd1	1.410216
A_68_P02745695	Shc1	1.410115
A_68_P01768509	Stk35	1.410081
A_68_P00197320	Serpine2	1.409812
A_68_P13390073	Crim1	1.409611
A_68_P11935737	Syngr1	1.409608
A_68_P01606729	Entpd2	1.409584
A_68_P02127773	2310047O13Rik	1.409435
A_68_P11438500	D14Ertd449e-C330003B14Rik	1.409267
A_68_P06506013	Ruvbl2	1.409265
A_68_P04225894	Slc10a6	1.409035
A_68_P09739582	Nfe2l1	1.40894
A_68_P09099281	A830053O21Rik	1.408666

A_68_P03619385	Abca1	1.40855
A_68_P05106728	Atg7	1.408502
A_68_P01164453	Slc24a5	1.408495
A_68_P04573997	Akap9	1.408491
A_68_P04926215	Fzd10	1.408413
A_68_P04548189	Mvk	1.408201
A_68_P07321200	Terf2ip	1.408189
A_68_P13667994	Txnl4	1.408137
A_68_P08704016	Perp	1.408008
A_68_P04993591	C130026L21Rik	1.407896
A_68_P12755351	Qtrtd1	1.407895
A_68_P14223579	Lgals12-Hrasls5	1.407721
A_68_P01887581	Shc4	1.407557
A_68_P06087354	Pnpla2	1.407456
A_68_P08769438	Col18a1	1.407443
A_68_P01312676	D2Ertd391e	1.407283
A_68_P08484085	1810043G02Rik	1.407145
A_68_P01268002	Lrp4	1.406906
A_68_P07866293	Angptl6	1.406897
A_68_P14474026	Gda	1.40678
A_68_P02611641	ENSMUST00000071298.4:-360	1.40673
A_68_P04827265	6530401C20Rik	1.406628
A_68_P07312071	Sf4	1.406581
A_68_P09752136	Laptm4a	1.406447
A_68_P11779116	Pou4f1	1.4062
A_68_P00672918	Arid5a	1.40602
A_68_P12087654	Cyc1	1.405795
A_68_P12599801	Ccdc58	1.405643
A_68_P01159756	Hrh3	1.405563
A_68_P13728786	Dsc2	1.405461
A_68_P06236830	Tmem9b	1.40538
A_68_P07400832	Syce2	1.405056
A_68_P14800321	Dusp9	1.405003
A_68_P05344579	Tnfrsf1a	1.404816
A_68_P06336171	Kcnc1	1.404805

A_68_P11347693	Bmpr1a	1.404725
A_68_P08070275	Wdr48	1.404703
A_68_P09521152	Ogdh	1.404657
A_68_P09052341	Vps25	1.40439
A_68_P13117097	Bak1	1.404249
A_68_P01065374	Armc9	1.404223
A_68_P08145910	6230410P16Rik	1.404188
A_68_P03647537	Slc35d1	1.404044
A_68_P07200483	Ier2	1.403966
A_68_P07746395	Pik3cb	1.403855
A_68_P08169692	Mtmr2	1.403774
A_68_P13166533	Ddah2	1.403479
A_68_P05117228	Anxa4	1.403256
A_68_P05419220	Cdca3	1.402922
A_68_P02246977	BC005624	1.402907
A_68_P13535592	Kcng3	1.402734
A_68_P01762541	Sall4	1.402549
A_68_P05984762	Fah	1.402305
A_68_P10182243	Esrrb	1.402228
A_68_P03211665	5830433M19Rik	1.401909
A_68_P11815323	Jub	1.401794
A_68_P14457991	Prkg1	1.401775
A_68_P09287103	D130058I21Rik	1.401518
A_68_P07336121	Mvd-9330133O14Rik	1.401257
A_68_P09038460	Mink1	1.400997
A_68_P13136058	Rps2	1.400933
A_68_P08395271	Smarcc2	1.400637
A_68_P06596029	Ppp5c	1.400623
A_68_P00947977	Lpgat1	1.400579
A_68_P07243848	Ptger1	1.400369
A_68_P09368962	Sox9	1.400111
A_68_P00575084	Ivns1abp	1.400032
A_68_P04319123	Iqce	1.399843
A_68_P07183169	Nek5	1.399702
A_68_P14350019	Smc5	1.399613

A_68_P13118031	H2-Ke6	1.399464
A_68_P01966879	Freq	1.399423
A_68_P14345132	Pcgf5	1.399081
A_68_P01238229	Pacsin3	1.398803
A_68_P05991307	Pde3b	1.398775
A_68_P02545177	Cttnbp2nl	1.398716
A_68_P14323852	Psd	1.39869
A_68_P14070108	Sap130	1.398543
A_68_P05993284	Nr1h2	1.398225
A_68_P00258736	ENSMUST00000066061.3:55	1.398031
A_68_P06431492	Lmo1	1.397984
A_68_P01299919	Hoxd3	1.3979
A_68_P12600289	Pcyt1a	1.397789
A_68_P13149887	Zfp598	1.397554
A_68_P06939152	E2f4	1.397251
A_68_P11746907	Zfp503	1.397196
A_68_P05985286	Sergef	1.39704
A_68_P12361670	Zfp385	1.396871
A_68_P14996501	Bgn	1.396756
A_68_P01894888	Itpka	1.396384
A_68_P04148393	Tmed5	1.395791
A_68_P01288371	Fbn1	1.395572
A_68_P09486902	Stc2	1.395364
A_68_P07046584	Asah1	1.395332
A_68_P06621037	Sec23ip	1.394899
A_68_P13472939	Lama1	1.394849
A_68_P01309646	Gzf1	1.394803
A_68_P03926231	Necap2	1.394675
A_68_P11556763	A430057M04Rik	1.394618
A_68_P10775535	Esm1	1.394554
A_68_P05104718	Wnt2	1.394544
A_68_P13612986	Epc1	1.394476
A_68_P05404915	Prr15	1.393836
A_68_P05541771	Fgf23	1.393465
A_68_P12276707	Tmem74	1.393354

A_68_P04131879	Tmem142a	1.39334
A_68_P00658070	Gbx2	1.39303
A_68_P01970209	Fjx1	1.392996
A_68_P03326897	Gpr153	1.392775
A_68_P11906856	Ccnt1	1.392682
A_68_P06219796	A930008G19Rik	1.392584
A_68_P03522571	Nbl1	1.392393
A_68_P02259738	Fbxo3	1.392196
A_68_P06448743	Clptm1	1.392008
A_68_P00366679	Stat4	1.391931
A_68_P02071364	Wisp2	1.391697
A_68_P13088188	2600005C20Rik	1.391603
A_68_P05915386	1700008P20Rik	1.39133
A_68_P09136314	Baiap2	1.391135
A_68_P01251024	Gfra4	1.390863
A_68_P06444721	Snrpa	1.390819
A_68_P12432039	Galnt6	1.390555
A_68_P08263375	Kif5a-Dctn2	1.390248
A_68_P10959245	Tcfap2a	1.389782
A_68_P13355809	Grm4	1.38968
A_68_P13218398	Rfx2	1.389655
A_68_P14143571	Nkx2-3	1.389652
A_68_P09575640	Ddx52	1.389378
A_68_P04225894	Slc10a6	1.389107
A_68_P06506913	Trim3	1.389077
A_68_P01970205	Fjx1	1.389068
A_68_P12384339	Faim2	1.388914
A_68_P04027659	Zfp46	1.388822
A_68_P13518978	Satb1	1.388787
A_68_P00229523	Rcor3	1.388748
A_68_P09530799	H3f3b-Zc3h5	1.38853
A_68_P03606820	Psmb2	1.388438
A_68_P05290332	Gpr85	1.388344
A_68_P09307716	Doc2b	1.388135
A_68_P13106791	Tulp1	1.38813

A_68_P04979692	9430057O19Rik	1.388107
A_68_P09283842	Dnajc7	1.388086
A_68_P14437291	5730596K20Rik	1.388059
A_68_P12756270	Pigz	1.387949
A_68_P12599777	2310056P07Rik	1.387905
A_68_P10336568	Clec14a	1.38777
A_68_P06534995	Cdkn1c	1.387423
A_68_P04148393	Tmed5	1.38708
A_68_P03843357	Ppih	1.387038
A_68_P05899443	Rgma	1.387037
A_68_P03730091	Ube2j2	1.387025
A_68_P09825102	Spnb1	1.386913
A_68_P09034561	Gas2l1	1.3869
A_68_P12603717	Sim2	1.386852
A_68_P11500059	Mrpl52	1.386783
A_68_P13265576	Jmjd2b	1.386546
A_68_P12109009	Rapgef3	1.3864
A_68_P04267219	Emid2	1.386388
A_68_P11899315	BC024139	1.386175
A_68_P02983160	Pcdh10	1.38612
A_68_P08756565	Col13a1	1.385371
A_68_P09944887	Vsnl1	1.385207
A_68_P04773930	Aym1	1.385083
A_68_P08396864	Crsp3	1.384899
A_68_P02482328	Vps72	1.384805
A_68_P09221420	1110014K08Rik	1.38463
A_68_P01655095	Spata2	1.38454
A_68_P05297003	Copg2	1.384242
A_68_P09056902	1700052K11Rik	1.383926
A_68_P01647847	Atp5e	1.383894
A_68_P01891391	Pdia3	1.383835
A_68_P00173418	Atic	1.383755
A_68_P03554177	Mycl1	1.383272
A_68_P04947548	Uchl1	1.383216
A_68_P03990834	Ube2r2	1.383192

A_68_P04553689	Cenpc1-AI586015	1.383103
A_68_P02598927	Mynn	1.383037
A_68_P04090888	D4Wsu114e	1.382935
A_68_P01643181	Zswim1	1.382825
A_68_P05148540	Nagk	1.382579
A_68_P04062160	Ythdf2	1.382375
A_68_P06316193	Ebf3	1.382308
A_68_P09866881	Rcor1	1.382291
A_68_P03372374	Zswim5	1.382106
A_68_P06614084	Nr2f2	1.381956
A_68_P10538246	Cetn3	1.381864
A_68_P10435878	Matn3	1.381514
A_68_P14396329	Mxi1	1.381129
A_68_P05297197	Tmem111	1.380924
A_68_P03003493	Kcnn3	1.380836
A_68_P03296780	Klhl9	1.380505
A_68_P03728571	B230396O12Rik	1.380472
A_68_P06035662	U2af1l4	1.380132
A_68_P01531594	Mdk	1.380085
A_68_P09618735	Aldoc	1.37995
A_68_P04736301	Plod3	1.379897
A_68_P06837702	Thap1	1.379833
A_68_P09641175	Slc16a11	1.379772
A_68_P12923443	Pmm2	1.37977
A_68_P07140101	2310036O22Rik	1.379761
A_68_P13874169	Rps14	1.379688
A_68_P12061444	Tob2	1.379532
A_68_P09065614	ENSMUST00000039627.4:-4128	1.379415
A_68_P10492574	Ippk	1.379165
A_68_P09358750	Casc3	1.37902
A_68_P02650789	Setd7	1.379015
A_68_P03980046	Zdhhc18	1.37898
A_68_P11790058	Dzip1	1.378915
A_68_P10785709	1300014I06Rik	1.378813
A_68_P05072118	4631427C17Rik	1.378789

A_68_P11456818	Slc25a37	1.378742
A_68_P06524443	Mtg1	1.378636
A_68_P14233015	Ccdc85b-Fibp	1.378482
A_68_P13844477	Fchsd1	1.378292
A_68_P01293034	4833422F24Rik	1.37812
A_68_P08711144	Nnp1-Cstb	1.37781
A_68_P11632181	Jph4	1.377748
A_68_P02396754	Aadacl1	1.377644
A_68_P09069020	Arl5c	1.37755
A_68_P07045118	4930481F22Rik	1.377452
A_68_P13678357	Atp5a1	1.377243
A_68_P04502460	Rnf6	1.377125
A_68_P11226193	Hs6st3	1.376937
A_68_P03530044	Cpt2	1.376568
A_68_P11374615	Grid1	1.376491
A_68_P11209114	Camk2g	1.376363
A_68_P13426419	Slc22a3	1.376332
A_68_P07822223	Nr2e3	1.376269
A_68_P02536571	Slc16a1	1.376226
A_68_P06654367	Plekhg2	1.376085
A_68_P14392026	Pten	1.376069
A_68_P14231962	Cnnm2	1.375977
A_68_P10857129	Cdc14b	1.375892
A_68_P01338331	Hoxd9	1.375892
A_68_P04909159	D5Wsu178e	1.375863
A_68_P05112722	5830446M03Rik	1.375693
A_68_P04896136	Htr5a	1.375403
A_68_P03188324	Mtx1	1.375399
A_68_P07804000	Ccpg1	1.375399
A_68_P04360651	Ache	1.375347
A_68_P05725849	Hoxa1	1.37527
A_68_P13506305	Ccnd3	1.374951
A_68_P06943436	Irx6	1.374926
A_68_P10284063	Tgfb3-1700019E19Rik	1.374346
A_68_P08300526	Mon2	1.374212

A_68_P09214099	Plekhh3	1.374172
A_68_P08229110	Tnfaip3	1.374171
A_68_P12451534	Atf7	1.374133
A_68_P08714775	Tcba1	1.373898
A_68_P08936079	A_68_P08936079	1.373831
A_68_P04711913	Arl6ip4	1.373688
A_68_P02843046	Prkci	1.373503
A_68_P08288178	Tmtc2	1.373478
A_68_P11507565	Pcdh17	1.373314
A_68_P09393918	Nsf	1.373122
A_68_P06620448	5430432N15Rik	1.373091
A_68_P14729681	Utp14a	1.373044
A_68_P09644084	Srebf1	1.372953
A_68_P04071366	Plaa	1.372942
A_68_P10419758	Chx10	1.372918
A_68_P14930315	Phka2	1.372906
A_68_P09439572	Bcl6b	1.372588
A_68_P03214207	Slc9a1	1.372505
A_68_P03139924	Zbtb7b	1.372303
A_68_P00400070	Kif1a	1.372232
A_68_P12079965	2810451A06Rik	1.372009
A_68_P07098077	Helt	1.37197
A_68_P09018398	Gprc5c	1.371571
A_68_P11173734	Efs-Il25	1.371543
A_68_P01542525	Ghrh	1.371424
A_68_P01305510	Ncoa5	1.371345
A_68_P09304808	Stac2	1.371162
A_68_P12059852	Smarcd1	1.37113
A_68_P01897769	F730014I05Rik	1.370526
A_68_P03402252	Jmjd2a	1.370393
A_68_P01522585	Pxmp4	1.370355
A_68_P03560289	Elavl2	1.370244
A_68_P01776405	Olfml2a	1.370219
A_68_P11948519	Aqp5	1.369786
A_68_P09731018	Myo1c	1.369689

A_68_P01296062	Gm757	1.369492
A_68_P09128453	Cbx4	1.369373
A_68_P04567450	Klf3	1.369206
A_68_P07849832	Plod2	1.369079
A_68_P02824933	Acp6	1.368999
A_68_P11937879	BC030396	1.368711
A_68_P14473024	Cabp2	1.368553
A_68_P14029021	Galr1	1.368517
A_68_P09288447	Pycr1	1.368094
A_68_P14040519	Pcdhgb5	1.368076
A_68_P04528236	Trim50	1.36805
A_68_P11507594	Pcdh17	1.368004
A_68_P04306525	Zan	1.367971
A_68_P14329940	Emx2	1.367887
A_68_P08034953	Plod2	1.367712
A_68_P14977649	Ube2a	1.367694
A_68_P06657211	Zfp629	1.367464
A_68_P10509192	Pitx1	1.367427
A_68_P09926608	BC048191:-5384	1.367412
A_68_P08937904	Eef2	1.367389
A_68_P05308436	Sec61a1	1.367066
A_68_P10681710	Zfp367	1.366745
A_68_P12544071	Dirc2	1.366414
A_68_P08850557	Tpd52l1	1.366224
A_68_P08108310	Dusp7	1.366104
A_68_P12461149	Krt2-25	1.365982
A_68_P01545930	Mrpl41-Pnpla7	1.36588
A_68_P04553689	Cenpc1-AI586015	1.365725
A_68_P03553494	Espn	1.365602
A_68_P03544448	Gnl2	1.365596
A_68_P03468979	Prdm16	1.365587
A_68_P09559324	Pdlim4	1.365574
A_68_P13406566	Tnfrsf21	1.365562
A_68_P04945505	Apbb2	1.365452
A_68_P09562188	4930412M03Rik	1.365373

A_68_P06122748	Car11	1.365326
A_68_P14204208	Cox8a	1.36519
A_68_P15210311	Sox3	1.365151
A_68_P09652799	Syngr2	1.365124
A_68_P07955703	5730536A07Rik	1.365076
A_68_P08710844	Trpm2-1810043G02Rik	1.364919
A_68_P09367780	Pfn1-Eno3	1.364641
A_68_P07533500	Xrn1	1.364598
A_68_P07800229	Tspan3	1.364446
A_68_P06621039	Sec23ip	1.364314
A_68_P00513237	Stk17b	1.364225
A_68_P11602112	Dnajc15	1.364046
A_68_P05987579	Rnf121	1.363856
A_68_P07889708	Mon1a	1.363671
A_68_P05584898	Bcat1	1.363543
A_68_P10254191	Egln3	1.363475
A_68_P06381447	Rasl2-9	1.363394
A_68_P10248353	Kcnk13	1.36297
A_68_P04345686	2410025L10Rik	1.362961
A_68_P04137812	Epo	1.362834
A_68_P06930178	B3gnt3	1.36283
A_68_P09207680	Utp18	1.362568
A_68_P06262927	Trim68	1.36233
A_68_P06843511	2810428I15Rik	1.362321
A_68_P10548283	Muted	1.362311
A_68_P09506627	Serpinf2	1.361829
A_68_P07319297	Rrad	1.361657
A_68_P08257942	BC030440	1.361606
A_68_P09086358	Gas2l1	1.361406
A_68_P13472163	Six2	1.361346
A_68_P07373423	A_68_P07373423	1.361299
A_68_P02991822	Tspan2	1.361271
A_68_P03975839	Prdm16	1.361225
A_68_P09431553	St6galnac2	1.36106
A_68_P14165549	Sh3pxd2a	1.36092

A_68_P03087427	Pklr	1.360755
A_68_P06189872	Mesp1	1.360117
A_68_P06767594	Fnta	1.359884
A_68_P08782489	Rkhd1	1.359853
A_68_P09330589	Rasd1	1.359832
A_68_P07960242	Angptl6-Ppan	1.359635
A_68_P09343918	BC030477	1.359591
A_68_P06275562	Uqcrc2	1.359574
A_68_P06957976	Dctd	1.359437
A_68_P12292478	Card10	1.359368
A_68_P09621168	Ankrd43	1.359124
A_68_P07023164	Sin3b	1.359102
A_68_P08049234	Copb2	1.3591
A_68_P00344185	Sox17	1.359051
A_68_P02752639	Hist2h2be	1.358796
A_68_P03926231	Necap2	1.358794
A_68_P08241070	D10Ertd610e	1.35868
A_68_P02208449	Pdk1	1.358569
A_68_P07818869	Yap1	1.35845
A_68_P04715249	Tbx5	1.358255
A_68_P04573997	Akap9	1.358183
A_68_P04959693	Vps33a	1.358157
A_68_P01628470	Rassf2	1.35812
A_68_P07312427	Dynlrb2	1.357711
A_68_P12988162	Slc12a8	1.35762
A_68_P05744316	Pde3a	1.357319
A_68_P03447018	Cd72	1.357295
A_68_P05984752	Fah	1.357283
A_68_P09271419	Aatf	1.35714
A_68_P08935045	2310011J03Rik	1.35709
A_68_P11697259	Dis3	1.357089
A_68_P15210082	Praf2	1.357039
A_68_P09476913	Shbg	1.357021
A_68_P07308039	2410018C20Rik	1.35682
A_68_P00583491	Irf6	1.356818

A_68_P10325476	Mycn	1.356815
A_68_P09535305	Dhx33	1.356791
A_68_P10205334	Yy1	1.356638
A_68_P03107450	Creb3l4-Slc39a1	1.356226
A_68_P00395221	5330401P04Rik	1.355914
A_68_P01175967	Dgkz	1.355853
A_68_P04116773	Thrap2	1.3557
A_68_P07412696	Edc4	1.355528
A_68_P07590783	Traip	1.355514
A_68_P06787361	Tusc3	1.355374
A_68_P05288271	Ppih	1.355363
A_68_P00578461	Zc3h11a	1.354905
A_68_P15038878	Apln	1.354848
A_68_P13272683	Epas1	1.354825
A_68_P04071366	Plaa	1.354794
A_68_P13270003	Zfp598	1.354616
A_68_P01648426	3110001A13Rik	1.354465
A_68_P08628561	Lgr5	1.354383
A_68_P08526428	Ddit3	1.354312
A_68_P03346618	Id3	1.354262
A_68_P08151133	Ctnnb1	1.354251
A_68_P03041163	Sfrp2	1.354188
A_68_P00850156	Lhx9	1.354146
A_68_P01982873	BC066135	1.354133
A_68_P02650791	Setd7	1.35405
A_68_P01996978	2310042G06Rik	1.353975
A_68_P10900081	Mier3	1.353863
A_68_P09530012	1110014K08Rik	1.353795
A_68_P08873707	Rpl41	1.353531
A_68_P08215262	Cnn2	1.353368
A_68_P03629045	Lin28	1.353088
A_68_P13359198	Lrpprc	1.352684
A_68_P02942719	D3Ucla1	1.352676
A_68_P13733932	Map3k8	1.352645
A_68_P11042513	4930544M13Rik	1.352578

A_68_P11979317	Tssk5	1.352506
A_68_P12380453	Matn2	1.35234
A_68_P12267844	Krt18	1.352302
A_68_P04852339	A230097K15Rik	1.352224
A_68_P05286103	Wnk1	1.351995
A_68_P03261704	Dctn3	1.351677
A_68_P15121552	Morc4	1.351601
A_68_P05362381	Camk1	1.351543
A_68_P04157606	Xrcc2	1.351487
A_68_P12816958	Ephb3	1.351482
A_68_P07561567	Entpd3	1.351388
A_68_P14209540	Rin1	1.351306
A_68_P10141170	Prkch	1.351304
A_68_P06740718	2400003C14Rik	1.35104
A_68_P03876320	Zfp691	1.350926
A_68_P12183195	Nfe2	1.350856
A_68_P08902269	Slc35d3	1.350802
A_68_P01571536	Aurka-Cstf1	1.350651
A_68_P03785877	BC055111-Ttc22	1.350646
A_68_P02133880	Plxdc2	1.350433
A_68_P08642932	Smarcc2	1.350404
A_68_P08168098	Ankrd25	1.350332
A_68_P04667157	Snx17	1.350214
A_68_P14751401	Usp11	1.350082
A_68_P01833937	Pax6	1.350024
A_68_P03349816	Hkr3	1.350021
A_68_P01326999	Ndrg3	1.349854
A_68_P09547541	BC017647	1.349829
A_68_P14345668	Slc22a8	1.349805
A_68_P04138127	Fzd10	1.349726
A_68_P07581372	Zmynd10	1.34956
A_68_P11281790	2600011E07Rik	1.349521
A_68_P01880312	Abtb2	1.349509
A_68_P08065769	Fbxl12	1.349498
A_68_P14204146	Hspa12a	1.349416

A_68_P14154165	Ina	1.349086
A_68_P11888728	Poldip3	1.348888
A_68_P02189747	Hrh3	1.348859
A_68_P09491191	D130058I21Rik	1.348836
A_68_P12229235	Rrm2b	1.348748
A_68_P13614347	B4galt6	1.348725
A_68_P07513276	Pcolce2	1.348558
A_68_P14251879	Mtvr2	1.348314
A_68_P12246601	Myo10	1.347759
A_68_P02376968	Gipc2	1.34724
A_68_P03413661	Padi4	1.347227
A_68_P13867090	Pard6g	1.347198
A_68_P10256825	Erh	1.347184
A_68_P03581861	St3gal3	1.346935
A_68_P02042838	Bub1	1.346842
A_68_P09349428	Pscd1	1.34676
A_68_P06975475	Gpr124	1.346702
A_68_P13450829	Tapbp	1.346546
A_68_P13760300	Cxxc1	1.346271
A_68_P02034225	Itga6	1.34623
A_68_P01668369	Slc35c1	1.346227
A_68_P05743578	Skap2	1.346152
A_68_P00932141	Epha4	1.345694
A_68_P03666605	Astn2	1.345672
A_68_P12569215	Dscr3	1.345575
A_68_P08358935	Lace1	1.34541
A_68_P12809557	B4galt4	1.345325
A_68_P11067498	Phactr1	1.345283
A_68_P12233069	Mb	1.344955
A_68_P06519012	AI480556-Rras	1.344768
A_68_P07281036	Gse1	1.344759
A_68_P02773136	5730470L24Rik	1.344748
A_68_P04780549	Scarb2	1.344603
A_68_P15158572	Ofd1	1.3446
A_68_P09262940	BC025575	1.344398

A_68_P09132227	Cacng4	1.344268
A_68_P06926957	Nob1	1.344266
A_68_P14674510	Slc6a8	1.344259
A_68_P02018771	Snap25	1.343965
A_68_P11526178	A_68_P11526178	1.343918
A_68_P14452830	Tle4	1.343911
A_68_P04442361	Msi1	1.343595
A_68_P06269772	Fzd4	1.343371
A_68_P11740899	Bmp4	1.343306
A_68_P03814073	Rbm35a	1.343292
A_68_P09555116	E130012A19Rik	1.343019
A_68_P13425847	Klc4-Mrpl2	1.342837
A_68_P06410140	Zfp710	1.342657
A_68_P07976988	4733401H18Rik-Dalrd3	1.342376
A_68_P04803354	Arpc1b	1.34237
A_68_P03126835	Shox2	1.342282
A_68_P00022370	Zfp451	1.342006
A_68_P15105004	Rbmx	1.341812
A_68_P01463378	Psmf1	1.341786
A_68_P09885031	Btbd7	1.341409
A_68_P14420132	Ldb1	1.341373
A_68_P08817684	Sgk	1.34136
A_68_P00225465	Lamc2	1.341294
A_68_P03547730	Cc2d1b	1.341291
A_68_P04946573	Fbxl5	1.341015
A_68_P04542655	Cdk8	1.340796
A_68_P02120879	3230401D17Rik	1.34075
A_68_P14299210	Foxb2	1.340422
A_68_P03226841	Pla2g2c	1.340313
A_68_P04153080	1200011O22Rik	1.3403
A_68_P05187965	Podxl	1.340224
A_68_P11260838	Bmpr1a	1.340096
A_68_P09116447	Tmem132e	1.340034
A_68_P06909746	Nup133	1.340005
A_68_P09390157	4933400C05Rik	1.339906

A_68_P09703832	Epn3	1.33988
A_68_P13106317	Tgif	1.339856
A_68_P07037040	Upf1	1.339657
A_68_P12240204	Rangap1	1.339423
A_68_P00542898	Nppc	1.339229
A_68_P14212477	Cpt1a	1.338795
A_68_P00859453	Lhx9	1.33871
A_68_P09485925	Rnf135	1.338685
A_68_P14842575	Ube1x	1.338503
A_68_P02492290	Mcl1	1.338463
A_68_P02142088	3230401D17Rik	1.338314
A_68_P08883003	Ppa1	1.338308
A_68_P12608100	Lztr1	1.338218
A_68_P12815673	Dcbld2	1.33818
A_68_P04481832	9530056K15Rik	1.337965
A_68_P06897654	Slc35e1	1.337857
A_68_P02293891	Wdr3-Gdap2	1.337747
A_68_P06292783	Sae1	1.337643
A_68_P09207933	Sf3a1	1.337435
A_68_P01506207	Ndor1-C730025P13Rik	1.337302
A_68_P10419767	Chx10	1.337082
A_68_P02123487	Manbal	1.336735
A_68_P14467080	1810009A15Rik	1.336308
A_68_P14295260	Foxb2	1.336267
A_68_P11575462	Xpo4	1.33622
A_68_P05427993	Magi1	1.336187
A_68_P13172213	Ccnf	1.336111
A_68_P14229760	Npas4	1.336014
A_68_P10050034	Trmt5	1.336002
A_68_P10902235	Pitx1	1.335996
A_68_P13429542	Pbx2	1.335976
A_68_P08149916	Nt5e	1.335937
A_68_P09457380	Adamts2	1.335932
A_68_P08108313	Dusp7	1.335891
A_68_P00163908	Twist2	1.335701

A_68_P07518222	BC025114:843	1.335627
A_68_P12635588	Ehhadh	1.335376
A_68_P00931378	En1	1.335294
A_68_P05377564	Tacstd2	1.335229
A_68_P03350592	Txndc12	1.335144
A_68_P04562151	Hsd17b11	1.33507
A_68_P04137562	Nupl2	1.335036
A_68_P12862382	ORF28	1.334956
A_68_P01221321	0610011L14Rik	1.334937
A_68_P04203215	Rpl9-Lias	1.334619
A_68_P07349292	BC025816	1.334522
A_68_P13293615	Gbl	1.33426
A_68_P08995123	Nlgn2-1810027O10Rik	1.334214
A_68_P01337579	Dido1	1.334109
A_68_P07697198	Acaa1a	1.333893
A_68_P08197986	Snapc5	1.333865
A_68_P14804684	Zic3	1.333853
A_68_P09247554	Nf2	1.333698
A_68_P10666960	Jarid2	1.333693
A_68_P06307711	Med25	1.333618
A_68_P01092822	Fmo1	1.333542
A_68_P13388147	Bat5	1.333336
A_68_P03937868	Srrm1	1.333314
A_68_P08656295	Phyhipl	1.333274
A_68_P05419190	Smarcad1	1.333171
A_68_P14428140	Tlx1	1.333111
A_68_P11739540	Gjb2	1.333053
A_68_P02073678	2410042D21Rik	1.332855
A_68_P01018072	Ahctf1	1.332614
A_68_P05552654	Gabarapl1	1.332503
A_68_P05111640	Trim24	1.332475
A_68_P06706577	Irx5	1.332207
A_68_P02151088	Bpi	1.332199
A_68_P05917961	Syt3	1.33197
A_68_P07695540	2610101N10Rik	1.331769

A_68_P13081532	Hmga1	1.331669
A_68_P07036341	Foxl1	1.331611
A_68_P04090886	D4Wsu114e-Fv1	1.331582
A_68_P07723906	Sema3f	1.331402
A_68_P08297371	Dip3b	1.331343
A_68_P11213240	Top2b	1.331251
A_68_P04316294	Mapre3	1.331193
A_68_P05814338	Pdzrn3	1.331166
A_68_P09178147	Osm	1.331118
A_68_P06572786	1500002O20Rik	1.331002
A_68_P02072158	Hspa5	1.330972
A_68_P14560505	Chst7	1.330844
A_68_P13747694	Rnuxa	1.330572
A_68_P03230057	Ubiad1-Frap1	1.330502
A_68_P14139346	Sall3	1.330424
A_68_P09084216	Aebp1	1.330364
A_68_P10219280	Plekhg3	1.330362

Supplementary Table 2: 437 genes that were hypermethylated in KO offspring compared with those in N offspring both on d16 and d100

1110008B24Rik
1600002H07Rik
1810009A15Rik
1810043G02Rik
1810054D07Rik
2310010M24Rik
2310011J03Rik
2400001E08Rik
2400006H24Rik
2410166I05Rik
2600011E07Rik
2610042O14Rik
2810030E01Rik-Mllt10
2810432D09Rik
2810432D09Rik
2810451A06Rik
4733401H18Rik-Dalrd3
4833422F24Rik
4930418G15Rik
5033414K04Rik
5330439J01Rik
5330439J01Rik
5730419I09Rik
5730470L24Rik
5730596B20Rik
5730596K20Rik
6230410P16Rik
6530401C20Rik
9130019P16Rik
A_68_P08936079
A230097K15Rik
Acaa1a
Acat3

Acp5
Actg1
Acvr1
Adam12
Adam12
Adcy9
Agpat2
AI450540
Akap12
Akr1a4
Aldh3a2
Ankrd43
Ap2a1
Arid1a
Arid1a
Arid1a
Arid2
Arid5b
Arl5c
Arl5c
Armet
Armet
Arpc1b
Atoh8
Atp5g2
Axud1
Axud1
B3gnt7
B3gnt7
B830017H08Rik
Baiap2
Bat5
BC018371
BC021891
BC024139

BC030396
BC030396
BC030440
BC030440
BC031181
BC048191:-5384
BC066135
Birc3
Brd7
Bst2
C230093N12Rik
Camk2g
Card10
Cartpt
Ccbl1
Ccdc3
Ccdc84
Cck
Ccnd3
Ccnf
Cdc20
Cdc20
Cdc7
Cdk8
Cdkn1c
Chd1
Chrd
Clta
Clta
Cnnm2
Cops7a
Cpeb1
Cpn1
Cpt1a
Ctdsp1

Cttnbp2nl
Cxcr4
Cybasc3
Cyld
D11Ertd636e
D130058I21Rik
D130058I21Rik
D3Ucla1
D5Wsu178e
D630029K05Rik
D630029K05Rik
Ddit4
Derl1
Dgkz
Dhrs4
Dmrt2
Dmrt2
Doc2b
Dock6
Dpf3
Dpysl3
Dusp1
Dusp6
Dzip1
Dzip1
E430028B21Rik
Efna1
EG328479
Egln3
Egr2
Egr3
Egr3
Ehd3
Ehhadh
Ela2

Elovl1
Elovl1
Emid2
Emid2
En1
En1
En2
En2
Epha4
Epha4
Esrrb
Etf1
Etv5
Etv5
F730014I05Rik
F730014I05Rik
Faim2
Fchsd1
Fchsd1
Fgf17
Fgf21
Fgf21-Fut1
Fkbp11
Fmnl1
Fmo1
Foxa2
Foxb2
Foxb2
Foxb2
Foxo3a
Foxo6
Fscn1
Fscn1
Fzd5
Fzd5

Gadd45g
Gas2l1
Gas2l1
Gata3
Gata3
Gata3
Gata5
Gfra4
Gfra4
Gja3
Gjb2
Gpbp1
Gprc5c
Gtf2h4
Gzf1
Hars2
Hhat
Hk2
Hkdc1
Hkr3
Hmga1
Hnrpd
Hoxb4
Hoxd3
Hoxd3
Hps6
Hrh3
Hrh3
Hs6st3
Hs6st3
Hsd17b11
Hspa12b
Icmt
Icmt
Id3

Igf1r
Igf1r
Il10
Ina
Ina
Ing1
Inhbe
Irf2
Irx5
Irx5
Isl2
Itih1
Jarid2
Jub
Jub
Jund1
Kcnab3
Khdrbs3
Klf9
Klhl9
Krt18
Lama1
Ldb1
Ldb1
Lemd1
Lemd1
Lgi3
Lgr5
Lhx1
Lif
Lrpprc
Lrrc4c
Luzp2
Lynx1
Magi3

Mapre3
Mcts2
Mdm1
Mfsd3
Mgat2
Mkln1
Mon2
Mvk
Mxi1
Myo10
Myo10
Myo10
Myo1c
Myo7a
Nbl1
Nek2
Neud4
Nfe2l1
Nfe2l1
Nfic
Nmur1
Notch1
Npr2
Nr1d1
Nr2e3
Nr2f2
Nr2f2
Nt5e
Ntng2
Oaz1
Pacsin1
Pacsin1
Pacsin3
Pah
Pax6

Pcdh10
Pcdh17
Pcdh17
Pcdhga5
Pcgf2
Pcgf5
Pcyt1a
Pcyt1a
Pcyt2
Pde3a
Pde3a
Pebp1
Peci
Pfn1
Phactr1
Phf5a
Pigz
Pik3r1
Pitpna
Pitx1
Pitx1
Pklr
Pla1a
Pla1a
Pla2g2c
Pla2g7
Plec1
Plec1
Plekhf1
Plekhg2
Plekhg2
Plekhg2
Pnpla2
Ppa1
Ppap2b

Ppap2b
Ppm1k
Ppp1r13b
Ppp2r2d
Ppp2r5c
Pptc7
Pptc7
Prdm1
Prdm5
Prodh
Psmd11
Ptger1
Ptprs
Qdpr
Rad9
Rassf3
Raver1
Rbm35a
Rbm9
Rcc2
Rcor2
Rdh7
Rgs6
Rnf121
Rnf135
Rnf185
Rnuxa
Rpl35
Rpp14
Saa4
Sall4
Scube3
Scube3
Scyl2
Sepx1

Sfrs2ip
Sgk
Sh2b2
Sh3pxd2a
Shc4
Six1
Six1
Six1
Slc10a6
Slc10a6
Slc23a2
Slc25a29
Slc2a9
Slc2a9
Slc35c1
Slc38a3
Slc44a2
Smad6
Smarcc2
Smarcc2
Smug1
Snf1lk
Sox2
Sphk2
Sphk2
Spnb2
Spryd4
Srebf1
Srebf1
Srebf1
Srrm1
St3gal3
Stk11
Stk11
Sulf2

Sulf2
Syngr1
Tal2
Tbl1xr1
Tbx3
Tcba1
Tcf2
Tcf7
Tcfap2c
Terf2
Tgm4
Tle4
Tle4
Tmem132a
Tmem26
Tnfrsf19l
Tnfrsf19l
Tob2
Tpd52
Tpt1
Trhde
Trmt5
Tssk4
Ttc12
Ttc33
Twist2
U46068
Uap1
Uap1
Ube2j2
Ube2j2
Ung
Ung
Usp2
Vps33b

Wasf3
Wnt2b
Wnt6
Yap1
Ypel4
Ypel4
Yy1
Zan
Zc3h11a
Zdhhc18
Zdhhc7
Zfp503
Zfp503
Zfp710
Zmynd10
Zswim5
Zswim5
Zswim5
Zyx

Supplementary Table 3a: KOA vs KO 716 hypomethylation genes on d16

Probe Name	Gene Name	Methylation difference value
A_68_P04039916	Nadk	0.33438
A_68_P13790503	3110002H16Rik	0.335319
A_68_P04677936	Polr2j	0.373654
A_68_P04737532	Cds1	0.379397
A_68_P11801698	Pou4f1	0.380366
A_68_P04039916	Nadk	0.384251
A_68_P03848124	Musk	0.386288
A_68_P04895996	Mapk10	0.3874
A_68_P10347437	Pax9	0.392248
A_68_P06926959	Nob1	0.403682
A_68_P13286098	Enpp4	0.403829
A_68_P07165457	Ints10	0.411601
A_68_P04621660	BC003324-6330548G22Rik	0.413014
A_68_P01769357	Mrg1	0.417223
A_68_P02043611	Disp2	0.420546
A_68_P07218631	Dusp4	0.427754
A_68_P06513729	E2f8	0.431134
A_68_P10030624	AW125753	0.431589
A_68_P03939278	Glipr2	0.432663
A_68_P10878754	Cks2	0.433079
A_68_P03695515	Lrrc47	0.439032
A_68_P09713729	Emid1	0.439182
A_68_P09322906	Lhx1	0.443472
A_68_P05422385	Dppa3	0.444436
A_68_P08466857	Lrig3	0.445734
A_68_P08514851	Nr2e1	0.447573
A_68_P10140483	Rrm2	0.448763
A_68_P04234539	Pdx1	0.451351
A_68_P13890291	BC019561	0.452449
A_68_P08981068	Pip5k2b	0.452795
A_68_P04621660	BC003324-6330548G22Rik	0.454152
A_68_P10941074	Zfyve16	0.458803
A_68_P11235588	Ccdc122	0.459644

A_68_P06751865	Slc7a6	0.460285
A_68_P10435877	Matn3	0.461617
A_68_P14335316	D19Wsu162e	0.464013
A_68_P14280877	Mxi1	0.46806
A_68_P09177142	Mpp3	0.470142
A_68_P09399954	Znhit3	0.470294
A_68_P04286123	Art3	0.472642
A_68_P07991425	Zic1	0.473978
A_68_P13797523	Pcdhga6	0.476046
A_68_P02367976	Isg20l2	0.476351
A_68_P08061094	2810417H13Rik	0.476609
A_68_P00783773	Ahctf1	0.477431
A_68_P02305769	Pld1	0.478533
A_68_P04568812	Hdh	0.479961
A_68_P00882730	Rnasel	0.48209
A_68_P13323539	Mapk14	0.483075
A_68_P09250456	Hoxb8	0.483086
A_68_P01173264	Dynlrb1	0.483994
A_68_P09477297	Hoxb5	0.485658
A_68_P11564443	9430077A04Rik-Prrxl1	0.487194
A_68_P00674953	Tor3a	0.490288
A_68_P07861271	Oaz2	0.491995
A_68_P12887986	Bcl6	0.492441
A_68_P07349283	Rbm13	0.492655
A_68_P10631307	Otp	0.495329
A_68_P05213176	Vamp8	0.496142
A_68_P07862963	Ihpk2	0.496748
A_68_P04917693	Eif3s9	0.49723
A_68_P03722059	Dhrs3	0.498595
A_68_P13298312	Epas1	0.498837
A_68_P10193185	Plek2	0.499316
A_68_P03307855	Hp1bp3	0.503824
A_68_P12968677	Opa1	0.504315
A_68_P12536557	BC027231	0.50987
A_68_P03208679	Hes5	0.509889

A_68_P03944620	E130114P18Rik	0.51431
A_68_P07041328	Tll1	0.514337
A_68_P12836214	Setd4-Cbr1	0.515803
A_68_P11653564	Rhobtb2	0.516433
A_68_P02650795	Setd7	0.516998
A_68_P09049964	Gria1	0.517365
A_68_P00572751	Cfc1	0.517766
A_68_P07550953	Zfp445	0.517841
A_68_P11145651	Klf5	0.520795
A_68_P06907386	Mrps31	0.522736
A_68_P06127908	Pak1	0.523321
A_68_P06506665	Apoe	0.52476
A_68_P04480991	Wfs1	0.526861
A_68_P02755413	Polr3gl	0.527058
A_68_P03123565	She	0.527206
A_68_P08694627	Dna2l	0.52751
A_68_P08215908	Suox	0.528381
A_68_P11350422	Anxa7	0.530776
A_68_P11656442	Gfra2	0.532242
A_68_P13251243	Cyp4f13	0.535215
A_68_P03968118	Car8	0.535363
A_68_P09568010	Ormdl3	0.535565
A_68_P00931382	En1	0.536037
A_68_P13255091	Pcaf	0.536308
A_68_P03771652	Tmem68	0.536744
A_68_P04480991	Wfs1	0.538198
A_68_P13032833	Eif2b5	0.538408
A_68_P13696223	Onecut2	0.538472
A_68_P03944620	E130114P18Rik	0.53884
A_68_P05951989	Bub3	0.53905
A_68_P03208679	Hes5	0.539328
A_68_P04039488	9630013D21Rik	0.539866
A_68_P12636173	Lsg1	0.540489
A_68_P07036094	Mcm5	0.543046
A_68_P04926197	Fzd10	0.543071

A_68_P04398639	Evi5	0.544129
A_68_P02983152	Pcdh10	0.544519
A_68_P11483530	Ebf2	0.545231
A_68_P09273682	Becn1	0.545993
A_68_P00576308	Mosc2	0.547011
A_68_P01617284	4930402H24Rik	0.547219
A_68_P05889182	Xrcc1	0.547257
A_68_P14431113	Mxi1	0.549289
A_68_P10157596	Dicer1	0.550286
A_68_P11079538	Sirt5	0.550441
A_68_P08701847	Rbms2	0.550909
A_68_P09245911	Ifi47	0.55136
A_68_P06428107	Zfp36	0.553325
A_68_P02173326	Smtnl1-Timm10	0.554928
A_68_P02938391	Tmem79-Smg5	0.556032
A_68_P12550711	Stch	0.556045
A_68_P08532207	Slc35e3	0.556395
A_68_P00581118	Rbbp5	0.556687
A_68_P13551633	Socs5	0.562088
A_68_P03353696	Sdc3	0.562721
A_68_P09018865	Cbx4	0.564329
A_68_P14979287	Kif4	0.56485
A_68_P10857139	Cdc14b	0.56525
A_68_P14108737	ENSMUST00000083658.1:429	0.566965
A_68_P00303096	Lamc2	0.567207
A_68_P01085703	Ptp4a1	0.567396
A_68_P06599540	Armc5	0.567641
A_68_P15175265	Tcfe3	0.567909
A_68_P11450608	Rhobtb2	0.568469
A_68_P07096563	B130050I23Rik	0.568706
A_68_P11069441	Plk2	0.568849
A_68_P02940604	Mgst2	0.570302
A_68_P05715101	Xpc	0.570338
A_68_P13515259	Pim1	0.570383
A_68_P11505019	Zfp503	0.571278

A_68_P14458023	Prkg1	0.572162
A_68_P07339397	4921524J17Rik	0.572609
A_68_P11419740	Nkx3-1	0.573838
A_68_P00149897	Tagln2	0.574717
A_68_P12816944	Ephb3	0.575738
A_68_P03819892	4922503N01Rik	0.576167
A_68_P02189368	Eif2ak4	0.576285
A_68_P12755374	App	0.577126
A_68_P14438125	Actr1a	0.577142
A_68_P09591872	Nsg2	0.577258
A_68_P02455823	Polr3gl	0.577305
A_68_P13883491	Pcdha9	0.577378
A_68_P12853498	Mx2	0.578649
A_68_P00242827	EG277333	0.578667
A_68_P12063911	A_68_P12063911	0.579329
A_68_P07835083	Entpd3	0.579778
A_68_P05885506	Dbx1	0.580525
A_68_P11752826	Socs4	0.580606
A_68_P11111042	Tcfap2a	0.581459
A_68_P07893192	Folr4	0.58195
A_68_P09437147	Pfn1-Eno3	0.582255
A_68_P01127576	Tsga10-EG623661	0.58265
A_68_P03127713	Sox2	0.582879
A_68_P01725957	Pfdn4	0.583498
A_68_P01197065	2700007P21Rik	0.584847
A_68_P13895708	Slc12a2	0.584985
A_68_P09474540	Hoxb3	0.58501
A_68_P09997292	Id2	0.585413
A_68_P01963949	Rbm39	0.585492
A_68_P00206509	St8sia4	0.585526
A_68_P02060987	Defb25-Rem1	0.585842
A_68_P06266771	Ilk	0.586458
A_68_P00588267	Cflar	0.586902
A_68_P04586625	Fbxl10	0.587923
A_68_P05970380	Capns1	0.588775

A_68_P10895011	Cltb	0.590025
A_68_P08999972	Tbx21	0.590125
A_68_P08621597	D10Wsu102e	0.593262
A_68_P07788751	1300017J02Rik	0.59338
A_68_P06294286	Epn1	0.594456
A_68_P10301168	Tgfb3-1700019E19Rik	0.595093
A_68_P08815423	Tsfm	0.595449
A_68_P10341311	Vsnl1	0.595459
A_68_P10745919	Ipo11	0.597311
A_68_P07498420	Sin3a	0.597796
A_68_P08890336	Spock2	0.600832
A_68_P04040117	4732473B16Rik	0.60207
A_68_P07361088	Rhou	0.602513
A_68_P08172363	Spa17	0.602686
A_68_P13117095	Bak1	0.603753
A_68_P05912818	Mkrn3	0.603907
A_68_P08656285	Phyhipl	0.604453
A_68_P05416729	Cxcl12	0.604653
A_68_P03751631	Zc3h12a	0.604911
A_68_P05807222	Trim24	0.604918
A_68_P06284631	Tbc1d17	0.605519
A_68_P01943851	AA467197	0.605597
A_68_P10205319	Yy1	0.606662
A_68_P00820783	Capn8	0.606844
A_68_P04000865	Rnf207-Rpl22	0.607215
A_68_P07554616	Rbms3	0.607697
A_68_P09078515	Meis1	0.608781
A_68_P14160293	Emx2	0.609316
A_68_P00316905	Tcfcp2l1	0.610395
A_68_P07853424	Cgnl1	0.610782
A_68_P11073758	Zfp366	0.611014
A_68_P12487908	1700021K19Rik-Fyttd1	0.611418
A_68_P04970366	Chst12	0.612011
A_68_P01454242	ENSMUST00000083520.1:-1564	0.612698
A_68_P03685403	Dmrta2	0.613915

A_68_P08077145	Nedd4	0.614169
A_68_P03079741	Map2k1ip1	0.615654
A_68_P01569256	Traf6	0.616308
A_68_P03353902	1110049F12Rik	0.61634
A_68_P08395277	Smarcc2	0.61691
A_68_P12901529	Hunk	0.617503
A_68_P04340618	Ung	0.617519
A_68_P07950291	Sh3px3	0.617946
A_68_P02110665	Stam2	0.618036
A_68_P06099522	Apoc1	0.618205
A_68_P00236760	Tmem163	0.618859
A_68_P11137005	Spry2	0.618875
A_68_P11516634	Plekhc1	0.618895
A_68_P09038979	Kremen1	0.618943
A_68_P03685403	Dmrta2	0.6193
A_68_P14350160	Gfra1	0.619379
A_68_P05732741	Ccnd2	0.619453
A_68_P08997356	Ankrd40	0.620263
A_68_P13067632	Pkd1	0.62063
A_68_P14385037	Smarca2	0.621384
A_68_P07614034	Jam3	0.621407
A_68_P14257641	Stambpl1	0.621458
A_68_P14802381	Plxna3	0.622124
A_68_P13643168	Gata6	0.6229
A_68_P01011002	Elk4	0.622918
A_68_P13330489	Ehd3	0.623348
A_68_P10556075	Hivep1	0.623367
A_68_P09817295	Dlst	0.623716
A_68_P13006429	Ufd1l	0.623814
A_68_P07179412	Rbpms	0.623953
A_68_P09632282	Dusp3	0.624543
A_68_P10265199	Kns2	0.624648
A_68_P00129085	1110058L19Rik	0.624708
A_68_P11828305	ENSMUST00000083663.1:-2499	0.625622
A_68_P03211661	2610204G22Rik	0.625705

A_68_P09805220	Trib2	0.6261
A_68_P01641588	Serinc3	0.626813
A_68_P04178087	Phox2b	0.626975
A_68_P02251924	Uckl1	0.627475
A_68_P12422403	Ep300	0.627625
A_68_P07491070	Ephb1	0.629197
A_68_P11922964	Klf10	0.630402
A_68_P08778846	Gja1	0.630408
A_68_P11739627	Gata4	0.631572
A_68_P00494851	Degs1	0.631716
A_68_P08284201	Wif1	0.631745
A_68_P03869852	Pax7	0.631814
A_68_P06934687	Jph3	0.632188
A_68_P04040117	4732473B16Rik	0.632617
A_68_P06874491	3110005G23Rik	0.632634
A_68_P14115940	Smad7	0.633235
A_68_P14187519	Lbx1	0.633418
A_68_P02617476	Uox	0.63346
A_68_P14385433	Pik3ap1	0.634476
A_68_P03628649	Toe1-Mutyh	0.63461
A_68_P08256155	Moxd1	0.635139
A_68_P07791783	Suhw4	0.635868
A_68_P04562414	Stx1a	0.636046
A_68_P08803717	Hsd17b6	0.63753
A_68_P13439820	Ptprs	0.638461
A_68_P07923222	AI593442	0.638891
A_68_P13883460	Pcdha9	0.638949
A_68_P04575353	Gbas	0.639651
A_68_P15201288	Zfx	0.639693
A_68_P07813354	Gclc	0.640072
A_68_P14735269	Gria3	0.640582
A_68_P06320275	Cyfip1	0.6408
A_68_P08156834	1110032A03Rik	0.640898
A_68_P00934875	Rxrg	0.64105
A_68_P00718610	Rrs1	0.641141

A_68_P11539394	Gpc5	0.641244
A_68_P09314768	Grn	0.641465
A_68_P04365068	Klb	0.641597
A_68_P03342791	Arhgef19	0.642442
A_68_P10913242	Wrnip1	0.642767
A_68_P05939018	A630091E08Rik	0.643065
A_68_P05217166	Ephb6	0.643151
A_68_P06592634	Cd3eap-Ppp1r13l	0.643194
A_68_P00793519	Ihh	0.64424
A_68_P02153759	Cstf3	0.644402
A_68_P06168319	Isg20l1	0.644571
A_68_P14121622	Tcof1	0.64622
A_68_P05793539	Cpa1	0.647262
A_68_P08248934	Tmpo	0.647564
A_68_P03211661	2610204G22Rik	0.647882
A_68_P05565689	Ptms	0.64816
A_68_P04455959	Zfp469	0.649303
A_68_P04734427	Shroom3	0.649304
A_68_P13266254	Thbs2	0.649436
A_68_P11082814	Snag1	0.649618
A_68_P09483214	Hist3h2a	0.649656
A_68_P12049849	Mpped1	0.649965
A_68_P00214091	Pkp1	0.649972
A_68_P07010147	Hand2	0.65002
A_68_P06173453	2310008H09Rik	0.65024
A_68_P03566955	Fbxo42	0.650412
A_68_P07716416	Crtap	0.650597
A_68_P02254945	Coq4	0.650854
A_68_P05138561	Cdkn1b	0.651146
A_68_P13019175	Pcnp	0.651152
A_68_P11700529	Cacna2d3	0.651248
A_68_P01718593	Rexo4-Adamts13	0.651473
A_68_P13523043	Akap8l	0.651674
A_68_P08277015	Cart1	0.651726
A_68_P13251244	Cyp4f13	0.651959

A_68_P03041147	Sfrp2	0.652126
A_68_P03738874	Ambp	0.652361
A_68_P13339417	ENSMUST00000086914.3:-1812	0.652432
A_68_P04978120	Fosl2	0.652592
A_68_P08530152	Cnn2	0.652908
A_68_P05547385	Gprin3	0.653193
A_68_P15093622	Nxt2	0.654235
A_68_P08538267	Gnptab	0.654511
A_68_P01592489	Ehf	0.654882
A_68_P04011260	6230416J20Rik	0.655016
A_68_P12958415	Alg3-1810009K13Rik	0.655359
A_68_P14494409	Dmrt2	0.656318
A_68_P03505622	E2f2	0.656864
A_68_P03228980	Acot7	0.658109
A_68_P14979296	Pdzd11	0.658331
A_68_P11347694	Bmpr1a	0.659216
A_68_P05452380	Znrf2	0.659383
A_68_P02737741	Psmd4	0.659422
A_68_P03127703	Sox2	0.660056
A_68_P14780157	MGC107702	0.661264
A_68_P06409764	Atp2a1	0.661951
A_68_P03444650	Tmem67-C430048L16Rik	0.662466
A_68_P00064672	Cd55	0.662967
A_68_P04010940	Foxd2	0.663192
A_68_P08618192	Atcay-9130206N08Rik	0.664072
A_68_P06625976	Lypd4	0.664282
A_68_P03810519	Zcchc7	0.664435
A_68_P13722576	Zfp532	0.664456
A_68_P09099826	Srebf1	0.665382
A_68_P13158664	1300003B13Rik	0.665569
A_68_P05974477	A630091E08Rik	0.665787
A_68_P06053252	3200002M19Rik	0.666145
A_68_P00408838	Cspp1	0.666217
A_68_P01348198	Surf6	0.666297
A_68_P14171227	Sfxn4	0.666378

A_68_P14889744	Mid1ip1	0.667118
A_68_P12871003	Sema5b	0.668721
A_68_P06193351	Dmpk	0.668908
A_68_P07263029	Cenpt	0.669163
A_68_P10860226	Gmpr	0.670033
A_68_P05950422	Smpd1	0.670039
A_68_P13300318	Tsga2	0.670137
A_68_P03953505	Ppp1r8	0.671041
A_68_P09440235	Ehbp1	0.671147
A_68_P08187608	Pml	0.671575
A_68_P02312655	Tbx15	0.672018
A_68_P03041132	Sfrp2	0.672037
A_68_P10730038	Tbce	0.672223
A_68_P04844132	Cxcl4	0.672474
A_68_P05102045	Sec13	0.673579
A_68_P14448901	Ints5	0.673908
A_68_P11569895	Ppyr1	0.674548
A_68_P04859957	Slc12a9	0.67466
A_68_P09436612	Adra1b	0.675091
A_68_P07540084	AW551984	0.675487
A_68_P04460830	Mospd3	0.675602
A_68_P06208765	Prmt1	0.676579
A_68_P12214035	Creld2	0.676927
A_68_P06583785	Pvrl2	0.677409
A_68_P01079257	Fzd7	0.677878
A_68_P03156403	Palmd	0.678009
A_68_P05891727	Grik5-Zfp574	0.678212
A_68_P06490200	2410015N17Rik	0.678538
A_68_P09395674	1700007I06Rik-Stk10	0.679022
A_68_P11971534	Kcns2	0.679256
A_68_P04893782	Snx8	0.679478
A_68_P06962856	Lig4	0.679523
A_68_P07558348	Lbxcor1	0.679531
A_68_P09656833	Agxt2l2	0.680108
A_68_P10466438	Ptch1	0.680343

A_68_P08761742	5330438D12Rik	0.681098
A_68_P06939996	Il15	0.681167
A_68_P04277065	Cxcl1	0.681199
A_68_P00451766	Nmnat2	0.681712
A_68_P01984542	1700011J10Rik	0.682102
A_68_P06662895	Art2b	0.682536
A_68_P01943849	AA467197	0.682699
A_68_P13383251	H2-T24	0.682821
A_68_P07618605	Gnai2	0.683057
A_68_P12140837	Eif3s6ip	0.683122
A_68_P06771773	Snapc2	0.683176
A_68_P06788634	BC021891	0.683224
A_68_P05461992	EG232599	0.683441
A_68_P05439808	Irak2	0.683478
A_68_P11925010	Ppara	0.683579
A_68_P05872318	Scnn1a	0.684243
A_68_P13978271	Mkx	0.684545
A_68_P12648829	Gp5	0.685045
A_68_P04926190	Fzd10	0.685184
A_68_P04120983	Mad1l1	0.686045
A_68_P03293677	Slc35a1	0.686215
A_68_P02404444	Cxxc4	0.686707
A_68_P07505359	Eif3s4	0.686787
A_68_P02798117	Slc7a11	0.687623
A_68_P00270265	Slc41a1	0.687973
A_68_P05905001	Ccnd1	0.688101
A_68_P07300676	4930402E16Rik	0.689211
A_68_P14001180	Aldh7a1	0.689492
A_68_P13897671	Tnfaip8	0.689506
A_68_P04607968	Rpl6	0.689697
A_68_P06846135	Fbxl8	0.689719
A_68_P03843631	BC025833	0.68999
A_68_P07513274	Pcolce2	0.690596
A_68_P11108180	Nup153	0.69145
A_68_P09651374	Actg1	0.69275

A_68_P12281826	Grhl2	0.693157
A_68_P00221558	Prrx1	0.693164
A_68_P07251405	Foxf1a	0.693441
A_68_P01486057	Samhd1	0.693737
A_68_P01736457	Ypel4	0.693764
A_68_P01299887	Hoxd3	0.693943
A_68_P06045434	Frag1	0.694189
A_68_P13295845	Pdpk1	0.69448
A_68_P06226765	1810054G18Rik	0.69478
A_68_P01470481	Cse1l	0.695074
A_68_P12971556	Ifngr2	0.695351
A_68_P12066484	Hoxc10	0.696375
A_68_P03093929	Prpf38b	0.69639
A_68_P07922098	Rbm7	0.696685
A_68_P10111464	Prima1	0.697769
A_68_P11546934	Dlg7	0.698061
A_68_P06887581	Hsf4	0.698102
A_68_P02436074	Ccbl2	0.698306
A_68_P01949031	Mdk	0.698454
A_68_P03854451	BC003266	0.698745
A_68_P09038027	Igf2bp1	0.69921
A_68_P14030415	Etf1	0.699245
A_68_P10006538	Rps6ka5	0.699632
A_68_P04859957	Slc12a9	0.699783
A_68_P04901404	Tmem142a	0.699961
A_68_P05808775	Bhlhb2	0.699968
A_68_P06095045	Aldoa	0.700418
A_68_P09533960	Dusp14	0.700664
A_68_P14525996	Timm8a1	0.701036
A_68_P07299665	Nfix	0.701278
A_68_P06928235	Spire2	0.701613
A_68_P07910201	Lyzl4	0.701688
A_68_P04350697	Gtf2i	0.702071
A_68_P03900317	Prpf38a	0.702588
A_68_P09739930	Hoxb7	0.70278

A_68_P03953505	Ppp1r8	0.702818
A_68_P11585136	Nupl1	0.70289
A_68_P03647830	Kti12	0.702928
A_68_P13107839	Abcg8	0.703674
A_68_P11534471	Lgals3	0.703816
A_68_P02123034	Zfhx1b	0.704136
A_68_P09541955	Map2k6	0.70421
A_68_P07679434	Atp1b3	0.704446
A_68_P15209143	Plp2	0.704493
A_68_P09195114	A830031A19Rik-Bcl11a	0.704913
A_68_P09914489	Daam1	0.705003
A_68_P12212512	Amigo2	0.705003
A_68_P14549390	Zic3	0.705168
A_68_P10277800	Myt1l	0.705632
A_68_P08764275	Csnk1g2	0.70566
A_68_P12334898	Zhx2	0.706078
A_68_P06412434	Bnc1	0.706683
A_68_P08479961	Slc19a1	0.706875
A_68_P04553689	Cenpc1-AI586015	0.707637
A_68_P09926633	BC048191:-3942	0.707901
A_68_P04522816	Pdx1	0.707962
A_68_P10300471	D230037D09Rik	0.708385
A_68_P05529381	Rerg	0.708454
A_68_P06986126	Ppp2cb	0.70891
A_68_P06789607	Kcng4	0.70991
A_68_P13976398	Csnk1a1	0.710593
A_68_P07757445	Rab3d	0.711037
A_68_P00041396	Mgat4a	0.711187
A_68_P04081654	Dio1	0.711219
A_68_P03900317	Prpf38a	0.711558
A_68_P09937351	Flrt2	0.711792
A_68_P05987685	Olfr503	0.712091
A_68_P13896689	Zfp516	0.712217
A_68_P14255652	Cybasc3	0.712276
A_68_P11855909	Ly6d	0.712405

A_68_P15089789	EG546088	0.712543
A_68_P13282822	Qk	0.712736
A_68_P14918058	Ap1s2	0.713621
A_68_P10838522	Jarid2	0.713903
A_68_P09995749	Ppm1a	0.71459
A_68_P07813357	A_68_P07813357	0.71467
A_68_P07134632	Rbm35b	0.715007
A_68_P00834946	Copa	0.715046
A_68_P11559162	Nid2	0.715649
A_68_P12386971	Ank	0.715761
A_68_P08653425	Sh3md4	0.71583
A_68_P05976681	C330005M16Rik	0.716371
A_68_P08967215	Grik2	0.716681
A_68_P00588306	Slco5a1	0.717103
A_68_P13906889	Sall3	0.717174
A_68_P04247170	Zfp326	0.717347
A_68_P09031100	Myo1c	0.717408
A_68_P04487381	1110008J03Rik	0.717462
A_68_P09521694	1110014K08Rik	0.717536
A_68_P01182959	Nfatc2	0.717548
A_68_P05627272	Galnact2	0.718159
A_68_P10269934	Evl	0.718532
A_68_P01717991	Btbd14a	0.718559
A_68_P04606873	Scarb2	0.718806
A_68_P09412315	Ube2b-Cdkl3	0.718836
A_68_P04350697	Gtf2i	0.71887
A_68_P03602815	Htr1d	0.718974
A_68_P06483147	2310044H10Rik	0.71928
A_68_P06201943	Usp47	0.719311
A_68_P00590849	Ncl	0.719372
A_68_P10723432	Zfp817	0.719681
A_68_P07561235	Hspa8	0.719709
A_68_P10072364	ENSMUST00000083499.1:-3315	0.72005
A_68_P12761113	Stch	0.720295
A_68_P09132227	Cacng4	0.720326

A_68_P02269102	Arl5a	0.720427
A_68_P00245688	Ttll4	0.720452
A_68_P10137568	Fbln5	0.720729
A_68_P04723864	Cenpa	0.720768
A_68_P06061321	Kdelr1	0.720771
A_68_P02896233	Usp13	0.720775
A_68_P12636185	Lsg1	0.721036
A_68_P01214281	Nmt2	0.721116
A_68_P05495854	Casd1	0.721235
A_68_P03930447	Cldn19	0.721534
A_68_P12979570	Olig2	0.721592
A_68_P07291347	Calb2	0.721864
A_68_P02561233	Nexn	0.722171
A_68_P00904213	Asnsd1	0.722255
A_68_P08629281	Adarb1	0.722256
A_68_P06848465	Dnajb1	0.722396
A_68_P12445601	Fkbp11	0.722488
A_68_P11375105	Ap1g2	0.722501
A_68_P10147981	Dnmt3a	0.723924
A_68_P06614033	B3gnt8	0.724026
A_68_P13271863	Gtf2h4	0.724153
A_68_P06249760	Kcnc3	0.724744
A_68_P13019315	Nit2	0.724859
A_68_P03184759	Gucy1a3	0.724905
A_68_P08026226	Sema3b	0.725159
A_68_P14142812	Nolc1	0.725165
A_68_P02314772	Mtx1-Thbs3	0.725543
A_68_P03806051	Usp1	0.725649
A_68_P14524801	Esx1	0.725683
A_68_P01640329	Btbd3	0.725986
A_68_P07920795	Apoa4	0.726049
A_68_P10905580	Nrn1	0.726352
A_68_P03082194	Prkab2	0.72646
A_68_P02205490	Epb4.1l1	0.726589
A_68_P13205532	Glo1	0.727196

A_68_P11851446	4930570C03Rik	0.727507
A_68_P03594238	Epha7	0.727787
A_68_P12627348	Cldn1	0.727885
A_68_P03353902	1110049F12Rik	0.728117
A_68_P10636349	Mrs2l	0.728348
A_68_P03534901	Mycl1	0.728365
A_68_P04808473	Ephb4	0.72853
A_68_P08903576	ORF61-Cnn2	0.728539
A_68_P07983768	Eomes	0.728753
A_68_P14462408	Rtn3	0.728887
A_68_P04056540	Mmp23	0.728891
A_68_P14109314	Osbpl1a	0.729085
A_68_P08005228	Acy1	0.729125
A_68_P04629065	Gfi1	0.729461
A_68_P10477201	Sfxn1	0.729557
A_68_P07569559	Sacm1l	0.72975
A_68_P07037178	Nanos3	0.729966
A_68_P03303258	Errfi1	0.730438
A_68_P08169692	Mtmr2	0.73051
A_68_P06121239	Dll3	0.730619
A_68_P02545158	Cttnbp2nl	0.730689
A_68_P01811585	Tsc1	0.73073
A_68_P07746050	Vprbp	0.731166
A_68_P15075276	Zfp92	0.73123
A_68_P09374089	Pdlim4	0.731263
A_68_P07777017	Foxb1	0.731377
A_68_P09032003	Pold2	0.731473
A_68_P06660297	Zfp536	0.731479
A_68_P07406055	Got1l1	0.731995
A_68_P06220704	Abcc6-Nomo1	0.732064
A_68_P04051233	2310026E23Rik	0.732118
A_68_P03634721	Ccdc21	0.732281
A_68_P09042040	ENSMUST00000021240.4:169	0.732355
A_68_P11605070	Klhl1	0.732676
A_68_P11726132	Fgf9	0.732736

A_68_P03514184	Srm	0.733083
A_68_P14732983	Phka1	0.733142
A_68_P12674824	Dvl3	0.733291
A_68_P04459423	Tmem130	0.733407
A_68_P09689162	Hoxb2	0.733434
A_68_P05958269	Scube2	0.73353
A_68_P05393698	Mest	0.734038
A_68_P07189858	Il12rb1	0.734241
A_68_P03532964	B230396O12Rik	0.734283
A_68_P10747279	1110007C09Rik	0.73432
A_68_P04844132	Cxcl4	0.73445
A_68_P03807576	Wdtc1	0.734535
A_68_P09061472	Ltc4s	0.73459
A_68_P09413265	Adam11	0.734715
A_68_P12842136	Cdgap	0.734782
A_68_P13293615	Gbl	0.73485
A_68_P12721918	1500031L02Rik	0.734869
A_68_P02947763	Spg20	0.734883
A_68_P01424449	Tbr1	0.73499
A_68_P10627340	Cdkal1	0.735134
A_68_P14987574	Sms	0.735601
A_68_P07497064	Rdx	0.735837
A_68_P14554864	Mid1ip1	0.735903
A_68_P11937901	BC030396	0.736086
A_68_P01200951	Epc2	0.736114
A_68_P09225377	Sfrs1	0.736243
A_68_P15216256	Efnb1	0.736452
A_68_P04412246	Dgkq	0.736456
A_68_P10815405	Sfxn1	0.736636
A_68_P14045062	Sh3tc2	0.73703
A_68_P02755421	Polr3gl	0.737348
A_68_P06188076	Ccdc123	0.737588
A_68_P06069472	Wnt11	0.737674
A_68_P14231112	Rps6kb2	0.738043
A_68_P04277062	Cxcl1	0.738067

A_68_P06506007	Gys1	0.738125
A_68_P13533864	Ddx11	0.738137
A_68_P06554264	Ndnl2	0.738409
A_68_P06098132	Zfp553	0.738799
A_68_P06316193	Ebf3	0.738807
A_68_P11404050	Lcp1	0.738821
A_68_P11205877	Zfp503	0.739096
A_68_P07444192	Indol1	0.739462
A_68_P05548217	Chd4	0.739612
A_68_P06626588	6430526N21Rik	0.739613
A_68_P04167205	Adrbk2	0.74002
A_68_P02504418	Tpd52	0.740155
A_68_P09077708	Trim7	0.74027
A_68_P04329254	Cdk2ap1	0.74029
A_68_P02638045	Lysmd1	0.740636
A_68_P06125211	Athl1	0.741247
A_68_P07831909	Tle3	0.741251
A_68_P00694567	Crry	0.741286
A_68_P04306467	Whsc2	0.741304
A_68_P08799615	Cdc2l6	0.741383
A_68_P01311715	Hspa5	0.741452
A_68_P05331102	Gng11	0.741524
A_68_P00716971	Tnfrsf11a	0.741673
A_68_P13679277	Pcdhga4	0.741934
A_68_P14240187	Sart1	0.742043
A_68_P12788633	Tmem41a	0.742073
A_68_P13337800	Slc37a1	0.742239
A_68_P01956107	Spred1	0.742308
A_68_P01220442	Angptl2	0.742356
A_68_P01783249	Ak1	0.742411
A_68_P13685522	Pcyox1l	0.742463
A_68_P01364522	Fbn1	0.742516
A_68_P03338382	Dmrta2	0.742552
A_68_P03599365	Lzic	0.742687
A_68_P12269379	Slc1a3	0.742726

A_68_P00439340	Vangl2	0.742912
A_68_P03730164	Tmem54	0.743161
A_68_P14097628	2010001M09Rik	0.743194
A_68_P14015399	Grp	0.743211
A_68_P10573753	BC021881:-1155	0.743413
A_68_P14960254	Gpc4	0.743477
A_68_P15206339	Sox3	0.743529
A_68_P04861691	2610024G14Rik	0.743561
A_68_P10884024	Rsl1	0.743648
A_68_P09417907	Hoxb2	0.743755
A_68_P12390812	pPtp4a3	0.744002
A_68_P06413052	Axl	0.74411
A_68_P08513988	Cdk4	0.744122
A_68_P10070767	Rock2	0.744417
A_68_P02145945	Clp1	0.744424
A_68_P10390439	C130076O07Rik	0.744766
A_68_P08969524	Pdxk	0.744987
A_68_P14245824	Rcl1	0.745044
A_68_P14844556	Rs1	0.745045
A_68_P12081602	Rbm9	0.745046
A_68_P14554584	Pdk3	0.745052
A_68_P05193381	Phf14	0.745277
A_68_P14612493	Pnck	0.745332
A_68_P09350828	Smcr8	0.745427
A_68_P04044353	Med18	0.745706
A_68_P07582959	Endod1	0.745813
A_68_P09143801	Pps	0.745821
A_68_P14313887	Shoc2	0.745899
A_68_P02979898	Arhgef2	0.746071
A_68_P12138338	Hoxc13	0.74619
A_68_P02116516	Zcchc3	0.746193
A_68_P08985425	Crhr1	0.746215
A_68_P04836173	Ift81	0.746275
A_68_P13359198	Lrpprc	0.746291
A_68_P05182308	Gpr85	0.746631

A_68_P06643189	Rps19	0.746757
A_68_P03747411	Tesk1	0.74684
A_68_P03747745	Ptprf	0.74713
A_68_P09563208	5730593N15Rik	0.747144
A_68_P04688861	Actr3b	0.747413
A_68_P12500585	Hira	0.748553
A_68_P12069845	5730557B15Rik	0.748675
A_68_P06924340	Foxf1a	0.748685
A_68_P02381326	Slc25a24	0.748822
A_68_P01697623	Pbx3	0.748902
A_68_P03898253	D630039A03Rik	0.749022
A_68_P02046834	Egfl7	0.749039
A_68_P10645306	Nfil3	0.74924
A_68_P09258755	A630050E13Rik	0.749273
A_68_P02711184	Rap1a	0.749381
A_68_P09240372	Tbkbp1	0.749805
A_68_P09292536	Gria1	0.749929
A_68_P09595945	Cdc6	0.749965

Supplementary Table 3b: KOA vs KO 172 hypermethylation genes on d16

Probe Name	Gene Name	Methylation difference value
A_68_P07263831	Shcbp1	2.132419
A_68_P14368537	Ankrd1	1.771911
A_68_P13823227	Pcdhga11	1.703845
A_68_P15081221	Wdr13	1.700032
A_68_P03224123	Spata21	1.632593
A_68_P09009571	Olfr223	1.631738
A_68_P03224123	Spata21	1.606029
A_68_P05448315	Kcna5	1.596381
A_68_P01519278	1700037H04Rik	1.593843
A_68_P08377436	Ascl1	1.564098
A_68_P06550934	Lrrc4b	1.554605
A_68_P07829944	Paqr9	1.534036
A_68_P03075997	Dcamkl2	1.529319
A_68_P06683149	Gtl3	1.512552
A_68_P07341251	Spata4	1.509469
A_68_P03251705	Ccnc	1.505269
A_68_P10499714	Irf4	1.501672
A_68_P09361410	Alox15	1.500519
A_68_P04331228	Dtx1	1.500433
A_68_P09891693	Cdkl1	1.498414
A_68_P10356757	Trim9	1.49836
A_68_P04844116	Cxcl4	1.489747
A_68_P03912425	Aqp7	1.488285
A_68_P12255657	Baiap2l2	1.477926
A_68_P00072270	Ptpn14	1.477407
A_68_P12074308	Galr3	1.466488
A_68_P04300636	Pxn	1.447611
A_68_P07024704	1700029J07Rik	1.443679
A_68_P05958477	Tmem16e	1.441569
A_68_P09440376	Spag5	1.439846
A_68_P14099757	B230399E16Rik	1.439474
A_68_P09922529	Ptprn2	1.439467
A_68_P03406403	Ubxd3	1.436197

A_68_P05934226	Kcnc3	1.434424
A_68_P07620405	Gtf2a2	1.430305
A_68_P02203172	Elf5	1.42878
A_68_P12151832	Wnt10b	1.42828
A_68_P03991577	Nfx1	1.426525
A_68_P05111640	Trim24	1.423991
A_68_P13355809	Grm4	1.423196
A_68_P10492735	Pitrm1	1.423047
A_68_P07945909	Slc25a20	1.421333
A_68_P03759607	1810019J16Rik	1.4211
A_68_P04806722	Rint1	1.420651
A_68_P06099228	2310044H10Rik	1.420063
A_68_P08980029	Krt27	1.419464
A_68_P04390199	Ung	1.419038
A_68_P13436841	Brd2	1.417372
A_68_P08481839	Pcsk4	1.415729
A_68_P10401129	Sgpp1	1.415716
A_68_P10190658	Msgn1	1.413983
A_68_P05321956	Kcnd2	1.412861
A_68_P05958473	Tmem16e	1.412171
A_68_P01134681	Col19a1	1.410497
A_68_P00400075	Kif1a	1.409992
A_68_P01499103	Ggta1	1.406855
A_68_P14103036	Gm672	1.405967
A_68_P09329905	Sp2	1.402671
A_68_P12079965	2810451A06Rik	1.402134
A_68_P12382326	A4galt	1.400838
A_68_P03639993	Matn1	1.400078
A_68_P01552185	Prosapip1	1.39921
A_68_P12120913	Aqp5	1.397422
A_68_P03107074	Ccdc39	1.397366
A_68_P12441858	Adamts20	1.397252
A_68_P13927242	Pcdhac2	1.397074
A_68_P13090368	BC008155	1.396857
A_68_P11679711	Dnajc3	1.393891

A_68_P13488443	Prss34	1.392889
A_68_P13340765	2410015M20Rik-Rpl36	1.392881
A_68_P10006453	Foxg1	1.391241
A_68_P14271693	Dmrt1	1.389175
A_68_P04671528	Slc10a4	1.389035
A_68_P12003446	Oxct1	1.387786
A_68_P10147998	Ppp2r5c	1.387527
A_68_P10168388	Otub2	1.387269
A_68_P09143803	Pps	1.387207
A_68_P06046412	H47	1.387182
A_68_P01442677	Adam33	1.384526
A_68_P04607980	Rpl6	1.383943
A_68_P00711135	Slc45a3	1.383073
A_68_P00366170	1110034B05Rik	1.383055
A_68_P05944068	Art1	1.382583
A_68_P05123645	Edem1	1.380316
A_68_P08688126	Pip5k2c	1.379071
A_68_P13551185	H2-M10.2	1.378498
A_68_P09229843	Sec14l1	1.375964
A_68_P00179487	Dusp27	1.375781
A_68_P00331311	EG240916	1.37409
A_68_P05889184	Xrcc1	1.373265
A_68_P12433410	Letmd1	1.372015
A_68_P04964830	Ache	1.371675
A_68_P03809570	Ubap2	1.370658
A_68_P09443620	Tcf7	1.36996
A_68_P06900371	Ogfod1	1.369856
A_68_P11758642	Zic2	1.369756
A_68_P01944492	4930526D03Rik	1.368976
A_68_P05085703	Itpr1	1.367395
A_68_P06526842	Dpysl4	1.36715
A_68_P03472730	Pou3f1	1.367121
A_68_P06996032	Bcar1	1.365262
A_68_P01381082	Myt1	1.364911
A_68_P13937104	Myo5b	1.364284

A_68_P03702179	Asph	1.363066
A_68_P01591335	Rtel1	1.362107
A_68_P14385196	Cabp4	1.361633
A_68_P14362302	Rps6ka4	1.361527
A_68_P00708957	Pax3	1.361
A_68_P00174516	Chrnd	1.359577
A_68_P09115945	BC022224	1.358838
A_68_P10626468	BC003212:608	1.358025
A_68_P09977013	Psma6	1.357984
A_68_P11288375	Grid1	1.357733
A_68_P09585043	Lig3	1.357034
A_68_P07257166	Ssbp4-Lrrc25	1.356851
A_68_P06523723	Htra1	1.356832
A_68_P04456805	Aym1	1.356668
A_68_P02301827	Tpd52	1.355564
A_68_P00216168	1500015O10Rik	1.354775
A_68_P00445737	En1	1.354372
A_68_P10845417	Pak1ip1	1.353697
A_68_P14408920	Sart1	1.352719
A_68_P04538401	Rxfp2	1.352173
A_68_P01695051	Jag1	1.351864
A_68_P13387156	Ntn2l	1.35018
A_68_P08013981	Foxb1	1.349942
A_68_P07296135	BC003267	1.349777
A_68_P05950428	Smpd1	1.349644
A_68_P13770976	Cdh2	1.349552
A_68_P00406171	Sccpdh	1.348715
A_68_P02110645	Ccbl1	1.348042
A_68_P09101722	Coasy	1.347497
A_68_P03526224	Cda	1.345901
A_68_P07773646	Sorl1	1.345321
A_68_P10057181	Prkcm	1.345045
A_68_P09403409	Gal3st1	1.344925
A_68_P14280238	Pik3ap1	1.343834
A_68_P10336558	Clec14a	1.343737

A_68_P10220901	Cdkl1	1.343247
A_68_P06519231	4933417E01Rik	1.343013
A_68_P09588060	Osm	1.343004
A_68_P04163384	Steap1	1.341706
A_68_P12283480	Zfpm2	1.341623
A_68_P14479477	Adrb1	1.340918
A_68_P06063501	Ap2s1	1.340393
A_68_P05960647	4930451I11Rik	1.339685
A_68_P02298774	Amigo1	1.33957
A_68_P03720734	Mmel1	1.339139
A_68_P05284645	Rbm28	1.338891
A_68_P03759607	1810019J16Rik	1.338293
A_68_P06897654	Slc35e1	1.338042
A_68_P08526444	Ddit3	1.337769
A_68_P08976649	Cnp1	1.3371
A_68_P09609496	Rab5c	1.337036
A_68_P04262918	Zfp316	1.336621
A_68_P00471093	Rgs7	1.336466
A_68_P07921855	Tmem30a	1.336448
A_68_P12262296	Upk3a	1.33564
A_68_P13155345	A_68_P13155345	1.33554
A_68_P10561599	Prss16	1.334579
A_68_P10997971	Hist1h4i	1.334085
A_68_P03324692	H6pd	1.332751
A_68_P00418602	Adora1	1.332424
A_68_P11564485	Prrxl1	1.332232
A_68_P01293034	4833422F24Rik	1.331791
A_68_P07688663	Atm	1.331726
A_68_P03978053	Grhl3	1.331699
A_68_P03720734	Mmel1	1.331498
A_68_P02251909	Uckl1	1.331232
A_68_P00303681	Igfbp5	1.331168
A_68_P07842272	Usp4	1.330904
A_68_P08817669	Sgk	1.330577

Supplementary Table 4: 163 common genes between the genes hypomethylated in KOA offspring compared with KO offspring and those, that were hypermethylated in the KO offspring compared with N offspring on d16

1110008J03Rik
1110008J03Rik
1110049F12Rik
1500031L02Rik
2700007P21Rik
6230416J20Rik
6230416J20Rik
Abcg8
Abcg8
Acot7
Actg1
Acy1
Adam11
Agxt2l2
AI593442
Aldoa
Ambp
Apoa4
Arhgef19
Arhgef19
Armc5
Athl1
B3gnt8
BC021891
BC021891
BC030396
Bhlhb2
Bnc1
Btbd14a
Capn8
Capns1
Casd1

Ccdc122
Cdgap
Cldn19
Clp1
Clp1
Csnk1g2
Cttnbp2nl
Cxcl1
Cxcl1
Cxcl4
Cybasc3
Cyp4f13
Cyp4f13
Ddx11
Defb25-Rem1
Dgkq
Dio1
Dio1
Dio1
Dio1
Dmrt2
Dnmt3a
Dnmt3a
EG232599
Egfl7
Egfl7
Egfl7
Ehd3
En1
Epb4.1l1
Errfi1
Errfi1
Errfi1
Etf1
Evi5

Evi5
Fbxl10
Fbxl10
Fkbp11
Flrt2
Gnai2
Gnptab
Gpc4
Grhl2
Gtf2h4
Hand2
Hoxb13
Hoxb2
Hoxb5
Hoxb7
Hoxc13
Hoxd3
Hp1bp3
Hp1bp3
Hsd17b6
Hsf4
Ifi47
Indol1
Indol1
Irak2
Jarid2
Kcnc3
Kdelr1
Lgals3
Lhx1
Lrig3
Lrpprc
Med18
Mid1ip1
Mxi1

Myo1c
Nmt2
Nmt2
Nrn1
Nxt2
Olfr503
Osbpl1a
Palmd
Palmd
Pax7
Pcdh10
Pcdha9
Phf14
Phox2b
Ptprf
Ptprf
Ptprs
Rbm13
Rbm9
Rbms2
Rdx
Rps6ka5
Rps6ka5
Rtn3
Sacm1l
Sema3b
Sh3tc2
Slc12a9
Slc19a1
Slc1a3
Slc37a1
Slc37a1
Slc37a1
Slc7a11
Slc7a6

Smarcc2
Smcr8
Smcr8
Snag1
Snapc2
Sox2
Srebf1
St8sia4
Stx1a
Tagln2
Tbkbp1
Tcfe3
Tcfe3
Timm8a1
Tnfrsf11a
Tpd52
Tsfm
Tsga10-EG623661
Ung
Ypel4
Yy1
Zfp326
Zfp503
Zfp503
Zfp532
Zfp536

Supplementary Table 5

a The number of genes with DNA methylation changes in the liver of KOA offspring compared with N offspring on d16.

	**Number of genes**		
	**Total number**	**DNA hypomethylation**	**DNA hypermethylation**
**d16**	28983	150	1034

b Pathway analysis of genes that were DNA hypermethylated in KOA offspring relative to those in N offspring on d16.

**Pathway**	***P*-values**
beta-Alanine metabolism	3.80E-05
Renal cell carcinoma	1.20E-03
Axon guidance	2.60E-03
PPAR signaling pathway	3.10E-03
Pyruvate metabolism	1.00E-02
Cysteine and methionine metabolism	1.60E-02
Lysosome	2.00E-02
Galactose metabolism	3.30E-02
Tryptophan metabolism	3.50E-02
Fc gamma R-mediated phagocytosis	3.60E-02
Glycolysis / Gluconeogenesis	3.60E-02

Supplementary Table 6a: N vs KOA 1034 hypermethylation genes on d16

Probe Name	Gene Name	Methylation difference value
A_68_P08659099	Atp2b1	5.619649
A_68_P12362697	Gpt1	5.149239
A_68_P08321362	Apon	4.589806
A_68_P05887814	Echs1	4.528749
A_68_P11542106	Itih1	4.368549
A_68_P08974352	ENSMUST00000100735.1:-5885	4.026455
A_68_P05932565	Polr2i	3.996813
A_68_P07593867	Slc38a3	3.856302
A_68_P10973374	Slc17a3	3.335603
A_68_P10134551	Lrfn5	3.289013
A_68_P01293034	4833422F24Rik	3.240662
A_68_P03900413	Klhdc7a	3.172417
A_68_P08514851	Nr2e1	3.168675
A_68_P08066253	Slc38a3	3.084246
A_68_P09620647	Dcxr	2.996779
A_68_P04983814	Abcb9	2.95639
A_68_P05416729	Cxcl12	2.87807
A_68_P03307859	Hp1bp3	2.805745
A_68_P08778846	Gja1	2.723943
A_68_P03430349	2010305A19Rik	2.719414
A_68_P05519173	Mkln1	2.689089
A_68_P11972358	Cbx7	2.630877
A_68_P03733137	Rnf186	2.594756
A_68_P08992028	Pnpo	2.593888
A_68_P03900439	Klhdc7a	2.586357
A_68_P06602919	Ucp3	2.554169
A_68_P08122216	Clpx	2.5448
A_68_P03353696	Sdc3	2.542607
A_68_P10766266	Slc12a7	2.527982
A_68_P12303780	Hoxc13	2.511596
A_68_P04376201	Micall2	2.50517
A_68_P00873631	Ube2f	2.491692
A_68_P04970366	Chst12	2.454741

A_68_P12254132	1810015C04Rik	2.439941
A_68_P14652218	ENSMUST00000037596.5:-3006	2.377726
A_68_P08176000	Myo1e	2.370593
A_68_P09620642	Dcxr	2.363741
A_68_P13319154	9530058B02Rik	2.355943
A_68_P05943561	Slc27a5	2.321858
A_68_P08364613	Gstt3	2.285871
A_68_P13070859	S3-12	2.278895
A_68_P04621660	BC003324-6330548G22Rik	2.275243
A_68_P06410148	Zfp710	2.248696
A_68_P13374816	Zbtb12	2.232155
A_68_P05307915	Adipor2	2.200657
A_68_P12651548	Pla1a	2.188295
A_68_P13360070	A_68_P13360070	2.185616
A_68_P11937882	BC030396	2.179033
A_68_P06121225	Dll3	2.178701
A_68_P03123565	She	2.171821
A_68_P07210491	F10	2.154816
A_68_P03375546	Slc25a34	2.14218
A_68_P03496599	Il22ra1	2.105849
A_68_P04010940	Foxd2	2.087417
A_68_P07688624	Gramd1b	2.080436
A_68_P11040896	Peci	2.078433
A_68_P04081654	Dio1	2.069482
A_68_P07238996	Ell	2.069469
A_68_P09257037	ENSMUST00000083595.1:-5713	2.068223
A_68_P09628447	Arfl4	2.065944
A_68_P01219086	Pck1	2.053895
A_68_P03496599	Il22ra1	2.036504
A_68_P04390199	Ung	2.026332
A_68_P02145945	Clp1	2.024907
A_68_P15077935	Sox3	2.022275
A_68_P08005788	Odf3l1	2.01059
A_68_P09103592	Igf2bp1	1.995923
A_68_P00945758	Cacna1e	1.980567

A_68_P15077927	Prkx	1.970641
A_68_P00783024	Farp2	1.964128
A_68_P06677187	Agt	1.956564
A_68_P10666960	Jarid2	1.955235
A_68_P02110645	Ccbl1	1.950816
A_68_P04324543	Nat8l	1.946424
A_68_P07622451	Apoc3	1.940699
A_68_P04835083	Fbxw8	1.939241
A_68_P13107839	Abcg8	1.938998
A_68_P09669340	Rab11fip4	1.937035
A_68_P00880269	Zfand2b	1.936263
A_68_P02504542	Zfp697	1.931372
A_68_P11180833	Itih3	1.924031
A_68_P09252755	ENSMUST00000100735.1:-6717	1.922781
A_68_P00458829	Rab17	1.920212
A_68_P06166301	Sphk2	1.91819
A_68_P09295953	Pctp	1.912085
A_68_P01309646	Gzf1	1.884655
A_68_P07444192	Indol1	1.883396
A_68_P01342780	Ccbl1	1.881844
A_68_P01359867	Nxt1	1.879403
A_68_P04914473	Rac1	1.870577
A_68_P09455667	Clk4	1.869642
A_68_P07361088	Rhou	1.869463
A_68_P13172382	Rhot2	1.868646
A_68_P07178590	Sf3b3-Cog4	1.865421
A_68_P13107615	Ticam1	1.863384
A_68_P10766271	Slc12a7	1.855035
A_68_P04983819	Abcb9	1.854886
A_68_P06519231	4933417E01Rik	1.851113
A_68_P08183902	Hyal1	1.850573
A_68_P14987574	Sms	1.84811
A_68_P07697198	Acaa1a	1.84407
A_68_P03896383	Prdm16	1.841264
A_68_P07282392	Ier2	1.841222

A_68_P03987047	Arhgef19	1.838936
A_68_P12051107	Naprt1	1.837565
A_68_P00662466	Il1r1	1.832005
A_68_P02283357	Schip1	1.831677
A_68_P00041396	Mgat4a	1.830827
A_68_P06041220	0610012D14Rik	1.823279
A_68_P03797750	Cdc42	1.821291
A_68_P14844618	Hmgb3	1.81758
A_68_P03396713	Cpne3-2410005O16Rik	1.798519
A_68_P01628111	Dab2ip	1.797675
A_68_P07023164	Sin3b	1.796968
A_68_P01647847	Atp5e	1.794192
A_68_P08416674	Pbld	1.791727
A_68_P06368733	Tnfrsf23	1.785241
A_68_P09134930	Scn4a	1.784074
A_68_P12156053	Tigd5	1.782759
A_68_P08965912	Adora2a	1.782103
A_68_P03466723	Txlna	1.775795
A_68_P15086158	Bcor	1.774253
A_68_P02848277	1100001I22Rik	1.772046
A_68_P09529779	Smarcd2-Tcam1	1.769939
A_68_P02335077	Dpyd	1.766849
A_68_P04389279	Fscn1	1.763812
A_68_P06485913	Mtap6	1.763041
A_68_P14259005	AI450540	1.760722
A_68_P07467134	Slc38a3	1.760431
A_68_P09289118	Aldh3a2	1.758631
A_68_P03733127	Rnf186	1.757996
A_68_P10435877	Matn3	1.755564
A_68_P08533341	Nodal	1.753933
A_68_P08415566	Mettl7b	1.752933
A_68_P09669852	Car10	1.752597
A_68_P02127773	2310047O13Rik	1.752025
A_68_P14420129	Ldb1	1.751049
A_68_P09304807	Stac2	1.745528

A_68_P12204213	Sh3bp1	1.742389
A_68_P01015220	AA408296	1.735834
A_68_P01406624	Bmf	1.734595
A_68_P13977860	Pcdhga5	1.734358
A_68_P11960753	Pick1	1.73337
A_68_P08736561	Atp2b1	1.73138
A_68_P04113179	P2rx4	1.73121
A_68_P04709688	D430033A06Rik	1.728483
A_68_P14934620	Bex4	1.727452
A_68_P07946875	Mst1	1.726311
A_68_P00372595	Agxt	1.724205
A_68_P13425194	Acat3	1.723472
A_68_P13586042	C3	1.720933
A_68_P10554343	Pik3r1	1.720797
A_68_P14768398	Apex2	1.718605
A_68_P06739164	Wwc2	1.718489
A_68_P13594309	Lipg	1.718368
A_68_P04881066	Gatad1	1.716646
A_68_P09245911	Ifi47	1.714575
A_68_P07339397	4921524J17Rik	1.714267
A_68_P06137550	Strn4	1.713789
A_68_P00316905	Tcfcp2l1	1.712471
A_68_P12412293	Irak4	1.71057
A_68_P11522706	Clu	1.706848
A_68_P09582270	2010305C02Rik	1.700087
A_68_P00904876	Tsn	1.696888
A_68_P13186432	Kcnk12	1.696273
A_68_P08600139	Sesn1	1.695192
A_68_P10477201	Sfxn1	1.693759
A_68_P12046833	ENSMUST00000083630.1:-3976	1.693683
A_68_P04113179	P2rx4	1.691899
A_68_P09420564	Lrrc46	1.691015
A_68_P06049341	Egln2	1.690497
A_68_P13201106	Jmjd2b	1.688388
A_68_P10002168	Dpf3	1.68526

A_68_P07364190	Dcun1d2	1.684255
A_68_P09186162	Socs7	1.683958
A_68_P10526799	Pik3r1	1.681965
A_68_P04546813	Slc2a9	1.680349
A_68_P06160078	1600014C10Rik	1.679461
A_68_P03160397	Mab21l1	1.67338
A_68_P07054328	Thap11	1.670121
A_68_P06293260	Mir16	1.66929
A_68_P03296751	Klhl9	1.668838
A_68_P00904213	Asnsd1	1.668315
A_68_P07046503	Nfix	1.667995
A_68_P11558065	Dhrs4	1.667942
A_68_P06168319	Isg20l1	1.667232
A_68_P04349851	Lrch4	1.666799
A_68_P11980858	Arid2	1.663889
A_68_P06397866	Ldha	1.661735
A_68_P01214326	Nmt2	1.661045
A_68_P08185653	BC033915	1.66055
A_68_P11132687	Ppp3cc	1.660509
A_68_P03379272	Mxra8	1.659388
A_68_P09606302	Ern1	1.65789
A_68_P01982873	BC066135	1.655947
A_68_P12045187	AK162044	1.65129
A_68_P09582284	2010305C02Rik	1.651021
A_68_P11534471	Lgals3	1.648572
A_68_P14217831	Klf9	1.647805
A_68_P11564443	9430077A04Rik-Prrxl1	1.647322
A_68_P07078935	Atbf1	1.646868
A_68_P04290359	Xrcc2	1.646374
A_68_P04668612	Qdpr	1.645641
A_68_P13155345	A_68_P13155345	1.644135
A_68_P04081647	Dio1	1.644129
A_68_P04014066	Mfsd2	1.642711
A_68_P03995316	Decr1	1.642297
A_68_P09176959	Il13	1.638504

A_68_P04316025	Arhgap24	1.638175
A_68_P04286123	Art3	1.637273
A_68_P04650365	Ptpn11	1.637044
A_68_P03747731	Ptprf	1.634508
A_68_P01587241	Egfl7	1.633844
A_68_P13196770	1110002E23Rik-Frs3	1.632658
A_68_P01251863	Notch1	1.632458
A_68_P10037181	Rtn1	1.629815
A_68_P03513611	St3gal3	1.628439
A_68_P06848458	Dnajb1	1.627064
A_68_P06048161	Pak4	1.626297
A_68_P12465455	Kdelr3	1.625902
A_68_P04316294	Mapre3	1.62544
A_68_P10336568	Clec14a	1.625074
A_68_P09153383	Ern1	1.62192
A_68_P14385433	Pik3ap1	1.621597
A_68_P09694962	Hn1	1.618952
A_68_P05343553	Pde3a	1.618652
A_68_P05872318	Scnn1a	1.616043
A_68_P08713345	Apof	1.614903
A_68_P06873958	Ranbp10	1.614498
A_68_P07193768	Cdh13	1.61078
A_68_P03805415	Ambp	1.610693
A_68_P07697209	Acaa1a	1.610606
A_68_P06061321	Kdelr1	1.609645
A_68_P06473014	Slc27a5	1.609588
A_68_P13614662	Slc4a9	1.60863
A_68_P10927042	Bmp6	1.608036
A_68_P06962856	Lig4	1.607312
A_68_P15175265	Tcfe3	1.607124
A_68_P14458023	Prkg1	1.605639
A_68_P08195648	Glb1	1.604787
A_68_P08595238	2310011J03Rik	1.604341
A_68_P00317108	Cxcr4	1.604316
A_68_P14501828	Fads1	1.60073

A_68_P07326865	Cyb5b	1.599305
A_68_P06914051	Lsm4	1.598252
A_68_P07946873	Mst1	1.598229
A_68_P08204430	Timp3	1.597375
A_68_P09361410	Alox15	1.597205
A_68_P13147684	Rftn1	1.596753
A_68_P09820901	Rin3	1.59658
A_68_P04449097	Suds3	1.596226
A_68_P14127854	Nol4	1.595347
A_68_P06727821	BC019943	1.594963
A_68_P10147998	Ppp2r5c	1.594754
A_68_P06804280	Irf2	1.594748
A_68_P01603452	C630035N08Rik	1.593115
A_68_P13112327	Gabbr1	1.591262
A_68_P02501124	Nexn	1.591232
A_68_P06221701	Bag3	1.590427
A_68_P08625141	Rab32	1.590396
A_68_P07684876	Tgm4	1.589459
A_68_P05108100	Slc2a3	1.588012
A_68_P12958415	Alg3-1810009K13Rik	1.586551
A_68_P00828037	AW822216	1.586416
A_68_P01293546	A530013C23Rik	1.586085
A_68_P10481288	Gcnt2	1.584906
A_68_P09975941	Crip1	1.584701
A_68_P07117078	Cbfa2t3h	1.58355
A_68_P09663278	Rpl23a-Rab34	1.582134
A_68_P00582015	Mdm4	1.581982
A_68_P08454871	Nodal	1.581463
A_68_P06300520	Bccip	1.580495
A_68_P08191110	Ifrd2	1.578902
A_68_P04976323	Cdk8	1.576476
A_68_P12771414	Dcbld2	1.576329
A_68_P12843197	Ehhadh	1.57599
A_68_P05117228	Anxa4	1.575755
A_68_P11311189	Lrtm1	1.571723

A_68_P02798123	Slc7a11	1.571652
A_68_P08450520	Hkdc1	1.570532
A_68_P09288533	Slc36a2	1.569649
A_68_P06844279	Tom1	1.566898
A_68_P09613181	Coro6	1.566772
A_68_P11551197	Ccdc25	1.566719
A_68_P07517444	Apoa1	1.565848
A_68_P07505359	Eif3s4	1.563028
A_68_P10685829	Barx1	1.562496
A_68_P05754477	C1rl	1.561867
A_68_P10035212	Zfyve1	1.561049
A_68_P14742409	Pdzx	1.560744
A_68_P07513274	Pcolce2	1.560314
A_68_P15013698	Las1l	1.560176
A_68_P05946610	Tead1	1.560166
A_68_P12355067	BC084730:-1892	1.560033
A_68_P03514184	Srm	1.558492
A_68_P06327205	Kcnc3	1.557531
A_68_P12162353	Ldoc1l	1.557113
A_68_P10702707	Slc35d2	1.556992
A_68_P03747745	Ptprf	1.553534
A_68_P05307920	Adipor2	1.553371
A_68_P13847095	Brunol4	1.552841
A_68_P08129723	Qtrt1	1.552486
A_68_P08173092	Cyp8b1	1.55231
A_68_P02251909	Uckl1	1.549621
A_68_P01519278	1700037H04Rik	1.549541
A_68_P14830743	Gnl3l	1.54906
A_68_P12677558	Mrap	1.54899
A_68_P04761491	Sds	1.548369
A_68_P07400685	Vac14	1.546575
A_68_P05345709	Magi1	1.546434
A_68_P05500620	Stk31	1.543976
A_68_P09283753	Spata20	1.543006
A_68_P08500787	Slc16a10	1.542564

A_68_P14568988	Rnf12	1.541526
A_68_P09028343	D11Lgp2e	1.540929
A_68_P08694627	Dna2l	1.540804
A_68_P04742466	Ywhag	1.540448
A_68_P12074308	Galr3	1.540292
A_68_P08715400	Fyn	1.539389
A_68_P03581859	St3gal3	1.538098
A_68_P06035787	Tm6sf1	1.538059
A_68_P04316025	Arhgap24	1.537882
A_68_P04422939	Ssh1-Dao1	1.537264
A_68_P07666475	Pfkfb4	1.536921
A_68_P04504900	Hsd17b13	1.534446
A_68_P03332180	Ttll10	1.533974
A_68_P00625151	Centg2	1.533892
A_68_P04455536	Dr1	1.533527
A_68_P14224733	Map4k2	1.53245
A_68_P04504900	Hsd17b13	1.532262
A_68_P04000868	Rnf207-Rpl22	1.531211
A_68_P11685275	Galntl2	1.530369
A_68_P03203839	Cort	1.530245
A_68_P09329905	Sp2	1.529679
A_68_P07869529	Cep70	1.529645
A_68_P12451528	Atf7	1.529564
A_68_P12276707	Tmem74	1.529366
A_68_P12306344	Krt18	1.529062
A_68_P08474950	Mtap7	1.528491
A_68_P03912425	Aqp7	1.528408
A_68_P14706450	Diap2	1.527589
A_68_P10499714	Irf4	1.526752
A_68_P03464515	Sdhb	1.526331
A_68_P14393451	Ndufb8	1.525609
A_68_P14612493	Pnck	1.525148
A_68_P09817295	Dlst	1.525118
A_68_P04936402	Slc30a9	1.523606
A_68_P04901404	Tmem142a	1.523285

A_68_P05433825	Gm839	1.522913
A_68_P13879820	Slc23a1	1.522039
A_68_P01137518	Niban	1.52036
A_68_P05678119	Inmt	1.519093
A_68_P08321588	BC025920	1.518189
A_68_P15039755	Zmym3	1.517238
A_68_P00493944	Hs6st1	1.514766
A_68_P03881913	D530005L17Rik	1.513696
A_68_P03463892	Srrm1	1.51265
A_68_P09982964	2410016O06Rik	1.51263
A_68_P00186067	A830006F12Rik	1.511897
A_68_P01293529	A530013C23Rik	1.511345
A_68_P09089981	Tax1bp3	1.511277
A_68_P03203839	Cort	1.508423
A_68_P06655108	Lrdd	1.506802
A_68_P11169779	Ndst2	1.506502
A_68_P14897879	Gprasp1	1.505858
A_68_P11918182	Galr3	1.50571
A_68_P11736607	Slc25a37	1.505709
A_68_P06683149	Gtl3	1.505077
A_68_P03232913	Pgm2	1.504647
A_68_P03720734	Mmel1	1.504332
A_68_P01176002	Clp1	1.503881
A_68_P07306669	Car5a-Banp	1.503682
A_68_P06422253	Zfp668-Zfp646	1.503321
A_68_P10259565	Rgs6	1.503003
A_68_P03332180	Ttll10	1.502303
A_68_P05885085	Gfpt1	1.50197
A_68_P03832001	Pax5	1.501002
A_68_P04551716	Crybb3	1.500762
A_68_P00372598	Agxt	1.499331
A_68_P04909167	D5Wsu178e	1.49895
A_68_P11564485	Prrxl1	1.498428
A_68_P02657234	Car3	1.49833
A_68_P04909167	D5Wsu178e	1.497502

A_68_P03899556	Thap3	1.497086
A_68_P04271280	Mdh2	1.49693
A_68_P08357112	Trhde	1.496788
A_68_P04267219	Emid2	1.495906
A_68_P11653564	Rhobtb2	1.495857
A_68_P08817448	D10Jhu81e	1.495613
A_68_P04948730	Cops4	1.495533
A_68_P11079538	Sirt5	1.494837
A_68_P10046450	Serpina5	1.494418
A_68_P08555474	Atoh7	1.492932
A_68_P10113821	Jundm2	1.492866
A_68_P09025065	Ugp2	1.490674
A_68_P09937357	Flrt2	1.490661
A_68_P02938129	Ctbs	1.490448
A_68_P09845387	Ppp1r13b	1.48983
A_68_P08824256	Traf3ip2	1.489383
A_68_P14330355	Rnaseh2c	1.48891
A_68_P09565021	D11Ertd636e	1.488711
A_68_P01561177	Dennd1a	1.488701
A_68_P03634721	Ccdc21	1.487729
A_68_P03727929	Trp73-Wdr8	1.487154
A_68_P09379981	Smarcd2-Tcam1	1.487094
A_68_P08834117	Ddit4	1.487061
A_68_P07214031	Tnrc9	1.486875
A_68_P03518600	Pcsk9	1.486867
A_68_P14704391	Cited1	1.486007
A_68_P06364198	Zfp30	1.485856
A_68_P14702909	Tro	1.485688
A_68_P14047108	Psd2	1.485627
A_68_P08888274	Cd63	1.48559
A_68_P07947580	Tdgf1-Lrrc2	1.485325
A_68_P08783495	Cirbp	1.484897
A_68_P08980029	Krt27	1.484247
A_68_P01628078	Dab2ip	1.484042
A_68_P07256667	D030016E14Rik	1.483922

A_68_P03899301	Zbtb8	1.483826
A_68_P12138338	Hoxc13	1.483194
A_68_P10499724	Irf4	1.48285
A_68_P11456818	Slc25a37	1.482026
A_68_P05640423	Ldhb	1.481345
A_68_P08526773	Ltv1	1.480891
A_68_P14278546	Clcf1	1.480356
A_68_P05988526	Dkkl1-Tead2	1.479889
A_68_P03852202	Tbc1d2	1.479596
A_68_P09063281	Itgb4	1.479567
A_68_P11132853	5730469M10Rik	1.478402
A_68_P04365068	Klb	1.477975
A_68_P04026755	Adprhl2	1.477906
A_68_P09409119	Gprc5c	1.477774
A_68_P09214836	Mpdu1	1.47771
A_68_P03654137	St3gal3	1.476231
A_68_P04590703	Stx1a	1.475218
A_68_P06656873	Gdpd5	1.474837
A_68_P12268481	Aqp2	1.47469
A_68_P03176909	6530418L21Rik	1.474504
A_68_P10960939	Cd83	1.47429
A_68_P01906267	Sohlh1	1.473745
A_68_P12074358	Pycrl	1.472776
A_68_P10667411	Mylip	1.471582
A_68_P10406991	4831426I19Rik	1.471236
A_68_P09467086	Ints2	1.470856
A_68_P00327181	Lemd1	1.470616
A_68_P14368537	Ankrd1	1.470332
A_68_P03896713	Cdkn2c	1.470126
A_68_P03935411	Aqp3	1.469318
A_68_P09166156	Tmc6-Tmc8	1.468933
A_68_P06085388	Ccne1	1.468697
A_68_P01943462	Ctsz	1.468533
A_68_P00072271	Ptpn14	1.468269
A_68_P13359207	Lrpprc	1.468152

A_68_P09193111	Eif1	1.467945
A_68_P01804429	Pxmp4-Zfp341	1.467591
A_68_P05979604	Lsr	1.467481
A_68_P13176455	Galnt14	1.467415
A_68_P07124748	Lypla3	1.466889
A_68_P04071366	Plaa	1.466764
A_68_P09153352	Rnf185	1.465903
A_68_P03505622	E2f2	1.464979
A_68_P12877631	Ahsg	1.464225
A_68_P11111057	Tcfap2a	1.463548
A_68_P08377706	Tle6	1.462789
A_68_P05872300	Scnn1a	1.461799
A_68_P10766257	Slc12a7	1.461562
A_68_P01092733	Lbr	1.460251
A_68_P11867189	Pfdn5	1.460001
A_68_P10437458	Slc35b3	1.459885
A_68_P11911303	Fkbp11	1.459781
A_68_P11209114	Camk2g	1.459623
A_68_P07738687	Ncam1	1.459108
A_68_P00273226	EG240916	1.458418
A_68_P02986715	Tmem144	1.457984
A_68_P11288375	Grid1	1.457847
A_68_P13319163	9530058B02Rik	1.457545
A_68_P14814452	Gripap1	1.457428
A_68_P14280238	Pik3ap1	1.457419
A_68_P05285116	Cpa2	1.457155
A_68_P05959998	Lmtk3	1.456797
A_68_P04026755	Adprhl2	1.45652
A_68_P12056452	Slc39a4	1.456413
A_68_P09656833	Agxt2l2	1.455604
A_68_P03795291	Pink1	1.455548
A_68_P13411229	Atp6v1e2	1.455289
A_68_P08446465	Ncln	1.455214
A_68_P08339442	Sar1a	1.454726
A_68_P07561489	2310005P05Rik	1.454545

A_68_P00418602	Adora1	1.454265
A_68_P13921216	Kif20a	1.453341
A_68_P05904990	C330005M16Rik	1.452912
A_68_P07950325	Sh3px3	1.452875
A_68_P10669255	Agtpbp1	1.452506
A_68_P13620460	LOC639653	1.451731
A_68_P00082872	Prei3	1.451494
A_68_P15004426	Armcx3	1.451056
A_68_P13161337	Rpl10a	1.450924
A_68_P10093139	Gm1568	1.450446
A_68_P08085317	Map2k5	1.450197
A_68_P09154524	Cog1	1.45015
A_68_P08110716	Acp5	1.449856
A_68_P09317362	2310067B10Rik	1.449475
A_68_P12092114	Krt79	1.44944
A_68_P14794097	Zic3	1.449335
A_68_P02590895	BC028528	1.44907
A_68_P04825881	Fbxl10	1.448834
A_68_P08350042	Hmha1	1.448409
A_68_P11215454	Olfm4	1.448167
A_68_P10959245	Tcfap2a	1.448068
A_68_P04393156	Trafd1-C330023M02Rik	1.447876
A_68_P05715101	Xpc	1.447498
A_68_P12677554	Mrap	1.44726
A_68_P06682522	Herpud1	1.447101
A_68_P00346078	Selp	1.446255
A_68_P01704832	Rtn4rl2	1.446219
A_68_P07946225	Dnmt1	1.446069
A_68_P12636173	Lsg1	1.446016
A_68_P01853107	Zbtb43	1.445889
A_68_P13059000	Hmga1	1.445519
A_68_P09349429	Pscd1	1.445206
A_68_P05495655	Znhit4	1.445202
A_68_P06784221	Ppp2cb	1.44518
A_68_P00976261	Capn2	1.443754

A_68_P05919592	Dctn5	1.442914
A_68_P04316598	Tesc	1.442183
A_68_P14148146	Zdhhc6	1.44207
A_68_P13322670	Prr3	1.44174
A_68_P01334676	9230107O10Rik	1.441045
A_68_P14104697	Cul2	1.440976
A_68_P04803447	Afp	1.44087
A_68_P12669014	Pcqap	1.440071
A_68_P01997700	Lsm14b	1.438893
A_68_P08779365	Zbtb24	1.438717
A_68_P03087427	Pklr	1.438709
A_68_P09119456	Spata20	1.438591
A_68_P08991556	Mrps24	1.438477
A_68_P07559432	D930028F11Rik	1.43747
A_68_P09115945	BC022224	1.43716
A_68_P05161860	Dusp11	1.436969
A_68_P05448315	Kcna5	1.436499
A_68_P13695623	Ablim3	1.436244
A_68_P13499441	Slc29a1	1.436126
A_68_P03428697	Dffa	1.435921
A_68_P09108875	Aatk	1.435872
A_68_P04030079	1700003M02Rik	1.435819
A_68_P04742466	Ywhag	1.435035
A_68_P01918764	Sdc4	1.434142
A_68_P02480010	Bmpr1b	1.433834
A_68_P08817485	D10Jhu81e	1.433749
A_68_P04568812	Hdh	1.433407
A_68_P12659824	Sema5b	1.432966
A_68_P11949856	Arhgap8	1.432242
A_68_P12256637	Lifr	1.43218
A_68_P11235583	Ccdc122	1.431807
A_68_P01075343	Nmur1	1.431739
A_68_P03602285	9930104L06Rik	1.431719
A_68_P10718192	Id4	1.431203
A_68_P08376605	Lrrc3	1.430762

A_68_P01182959	Nfatc2	1.430751
A_68_P04125045	Prom1	1.430601
A_68_P12175545	Galr3	1.43003
A_68_P04323144	Sult1d1	1.429318
A_68_P05251168	8430408G22Rik	1.429266
A_68_P13929227	Wnt8a	1.429108
A_68_P12293869	Cyp2d26	1.429025
A_68_P14842575	Ube1x	1.428913
A_68_P04871762	Gprk2l	1.428672
A_68_P04698270	Fndc4	1.428474
A_68_P10942741	Tmed9	1.427877
A_68_P04107067	Rpl21	1.427654
A_68_P00170655	4931440L10Rik-Jarid1b	1.427606
A_68_P08713341	Apof	1.427588
A_68_P14836142	Trex2	1.427525
A_68_P03899556	Thap3	1.427412
A_68_P01944480	4930526D03Rik	1.4271
A_68_P09718198	Sphk1	1.427011
A_68_P04909159	D5Wsu178e	1.426943
A_68_P09697832	Sec14l4	1.426688
A_68_P01182844	Slc30a4	1.426611
A_68_P03405759	Txlna	1.426604
A_68_P09398667	2210020M01Rik	1.426216
A_68_P03709371	Plcl4	1.426053
A_68_P10323285	Rps6ka5	1.424931
A_68_P07299665	Nfix	1.424464
A_68_P11477858	Tpt1	1.424277
A_68_P01173674	2010011I20Rik	1.423893
A_68_P08184605	Rbp2	1.422313
A_68_P09410666	Ankrd43	1.421737
A_68_P11308601	Tgm1	1.421416
A_68_P00396252	Hhat	1.421254
A_68_P02944888	Prune	1.421197
A_68_P05678030	Hoxa11	1.421079
A_68_P00864420	Lefty1	1.420885

A_68_P00992012	Lemd1	1.420739
A_68_P08138672	AI593442	1.42035
A_68_P10628441	F2rl1	1.420108
A_68_P01337232	Ptgis	1.419762
A_68_P06857332	Hmox1	1.41975
A_68_P10556434	Rnf44-BC040758	1.419585
A_68_P02653596	4632404H12Rik-Ube2q1	1.419406
A_68_P04904125	Pdgfa	1.419133
A_68_P06212420	Egln2	1.418662
A_68_P04121317	Tmem130	1.416783
A_68_P04899482	Brap	1.416621
A_68_P15216275	Efnb1	1.41555
A_68_P04397082	Cops6	1.415353
A_68_P07513276	Pcolce2	1.415076
A_68_P07218229	Slc12a4	1.413568
A_68_P04306177	Crcp	1.413382
A_68_P12240032	Eppk1	1.412875
A_68_P13975465	Pcdha3	1.41283
A_68_P06390438	AI428936	1.412436
A_68_P13594311	Lipg	1.412391
A_68_P06583812	Zfp84	1.412358
A_68_P03466723	Txlna	1.412004
A_68_P04899482	Brap	1.411886
A_68_P01127569	Tsga10-EG623661	1.411843
A_68_P06554264	Ndnl2	1.411804
A_68_P10113544	Bag5	1.411329
A_68_P06098719	Zfp579	1.411065
A_68_P08161575	Acpp	1.411036
A_68_P07775112	Phldb1	1.40924
A_68_P10913242	Wrnip1	1.409125
A_68_P06926959	Nob1	1.40853
A_68_P07444231	Nanos3	1.408003
A_68_P01668312	E2f1	1.407714
A_68_P07720142	Fxyd6	1.407381
A_68_P10372966	Hif1a	1.407126

A_68_P04435271	Man2b2	1.406616
A_68_P09593309	OTTMUSG00000000934-1700020L24Rik	1.406483
A_68_P08068605	Tpm1	1.406398
A_68_P12979239	Ppl	1.40558
A_68_P08395271	Smarcc2	1.405527
A_68_P06020155	Slc22a18	1.405125
A_68_P01506207	Ndor1-C730025P13Rik	1.404116
A_68_P10509311	AU042651	1.40399
A_68_P12871002	Sema5b	1.403742
A_68_P03494298	1110020C03Rik	1.403589
A_68_P06247672	5430432N15Rik	1.403439
A_68_P06907386	Mrps31	1.403207
A_68_P09078461	Anapc11	1.403101
A_68_P07134632	Rbm35b	1.402672
A_68_P13797523	Pcdhga6	1.402619
A_68_P09534568	Galk1	1.402563
A_68_P11507565	Pcdh17	1.402404
A_68_P10960928	Cd83	1.401914
A_68_P01772350	Lcn12	1.401837
A_68_P12256635	Lifr	1.401321
A_68_P03533898	Nbl1	1.401021
A_68_P11031496	Klf6	1.40099
A_68_P08052081	Sema3b	1.400213
A_68_P08817347	Upb1	1.400141
A_68_P14814429	Gripap1	1.399972
A_68_P14022680	Matr3	1.399952
A_68_P04671642	Unc84a	1.399699
A_68_P13348089	Slc29a1	1.39949
A_68_P04489999	Tchp	1.399278
A_68_P01081177	Slco5a1	1.399243
A_68_P09469784	Nefh	1.398879
A_68_P12462766	Top1mt	1.3988
A_68_P13874169	Rps14	1.398646
A_68_P14386452	Naaladl1	1.398299

A_68_P13175446	Haghl	1.398104
A_68_P13173432	Decr2	1.397833
A_68_P04893782	Snx8	1.397799
A_68_P07829944	Paqr9	1.397399
A_68_P11000859	Lman2-Rgs14	1.397116
A_68_P04761491	Sds	1.396979
A_68_P10354469	Jag2	1.396256
A_68_P03595059	Slc2a1	1.395236
A_68_P10640236	Nid1	1.39522
A_68_P06609275	Dmn	1.394924
A_68_P14458284	Slc3a2	1.39487
A_68_P03811052	4732473B16Rik	1.394516
A_68_P09618735	Aldoc	1.394351
A_68_P05514834	4921507P07Rik	1.394225
A_68_P02810083	ENSMUST00000083570.1:-374	1.393739
A_68_P02173326	Smtnl1-Timm10	1.393303
A_68_P13291042	Fkbpl	1.393011
A_68_P04003810	Gpx7	1.39278
A_68_P12052843	AW549877	1.392723
A_68_P08425563	Lss	1.392357
A_68_P09177531	Atp5g1	1.392234
A_68_P05954798	Tmem160	1.391956
A_68_P09539387	Tnfsf12	1.39175
A_68_P11890247	Rbm9	1.391445
A_68_P01251035	Gfra4	1.391445
A_68_P09628737	Meis1	1.391253
A_68_P08054984	Rbpms2	1.390078
A_68_P08980723	4933414I15Rik	1.389525
A_68_P06124550	Ifitm6	1.389417
A_68_P00640827	Adipor1	1.389397
A_68_P14737878	Ppp1r3f	1.38934
A_68_P08546101	Rdh7	1.389291
A_68_P14765505	Eda	1.389147
A_68_P07485273	D930028F11Rik	1.389121
A_68_P02452306	Lxn	1.388964

A_68_P13728082	Lims2	1.388765
A_68_P04756674	Nsun5	1.388709
A_68_P00379196	Slc11a1	1.388581
A_68_P04267219	Emid2	1.388229
A_68_P04202508	2900002H16Rik	1.388114
A_68_P06523791	Zfp94	1.388046
A_68_P00761121	5033414K04Rik	1.387774
A_68_P00641555	Nr1i3	1.387621
A_68_P14323652	Sfxn3	1.387516
A_68_P08043009	ENSMUST00000040025.5:86676	1.387397
A_68_P04600442	Gak-Tmem175	1.387309
A_68_P12927819	Hrg	1.387228
A_68_P10211435	Slc25a29	1.386406
A_68_P03323239	Hsdl2	1.386274
A_68_P13551633	Socs5	1.386213
A_68_P09357796	Rara	1.385957
A_68_P07210532	F10	1.385922
A_68_P06420209	Athl1	1.385804
A_68_P03324692	H6pd	1.385694
A_68_P03533898	Nbl1	1.385539
A_68_P07272472	Zcchc14	1.385187
A_68_P09536877	Pnmt	1.38475
A_68_P04564491	Arpc1a	1.384418
A_68_P14408920	Sart1	1.384268
A_68_P06181223	Abcc6	1.384253
A_68_P13214626	Trip10	1.384146
A_68_P03703507	Srrp	1.383383
A_68_P07924824	Col7a1	1.383194
A_68_P06168316	Isg20l1	1.382971
A_68_P13117097	Bak1	1.382725
A_68_P09525412	Hes7	1.3826
A_68_P14349495	Gna14	1.382571
A_68_P12448089	Csnk1e	1.382542
A_68_P07844432	Tmem25	1.38221
A_68_P03513133	Elovl1	1.382112

A_68_P05591343	Zyx	1.381698
A_68_P07341251	Spata4	1.381511
A_68_P11890938	Slc25a17	1.38148
A_68_P04403726	En2	1.381455
A_68_P00206509	St8sia4	1.381311
A_68_P08464845	ORF61	1.381265
A_68_P03859840	Kif12	1.38122
A_68_P07068098	Lamp1	1.381108
A_68_P14154161	Ina	1.380776
A_68_P01828463	Cobll1	1.380624
A_68_P12199090	4930572J05Rik	1.380609
A_68_P11754145	Mapk8	1.38022
A_68_P12003446	Oxct1	1.380044
A_68_P13367496	Trim26	1.379473
A_68_P01293536	A530013C23Rik	1.379134
A_68_P01574979	Nnat	1.378923
A_68_P07651826	Camkv	1.378867
A_68_P06272860	2410127E16Rik	1.378846
A_68_P14428140	Tlx1	1.378692
A_68_P08043229	BC031353	1.378254
A_68_P00789027	Ankrd39	1.377282
A_68_P00072270	Ptpn14	1.377265
A_68_P13697471	Pcdha5	1.3771
A_68_P00207845	Ncl	1.37703
A_68_P09368961	Sox9	1.37696
A_68_P07723898	Sema3f	1.376629
A_68_P04834757	Cutl1	1.37658
A_68_P11030208	Ddx4-9130023D20Rik	1.376209
A_68_P05933804	Thrsp	1.375919
A_68_P09648521	Hdac5	1.375745
A_68_P13075872	Mas1-Mrgprh	1.3757
A_68_P14228457	Rrp12	1.375458
A_68_P12150006	Kif21a	1.375324
A_68_P00227415	Osgepl1	1.375116
A_68_P10301168	Tgfb3-1700019E19Rik	1.375113

A_68_P07828149	Rbpms2	1.374737
A_68_P13428964	1700001C19Rik	1.374676
A_68_P11712816	Atp8a2	1.374544
A_68_P14706457	Diap2	1.374526
A_68_P08036926	Chst2	1.374478
A_68_P07629982	Tmed1	1.374422
A_68_P15177716	4933436I01Rik	1.37438
A_68_P02034225	Itga6	1.374306
A_68_P00656928	2500001K11Rik	1.373611
A_68_P11166311	Clu	1.373603
A_68_P02148570	1700123D08Rik	1.373449
A_68_P03059990	Ptgfr	1.373345
A_68_P11855104	5730410E15Rik	1.372925
A_68_P08315623	Ddt	1.3729
A_68_P02004607	Gm1631	1.3727
A_68_P11708828	Rnase10	1.372173
A_68_P06867905	Slc5a5	1.372124
A_68_P05960647	4930451I11Rik	1.371871
A_68_P06274249	Klf13	1.371567
A_68_P00552869	Capn8	1.371189
A_68_P13867110	Pard6g	1.371174
A_68_P11529416	Mtmr9	1.370879
A_68_P11537416	Nisch-Tnnc1	1.370867
A_68_P05838606	Cd9	1.370573
A_68_P03583518	Thrap3	1.369934
A_68_P08840694	Arid3a	1.369653
A_68_P04346888	Ppp2r2c	1.369525
A_68_P03833504	Pink1	1.369238
A_68_P13871637	Ndst1	1.369179
A_68_P03635372	Plekhf2	1.36905
A_68_P03185907	Enpep	1.368886
A_68_P06314101	Myo7a	1.3688
A_68_P14369063	Scd4	1.36879
A_68_P01619771	Insm1	1.368782
A_68_P07024704	1700029J07Rik	1.368462

A_68_P07678840	2010110K16Rik	1.368421
A_68_P08396865	Crsp3	1.368407
A_68_P07748462	Rpp25	1.368336
A_68_P05732548	Arhgdib-Pde6h	1.368284
A_68_P08485276	Rfx4	1.368004
A_68_P12500292	Epha3	1.36753
A_68_P01164453	Slc24a5	1.367509
A_68_P07503953	Crabp1	1.366513
A_68_P12128709	Racgap1	1.366488
A_68_P11567764	4930578I06Rik	1.366367
A_68_P13126791	Atp6v1g2	1.366292
A_68_P09050623	Bzrap1	1.366273
A_68_P01920530	Uap1l1	1.365508
A_68_P08826802	Hkdc1	1.365491
A_68_P07536331	Lrrn6a	1.365224
A_68_P14899047	Tsc22d3	1.365062
A_68_P04488676	Slc26a1	1.365019
A_68_P13251986	Srrm2	1.364884
A_68_P14153156	Chuk	1.364786
A_68_P07306676	Car5a	1.364733
A_68_P02581370	Wnt2b	1.364599
A_68_P12191245	Dgat1	1.364556
A_68_P12062341	Pla2g6	1.364451
A_68_P01288757	Slc20a1	1.364246
A_68_P13415559	Sepx1	1.364204
A_68_P06122746	Car11	1.364025
A_68_P00719131	Lad1	1.363767
A_68_P08773799	Dnajc14	1.363732
A_68_P03075997	Dcamkl2	1.363686
A_68_P03535794	Tal2	1.363617
A_68_P05541947	Reep1	1.363366
A_68_P12788633	Tmem41a	1.363233
A_68_P06292783	Sae1	1.363202
A_68_P07400446	Snai3	1.363009
A_68_P07089321	Foxl1	1.362602

A_68_P09575212	Rsad1	1.362435
A_68_P06599176	Grin2d-Kdelr1	1.362242
A_68_P05358487	Rassf8	1.362197
A_68_P03721921	Dhcr24	1.361967
A_68_P04420388	Grsf1	1.361566
A_68_P01440416	Fbxw2	1.361542
A_68_P03513105	Elovl1	1.361494
A_68_P13560632	Slc22a7	1.361343
A_68_P07200483	Ier2	1.361322
A_68_P06195728	AY078069	1.361173
A_68_P06115762	Cacng3	1.360879
A_68_P00995269	Atp1b1	1.360712
A_68_P08538267	Gnptab	1.36063
A_68_P04899188	Ubc	1.360629
A_68_P12126766	Tmprss6	1.36032
A_68_P06375734	Ccdc106	1.360018
A_68_P07976988	4733401H18Rik-Dalrd3	1.359907
A_68_P14109314	Osbpl1a	1.35965
A_68_P05119378	Tspan33	1.359378
A_68_P07596637	Vsig2	1.359271
A_68_P08299334	1700021F05Rik	1.359211
A_68_P09638194	Rad51l3	1.359125
A_68_P04979692	9430057O19Rik	1.359009
A_68_P01943459	Ctsz	1.358991
A_68_P01342941	Ass1	1.358922
A_68_P05437345	Rtkn	1.358591
A_68_P05075148	Scnn1a	1.358582
A_68_P02474696	Eltd1	1.358534
A_68_P12638874	Tbx1-4930588K23Rik	1.358483
A_68_P00905137	B3gnt7	1.358442
A_68_P00812329	Pou3f3	1.358345
A_68_P00400075	Kif1a	1.358243
A_68_P09179041	1110067D22Rik	1.358208
A_68_P14605630	Hcfc1	1.358173
A_68_P11319776	Stk24	1.358142

A_68_P12415129	1700019P01Rik	1.357988
A_68_P03176910	6530418L21Rik	1.357851
A_68_P07818473	Eva1	1.357778
A_68_P08530152	Cnn2	1.357437
A_68_P11966159	Pkdrej-AW124722	1.356999
A_68_P03730091	Ube2j2	1.356958
A_68_P07588985	Birc3	1.356853
A_68_P13069277	Zfp161	1.356576
A_68_P11800686	Syt15	1.35645
A_68_P05752699	Impdh1	1.355613
A_68_P01348835	Kynu	1.355327
A_68_P03726231	Ptprf	1.355176
A_68_P14910238	Ids	1.355112
A_68_P12441858	Adamts20	1.354793
A_68_P06470224	Bet1l-Ric8	1.354612
A_68_P08490715	Ggt1	1.354475
A_68_P09250239	Zfp672	1.354305
A_68_P00194128	Cacybp	1.354282
A_68_P00245688	Ttll4	1.35397
A_68_P15159838	Rhox6	1.353884
A_68_P11014513	Otp	1.353793
A_68_P06127908	Pak1	1.353515
A_68_P01378985	Cacnb4	1.353068
A_68_P06872414	Efcbp2	1.35301
A_68_P09349698	2400006H24Rik	1.352811
A_68_P03720734	Mmel1	1.352229
A_68_P04567641	A230097K15Rik	1.352088
A_68_P12713735	Atp5o	1.352055
A_68_P13981763	Mapk4	1.352013
A_68_P10157510	A_68_P10157510	1.351816
A_68_P01345408	Api5	1.351781
A_68_P08903907	Tspan31-Centg1	1.351778
A_68_P09628625	Tmem88	1.351453
A_68_P06984570	Psmd7	1.3512
A_68_P10760640	Gas1	1.35105

A_68_P07401386	B3gnt3	1.35097
A_68_P14061985	Egr1	1.350666
A_68_P05548217	Chd4	1.35064
A_68_P00719128	Lad1	1.350552
A_68_P13349409	Sfrs3	1.35002
A_68_P07074552	Usp38	1.349978
A_68_P06626995	Hmx2	1.34984
A_68_P14461348	Cdca5	1.349516
A_68_P13134961	Tnfrsf21	1.349149
A_68_P08666230	Hnrph3	1.349019
A_68_P05231743	Zfp384	1.348981
A_68_P13556997	Tsga2	1.348651
A_68_P06087354	Pnpla2	1.34814
A_68_P09645952	OTTMUSG00000000934	1.347966
A_68_P09548393	Aoc3	1.347929
A_68_P12242248	Syngr1	1.347821
A_68_P06438476	Saps1	1.347799
A_68_P04546156	Trfr2	1.347261
A_68_P00626532	Atf3	1.347158
A_68_P05679178	Camk1	1.347021
A_68_P04731285	Kl	1.346698
A_68_P09413265	Adam11	1.34647
A_68_P14431117	Sf1	1.346376
A_68_P11247425	Entpd4	1.346079
A_68_P12255657	Baiap2l2	1.345019
A_68_P05228148	H1fx	1.344989
A_68_P09645961	OTTMUSG00000000934	1.344915
A_68_P09582843	Itga3	1.344811
A_68_P13067632	Pkd1	1.344641
A_68_P06393479	A830041P22Rik-BC028440	1.344343
A_68_P14849214	ENSMUST00000037596.5:-5074	1.344303
A_68_P08314943	Oaz1	1.344037
A_68_P12151407	2010109I03Rik	1.344025
A_68_P12018996	Pfkm	1.34388
A_68_P11972173	Rabl4	1.343842

A_68_P09009571	Olfr223	1.343837
A_68_P13834333	Srfbp1	1.343547
A_68_P04966921	Rpl21	1.34327
A_68_P05448304	Kcna5	1.34313
A_68_P09434018	Cntnap1	1.343124
A_68_P08882556	Ptbp1	1.343073
A_68_P13465706	Caskin1	1.342849
A_68_P15092233	Armcx2	1.342413
A_68_P06431967	Siglecf	1.342322
A_68_P06765322	Wwc2	1.342304
A_68_P06275315	Rgs10	1.34221
A_68_P09598068	Acox1	1.34209
A_68_P06184102	Ech1	1.341959
A_68_P02032727	Arl6ip6	1.341798
A_68_P01606835	Prnd	1.341071
A_68_P01986063	Mcm8	1.341042
A_68_P00064328	Mpzl1	1.340992
A_68_P03984305	Nfib	1.340978
A_68_P09359466	Sphk1	1.340949
A_68_P15009258	Gm784	1.340896
A_68_P08066246	Slc38a3	1.340809
A_68_P10492574	Ippk	1.340586
A_68_P03245499	Ripk2	1.340568
A_68_P12451934	Card10	1.340293
A_68_P00451766	Nmnat2	1.340258
A_68_P12449654	Ankrd46	1.34017
A_68_P09136314	Baiap2	1.33999
A_68_P04834404	Lphn3	1.339827
A_68_P07359887	Cox4i1	1.339736
A_68_P03953505	Ppp1r8	1.338938
A_68_P10293201	Sel1l	1.338653
A_68_P05099470	Cpne9	1.338103
A_68_P01963655	4932418E24Rik	1.337979
A_68_P06985598	Pkd1l2	1.337707
A_68_P12908674	Magmas	1.337585

A_68_P13075867	Mas1-Mrgprh	1.337503
A_68_P00991622	Capn8	1.337252
A_68_P03920582	Fgr	1.337216
A_68_P14989894	Ap1s2	1.336982
A_68_P13434054	B3galt4	1.33697
A_68_P13503529	C130040N14Rik	1.336904
A_68_P07574923	Coro2b	1.336902
A_68_P09736098	Hba-a1	1.336646
A_68_P05904665	Hpxn	1.33661
A_68_P02091744	Ncoa5	1.33604
A_68_P04277969	Rnf32	1.336019
A_68_P10974783	Prr7	1.335538
A_68_P09559562	Shmt1	1.335522
A_68_P01722996	Pck1	1.335297
A_68_P13119759	Nkx2-5	1.335287
A_68_P09307716	Doc2b	1.335262
A_68_P01640329	Btbd3	1.334992
A_68_P05891727	Grik5-Zfp574	1.334977
A_68_P08935364	Fyn	1.334879
A_68_P04731732	Sdsl	1.334687
A_68_P08173101	Cyp8b1	1.334479
A_68_P02160813	Inpp5e	1.334385
A_68_P05402372	Itpr2	1.334203
A_68_P02442112	Car14	1.334153
A_68_P00999801	Avpr1b	1.334075
A_68_P11406546	Ang1	1.333952
A_68_P06078807	Zfp446	1.333811
A_68_P01275891	Fign	1.333792
A_68_P03274719	Cd72	1.333666
A_68_P08894637	Tle2	1.333629
A_68_P09538169	D11Ertd18e	1.333536
A_68_P09229843	Sec14l1	1.333494
A_68_P02989786	Neurog2	1.333308
A_68_P07876589	Fbxo9-Ick	1.333285
A_68_P04608378	Rnf6-Cdk8	1.333156

A_68_P03673261	B3galt6	1.333018
A_68_P08073169	Apeh	1.332935
A_68_P02046877	Egfl7	1.332874
A_68_P11311203	Lrtm1	1.332561
A_68_P02305769	Pld1	1.332537
A_68_P11019594	Uimc1-Zfp346	1.33245
A_68_P08629268	Hkdc1	1.33233
A_68_P03854965	Marcksl1	1.332236
A_68_P04636799	Nub1	1.332012
A_68_P05342558	Gfpt1	1.331959
A_68_P14314015	Ehd1	1.331856
A_68_P12871001	Sema5b	1.331684
A_68_P12584046	2310061J03Rik	1.331668
A_68_P01424824	Rbl1	1.331663
A_68_P12017322	Cbx7	1.331613
A_68_P06653585	Plekha4	1.331332
A_68_P06406870	Zik1	1.33098
A_68_P06243313	Klk10	1.33087
A_68_P11609980	Jub	1.330582
A_68_P07010746	Abhd8	1.330312
A_68_P01717991	Btbd14a	1.330029
A_68_P03535794	Tal2	1.330011

Supplementary Table 6b: N vs KOA 150 hypomethylation genes on d16

Probe Name	Gene Name	Methylation difference value
A_68_P12755374	App	0.047829
A_68_P07910003	Elavl3	0.135364
A_68_P12853498	Mx2	0.150429
A_68_P04734427	Shroom3	0.175271
A_68_P01769357	Mrg1	0.187045
A_68_P10449774	2410002O22Rik-Trim23	0.288886
A_68_P12217051	9130401M01Rik	0.292903
A_68_P02404444	Cxxc4	0.305886
A_68_P13251244	Cyp4f13	0.367237
A_68_P09049964	Gria1	0.417974
A_68_P13251243	Cyp4f13	0.420593
A_68_P07592424	Lbxcor1	0.421406
A_68_P14259486	Rfx3	0.425236
A_68_P06810073	Aadat	0.430781
A_68_P14586192	Irs4	0.435363
A_68_P10208303	Brms1l	0.458816
A_68_P12423891	Rnd1	0.465988
A_68_P14659932	Slc25a5	0.481463
A_68_P06177032	Rgs9bp	0.500148
A_68_P05492016	Mkrn1	0.522946
A_68_P12536557	BC027231	0.527749
A_68_P03944620	E130114P18Rik	0.550509
A_68_P01268520	Zfhx1b	0.556625
A_68_P12360632	Zhx2	0.560654
A_68_P04895996	Mapk10	0.561765
A_68_P04277065	Cxcl1	0.561935
A_68_P04480991	Wfs1	0.56214
A_68_P05918908	Peg3	0.571169
A_68_P00552426	Dst	0.572084
A_68_P04262240	Mapk10	0.574887
A_68_P03075963	Rap1gds1	0.57979
A_68_P05122196	Fkbp14-Plekha8	0.58038
A_68_P03545245	Casp8ap2	0.58824

A_68_P04846731	Rasgef1b	0.597842
A_68_P05162900	Zfml	0.602215
A_68_P08629638	Ddt	0.60239
A_68_P13119458	Dscr1l1	0.607516
A_68_P08440358	Ahi1	0.60832
A_68_P11564394	9430077A04Rik	0.612977
A_68_P14628736	6430550H21Rik	0.619227
A_68_P09233032	Anxa6	0.61975
A_68_P07327905	Slc6a2	0.62084
A_68_P09296258	Hspa4	0.637876
A_68_P13992999	Pcdhgb2	0.641232
A_68_P09689225	Wbp2	0.643284
A_68_P03553526	Vamp3	0.645106
A_68_P14001180	Aldh7a1	0.650569
A_68_P09955204	Fut8	0.655423
A_68_P04763153	Rasal1	0.657537
A_68_P04247170	Zfp326	0.658156
A_68_P14292416	Hps6	0.660772
A_68_P11328115	Otx2	0.660979
A_68_P09134526	Lhx1	0.662558
A_68_P03553526	Vamp3	0.666399
A_68_P04277062	Cxcl1	0.673531
A_68_P07704674	Opcml	0.675132
A_68_P14519995	Bmx	0.677734
A_68_P11069910	Gcnt2	0.679741
A_68_P14336511	Arhgap19	0.68037
A_68_P04218037	Tbx3	0.681135
A_68_P09575135	BC025575	0.681867
A_68_P02032707	Prpf40a	0.684072
A_68_P01024689	Mosc2	0.684117
A_68_P09095193	Rhbdd3	0.684994
A_68_P10561224	Fbxl21	0.685629
A_68_P11960760	Pick1	0.687702
A_68_P11825632	Ptprg	0.690785
A_68_P06075223	Aplp1	0.692372

A_68_P00221558	Prrx1	0.692959
A_68_P04844116	Cxcl4	0.694124
A_68_P13657420	Pias2	0.695675
A_68_P00098491	Astn1	0.696062
A_68_P07379781	Tomm20	0.696558
A_68_P11465874	Tsc22d1	0.699961
A_68_P02070587	Zswim3	0.700566
A_68_P00478505	Pax3	0.701502
A_68_P07949632	Gramd1b	0.701855
A_68_P02870031	Tnrc4	0.702077
A_68_P00287878	B3gat2	0.702756
A_68_P06428107	Zfp36	0.705494
A_68_P07432747	E130303B06Rik	0.706874
A_68_P14103036	Gm672	0.708007
A_68_P13625730	Cbln2	0.711191
A_68_P13166533	Ddah2	0.713225
A_68_P06633063	Stk32c	0.714689
A_68_P14209573	Rin1	0.715253
A_68_P08111988	Armet	0.716054
A_68_P03211661	2610204G22Rik	0.717515
A_68_P03780585	Arhgef19	0.717638
A_68_P13365467	Rab11b	0.719827
A_68_P03704070	Dlgap3	0.720437
A_68_P09782980	Kcnf1	0.72053
A_68_P12157937	Cacnb3	0.721433
A_68_P06660297	Zfp536	0.721824
A_68_P03676855	Zfp189	0.721846
A_68_P02733796	2010200O16Rik-Taf13	0.722795
A_68_P02244930	Zfp533	0.723196
A_68_P01529996	Fnbp4	0.723251
A_68_P06099522	Apoc1	0.725501
A_68_P04040117	4732473B16Rik	0.725666
A_68_P14844556	Rs1	0.72603
A_68_P08462448	Hint3	0.726933
A_68_P12636185	Lsg1	0.726944

A_68_P07827956	Tbx20	0.727394
A_68_P06632773	Fxyd7	0.72763
A_68_P09528972	Zfp454	0.728529
A_68_P06322596	Kcnj11	0.729121
A_68_P10569036	Cep72	0.729511
A_68_P11486688	Lect1	0.733807
A_68_P06143604	Nell1	0.734952
A_68_P10891937	Foxq1	0.736229
A_68_P03895474	Sesn2	0.73625
A_68_P11226211	Hs6st3	0.736393
A_68_P00012012	Klf7	0.736507
A_68_P13166532	Ddah2	0.736669
A_68_P04993451	Ptpn11	0.736854
A_68_P13382521	Notch4	0.737645
A_68_P04544688	Tmem129-Tacc3	0.738028
A_68_P04519890	Kctd10	0.738175
A_68_P11738050	Homez	0.738653
A_68_P04810796	Stk32b	0.739159
A_68_P07099607	BC056474	0.739524
A_68_P11989894	Ptger4	0.740476
A_68_P07178595	Sf3b3	0.741103
A_68_P10195731	Arg2	0.742162
A_68_P14645891	Gemin8	0.742614
A_68_P00488032	Clk1	0.742704
A_68_P12291916	BC026404:12631	0.742741
A_68_P07154161	Eif4ebp1	0.742875
A_68_P03484743	Pax7	0.743264
A_68_P06770253	Fanca	0.743514
A_68_P01193243	Clp1	0.743544
A_68_P13271870	Gtf2h4	0.743926
A_68_P02401082	Ccdc109b	0.745035
A_68_P04677545	Msx1	0.745201
A_68_P03328804	Ube2j2	0.745247
A_68_P10022213	Foxa1	0.745399
A_68_P03630403	Asah3l	0.74561

A_68_P13340774	2410015M20Rik	0.746131
A_68_P04552017	Dpp6	0.746241
A_68_P10801669	Mterfd1	0.746333
A_68_P00351468	Creg2	0.746835
A_68_P03332653	Foxe3	0.747046
A_68_P13090368	BC008155	0.747122
A_68_P06740725	2400003C14Rik	0.747455
A_68_P05344583	Tnfrsf1a	0.747548
A_68_P02280033	Mafb	0.748266
A_68_P01569256	Traf6	0.748417
A_68_P09596165	Rap2ip	0.749031
A_68_P11235588	Ccdc122	0.749449

Supplementary Table 7a: N vs A 560 hypomethylation genes on d16

Probe Name	Gene Name	Methylation difference value
A_68_P08365020	Tbc1d15	0.500869
A_68_P07008633	Casp3	0.505797
A_68_P03003821	Wnt2b	0.51527
A_68_P13848882	Dym	0.522551
A_68_P06140698	Arnt2	0.524945
A_68_P09691322	1200011M11Rik	0.527728
A_68_P14154116	Rbm4b	0.532604
A_68_P09444605	Car4	0.538766
A_68_P03872557	Tgfbr1	0.543096
A_68_P12490512	Adamts1	0.543226
A_68_P11097546	Sox4	0.547857
A_68_P09350088	Pmp22	0.549097
A_68_P14498430	Prkg1	0.552351
A_68_P01214289	Nmt2	0.557171
A_68_P13129521	Crebl1	0.558529
A_68_P01575537	Sfrs6	0.560647
A_68_P12654503	Rsl1d1	0.564131
A_68_P05149207	Ptcd3-Rpo1-4	0.56449
A_68_P02060727	Slc25a25	0.565203
A_68_P06001672	Foxi2	0.569377
A_68_P06536662	Ccdc106	0.574652
A_68_P11632577	Zfp395	0.577971
A_68_P08062569	Ei24	0.580041
A_68_P08848709	Kcnmb4	0.580792
A_68_P06164674	1810054G18Rik	0.581639
A_68_P02559788	Pitx2	0.581992
A_68_P06076158	Isoc2b	0.586721
A_68_P10987283	3110031B13Rik	0.58899
A_68_P07532442	Aph1b	0.589121
A_68_P08077709	BC024479	0.589562
A_68_P10286533	Cdca4	0.589872
A_68_P11399114	Apex1	0.590815
A_68_P11342429	Samd4	0.590989

A_68_P06824946	Foxf1a	0.591817
A_68_P09921275	Ubxd4	0.592623
A_68_P09172983	Sec14l4	0.593475
A_68_P01869178	Foxa2	0.59376
A_68_P09729901	Phb	0.59491
A_68_P00314170	Idh1	0.596116
A_68_P12412863	Osr2	0.598148
A_68_P06244528	Tufm	0.598853
A_68_P14410370	Nkx2-3	0.598946
A_68_P01360285	Rbpsuhl	0.599538
A_68_P08697303	Helb	0.599588
A_68_P01528714	Rpl12	0.600795
A_68_P07037395	Ap3m2-1700041G16Rik	0.601063
A_68_P10774084	Hist1h2bm	0.601549
A_68_P06052141	Unc45a-Hddc3	0.601628
A_68_P04106454	BC057022	0.60198
A_68_P07909840	Sin3a	0.602733
A_68_P03570669	Fabp3	0.602833
A_68_P14256098	Npas4	0.603714
A_68_P10243773	Bcl11b	0.603817
A_68_P05706787	Plxnd1	0.606406
A_68_P06573939	Kcna7	0.606601
A_68_P02577702	Sox2	0.60721
A_68_P05795898	Bhlhb3	0.609601
A_68_P06075074	Ntrk3	0.609628
A_68_P06352184	BC053749	0.610676
A_68_P00657988	Farslb	0.612033
A_68_P05972664	2210013K02Rik	0.612501
A_68_P03352387	BC057893	0.613578
A_68_P11484995	3632451O06Rik	0.614658
A_68_P05127678	Epha1	0.616216
A_68_P14720303	Dnase1l1-Taz	0.616454
A_68_P03556612	Mllt3	0.61666
A_68_P03899353	A430005L14Rik	0.616919
A_68_P13918711	1700034H14Rik	0.617072

A_68_P07296368	Sall1	0.617074
A_68_P06233151	Tmem126b	0.61718
A_68_P01436221	ENSMUST00000083577.1:189	0.61836
A_68_P07152542	Rab20	0.619436
A_68_P11726126	Fgf9	0.619572
A_68_P09739785	Per1	0.620738
A_68_P05156924	Dysf	0.621153
A_68_P12005671	Prpf40b	0.621408
A_68_P04677560	Msx1	0.622712
A_68_P09136741	Naglu	0.622956
A_68_P12223496	Kcnq3	0.623809
A_68_P09632522	Smurf2	0.624083
A_68_P06599561	Armc5	0.624283
A_68_P08649166	Stk11	0.624447
A_68_P07561476	2310005P05Rik	0.624928
A_68_P11724373	Ngdn	0.625187
A_68_P01674481	Nusap1	0.628065
A_68_P11173083	Abhd6	0.628118
A_68_P05488834	Tac1	0.62836
A_68_P06464534	Spnb4-Blvrb	0.628566
A_68_P03746961	Akr1a4	0.628687
A_68_P07901779	Htr1b	0.628812
A_68_P05899940	Qpctl-Snrpd2	0.628999
A_68_P13693986	Pou4f3	0.629258
A_68_P02286790	Crabp2	0.62966
A_68_P06096075	Adam12	0.630096
A_68_P10241916	Vsnl1	0.63036
A_68_P12016231	Serhl	0.631152
A_68_P11569601	Ebf2	0.631364
A_68_P05308590	Cav1	0.632142
A_68_P03022365	Rusc1	0.632663
A_68_P09525406	Hes7	0.632704
A_68_P01778274	Sema6d	0.63312
A_68_P05254993	Tra2a	0.633871
A_68_P00716966	Tnfrsf11a	0.634232

A_68_P14065754	Colec12	0.635747
A_68_P05006204	Hist4h4	0.63607
A_68_P09461692	1700020C11Rik	0.636428
A_68_P09073254	Vat1-Rnd2	0.636471
A_68_P01491127	Acvr2a	0.636508
A_68_P13182576	Abcg1	0.637155
A_68_P12672584	Polr2h	0.63736
A_68_P13331861	Tgif	0.637449
A_68_P03476394	Ssbp3	0.637874
A_68_P05727103	Npy	0.63871
A_68_P06273550	Adm	0.639223
A_68_P12837500	Cryzl1	0.639716
A_68_P12798251	Pak2	0.639724
A_68_P05795785	Shfm1	0.6399
A_68_P14408416	Ubtd1	0.640124
A_68_P09239666	1300013J15Rik	0.640203
A_68_P11069210	Shc3	0.640934
A_68_P10594937	Lysmd3	0.641373
A_68_P00936375	Lhx9	0.641751
A_68_P02074278	Frmd5	0.641907
A_68_P00051079	Sulf1	0.642317
A_68_P04677554	Msx1	0.642591
A_68_P12236506	Dap	0.642604
A_68_P01674488	Oip5	0.642932
A_68_P06544721	Rpl13a	0.644186
A_68_P05906990	Snx26	0.645498
A_68_P05827499	2500002L14Rik	0.645502
A_68_P09559156	Igfbp4	0.645884
A_68_P01864417	Brd3	0.646402
A_68_P09103595	Igf2bp1	0.64737
A_68_P04101451	Gsh2	0.647661
A_68_P09276835	Hoxb9	0.647898
A_68_P06369055	Arrdc4	0.648964
A_68_P09198633	Atp5h	0.649331
A_68_P07679467	Celsr3	0.650478

A_68_P07699697	Rtp3	0.650493
A_68_P03801846	Phf13	0.650911
A_68_P09084915	Gprc5c	0.652366
A_68_P05634149	Hoxa13	0.652827
A_68_P01947374	Sp9	0.653133
A_68_P09218218	Tnfaip1	0.653137
A_68_P01869679	Zdhhc5	0.654147
A_68_P05640423	Ldhb	0.654679
A_68_P03076435	Pde4dip	0.654979
A_68_P00415705	Hlx1	0.655652
A_68_P01451276	Casc4	0.656031
A_68_P07734331	Hyal2	0.656151
A_68_P11625862	Kcnma1	0.656939
A_68_P11246055	Fgf17	0.656969
A_68_P12707530	Chrd	0.657628
A_68_P14015396	Grp	0.658852
A_68_P11100963	Inhba	0.658978
A_68_P05606420	Hoxa13	0.65918
A_68_P05552662	Gabarapl1	0.65986
A_68_P05433825	Gm839	0.660255
A_68_P13882854	Reep2	0.660606
A_68_P01596218	Rasgrp1	0.660644
A_68_P05297811	4833442J19Rik	0.662314
A_68_P07021650	Dusp4	0.663313
A_68_P13396158	Dusp1	0.664528
A_68_P09434031	Cntnap1	0.664996
A_68_P12371495	BC038822	0.664996
A_68_P10682988	Hist1h4a	0.665724
A_68_P14396595	Aldh18a1	0.66577
A_68_P13076456	Smoc2	0.66595
A_68_P08007504	Ilf3	0.666054
A_68_P04108821	1110039B18Rik	0.666436
A_68_P05445839	Shfm1	0.666564
A_68_P04504710	Grsf1	0.666592
A_68_P12421448	Mchr1	0.66754

A_68_P02931199	Tspan5	0.667541
A_68_P13267430	Pcaf	0.667837
A_68_P01410881	Gca	0.667847
A_68_P04862538	Sema3a	0.667917
A_68_P01312041	2310002J15Rik-1110061N23Rik	0.668167
A_68_P04955575	Flt3	0.668682
A_68_P13300383	AW049765-E130307M08Rik	0.668916
A_68_P13413621	Ankrd47	0.66939
A_68_P02200717	Tspyl3	0.669536
A_68_P04768576	Nkx6-1	0.669538
A_68_P02016914	Itgav	0.670073
A_68_P11739620	Gata4	0.670078
A_68_P12310639	Hoxc8	0.670117
A_68_P09071666	Mgat1	0.670292
A_68_P04973104	Wipi2	0.670292
A_68_P08866199	Ascl1	0.670377
A_68_P05808766	Bhlhb2	0.670524
A_68_P10518868	Foxf2	0.671423
A_68_P03995832	Syf2	0.671539
A_68_P04937975	Ncor2	0.672068
A_68_P03938391	Pef1	0.672175
A_68_P09850298	Smoc1	0.672245
A_68_P05877424	Tspan33	0.672277
A_68_P02689603	Arnt	0.672748
A_68_P06165260	Mark4	0.673008
A_68_P06906180	Ap1m1	0.673236
A_68_P02571344	Cnn3	0.673371
A_68_P14281079	Psat1	0.673424
A_68_P06782036	Alg11	0.67361
A_68_P09485731	Tbx4	0.674532
A_68_P01232646	Cst3	0.674726
A_68_P08634266	Sf3a2	0.675215
A_68_P03115033	Gria2	0.675506
A_68_P11569567	Ebf2	0.6758
A_68_P14872362	Utx	0.675807

A_68_P03809665	Klf4	0.676694
A_68_P03801846	Phf13	0.676953
A_68_P01125928	Ubxd2	0.677638
A_68_P14081599	5730405I09Rik	0.678002
A_68_P08158875	9030425E11Rik	0.67822
A_68_P11446136	Bmp4	0.678515
A_68_P07318780	Gcsh	0.679011
A_68_P03946036	Ptplad2	0.679133
A_68_P08284610	Amd2	0.679558
A_68_P06965890	Bcnp1	0.679957
A_68_P12738265	Cggbp1	0.68004
A_68_P09495631	Stard3	0.680216
A_68_P09072145	Aatf	0.680382
A_68_P08369634	Erbb3	0.680497
A_68_P05885523	Dbx1	0.680708
A_68_P03946036	Ptplad2	0.681023
A_68_P04254946	Tmem60	0.681957
A_68_P04697706	Tcf1	0.682181
A_68_P13260931	M6prbp1	0.682267
A_68_P12324306	Gga1	0.682454
A_68_P09504070	AI842396-Kif1c	0.682728
A_68_P12047179	Sh3bp1	0.683064
A_68_P04899482	Brap	0.683247
A_68_P02653829	BC028528	0.683526
A_68_P14597813	Gabre	0.683889
A_68_P13916985	Gm672	0.684167
A_68_P02198060	Ralgps1	0.684502
A_68_P06900383	Ogfod1	0.684668
A_68_P14380925	Rfk	0.684832
A_68_P05345707	Magi1	0.68532
A_68_P14351720	Pdlim1	0.685997
A_68_P04940013	Barhl2	0.686593
A_68_P04199401	Gusb	0.686807
A_68_P09538308	Cabp7	0.686993
A_68_P03160430	Mab21l1	0.687447

A_68_P12329031	Dmc1	0.687655
A_68_P03476394	Ssbp3	0.687681
A_68_P13318888	Snx9	0.688054
A_68_P04861999	Cabp1	0.688321
A_68_P06873958	Ranbp10	0.688609
A_68_P02535516	Neurog2	0.689472
A_68_P13673765	Lipg	0.68948
A_68_P13426581	Tnfrsf12a-Cldn6	0.689585
A_68_P02979898	Arhgef2	0.68973
A_68_P04235482	Slc30a3	0.689762
A_68_P04899482	Brap	0.68989
A_68_P11138872	Samd4	0.690061
A_68_P06529209	Taldo1	0.690247
A_68_P06583791	Pvrl2	0.690496
A_68_P07163869	Gab1	0.690632
A_68_P09481065	Prr11-1110001A07Rik	0.690795
A_68_P00473416	Rnpepl1	0.690999
A_68_P13415559	Sepx1	0.691139
A_68_P14537219	Armcx1	0.691315
A_68_P03687831	Ermap	0.691422
A_68_P14414957	Ehbp1l1	0.691724
A_68_P09563953	Limd2	0.692163
A_68_P07634008	Foxb1	0.692189
A_68_P02976065	Shox2-Rsrc1	0.692519
A_68_P10729122	Hiatl1	0.692864
A_68_P04436077	Gc	0.693298
A_68_P07697209	Acaa1a	0.69351
A_68_P01927620	Neurod1	0.693881
A_68_P08830459	Arid5b	0.694079
A_68_P10504060	Ndufs4	0.694205
A_68_P07809714	Rbms3	0.694629
A_68_P04306213	Mapre3	0.694629
A_68_P09837413	Sfrs5	0.694724
A_68_P03328784	Ube2j2	0.694823
A_68_P09492659	3300001G02Rik	0.695006

A_68_P00626527	Atf3	0.695009
A_68_P14707230	EG546088	0.695087
A_68_P06121126	Mia1	0.695094
A_68_P06698299	Afg3l1	0.695367
A_68_P05982377	Cyp2r1	0.695895
A_68_P03191519	Mrpl47-Ndufb5	0.69594
A_68_P09135481	Upp1	0.696065
A_68_P04199401	Gusb	0.696109
A_68_P05078226	Mrpl53	0.696232
A_68_P13525249	Zfp811	0.696243
A_68_P03938391	Pef1	0.69663
A_68_P07533201	Snx22	0.696652
A_68_P09608031	Egfr	0.696691
A_68_P12860637	B830017H08Rik	0.696735
A_68_P05833901	Hk2	0.696761
A_68_P07428142	Irx3	0.696896
A_68_P00527441	Tagln2	0.697524
A_68_P10313512	Moap1	0.69761
A_68_P02953988	Hist2h4-Hist2h3c1	0.697915
A_68_P03788606	Pex14	0.698027
A_68_P03150605	Golph3l	0.698248
A_68_P11018873	Fcho2	0.699109
A_68_P12381245	Atf1	0.699572
A_68_P09422847	Atox1	0.69972
A_68_P08249585	Rbms2	0.699808
A_68_P02000475	Nkx2-2	0.700131
A_68_P14478339	Cyp26a1	0.700196
A_68_P05088298	Hipk2	0.700718
A_68_P06219580	Hnrpl	0.700966
A_68_P13417509	Angptl4	0.701027
A_68_P00241275	Rgs2	0.701132
A_68_P08954659	Kitl	0.701509
A_68_P10732850	Exoc3	0.701547
A_68_P13359212	Lrpprc	0.701832
A_68_P08544256	Metap2	0.701902

A_68_P07654201	Cd109	0.701985
A_68_P06204163	ENSMUST00000083675.1:-5771	0.701986
A_68_P14693838	Pcyt1b	0.702199
A_68_P04148386	Tmed5	0.702543
A_68_P09138750	Aloxe3	0.703034
A_68_P03959130	Bsdc1	0.703661
A_68_P12312345	Ppp1r16a	0.703681
A_68_P10243406	Rps6ka5	0.703686
A_68_P07464718	Pml-Stoml1	0.703735
A_68_P11831952	Elp3	0.703791
A_68_P03556612	Mllt3	0.703988
A_68_P03735990	Tmco4	0.704052
A_68_P04436077	Gc	0.704142
A_68_P10125273	Prkch	0.704315
A_68_P01676300	Kcna4	0.704552
A_68_P09483217	Hist3h2bb	0.705021
A_68_P03937865	Srrm1	0.705194
A_68_P10728416	Rgs7bp	0.705577
A_68_P10113786	Bcl11b	0.706075
A_68_P10382368	Rtn1	0.706168
A_68_P13297332	Slc37a1	0.706231
A_68_P05892280	Sbk1	0.706553
A_68_P11834677	Pcdh17	0.706664
A_68_P06666325	Pou2f2	0.70698
A_68_P09306104	Vdac1	0.707094
A_68_P00360280	Avpr1b	0.707122
A_68_P09906828	Zfp36l1	0.707259
A_68_P06573422	C230052I12Rik	0.707349
A_68_P12157937	Cacnb3	0.707365
A_68_P03619421	Olfr1333	0.707438
A_68_P00844646	Ptma	0.707779
A_68_P07155759	Ctcf	0.707854
A_68_P08881426	Zdhhc17	0.707885
A_68_P01733191	BC061194	0.708258
A_68_P13260914	M6prbp1	0.708735

A_68_P13565030	Stk38	0.709041
A_68_P06938696	Pard6a	0.709155
A_68_P00602343	Wdr42a	0.709258
A_68_P11212365	Il17rd	0.709292
A_68_P09341747	Senp3	0.709644
A_68_P00526290	Atg9a	0.709777
A_68_P13723745	Pcdhga5	0.709888
A_68_P06046859	Etv2	0.710364
A_68_P06592637	Cd3eap-Ppp1r13l	0.710376
A_68_P06620172	Fus	0.710591
A_68_P06193327	Dmpk	0.710606
A_68_P04460561	Sh2b3	0.710722
A_68_P12407265	Tef	0.7109
A_68_P09587090	A830091I15Rik	0.711225
A_68_P15054742	Eda2r	0.711397
A_68_P09719318	Ccdc57	0.711649
A_68_P06140421	Sars2	0.712033
A_68_P12142294	Abcd2	0.712177
A_68_P14961796	Trpc5	0.712345
A_68_P02271076	Rapgef1	0.712643
A_68_P08369621	Erbb3	0.712969
A_68_P09649890	Eif5a	0.712971
A_68_P04696354	Iqce-AA881470	0.71299
A_68_P10049453	Id2	0.713081
A_68_P09568808	Sfi1-Eif4enif1	0.713583
A_68_P13013615	Igsf4d	0.713589
A_68_P09208581	Cntd1	0.713647
A_68_P00325462	2810430M08Rik	0.713896
A_68_P04097979	Dnajc11	0.714675
A_68_P11303277	Spcs1	0.715692
A_68_P04874436	Stag3	0.715736
A_68_P08549923	Onecut3	0.715888
A_68_P05235141	Rarres2	0.716064
A_68_P09631349	Rhbdf1-Mpg	0.716151
A_68_P00083043	Slc19a2	0.716486

A_68_P02653832	BC028528-Aph1a	0.716491
A_68_P13349715	1700065O13Rik	0.716502
A_68_P01238387	Coq4	0.716863
A_68_P11211281	Pcdh17	0.717418
A_68_P05299879	Hoxa3	0.717437
A_68_P04507294	Slc4a2	0.718484
A_68_P09147607	Map2k3	0.719153
A_68_P09597706	Wipi1	0.719248
A_68_P10368309	Psma3	0.719357
A_68_P10098010	Atxn3	0.719405
A_68_P06481214	Ap2a1	0.719977
A_68_P08987557	Trp53i13-1300007F04Rik	0.720026
A_68_P04874436	Stag3	0.721061
A_68_P14501274	Syvn1	0.721299
A_68_P14732705	Uxt	0.721463
A_68_P12111891	Bai1	0.721656
A_68_P04326746	G3bp2	0.722289
A_68_P12730433	Igf2bp2	0.722387
A_68_P03682305	Grhpr	0.722881
A_68_P01478847	Ptges2	0.723062
A_68_P00964479	Fn1	0.723493
A_68_P12975687	Gsk3b	0.723497
A_68_P08277025	Cart1	0.723565
A_68_P08162873	Atp5l	0.723698
A_68_P02707342	Syt6	0.723886
A_68_P01807485	Camk1d	0.72416
A_68_P07869537	Cep70	0.724272
A_68_P04945721	Preb	0.724402
A_68_P11603330	Wbp4	0.724413
A_68_P05763460	Gars	0.724506
A_68_P09563820	Nudcd2	0.724731
A_68_P13626981	Spry4	0.724832
A_68_P08946365	Plxnc1	0.724899
A_68_P08082111	Keap1	0.724921
A_68_P11787452	Clybl	0.725011

A_68_P07561473	2310005P05Rik	0.725429
A_68_P09933404	Cdc42bpb	0.725828
A_68_P03418155	Ppap2b	0.72583
A_68_P03573506	Ccdc28b	0.72623
A_68_P00422171	Chrnd	0.726411
A_68_P05108088	Slc2a3	0.726488
A_68_P12149421	6-Mar	0.727106
A_68_P08660207	Myf6	0.727324
A_68_P02147288	Col5a1	0.727437
A_68_P01081579	Nmnat2	0.728256
A_68_P04429252	5730509K17Rik	0.728622
A_68_P07898845	Myd88-Acaa1a	0.728653
A_68_P06579870	Mesp2	0.728672
A_68_P13107839	Abcg8	0.728914
A_68_P07973186	Rasgrf1	0.729117
A_68_P09481071	1110001A07Rik	0.729414
A_68_P03814083	Rbm35a	0.729871
A_68_P09277242	Cyb561	0.730123
A_68_P03399684	Arhgef10l	0.730159
A_68_P10492119	Gas1	0.73053
A_68_P01264561	ENSMUST00000083577.1:-3950	0.730798
A_68_P05589325	Asb4	0.730841
A_68_P11680040	Cebpe	0.731039
A_68_P03133033	Serpini1	0.731075
A_68_P08237687	Tbc1d15	0.73133
A_68_P09442531	3010026O09Rik	0.731365
A_68_P04432506	Lhx5	0.731476
A_68_P02649135	Slc39a8	0.731495
A_68_P05843901	Hoxa11	0.731793
A_68_P02138996	Slc12a6	0.731802
A_68_P11613188	Pou4f1	0.731862
A_68_P12371349	A_68_P12371349	0.732008
A_68_P09880659	Acot1	0.732066
A_68_P08992028	Pnpo	0.732344
A_68_P04768755	Actb	0.732382

A_68_P01761801	Raly	0.73285
A_68_P03206275	Rnf20	0.733172
A_68_P12891400	Hunk	0.733639
A_68_P12233048	H1f0	0.733664
A_68_P09550136	Etv4	0.733755
A_68_P02766324	Vps45	0.734077
A_68_P10202514	Aldh6a1	0.73435
A_68_P01489913	Bmi1	0.734359
A_68_P03043732	Fndc3b	0.734785
A_68_P03948130	2900042B11Rik	0.73506
A_68_P11911301	Fkbp11	0.735179
A_68_P06473598	Dedd2-Zfp526	0.735537
A_68_P14251712	BC032204	0.735569
A_68_P09289132	Pipox	0.735642
A_68_P14426428	Vldlr	0.735792
A_68_P09049858	Wnk4	0.73588
A_68_P08401341	Rnf126-Fstl3	0.735989
A_68_P08272296	E130306M17Rik	0.736003
A_68_P05958412	Lmo1	0.736244
A_68_P12557377	Chrd	0.736478
A_68_P08109654	Ankrd25	0.736486
A_68_P04926196	Fzd10	0.736739
A_68_P09304806	Stac2	0.736974
A_68_P02438391	Mrps21	0.73707
A_68_P05383480	Dync1i1	0.737648
A_68_P05104710	Wnt2	0.737768
A_68_P03560551	Frmd3	0.737819
A_68_P03841228	Zfp593	0.738007
A_68_P13187383	Riok2	0.738185
A_68_P08314939	Oaz1	0.738252
A_68_P03776199	Lyn	0.738776
A_68_P07533275	Rpsa	0.738983
A_68_P09467840	Sec14l2	0.739131
A_68_P13472930	Lama1	0.73922
A_68_P13832401	Pcdhgb1	0.739239

A_68_P09736143	Ush1g	0.739376
A_68_P14281775	Emx2	0.739497
A_68_P03676855	Zfp189	0.7395
A_68_P01403762	Cat	0.739656
A_68_P12042664	Cdc42ep1	0.739659
A_68_P05860624	Bid	0.739662
A_68_P07561953	Aph1c	0.739677
A_68_P04793013	2700038N03Rik	0.739793
A_68_P10580665	Hist1h2bk	0.740135
A_68_P05519173	Mkln1	0.740277
A_68_P05057768	Hk2	0.741122
A_68_P03888951	Mllt3	0.741147
A_68_P07231075	Tomm20	0.741439
A_68_P04671642	Unc84a	0.741594
A_68_P07748509	Rpp25	0.74173
A_68_P03619421	Olfr1333	0.741823
A_68_P04363207	Kntc1	0.741833
A_68_P09694614	Lsmd1	0.741962
A_68_P09042588	Tcf2	0.742079
A_68_P04629383	Pitpnm2	0.742114
A_68_P10022016	Mamdc1	0.74217
A_68_P11465793	Tsc22d1	0.742228
A_68_P03230060	Frap1	0.742246
A_68_P08698911	Naca	0.742353
A_68_P14407497	Scd2	0.742518
A_68_P14998770	Rpl36a	0.742596
A_68_P07561489	2310005P05Rik	0.742667
A_68_P13194682	Cfb	0.742776
A_68_P08200250	Ipmk	0.742925
A_68_P05599948	C1r	0.74305
A_68_P07353001	Upf1	0.743093
A_68_P07945856	Cdv3	0.743102
A_68_P02272810	Plcb1	0.743121
A_68_P09559259	Ngfr	0.743245
A_68_P08428861	Stat6	0.743478

A_68_P04534962	Triap1	0.743704
A_68_P11564466	9430077A04Rik	0.743751
A_68_P02033228	A930012O16Rik	0.743871
A_68_P10478798	Foxf2	0.743999
A_68_P04691965	Ogfod2	0.744179
A_68_P00844649	Ptma	0.74434
A_68_P13242268	Bat3	0.744418
A_68_P09080012	Gtlf3b	0.744614
A_68_P08269534	Cs	0.744634
A_68_P00991622	Capn8	0.744674
A_68_P11400722	Rem2	0.744709
A_68_P06928687	Got2	0.744755
A_68_P00127657	Wnt6	0.744911
A_68_P15153209	D0HXS9928E	0.745325
A_68_P08237398	Pa2g4	0.745513
A_68_P11886679	Itga5	0.746148
A_68_P01845807	Sephs1	0.746187
A_68_P00516296	Nit1	0.746433
A_68_P12635594	Ehhadh	0.746435
A_68_P13899746	Epb4.1l4a	0.746769
A_68_P14757489	Zcchc12	0.747434
A_68_P02294720	Lef1	0.747676
A_68_P03666611	Astn2	0.747785
A_68_P10743304	Hist1h3g	0.747804
A_68_P13240096	Rpl7l1	0.74782
A_68_P03068794	Kcnmb2	0.74788
A_68_P00563810	Mgst3	0.748033
A_68_P09379018	Mnt	0.748343
A_68_P12019183	Scrt1	0.74851
A_68_P01933643	Ggta1	0.748572
A_68_P00317122	Cxcr4	0.748644
A_68_P05259006	Tpi1	0.748674
A_68_P13230250	Ankrd12	0.748925
A_68_P07872982	ENSMUST00000034643.3:-4289	0.749137
A_68_P01853107	Zbtb43	0.7495

A_68_P02517306	Efna4	0.749648
A_68_P02241425	Inoc1	0.749996

Supplementary Table 7b: N vs A 409 hypermethylation genes on d16

Probe Name	Gene Name	Methylation difference value
A_68_P01442677	Adam33	2.34309
A_68_P14809906	Ldoc1	2.182542
A_68_P06186495	Lrrc27	2.09403
A_68_P13883453	Pcdha8	2.056548
A_68_P12138338	Hoxc13	2.016236
A_68_P10815405	Sfxn1	2.004238
A_68_P03540115	Espn-Hes2	2.000182
A_68_P13883460	Pcdha9	1.960946
A_68_P04945718	Preb-Tcf23	1.951468
A_68_P04945718	Preb-Tcf23	1.928502
A_68_P11534471	Lgals3	1.909895
A_68_P14270379	Nt5dc3	1.895435
A_68_P07737935	Fxyd2	1.891944
A_68_P02189755	Hrh3	1.887435
A_68_P00795918	Srp9	1.887293
A_68_P06376689	Npas1	1.885412
A_68_P02421072	Dnajb4-Fubp1	1.880806
A_68_P12773755	EG328644	1.874945
A_68_P04101457	Gsh2	1.834503
A_68_P05495854	Casd1	1.805508
A_68_P07425330	Arrdc2	1.80279
A_68_P01139487	Wnt6	1.790663
A_68_P01668312	E2f1	1.787956
A_68_P09804391	Siva1	1.787582
A_68_P12268481	Aqp2	1.771162
A_68_P01717991	Btbd14a	1.769291
A_68_P09165274	Nxn-Timm22	1.745295
A_68_P04346888	Ppp2r2c	1.741594
A_68_P03400995	Txn1	1.734468
A_68_P09177324	Olfr30	1.732713
A_68_P03974013	Gja4	1.729471
A_68_P01498685	A430105I19Rik	1.724954
A_68_P02564830	Igsf3	1.724518

A_68_P10614127	Ror2	1.722767
A_68_P12751159	B830017H08Rik	1.70917
A_68_P03228980	Acot7	1.707831
A_68_P08143591	Pdcd6ip	1.700578
A_68_P06208098	Hras1	1.698275
A_68_P13127671	Sox8	1.697824
A_68_P09018398	Gprc5c	1.682796
A_68_P03301110	Galnt12	1.678821
A_68_P12097344	Krt73	1.673928
A_68_P13365866	Ihpk3	1.670431
A_68_P05370614	Lrrc23	1.668623
A_68_P12068740	Pp11r	1.6674
A_68_P05278653	Capza2	1.660087
A_68_P08799615	Cdc2l6	1.656491
A_68_P13291042	Fkbpl	1.655958
A_68_P08137775	Trim29	1.644358
A_68_P13191224	Sema6b	1.642482
A_68_P07663357	Megf11	1.638227
A_68_P10027436	Rgs6	1.635025
A_68_P06318305	Igf1r	1.633508
A_68_P02779323	Trim45	1.626551
A_68_P08717087	Tcba1	1.625998
A_68_P04964439	Tmem132d	1.624712
A_68_P01383195	B230120H23Rik	1.617043
A_68_P14275989	Tbc1d12	1.61573
A_68_P09922529	Ptprn2	1.612314
A_68_P11941919	EG432987	1.612227
A_68_P09483960	Cxcl16	1.611037
A_68_P05970860	Phox2a	1.607537
A_68_P06152331	Bcl3	1.599857
A_68_P02483040	Ppa2	1.589217
A_68_P06926631	A230052G05Rik	1.587101
A_68_P00505719	3322402L07Rik	1.584498
A_68_P06565632	Lrrc27	1.584281
A_68_P05033637	Ltbr	1.583232

A_68_P04646216	BC004044	1.579388
A_68_P12117966	LOC554292	1.575279
A_68_P06983908	5033428A16Rik	1.570745
A_68_P13365875	Ihpk3	1.564213
A_68_P09949390	Btbd7	1.558795
A_68_P11215454	Olfm4	1.557819
A_68_P07897281	Rdx	1.554041
A_68_P03405759	Txlna	1.553528
A_68_P09513483	Vmo1	1.552141
A_68_P03400995	Txn1	1.549803
A_68_P03300156	Anp32b	1.549295
A_68_P12240046	Eppk1	1.544518
A_68_P06664663	Tex101	1.542541
A_68_P08660202	Myf6	1.540616
A_68_P08449896	Foxo3a	1.538192
A_68_P09309985	Krt35	1.534781
A_68_P10614129	Ror2	1.532037
A_68_P13176689	Cul7	1.53041
A_68_P00119253	B4galt3	1.52864
A_68_P01159756	Hrh3	1.528635
A_68_P13075872	Mas1-Mrgprh	1.528059
A_68_P11680943	Entpd4	1.525934
A_68_P00331311	EG240916	1.525499
A_68_P13496188	Traf7	1.52355
A_68_P11932638	Cacnb3	1.522527
A_68_P10512750	Thbs4	1.521117
A_68_P11855904	Ly6d	1.515977
A_68_P03819458	Rbp7	1.515733
A_68_P14243676	1700019N19Rik	1.515392
A_68_P11650775	Pnoc	1.514185
A_68_P11253316	Slc22a17	1.513158
A_68_P09017696	Krt42	1.510214
A_68_P11899315	BC024139	1.509074
A_68_P12129679	Csad	1.50561
A_68_P13927246	Pcdhac2	1.504887

A_68_P14346771	Ms4a7	1.504333
A_68_P12129693	Csad	1.503652
A_68_P06871160	Bst2-1110012M11Rik	1.500908
A_68_P12093074	Arhgap8	1.500185
A_68_P05596978	Rnf103	1.49965
A_68_P03625246	Zswim5	1.49615
A_68_P07871025	Htr1b	1.496005
A_68_P03300156	Anp32b	1.495502
A_68_P05904416	Klk15	1.495005
A_68_P07129218	Pkd1l2-Bcmo1	1.494286
A_68_P03555189	Bai2	1.49364
A_68_P08045042	Cacna2d2	1.493452
A_68_P11281788	2600011E07Rik	1.491899
A_68_P06631626	Wtip	1.491517
A_68_P04646216	BC004044	1.491433
A_68_P08675788	Slc17a8	1.490621
A_68_P03974014	Gja4	1.489772
A_68_P08980029	Krt27	1.487658
A_68_P12092825	Sstr3	1.486341
A_68_P06387117	Hdgfrp3	1.485299
A_68_P01757681	Creb3l1	1.481582
A_68_P01334676	9230107O10Rik	1.476058
A_68_P04331232	Dtx1	1.476011
A_68_P03370361	Cer1	1.474334
A_68_P01979586	Mmp24	1.473049
A_68_P04318728	Pebp1	1.472847
A_68_P01295326	Lamc3	1.471722
A_68_P04308903	Areg	1.470039
A_68_P10356757	Trim9	1.46986
A_68_P01619771	Insm1	1.462248
A_68_P09583142	P2rx1	1.461627
A_68_P07024704	1700029J07Rik	1.459602
A_68_P07559432	D930028F11Rik	1.459294
A_68_P07453357	Cd109	1.457014
A_68_P05893671	6330512M04Rik	1.45689

A_68_P13977860	Pcdhga5	1.456032
A_68_P01531594	Mdk	1.455592
A_68_P03361261	Ubxd3	1.454801
A_68_P05590714	Ret	1.453914
A_68_P14323852	Psd	1.451816
A_68_P07400685	Vac14	1.451249
A_68_P10324890	Dio3	1.451129
A_68_P06751294	Slc12a3	1.45073
A_68_P10739130	Hrh2	1.45014
A_68_P01887581	Shc4	1.447136
A_68_P11650789	Pnoc	1.445781
A_68_P07423734	Slc7a5	1.444347
A_68_P04873423	Kcnh2	1.44317
A_68_P05804599	Mrpl19	1.441819
A_68_P11971534	Kcns2	1.439005
A_68_P10573446	Larp5	1.435129
A_68_P09156504	Mfap4	1.434511
A_68_P13293615	Gbl	1.433212
A_68_P12461149	Krt2-25	1.433036
A_68_P12233648	Osmr	1.432645
A_68_P03075997	Dcamkl2	1.432491
A_68_P12323106	Jrk	1.431737
A_68_P13553860	BC031441-Ebi3	1.431056
A_68_P01654378	Cstl1	1.431036
A_68_P10096901	Bdkrb1	1.428969
A_68_P00789268	Myog	1.428938
A_68_P06096064	Adam12	1.427533
A_68_P05936205	Ifitm5	1.426306
A_68_P04854579	Hspb8	1.425306
A_68_P05372329	8430419L09Rik	1.425188
A_68_P04799896	Trim56	1.424642
A_68_P07453364	Cd109	1.42401
A_68_P14156202	Pdcd4	1.423262
A_68_P04506925	Gpr81	1.422663
A_68_P12438489	Sfrs2ip	1.422363

A_68_P12224465	Fbln1	1.421897
A_68_P06422662	Dmkn	1.421556
A_68_P05596974	Rnf103	1.421527
A_68_P00556266	Ctdsp1	1.420307
A_68_P03970943	Espn	1.420068
A_68_P09611938	Havcr2	1.418968
A_68_P10151513	Psma6	1.41846
A_68_P04799035	Nsg1	1.418253
A_68_P13103833	Smoc2	1.418214
A_68_P13166506	Ddah2	1.418187
A_68_P02009521	1190002A17Rik	1.417585
A_68_P03709353	Plcl4	1.417371
A_68_P03899301	Zbtb8	1.417202
A_68_P12362731	Mfsd3	1.416568
A_68_P11906547	Bai1	1.416568
A_68_P13385328	4930546H06Rik	1.416153
A_68_P12406891	Aqp6	1.415879
A_68_P03568532	Tspan1	1.415876
A_68_P08210289	9-Mar	1.415831
A_68_P00451766	Nmnat2	1.415778
A_68_P09631017	Csf2	1.414005
A_68_P07173287	EG13909	1.413959
A_68_P04425904	Wasf3	1.413345
A_68_P13190929	Zbtb9	1.413282
A_68_P11308601	Tgm1	1.412908
A_68_P12215401	Sstr3	1.412418
A_68_P07215117	Sfrp1	1.412338
A_68_P00528102	A130010J15Rik	1.412154
A_68_P03553498	Espn	1.41195
A_68_P12462764	Top1mt	1.411599
A_68_P02716554	Adh1	1.411018
A_68_P11658283	Kcnk5	1.41094
A_68_P07822241	Nr2e3	1.410808
A_68_P12323097	Jrk	1.410734
A_68_P09677979	Krt35	1.409825

A_68_P07565599	Gnb5	1.408292
A_68_P04316294	Mapre3	1.407638
A_68_P06633818	Gm1082	1.405985
A_68_P00993107	Lemd1	1.405865
A_68_P03413661	Padi4	1.404845
A_68_P13166533	Ddah2	1.404057
A_68_P06728122	Enpp6	1.403461
A_68_P12460882	Yaf2	1.402574
A_68_P14131435	Cdx1-Pdgfrb	1.402354
A_68_P02865901	Mef2d	1.402333
A_68_P05226947	Abcc9	1.401101
A_68_P03912407	Aqp7	1.400689
A_68_P06053429	Tssc4	1.400163
A_68_P06408617	Lsp1	1.400086
A_68_P06549860	2310033E01Rik	1.399809
A_68_P14605117	1700045I19Rik	1.399771
A_68_P00363449	Teddm1	1.399699
A_68_P12341775	Plec1	1.399275
A_68_P03854965	Marcksl1	1.398753
A_68_P12111323	Plec1	1.398476
A_68_P05938143	Klk15	1.398238
A_68_P07195280	Comp	1.397532
A_68_P08963632	Slc5a4a	1.397503
A_68_P09107785	Tcap	1.397479
A_68_P14261086	Syt7	1.39648
A_68_P09620647	Dcxr	1.395695
A_68_P00169777	Ikbke	1.395683
A_68_P09444804	Pyy	1.395386
A_68_P07319284	Rrad	1.39505
A_68_P05958473	Tmem16e	1.394619
A_68_P03457502	Zmynd12	1.394521
A_68_P02023974	1110061N23Rik	1.394486
A_68_P09459558	Tcf7	1.393455
A_68_P03226841	Pla2g2c	1.392659
A_68_P12298037	Lynx1	1.392549

A_68_P03448458	Sesn2	1.392427
A_68_P07820591	Pcbp4	1.39238
A_68_P02501124	Nexn	1.391383
A_68_P03720734	Mmel1	1.391196
A_68_P09483427	Sfrs2-2600014M03Rik	1.390894
A_68_P00444553	Darc	1.390467
A_68_P03909943	Arid1a	1.389192
A_68_P05972322	Xylt1	1.388761
A_68_P05244159	ENSMUST00000083652.1:2772	1.388054
A_68_P04528236	Trim50	1.387044
A_68_P01512424	Nr5a1	1.386465
A_68_P06129246	Ptgir	1.386124
A_68_P00052299	Gpc1	1.385347
A_68_P12273277	Acvrl1	1.384572
A_68_P06544745	Rpl13a	1.384276
A_68_P05208549	Dync1i1	1.383989
A_68_P09006081	Hoxb4	1.383845
A_68_P04543788	Epo	1.383701
A_68_P13658376	LOC639653	1.382952
A_68_P11939489	Zfp251	1.382311
A_68_P13396934	Pspn	1.382199
A_68_P01606835	Prnd	1.382113
A_68_P06620448	5430432N15Rik	1.381823
A_68_P09624543	Krt19	1.381113
A_68_P07317287	Aktip	1.380563
A_68_P03287181	C1qdc2	1.380508
A_68_P08800400	Polr3b	1.380063
A_68_P01655397	BC050777	1.379852
A_68_P02071364	Wisp2	1.379713
A_68_P13697471	Pcdha5	1.379344
A_68_P13456368	H2-M10.1	1.379336
A_68_P02989352	Dcamkl2	1.379329
A_68_P01781180	Bcas1	1.378844
A_68_P12298006	Lynx1	1.377705
A_68_P05879159	Suclg1	1.377306

A_68_P03413661	Padi4	1.376063
A_68_P04324543	Nat8l	1.376043
A_68_P07349274	Rbm13	1.375987
A_68_P09309975	Krt35	1.375793
A_68_P07506801	Cck	1.375411
A_68_P03448375	Tnfsf8	1.37474
A_68_P13467225	Arhgap28	1.374529
A_68_P04873423	Kcnh2	1.374163
A_68_P04222183	Smarcd3	1.373947
A_68_P05772946	Il17ra	1.373931
A_68_P07615452	Mst1r	1.373718
A_68_P04502132	Cldn13	1.372323
A_68_P09926896	2810002I04Rik	1.372186
A_68_P06865553	Chrnb3	1.371884
A_68_P10096903	Bdkrb1	1.370888
A_68_P04966920	Rpl21	1.37029
A_68_P03974035	Gja4	1.369796
A_68_P12092110	Krt79	1.36939
A_68_P11438500	D14Ertd449e-C330003B14Rik	1.368955
A_68_P09536877	Pnmt	1.368951
A_68_P07349292	BC025816	1.368751
A_68_P13058565	Slc22a2	1.368585
A_68_P13196755	Grm4	1.368241
A_68_P11528226	BC055107	1.367413
A_68_P03909943	Arid1a	1.366859
A_68_P09168909	Ngfr	1.366606
A_68_P13568346	Ly6g6c	1.366322
A_68_P01275517	Urm1	1.366155
A_68_P05617222	Fxyd4	1.365609
A_68_P07682087	ENSMUST00000083662.1:-4036	1.365597
A_68_P09448685	2210403B10Rik	1.364972
A_68_P11230853	Jub	1.364946
A_68_P10355900	Ckb-6720458F09Rik	1.363708
A_68_P03899398	N28178	1.363475
A_68_P12441858	Adamts20	1.363237

A_68_P00661821	Cnnm4	1.362611
A_68_P01782935	Ggtl3	1.362086
A_68_P12193009	Krt79	1.361886
A_68_P11042513	4930544M13Rik	1.360955
A_68_P04966043	0610009O03Rik	1.359467
A_68_P03964165	Gnl2	1.358997
A_68_P04326186	Sh3tc1	1.358857
A_68_P09218102	Dock2	1.358572
A_68_P01875370	Kcnb1	1.35788
A_68_P04346888	Ppp2r2c	1.357859
A_68_P09658179	Bahcc1	1.357685
A_68_P15185453	Tmem47	1.357529
A_68_P02034225	Itga6	1.357451
A_68_P01772352	Lcn12	1.356836
A_68_P12437042	Mapk15	1.356723
A_68_P07682089	ENSMUST00000083662.1:-4369	1.356568
A_68_P07334122	Exoc3l-E2f4	1.356412
A_68_P09611503	Doc2b	1.356372
A_68_P05952684	Bnc1	1.356203
A_68_P08138672	AI593442	1.356081
A_68_P07422544	Cpne7	1.355603
A_68_P05531486	Gdf3	1.355337
A_68_P02238998	Osbpl2	1.354655
A_68_P14339787	Adra2a	1.354605
A_68_P07950325	Sh3px3	1.354485
A_68_P05585260	Hrh1	1.354414
A_68_P08514482	Tac2	1.353772
A_68_P08782402	Jsrp1	1.353675
A_68_P09240367	Tbkbp1	1.352878
A_68_P09105482	Kcnip1	1.352624
A_68_P13449298	Scube3	1.35262
A_68_P07506791	Cck	1.35248
A_68_P07411349	Rab3a	1.352312
A_68_P11920360	Krt75	1.352057
A_68_P13429542	Pbx2	1.352009

A_68_P01092822	Fmo1	1.35155
A_68_P01220646	4833422F24Rik	1.350428
A_68_P01910329	Grb14	1.35011
A_68_P10014745	Pnma1	1.349738
A_68_P06641066	Rpl18	1.349681
A_68_P09263154	Mmp28-Taf15	1.349494
A_68_P13271868	Gtf2h4	1.349317
A_68_P04114789	Add1	1.348994
A_68_P09485551	Mafg	1.348189
A_68_P02026624	Tmem62	1.347905
A_68_P03135772	Mab21l2	1.347672
A_68_P05915391	1700008P20Rik	1.347339
A_68_P00634491	Tmem58	1.347231
A_68_P11567764	4930578I06Rik	1.34677
A_68_P07351402	Rab20	1.346479
A_68_P04355011	P2rx2	1.346273
A_68_P01267327	Trim44	1.346088
A_68_P04163384	Steap1	1.345586
A_68_P08300127	Olfr796	1.34554
A_68_P03502654	Aldh1b1	1.344962
A_68_P09645952	OTTMUSG00000000934	1.344829
A_68_P02752639	Hist2h2be	1.344661
A_68_P07530192	Col7a1	1.344185
A_68_P01249319	Sephs1	1.34342
A_68_P04808471	Ephb4	1.343316
A_68_P12074308	Galr3	1.343114
A_68_P09545756	Zfp454	1.343003
A_68_P13405326	Srbd1	1.342727
A_68_P06924600	BC051227	1.342376
A_68_P05547809	Lrrc23	1.342008
A_68_P03568532	Tspan1	1.341324
A_68_P10354469	Jag2	1.340861
A_68_P10060189	1200009I06Rik	1.340258
A_68_P12457546	2310016F22Rik	1.340194
A_68_P08979894	Krt42	1.339262

A_68_P07470088	Cdkn2d	1.339244
A_68_P08514391	Gng7	1.339227
A_68_P09072868	Pitpnm3	1.338663
A_68_P13150762	Clic1	1.338163
A_68_P09124054	Gfap	1.338131
A_68_P08888274	Cd63	1.338058
A_68_P05299461	Cpa1	1.337323
A_68_P01469129	Rem1	1.337275
A_68_P07847706	Gpr83	1.33646
A_68_P12177088	Ly6k	1.336186
A_68_P03679600	Tas1r2	1.336058
A_68_P03670798	Masp2	1.335352
A_68_P07005244	Best2	1.334757
A_68_P09042040	ENSMUST00000021240.4:169	1.334682
A_68_P07089318	Foxl1	1.33413
A_68_P13450793	Zbtb22	1.334052
A_68_P03495256	Ephb2	1.334023
A_68_P00815120	Wdr26	1.333762
A_68_P09104090	C1qtnf1	1.333483
A_68_P10126148	Lamb1-1	1.332216
A_68_P12423107	Adm2	1.331559
A_68_P07166167	Ndrg4	1.331269
A_68_P03153566	Mcl1	1.33114
A_68_P01498090	Plcg1	1.330655
A_68_P12476467	Pigp	1.330322
A_68_P04528236	Trim50	1.330284
